# COVID-19 alert level systems—Lessons learnt for future public health emergencies: A qualitative study

**DOI:** 10.1371/journal.pone.0351209

**Published:** 2026-06-18

**Authors:** Hana Rohan, Aaron F. Bochner, Lauren Brown, Elizabeth M. Ortiz, Amanda McClelland

**Affiliations:** 1 Georgetown University, Washington, District of Columbia, United States of America; 2 Resolve to Save Lives, New York, New York, United States of America; University of Wollongong, AUSTRALIA

## Abstract

**Background:**

During the COVID-19 pandemic, Alert Level Systems (ALS) were widely implemented as public health tools to communicate risk levels and recommend public health and social measures (PHSMs). However, the efficacy of ALS in mitigating disease spread and their impact on public health responses have not been systematically evaluated. This study aims to assess perceptions of ALS implementation across diverse jurisdictions and derive lessons for future public health emergencies.

**Methods:**

Key informant interviews were conducted remotely between December 2023 and March 2024 with senior stakeholders who were involved in ALS development and implementation during the COVID-19 pandemic, from eight jurisdictions: California (US), New Zealand, the Philippines, Rio Grande do Sul (Brazil), Singapore, South Africa, the United Kingdom, and the United States. A thematic analysis approach was applied to synthesize insights, focusing on the strengths, challenges, and key lessons from ALS implementation.

**Results:**

ALS were generally perceived by key informants as useful tools for communicating risk and supporting adherence to PHSMs due to their simplicity and transparency. However, significant challenges were identified, including difficulties in accessing reliable data, lack of clear ALS objectives, and insufficient community engagement. The study highlights the need for ALS to integrate social, economic, and epidemiological data in decision-making processes. Jurisdictions also reported that pre-existing ALS governance structures and stronger community feedback mechanisms could have improved implementation outcomes.

**Conclusions:**

ALS can serve as valuable public health communication tools in future epidemics, but their success depends on clear objectives, evidence-based PHSMs, and robust community engagement. Pre-emptive development of ALS structures and governance will improve preparedness for future epidemics. Transparent and flexible decision-making processes will be crucial for sustaining public trust.

## Introduction

During public health emergencies, it is typically necessary to communicate to the public changing levels of risk, along with recommended public health and social measures (PHSMs) to mitigate these risks. Alert Level Systems (ALS) are an emergency preparedness and response tool, often including colour-coded and/or numbered levels, used to centralize and simplify communication to inform individuals, communities, organizations, and/or decision-makers of their current risk of a particular hazard.

ALS were widely used during the COVID-19 pandemic both to communicate risk and translate policy into recommended actions, [1–7] and to facilitate internal assessments of risk by decision-makers. COVID-19 ALS were usually linked to recommended or mandated measures to reduce or mitigate transmission risk. As risk heightened, alert levels increased, and guidance or measures increased in stringency and number; as risk abated, levels decreased and measures were relaxed. [8] COVID-19 ALS typically relied on data produced by surveillance systems to underpin assessments of risks, set alert levels, and help assign appropriate PHSMs and other interventions.

While ALS are widely used in other ‘warning science’ areas (e.g., weather hazards, terrorism, natural disasters), [9] the COVID-19 pandemic was the first time such systems were utilised widely in a public health context. [10] Despite the widespread use of ALS during the pandemic, there is limited available documentation, evaluation data, or research on their use and impact. This research therefore aimed to utilise key informant interviews with senior stakeholders who participated in the development and implementation of ALS during the COVID-19 pandemic to document and synthesise perceptions around the perceived usefulness and implementation effectiveness of ALS in their jurisdictions, and identify key lessons learnt that have applicability for the use of ALS in future epidemics.

## Methods

Data collection consisted of key informant interviews with senior technical staff involved with the design or implementation of ALS in their jurisdictions. Case study jurisdictions were identified using a purposive approach utilising a review of the peer-reviewed and grey literature as well as the research team’s existing knowledge and networks. Jurisdictions were selected to provide a range of geographic diversity, epidemic experience, income level, and type of ALS implemented.

Key informants from study jurisdictions were identified purposively through publicly available document review as well as snowball sampling drawing initially from the existing networks of the project’s investigators. Identified key informants were invited to participate in an in-depth interview, and were also asked to recommend other potential participants from within their organisations to participate in the study.

Jurisdictions included in this review of ALS implementation experiences were: California, USA; New Zealand; The Philippines; Rio Grande do Sul, Brazil; Singapore; South Africa; United Kingdom; and the United States. All identified key informants from the above jurisdictions worked within Ministries of Health or associated agencies during the implementation of COVID-19 ALS. A total of 18 key informants were interviewed remotely between December 20^th^ 2023 and March 14^th^ 2024. Interviews lasted approximately 60–90 minutes. A list of their positions is available at S1 Appendix. Due to the seniority of identified key informants and the relatively small numbers of appropriate key informants available in each jurisdiction, data are presented without attribution to job title to ensure confidentiality.

Interviewees for all but one jurisdiction were conducted in English. Interviews with key informants from Brazil were conducted in Portuguese and transcripts were translated into English. Interviews were auto-transcribed, with interviewees’ permission, using Artificial Intelligence (AI) transcription software. Transcripts were subsequently reviewed alongside audio recordings and any transcription mistakes were corrected.

### Data analysis

Transcripts were imported into NVivo where they were analysed using a thematic analysis framework approach, [11] with codes derived from the interview topic guide assigned to lines of text in a small sample of interviews. Following review of the initial coding, a working analytical framework made up of codes and categories was applied to the remaining transcripts, while allowing for emerging themes and concepts in later transcripts to be coded and categorised. Coding was conducted by the lead researcher. Emerging themes and the evolving analytical framework were discussed periodically with members of the research team to support reflexivity and analytical coherence. Coding proceeded iteratively, with refinement of categories as analysis progressed. No substantively new themes emerged in later interviews, suggesting thematic saturation within the scope of the study.

After completion of all coding, the key categories relating to ‘lessons learnt’ were further developed to form the basis of the results section of this paper.

### Ethical considerations

The evaluation protocol was approved by the Resolve to Save Lives Research Committee, which determined that it met the criteria for minimal risk and was exempt from Institutional Review Board review. Potential participants were contacted individually via email and provided with a Participant Information Sheet describing the evaluation to inform their decision to participate. All key informants provided their consent to participate verbally or via email prior to commencing interviews. Transcripts were de-identified and anonymised, with only the lead researcher having access to a password protected spreadsheet on a local laptop linking interviewees to transcripts.

## Results

Overall, key informants perceived ALS to be useful tools for public communication of risks, due to their simplicity and ease of interpretation, the way they facilitated a perception of response transparency, and their perceived contribution to supporting epidemic control efforts. Examples of ALS used during the pandemic are available at S2 Appendix. None of the participating jurisdictions had formally evaluated the effectiveness of their systems, or systematically monitored their efficacy during implementation, largely as a result of the emergent context in which most ALS were incepted and the pace of their rollout and implementation. As a result, key informants relied on their own observations and perceptions of efficacy and challenges associated with ALS implementation.

The sections below present lessons learnt derived from the perceptions and experiences of the key informants interviewed for this study. While presented separately for clarity, these themes were often interrelated in practice. Lessons learnt have been organised into four thematic areas: 1) Identifying data and setting objectives for ALS; 2) setting PHSMs associated with ALS; 3) community engagement, trust, and credibility; and 4) decision-making processes.

### Identifying data and setting objectives for Alert Level Systems

#### Ensuring the most appropriate operational data are utilised for ALS decision-making.

Every jurisdiction reported significant challenges accessing the data needed for their ALS. Challenges shifted over the course of the pandemic, with the early phases affected by limited diagnostic capacity and later pandemic phases characterised by undetected and unreported cases and data systems becoming ‘overwhelmed’ (Interviewee 7). Health system capacity was increasingly viewed as the most critical indicator of epidemic severity, and several jurisdictions attempted to estimate incidence and set ALS levels by relying on proxy data such as COVID-19 death rates, ICU bed capacity, or modelling outputs to assess risk.

*“The consequences of that is that we were in some ways… making a lot of assumptions or best guesses… about various issues during the pandemic, and our decisions about moving to a higher or lower alert level, even remaining on an alert level, were dictated to by the quality of evidence.”* (Interviewee 1).

Alongside these challenges, several respondents also described the difficulty of balancing epidemiologic, economic, and social trade-offs when setting levels. While South Africa’s ALS took account of economic as well as epidemiological concerns, [8] data availability is also likely to have had an impact on the approach to structuring ALS decision-making, as the lack of social or economic data relative to available epidemiologic data hindered precautionary action through assessment of both sets of risks. [12]

#### Setting clear objectives for ALS.

Some respondents felt that a lack of clear objectives for their ALS contributed to difficulties determining the right data to use to set levels. The emergent pace of implementation and limited time to plan strategically contributed to this lack of clarity, and therefore appropriate decision-making about relevant data streams.

*“Be absolutely clear on what your intended outcome is… what are you trying to do with establishing the alert level [system]… And be clear on how the data actually relate to that outcome.”* (Interviewee 12)

Shifting the objective of the ALS without appropriately redesigning the system led to challenges with Singapore’s DORSCON system, which had been conceived as an internal coordination tool. [6,13] DORSCON set levels based on a risk assessment that used pre-determined criteria that prioritised risk aversion, and this led to problems when DORSCON was repurposed for use as a public communication tool.

*“So when we did risk assessment[s], while we looked at the nature of the disease, the disease transmissibility, the severity… these are not really salient to the public actually – and then there were also other risk factors we needed to account for like the availability of, for example, medical countermeasures, the ability of whether our healthcare system could cope, the effectiveness of our public health measures and… DORSCON didn't take [that] into consideration which was why it [was] kept at orange for over two years.”* (Interviewee 6)

This meant that until substantial population immunity from infections or vaccination had been attained, the underlying risk assessment didn’t change. There was also a reluctance to escalate to the top level (red) for fear of causing public alarm. Taken together, these features led DORSCON to be ‘stuck’ at orange for two years of the pandemic. Beyond challenges related to identifying appropriate data and setting objectives, respondents also reflected on how PHSMs linked to ALS were determined and implemented in practice.

### Setting PHSMs associated with ALS

#### Feasibility and practicality of PHSMs.

Several respondents discussed limitations associated with asking community members to adhere to measures for which there were structural barriers to implementation and noted the consequences for effective epidemic control. In Brazil and South Africa, decision-makers had to contend with local social structures and economic constraints that limited the extent to which PHSMs could feasibly be implemented.

*“So we had thought ‘we have a significant proportion of the population living in what we describe as informal settlements.’ So social distancing and being able to have ventilation is not a feasible option for them. So you can provide them with all that advice but it’s really impossible.”* (Interviewee 1)

In higher-income settings, social and economic considerations were less limiting to the structure and reach of ALS-associated measures, since in some circumstances the provision of resources to facilitate adherence to measures was more available to policymakers.

In South Africa, the structural limitations on peoples’ ability to adhere to more restrictive measures meant that there was a risk that the ALS lost credibility when it recommended measures that community members fundamentally couldn’t implement.

*“And some of the measures that we advocated were not relevant and not applicable… so if you're living in an environment where you have six people in a tin shack, social measures are not relevant.”* (Interviewee 2)

Conversely, in the Philippines, the provision of structural support to those in lockdown or quarantine was perceived by one respondent to have strengthened the social contract, built trust in public authorities, and improved willingness to adhere.

*“Because of the burden of the increasing number of cases of COVID at the height of pandemic, they were forced to be at home and not go out… And then they saw that the government is also responding and providing what they need, the basic needs… [and] so we get… the trust of the public.”* (Interviewee 14)

One UK respondent noted the negative epidemiological outcomes associated with socio-economic disparities in the country and differences in the support systems available to community members.

*“One area that came up throughout quite a lot of work was … what limited support [region x] sometimes had, for example. So [region x] might always be running quite hot because it doesn' have so much support around it”* (Interviewee 5).

The provision of enabling support to adhere to PHSMs was therefore described as having instrumental value for disease control efforts, as well as an important intrinsic value for trust-building and community engagement. [14,15]

#### Evidence-based PHSMs for improved response credibility.

Several interviewees reported that some mandated measures were not based in public health science and were therefore perceived by the public to be ‘irrational,’ damaging the credibility of the overall epidemic response. In New Zealand, one example arose around a lack of differentiation between measures for indoor and outdoor events.

*“As the pandemic had worn on, people started to question the logic of some of the measures. And, you know, an obvious one was [that] outdoor events were subject to the same restrictions as indoor ones, because of ventilation and other things. And so, you know, [people] started to sort of question the logic of that.”* (Interviewee 8)

An interviewee from South Africa noted that some measures had no basis in evidence-based outbreak responses.

*“There was a regulation that said we couldn't wear open-toed shoes. Now where in the world is there any science that says, open toe shoe would expose you to… even with Ebola unless you're working with the patient who's bleeding profusely then your open toe shoes would be a risk!”* (Interviewee 2)

These ‘irrational’ measures were implemented for several reasons in different geographies, with the early days of the pandemic often seeing them driven by uncertainty about COVID-19 transmission mechanisms and therefore a tendency towards risk aversion among decision-makers. In addition to questions about the feasibility and evidentiary basis of PHSMs, respondents emphasised that the legitimacy and effectiveness of ALS depended heavily on community engagement and trust.

### Community engagement, trust, and credibility

#### Decision-making transparency and public trust.

Perceptions of ALS decision-making transparency affected trust and credibility; the importance of transparent decision-making for trust-building has been demonstrated previously. [16,17] One South African respondent noted how a lack of transparency negatively affected public perceptions.

*“[There was a] lot of unhappiness with what people initially saw as the opacity of the decision-making process between the ministerial committee and cabinet decisions, because the advice from the ministerial committee initially was not made public.”* (Interviewee 4)

Moreover, it may have been important to consider the reputation of those engaged in decision-making, and the extent to which those actors might be perceived to be motivated towards transparency. In the UK, the decision to develop an ALS was influenced by the perception that similar tools had successfully managed terror threats, and as a result, intelligence community staff were brought into a novel agency called the Joint Biosecurity Centre (JBC) to develop an ALS for COVID-19. This participation of intelligence staff in ALS development may have affected public trust. [9]

*“The people who were initially brought in to set up the JBC - there were quite a few people who came from the intelligence community. Because there were two figureheads who the media were quite interested in, it started to take a little bit of a negative slump”* (Interviewee 17).

Many jurisdictions reported expending substantial effort on data transparency to circumnavigate accusations of opaque decision-making and to enhance trust. The US Center for Disease Control and Prevention’s (CDC) COVID Community Levels avoided setting levels based on predictive modelling since the complexity of the model ‘back end’ would obfuscate decision-making to the public at a time when trust-building was paramount.

*“[The public] want to know, like, where are we going? And at that point, with the amount of distress in the public, I don't think… that was the more quantitatively complex models, and we didn’t have the public trust really to be able to promulgate those.”* (Interviewee 10)

Several respondents cited data and methodological transparency as one of the bigger successes of their ALS.

*“And it probably has been a paradigm shift for public health response to major incidents, as in public expectations [for] transparency has never been higher. [It] demonstrated what can be done. So, I think it’s hard to describe that as anything other than a big success. And one that was worth the resource invested.”* (Interviewee 16)

### Community engagement and communications expertise for ALS design and implementation

Many respondents expressed regret at not engaging specialised communications or community engagement staff earlier in the process of developing their ALS, or at not having ensured community representation within decision-making fora. In almost all jurisdictions, the pace of ALS implementation and overall epidemic response meant that the ALS functioned more as a unidirectional risk communication tool than one that facilitated meaningful community engagement.

Only South Africa reported having established formal mechanisms for community feedback such as routine focus group discussions and a hotline from which community feedback (or ‘social listening’) data were incorporated into decision-making. Further, none of the jurisdictions reported substantive efforts to understand community needs or preferences to guide the design of their ALS. One respondent from New Zealand reported that while behavioural science research had been initiated to examine community responses to the ALS, it was rendered less important as the trajectory of the pandemic progressed.

*“[T]hat ended up being overtaken by more urgent requirements to understand vaccine readiness, so by the time the alert levels had done their job, it was then all about the vaccine.”* (Interviewee 3)

Similarly, no jurisdiction reported having community representation within decision-making fora, although South Africa did include traditional and faith healers within its social and behavioural science ministerial advisory committee. One respondent from New Zealand felt the lack of representation compromised the quality of guidance and advice provided to the public.

*“Looking back on it, there were people who were missing from that. We could have increased that a lot more. I do regret that we didn' have enough voices at that level from our Maori representatives of our Maori population.”* (Interviewee 9)

### Trade-offs in public communication of ALS

Many respondents discussed the importance of simple and interpretable ALS ‘front-end’ graphics that clearly conveyed response messages. Several jurisdictions reported grappling with how best to balance ensuring the public-facing or graphic elements of their ALS were simple enough to convey critical messages while also appropriately capturing epidemiological or geographic nuances and variations. In New Zealand, this meant a brief foray into having an alert level of 2.5 before it was abandoned.

*“Maybe we were just trying to please too many people… Our level settings were simple, we liked them because they were simple, it was a good communication tool, but it really wasn’t nuanced enough to deal with the complexity… [M]aybe we should just have accepted you’re either a three or a two, you can’t be anywhere in between… The main feedback was public confusion, business as being confused, a sense of outrage from some people.”* (Interviewee 9)

The need to have a simple, jurisdiction-wide tool also may have contributed to perceptions of inequity in some jurisdictions. In New Zealand and the United Kingdom, respondents discussed the geographic inequity associated with having a nationally focused system that didn’t account for differences in local transmission rates, and therefore recommended the same levels of stringency in measures in areas with very different epidemiological risk.

*““The challenge was, it was UK wide, so you could have alert level four in the far reaches of Wales where people have got three neighbours, and [also] in [region x] where [prevalence is much greater]. So like, it was a broad brush, but we had to do a lot more tailored stuff in local areas to help people understand what’s going on”* (Interviewee 17)

As noted by this respondent, the lack of granularity in the front-end system meant additional communication efforts were required, particularly in the worst-affected areas, to ensure communities understood their local situation. Both countries later transitioned to more of a localised approach, which also led to accusations of inequity when some geographic areas remained in lockdown, while others were subject to less restrictive measures. This contributed to substantial operational challenges, as noted by a respondent from New Zealand.

*“It also meant that some of the other factors that were present earlier on, like the sense of the whole community’s in this together… was eroded to some extent because we suddenly had this, ‘this is what’s happening in Auckland’ whereas the rest of the country is relatively free… plus just the sheer challenge of how do you manage your largest city in the country having restrictions around movement when there’s so much movement in and out of that city normally and just the ability to… hold that wall and I think that’s where it got increasingly complex.”* (Interviewee 9)

In South Africa, which has a quasi-federal political system, population mobility was also seen to militate against the feasibility of a geographically differentiated approach to level-setting. Attempts were therefore made to engage local leaders regarding those nationally-defined levels, but these were undermined by political factors, and perhaps because that engagement was focused primarily on informing local leaders about level changes, rather than engaging them in the level-setting process. Similar issues arose even in California, which took a granular (county-level) approach to its ALS.

*“Like anyone who lives on any border of a jurisdiction and even within our counties if there’s one intervention across the street from the other because they're on that border it’s hard… We all tried to do our best to figure out what thresholds to use, but there was some arbitrariness to that too, so it was how do you decide exactly which one to factor at these different alert levels.”* (Interviewee 7)

Alongside these external communication challenges, respondents also discussed internal governance structures that shaped how alert levels and associated measures were ultimately determined.

### Decision-making processes

#### Separation of technical teams and political decision-makers.

All jurisdictions reported having clear separation between the technical team analysing data, conducting risk assessments, and making recommendations (typically seated within Ministries of Health or associated agencies), and policy or political decision-makers who had the final say on any level-change. This was often because decision-makers had to consider factors beyond public health, which sometimes meant a deviation from technical team recommendations.

*“So the risk assessment was what we did behind the scenes, the public health risk assessment. And then we determined from the public health risk assessment, we put a recommendation up as to which alert level we thought the government should adopt. They didn’t always take our advice. Sometimes they went more and sometimes they went less.”* (Interviewee 8)

The separation of technical teams and political decision-makers helped to protect decisions (and scientists themselves) from ‘interference’ (Interviewee 17) and the political pressures that arose during very polarised debates about pandemic decision-making.

*“At the time, I had a permanent concern about how we position ourselves. And in the end, I think [we needed to] focus as much as possible on a scientific question, to look at the data. We are following what was determined here. We had a better way to defend ourselves against these polarized parties.”* (Interviewee 11)

By leveraging the perceived neutrality of scientists, difficult decisions were more easily justified to the public, which was further strengthened by transparent data-based decisions that could be publicly scrutinised.

*“So it was a government decision then around the alert level that… you could clearly see what the rationale and the articulation of the advice was by them.”* (Interviewee 9)

However, some key informants described challenges with this approach caused by an insufficient mix of technical disciplines within their technical team, which occasionally led to out-of-touch or disciplinarily siloed recommendations.

*“A lot of our scientific staff… could be impractical. And, you know, wanted to do it in the most epidemiologically rigorous way, which… can become almost essentially unusable to most members of the general public.”* (Interviewee 15)

### Manual adjudication of level-setting and PHSM assignment

A manual approach to level-setting provided the flexibility that many respondents valued, particularly given the dynamic nature of COVID-19, where fixed ‘thresholds’ were difficult to justify. Some jurisdictions adopted a more automated method, relying on predetermined thresholds, while others favoured a manual, yet still data-driven, approach. Several key informants viewed the pandemic’s unpredictability as a limitation for the use of automated methods, expressing concerns that such an approach might compromise political ownership of decision-making or reduce the involvement of relevant stakeholders in the process.

*“Everyone wanted thresholds, thresholds. Love thresholds, nice and definitive. And you point to a threshold and say, we’re above or below a threshold, therefore, we should do this. However, you then very quickly realize, especially in the case of something like COVID, where everything is evolving and novel in many ways. There’s no very solid ground to build a threshold on. And therefore, there’s very little way to keep it accurately live and updated.”* (Interviewee 16)

In California, which had a more automated approach to level-setting, there was a manual adjudication process embedded within the system to enable scrutiny of automated alert level assignments, account for data outliers (e.g., to review levels where positivity rates in less populated rural areas would exaggerate the risk), and to facilitate stakeholder engagement and buy-in.

### Stakeholder buy-in and ‘whole of government’ decision-making

Stakeholder buy-in was facilitated in most jurisdictions by taking a ‘whole-of-government’ approach to the review of technical recommendations and build consensus around decision-making. Some jurisdictions felt this helped ensure cross-government alignment and therefore garnered public buy-in around changes to levels or measures.

*“[Ministers] wouldn’t necessarily bring anything particularly for medical or data awareness… [but] without them, you wouldn’t have the political buy-in across the system. I think you would have had more issues of the public getting a perception of the government not being fully aligned on a lot of things. And so if you'e having ministers, the CMOs [Chief Medical Officers] and the analytical focus behind it, was it a nice… tri-partite way of working.”* (Interviewee 16)

While most key informants said this approach worked well in their jurisdictions, and was politically necessary, it did create substantial additional workload for overstretched teams. While this may have reduced the speed at which decisions could be made, it was considered by most interviewees to have ultimately led to greater efficiency.

*“As you might imagine, that… brings a lot more people with it, takes a lot more layers, takes a lot more time, but then everyone actually believes in it.”* (Interviewee 16)

This was even true for ALS where a more automated approach to level setting was taken, such as the US CDC’s.

*“There’s multiple other groups ranging from hospital associations, organizations of compromised people, sorts of physicians’ organizations, teachers’ associations. I had a very long list of groups that really have, it’s not just a courtesy, they really need to understand if a change like this is going to be made and will quite rightly be pretty vocal if they’re not well briefed. Then you have the media and the public at large. It was really just challenging – lots of work.”* (Interviewee 10)

## Discussion

Our results show that ALS were perceived to be useful public communication platforms during the COVID-19 pandemic. Respondents described how ALS, when accompanied by PHSMs, were believed to improve risk awareness and adherence. These findings reflect stakeholder perceptions of communication and implementation effectiveness rather than formal evaluations of epidemiological impact. Most respondents felt that linking an ALS to appropriate PHSMs helped their jurisdictions mitigate the impacts of the pandemic, particularly in a context where assessments of risk were very dynamic.

Respondents described how trust in the overall response was affected by the extent to which ALS decision-making was perceived to be transparent, consistent with findings in other settings. [16,17] A benefit of ALS implementation noted by multiple respondents was that it pushed jurisdictions to clearly define their decision-making methodologies, resulting in greater transparency. Involving trusted leadership figures and leveraging the perceived neutrality of science or scientists [18] in both decision-making and public communication were noted here and elsewhere as likely factors in ALS implementation success. [19] In addition, a ‘whole of government’ approach to decision-making, with community representation and engagement a feature of the decision-making process, can help build broad consensus and provide the diverse inputs required to make informed decisions given the whole of society impact of major health emergencies and the consequences of countermeasures.

In this study, respondents described several lessons learnt, which we synthesised into a set of practice-oriented recommendations for future ALS design (Table 1). While many recommendations are directly grounded in key informant accounts, others reflect cross-case synthesis and alignment with established literature on risk communication and early warning systems. Only one jurisdiction had utilised an ALS for public health emergencies developed before the emergence of COVID-19; other jurisdictions described how over-stretched teams had to rapidly develop and implement their ALS. Lack of clear ALS objectives, challenges accessing data, lack of community engagement and feedback mechanisms, use of untested communication products, recommending PHSMs that were not evidence-based, and limited monitoring and evaluation of ALS implementation and PHSM adherence are all examples of challenges that could have been mitigated had ALS governance structures and systems been developed in advance of an outbreak. Much has been written about the ‘tyranny of the urgent,’ and the ways in which focusing on technical (rather than social) solutions can exacerbate existing inequalities while weakening the overall public health response. [20–22] The COVID-19 pandemic was inevitably an exemplar of this phenomenon, with most jurisdictions prioritising ‘getting something up and running’ over consultation and engagement.

**Table 1:** Recommendations for ALS design and implementation for use in future epidemics.

**Setting objectives and identifying data for ALS**	• Involve a broad group of stakeholders in setting clear objectives for any ALS at the outset including the target audience (communities or individuals). • Develop decision-making processes, lists of possible indicators, communication strategies, and sample communication materials before an outbreak, so they can quickly be customized and operationalized when an outbreak emerges. • Supplement use of traditional epidemiological indicators (e.g., incidence rates) with relevant health system capacity indicators to enable better monitoring of transmission dynamics, disease severity, and the need for more stringent PHSMs to prevent the health system from being overwhelmed. • Where possible, plan to collect social and economic indicators for use in level-setting processes in order to balance epidemiologic, economic, and social impacts of measures. • Maintain flexibility by routinely reviewing and incorporating additional indicators into decision-making, including vaccines or other medical countermeasures as they become available.
**Setting PHSMs associated with ALS**	• Utilise clear and easily interpretable indicators and a transparent decision-making process to set PHSMs in order to build and sustain public trust. • Set ALS and PHSMs through multi-sectoral coalitions or groups that include diverse technical specialists, political stakeholders, and community representatives. • Ensure that PHSMs are evidence-based by having public health professionals routinely review measures before and during their implementation. • Ensure that PHSMs align with social/economic context to ensure they are feasible. • When possible, PHSMs should be supported by policies, interventions, and specific community engagement strategies to mitigate adverse social and/or economic impacts and improve adherence.
**Community engagement, trust, and credibility**	• Engage community organizations that represent communities uniquely affected by the outbreak when developing and modifying PHSMs (e.g., advocacy groups for marginalised communities, teacher’s organisations, hospital associations, healthcare worker organisations, business representatives). • Set levels using clear and easily interpretable indicators and a transparent decision-making process open to public review in order to build and sustain public trust. • Engage specialized communications and community engagement staff from the conception stage of the ALS. • Make the public’s interface with the ALS as clear and simple as possible and avoid creating too many levels • ALS and associated PHSMs should be determined with input from community representatives and adjusted through routine community feedback data collection. • Involve trusted leadership figures in both decision-making and public communication. • Test ALS communication products with the public prior to and throughout implementation (e.g., A/B testing). • Set and communicate PHSMs at the smallest geographic unit for which there are adequate data for informed decision-making and where mobility between geographic units is relatively limited, in order to maximize the relevance of alert levels and PHSMs and to maintain credibility. • ALS and PHSMs should be developed through multi-sectoral coalitions or groups that include diverse technical specialists in order to build consensus and unity around decisions.
**Decision-making processes**	• Separation between technical teams generating evidence-based recommendations and political decision-makers can help the technical team to provide impartial recommendations and allows the political team to rely on the credibility of technical experts to implement difficult decisions. • ALS decision-making processes and communications channels should be determined and communicated to the public in advance of an emergency. • Avoid using fixed thresholds for novel pathogens as data availability and scientific knowledge can rapidly change. When using fixed thresholds to set levels, incorporate a manual adjudication process.

A key lesson from the implementation of ALS with PHSMs was the importance of considering social and economic consequences alongside epidemiological efficacy. [9] The challenges and complexities associated with incorporating social and economic data into decision-making was found to predicate against ALS automation. Public engagement with the ALS and associated PHSMs relied, in most jurisdictions, upon high risk perception that waned over the course of the pandemic and in response to decreasing trust in public authorities, [23] not least because of the duration of the pandemic and the reliance upon mandates that gradually eroded the existing social contract. Identifying appropriate mechanisms to maintain community engagement is likely to be critical for the success of future ALS implementation. Other warning science areas emphasise the importance of viewing ALS as continuous integrated components of participatory systems, rather than as unidirectional, isolated risk communication tools. [24–26]

Ensuring that any ALS is fit-for-purpose locally also requires balancing the need to keep communication products simple while capturing epidemiological or geographic variations. Warnings have been shown to be most effective when they are hyperlocal, as this can help community members take actions appropriate for their locale [27,28] as well as avoiding accusations of inequity (when for example, communities experiencing limited local transmission are placed under restrictive measures because of heightened transmission elsewhere in a country or jurisdiction). However, implementing ALS at a sub-national level was found to be challenging due to incomplete or imprecise data, the inherent mobility of many populations, and differing priorities and pressures faced by nationally and locally elected political representatives.

Several respondents across varied jurisdictions noted the importance of ensuring that communities have agency to be able to implement the actions recommended by policymakers. Infeasible measures can cause the response to quickly lose credibility and fail to meet its targets. [29] Monitoring the social and economic effects of PHSMs among general and vulnerable populations, and then providing economic support or other interventions to mitigate negative impacts of PHSM implementation, can support adherence. In addition, recommended or mandated measures should be routinely reviewed by public health professionals to ensure they are evidence-based to facilitate credibility in any outbreak response or public authority overseeing a response and help maintain trust. [30]

### Study limitations

None of the jurisdictions consulted for this study conducted formal evaluations or assessments of their ALS implementation. However, many of the perceived challenges and successes associated with ALS implementation were shared by key informants representing diverse jurisdictions included in this study. These findings should therefore be interpreted as experiential insights rather than generalisable evidence of epidemiological effectiveness.

Given the purposive approach to identification of both case studies and key informants, the sample is not statistically representative. Instead, in the absence of other empirical data on ALS implementation during the COVID-19 pandemic, this research aimed to document the perceived lessons learnt around ALS for COVID-19 by expert key informants in order to ensure that information on the features that inform ALS implementation are available to the public health community and able to inform decision-making about their use in future public health emergencies. Recruitment of respondents via existing networks may have introduced biases towards participants with a particular view on the effectiveness of ALS in public health emergencies.

## Conclusions

Our findings, based on key informant perceptions rather than formal outcome evaluations, suggest that ALS have the potential to serve as useful public communication tools in future epidemics. The ability of ALS to streamline and simplify public communication around risk and the need for PHSMs may support future public health responses. However, the sustained success of any ALS relies upon its ability to build and sustain public trust. Developing the structure for ALS implementation before an epidemic, so that these structures only require adaption to the context of a given outbreak, will improve the ability of public health teams to implement a high-quality ALS.

ALS activities that jurisdictions can enact to prepare for the next epidemic include 1) aligning on ALS objectives; 2) identifying and securing access to key data and indicators; 3) defining roles, responsibilities, and processes for decision-making that will produce decisions at the right speed and cadence; 4) developing and testing sample communication materials and platforms; and 5) socialising the ALS with the public.

## Supporting information

S1 Appendix Respondent Designations (at the time of implementation of COVID-19 Alert Level Systems). (PDF)

S2 Appendix Examples of COVID-19 Alert Level Systems. (PDF)
