# Supplementary material for: COVID-19 alert level systems—Lessons learnt for future public health emergencies: A qualitative study
Source: PLoS One. 2026 Jun 18;21(6):e0351209. doi: 10.1371/journal.pone.0351209 (PMC13278578; doi:10.1371/journal.pone.0351209)
Supplement: S1 Appendix — (PDF) [file pone.0351209.s001.pdf]

SUPPLEMENTAL MATERIAL

COVID-19 Alert Level Systems – Lessons Learnt for Future Public Health Emergencies

ANNEX 1: Respondent Designations (at the time of implementation of COVID-19 Alert Level Systems)

Deputy Director-General for Health

Deputy Director

Chief Science Advisor

Deputy Director-General for Health

Deputy Director of Public Health

Deputy Director

Head Analyst

Head of Communications

Assistant Director

State Epidemiologist; Deputy Director

Head of Section

Departmental Director

Previously IM for COVID

Former Director of Public Health

Deputy Director

Principal Deputy Director

Senior Advisor

Director
