# Supplementary material for: COVID-19 alert level systems—Lessons learnt for future public health emergencies: A qualitative study
Source: PLoS One. 2026 Jun 18;21(6):e0351209. doi: 10.1371/journal.pone.0351209 (PMC13278578; doi:10.1371/journal.pone.0351209)
Supplement: S2 Appendix — (PDF) [file pone.0351209.s002.pdf]

SUPPLEMENTAL MATERIAL

COVID-19 Alert Level Systems – Lessons Learnt for Future Public Health Emergencies

ANNEX 2: Examples of COVID-19 Alert Level Systems

Table of Contents

Jurisdiction	Page number
New Zealand	2
Phillipines	7
Rio Grande do Sul, Brazil	36
Singapore	212
South Africa	246
United Kingdom	255
United States	265

COVID-19

Elimination Strategy for Aotearoa New Zealand

Objective: To prevent cases of COVID-19 | KOWHEORI-19 entering New Zealand while being ready to quickly eliminate any chains of transmission in the community

Approach: We learn and iterate our evidence and risk-based approach to the best set of public health measures balancing health, economic and social outcomes for New Zealand, while working closely with New Zealanders to grow and sustain social license for the measures taken

HEALTH RESPONSE PILLARS

STRATEGIC ACTION AREAS

KEEP IT OUT

1 Pre- & Border Settings, Managed Isolation & Quarantine

Strong but proportionate border settings flex and adapt as health security settings shift

- Clearly defined health criteria | testing at NZ border
- Mutually agreed health and transport protocols between countries for travel & transit
- MIQ settings and wrap-around services
- Negotiated quarantine (-free) travel arrangements
- Travel restrictions align to immigration settings
- Protocols for high-risk staff at border (air crew, maritime crew, airside workers, port workers, MIQ workers)

PREPARE FOR IT

2a Detection & Surveillance

Best-evidence protocols ensure cases are detected and transmission is controlled

- Testing protocols and targeting
- Disease surveillance, e.g. Genome sequencing, Influenza-like illness, Sentinel testing (community sampling)
- Advice via Healthline and other health providers

2b Public Health Measures

Base-level public health behaviours | Alert Level 1

- Lowest Alert Level setting
- Personal protection and hygiene, e.g. frequent hand washing
- Systems to support contact tracing, e.g. technology

STAMP IT OUT

3a Contact Tracing & Case Management

Targeted and timely activities minimise impacts of new outbreaks across the community and health system

- Testing protocols and targeting
- Technology-enabled tracing
- Quarantine & self-isolation protocols
- Escalation protocols
- Community leadership to facilitate tracing

3b Stronger Public Health Measures

Public adherence to tailored health interventions is high due to trust and confidence in our health settings | Alert Levels 2-4

- Staying at home | travel bans
- Limits on social gatherings
- Targeted and timely behavioural intervention campaigns for different population groups
- Community leaders champion public health messages

FOUNDATIONAL PILLAR

MANAGE THE IMPACT

4 Health System Readiness & Resilience

Proactive planning ensures a proportionate response to manage impacts of COVID-19 and maintain other services. Implementation of the COVID-19 vaccination programme in 2021.

- Health system is adequately prepared e.g. critical care resources and workforce capacity (including surge capacity)
- Vaccine(s) | Therapeutics – Vaccines
- Best Health Evidence Guidelines
- The Ministry / DHBs work with the sector and communities in the planning, coordination and delivery of our response

New Zealand COVID-19 **Alert Levels**

Unite against COVID-19

- **The Alert Levels are determined by the Government and specify the public health and social measures to be taken in the fight against COVID-19.** Further guidance is available on the **Covid19.govt.nz** website.
- The measures may be updated based on new scientific knowledge about COVID-19, information about the effectiveness of control measures in New Zealand and overseas, or the application of Alert Levels at different times (e.g. the application may be different depending on if New Zealand is moving down or up Alert Levels).
- Different parts of the country may be at different Alert Levels. We can move up and down Alert Levels.
- Essential services including supermarkets, health services, emergency services, utilities and goods transport will continue to operate at any level. Employers in those sectors must continue to meet health and safety obligations.
- Restrictions at the different Alert Levels are cumulative (e.g. at Alert Level 4, all restrictions at Alert Levels 1, 2 and 3 apply).

Updated 14 December 2020

	ALERT LEVEL 1	ALERT LEVEL 2	ALERT LEVEL 3	ALERT LEVEL 4
**OUTCOME**	Keep out global pandemic. Population prepared for increase in alert levels if necessary.	Physical distancing and restrictions on gatherings to address sporadic cases or a cluster in New Zealand.	Further restrictions on activities, including at workplaces and socially, to address a high risk of transmission within New Zealand.	Strong restrictions to limit all people movement and contact to contain community transmission and outbreaks.
**SUMMARY**	Be prepared, and be vigilant.<br>Border measures are in place.<br>Public health measures in place, but no physical distancing is needed.	Businesses open, but physical distancing requirements apply.<br>Gatherings limited.	Stay at home, other than for essential personal movement, and going to work/school.<br>Stay in extended bubble, which includes close family or caregivers.	Stay at home, other than for essential personal movement and doing essential work.<br>Stay in immediate household bubble.
**Public health measures**	Public health measures are guidance for everyone but are not a legal requirement.<br>No physical distancing requirements.	People should keep 2 metres from people they don't know in public and retail stores. Keep 1 metre in other environments like workplaces, gyms, libraries and cinemas where practicable.<br>Groups of friends and whānau should be limited to 100 people when socialising.	People required to keep 2 metres apart outside home where possible (apart from people within their extended bubble). This requirement does not apply to emergency and frontline public services (e.g. healthcare).<br>In a controlled environment such as a workplace, 1 metre distancing is required.	People should keep 2 metres apart at all times outside home, including at workplaces. This requirement does not apply to emergency and frontline public services (e.g. healthcare).

General public health advice:

- Regularly disinfect surfaces; wash and dry hands, cough and sneeze into elbow, don't touch your face; if you have cold or flu symptoms stay at home and ring Healthline or your GP.

Contact tracing:

- Ongoing contact tracing for all confirmed and probable new cases of COVID-19, with appropriate isolation measures put in place.
- QR codes issued by the NZ Government must be displayed in workplaces and on public transport to enable use of the NZ COVID Tracer App for contact tracing.

Testing:

- Testing of all potential cases of COVID-19 for people who meet the case definition (i.e. are displaying relevant symptoms). Tests will take place at dedicated Community-Based Assessment Centres or designated practices.
- Random testing within communities (including for people who are asymptomatic) may be carried out locally to inform understanding on the spread of the virus in certain areas.

Isolation and quarantine:

- Stringent self-isolation of those who display relevant symptoms of COVID-19, test positive for COVID-19, have been in close contact with someone who tests positive for COVID-19, including quarantine/managed isolation for those who have been overseas in the last 14 days. Quarantine facilities mandated for those who do not have sufficient capacity to self-isolate effectively.

Border:

- Robust border measures in place which safeguard against the risk of COVID-19 being transmitted into New Zealand. Currently, managed isolation or quarantine on arrival for 14 days before onward domestic travel.

<table>
<tr><th></th><th>ALERT LEVEL 1</th><th>ALERT LEVEL 2</th><th>ALERT LEVEL 3</th><th>ALERT LEVEL 4</th></tr>
<tr><td>Personal movement</td>
<td>No restrictions on personal movement.

Sports and recreational activities allowed.

People are encouraged to record where they have been and who they have been by using the NZ COVID Tracer App.</td>
<td>Leave home, but in a safe way.

Participating in sports and recreational activities is allowed, subject to conditions on gatherings, record keeping, hygiene requirements and – where practical – physical distancing.

People at higher-risk of severe illness from COVID-19 (e.g. older people and those with underlying medical conditions, especially if not well-controlled) may work and study, if they agree with their employer or education provider that they can do so safely.</td>
<td>People instructed to stay at home, other than for essential personal movement:
• Accessing local services and businesses
• Going to work or school (only for those who have to)
• Low risk recreation in local area
• Shared and extended bubble arrangements
• Emergencies and giving effect to court orders
• Travelling to permitted gatherings
• Limited customary purposes
• Relocating a home or business
• Medical reasons
• Those who have an exemption to travel because of compassionate reasons
• Foreign nationals leaving New Zealand
• New Zealanders resident in the Realm returning home, and
• People arriving in New Zealand from overseas and returning home after 14 days' isolation/quarantine at port of arrival (except air and marine crew).

People must stay within their immediate household bubble, but can extend this to connect with close family/whānau, or bring in caregivers, or support isolated people. This extended bubble should remain exclusive. Anyone who feels unwell must immediately self-isolate from others in their extended bubble.

People at higher-risk of severe illness from COVID-19 (e.g. those with underlying medical conditions, especially if not well-controlled, and the elderly) are encouraged to take additional precautions when leaving home. They may work, if they agree with their employer that they can do so safely.</td>
<td>People instructed to stay at home, other than for essential personal movement as defined in Health Act Order of 3 April 2020.

People must stay within their immediate household bubble. There may be extended bubbles where there are shared care and custody arrangements. Anyone who feels unwell must immediately self-isolate from others in their bubble.

Sports and recreational activities allowed if within scope of essential personal movement as defined in Health Act Order of 3 April 2020.

People at higher-risk of severe illness from COVID-19 (e.g. those with underlying medical conditions, especially if not well-controlled, and the elderly) are encouraged to take additional precautions when leaving home. They may work, if they agree with their employer that they can do so safely.</td></tr>
<tr><td rowspan="4">Travel and transport</td>
<td colspan="3">No restrictions on freight. All freight can be distributed and received.

All freight can enter and leave the country.</td>
<td>All freight can be distributed and received, with essential freight prioritised. This includes de-vanning, delivery to and receipt by businesses (including those businesses not currently permitted to trade for receipt only) and customers.

All freight can enter and leave the country.</td></tr>
<tr><td>Face coverings must be worn on domestic flights.</td>
<td colspan="3">Face coverings required on public transport and domestic flights (but not inter-island ferries) – school buses and children under 12 are exempt along with passengers in taxis or ride share services and people with disabilities or mental health conditions. Face coverings can be anything that covers your face, or a mask.</td></tr>
<tr><td colspan="2"></td>
<td colspan="2">Passengers and workers in transport stations and on public transport services must comply as far as reasonably practicable with the 1-metre physical distancing rule. On air transport services and small passenger service vehicles passengers must maintain physical distancing as far as reasonably practicable.</td></tr>
<tr><td>No restrictions on domestic travel.

Avoid mass transport if sick, awaiting a result from a COVID-19 test, or required/recommended to self-isolate.

Border restrictions remain in place.</td>
<td>You can travel, but do it in a safe way.

Do not use mass transport if required to self-isolate/quarantine, experiencing symptoms of COVID-19, awaiting a result from a COVID-19 test, suspected/probable/confirmed to have COVID-19, or if subject to an individual notice issued under section 70(1)(f) of the Health Act.

Appropriate physical distancing and other risk mitigating measures in place on public transport and aircraft as agreed by relevant agencies.</td>
<td>Travel is allowed for the following essential personal movement in your local area:
• Accessing local services and businesses
• Going to work and school
• Low risk recreation in local area
• Extended bubble arrangements, and
• Travelling to permitted gatherings.

Those travelling on public transport should avoid peak times unless they are going to work or school.</td>
<td>Personal travel (including the use of private cars or public transport) is only permitted within territorial authority, and for essential personal movement as defined in Health Act order of 3 April 2020.</td></tr>
</table>

	ALERT LEVEL 1	ALERT LEVEL 2	ALERT LEVEL 3	ALERT LEVEL 4
**Travel and transport (cont)**			**Travel between regions is allowed for the following essential personal movement:**<br>• Workers travelling to do essential work<br>• Going to work or school (only in neighbouring region)<br>• Shared bubble arrangements<br>• Relocating a home or business<br>• Those travelling for medical reasons<br>• Emergencies and giving effect to court orders<br>• Those who have an exemption to travel because of compassionate reasons<br>• Foreign nationals leaving New Zealand (except Cook Strait ferries)<br>• New Zealanders resident in the Realm returning home, and<br>• People arriving in New Zealand from overseas and returning home after 14 days' isolation/quarantine at port of arrival (except air and marine crew).<br>• Travelling out of Auckland to return to your primary residence.<br>All other travel is not allowed.	
**Gatherings**	No restrictions.<br>Organisers of gatherings are encouraged to keep records to enable contact tracing.	**All gatherings (such as weddings, birthdays, funerals and tangihanga) restricted to 100 people.**<br>Additional conditions on gatherings:<br>• Physical distancing and infection prevention and control requirements must be met.<br>• All gatherings must record attendees to ensure contact tracing may be conducted if necessary.<br>• All venues can open for the purposes of dining.<br>No participants allowed who have COVID-19 symptoms or who need to be in isolation/quarantine for any reason.	**Gatherings of up to 10 people at a time for wedding services, funerals and tangihanga.**<br>Wedding receptions or other celebrations are not allowed. Consumption of food/drink not permitted.<br>Workplaces, education facilities, public transport and supermarkets are not considered gatherings.<br>Additional conditions on gatherings:<br>• Physical distancing and infection prevention and control requirements must be met.<br>• All gatherings must record attendees to ensure contact tracing can be conducted if necessary.<br>• No participants allowed who have COVID-19 symptoms or who need to be in isolation/quarantine for any reason.	**All gatherings cancelled.**
**Public venues**	No restrictions.	**Public venues such as libraries and pools can open if they comply with public health measures and ensure 1 metre physical distancing and record keeping.**<br>Event facilities, including cinemas, stadiums, concert venues and casinos can have more than 100 people at a time, provided that there are no more than 100 in a defined space, and the groups do not mix.	**All public venues closed** (e.g. libraries, museums, cinemas, food courts, gyms, pools, amusement parks, playgrounds, farmers' markets).<br>**Public open spaces (e.g. parks) may be used**, but people need to maintain physical distancing outside their extended bubbles.	**All public venues closed** (e.g. libraries, museums, cinemas, food courts, gyms, pools, amusement parks, playgrounds, farmers' markets).<br>**Public open spaces (e.g. parks) may be used**, but people need to maintain physical distancing outside their bubbles.
**Health and disability care services**	No restrictions.	• Health and disability care services operate normally as far as possible.<br>• Hospitals will operate in line with the National Hospital Response Framework.<br>• Primary and community health providers will operate in line with the Community Response Framework.<br>• Physical distance and infection control guidelines followed.<br>• Remote consultations used wherever possible.	• Hospitals operate in line with the National Hospital Response Framework.<br>• Primary and community health providers will operate in line with the Community Response Framework.<br>• Residential facilities remain open with strict visitor policies. In home visiting required for priority populations.<br>• Pharmacies remain open.	• Hospitals operate in line with the National Hospital Response Framework.<br>• Primary and community health providers will operate in line with the Community Response Framework.<br>• Only urgent acute care conducted in person, maintaining public health guidelines. Routine care postponed.<br>• Residential facilities remain open with strict visitor policies. In home visiting required for priority populations.<br>• Pharmacies remain open.

	ALERT LEVEL 1	ALERT LEVEL 2	ALERT LEVEL 3	ALERT LEVEL 4
**Workplaces**	Businesses must operate safely **and** fulfil all their usual health and safety obligations. Alert Level 1 places no additional legal obligations on them:<br>• Businesses and services are encouraged to maintain records to enable contact tracing but this is not a legal requirement.	Businesses and workplaces must operate safely. This means:<br>• complying with general Alert Level 2 settings;<br>• meeting appropriate public health requirements for their workplace (e.g. having contact tracing systems and physical distancing); and<br>• fulfilling all other health and safety obligations.<br>All businesses are encouraged to use alternative ways of working if possible. Business premises can open for staff and customers provided they meet public health requirements. Services can also be provided on customers' premises (e.g. in homes).<br>Close contact services can operate if they meet public health measures including robust record keeping, good hygiene practices and minimised contact to the extent possible.<br>If a workplace cannot meet these measures it cannot open its physical premises.	People required to work from home unless that is not possible.<br>Workplaces can only open if:<br>• workers cannot work from home, **and**<br>• workplaces are operating safely, **and**<br>• customers are not allowed on premises, **and**<br>• businesses can trade without physical contact with customers (e.g. through phone/online orders, delivery, pick-up and drive-through).<br>Businesses cannot offer services that involve close personal contact, unless a supermarket, dairy, primary produce retailer (e.g. greengrocer, fish monger or butcher), pharmacy, petrol station or hardware store providing goods to trade customers, or it is an emergency or critical situation.<br>Supermarkets, dairies, primary produce retailers and petrol stations can have customers on premises. Retail is possible through delivery and non-contact collection of goods and prepared food at the door (including the doors of businesses located inside malls). No consumption of food/drink is allowed by customers on premises.<br>If businesses cannot operate safely, staff must not go to work and premises should remain closed.<br>"Operating safely" means:<br>• complying with Alert Level 3 settings in this table, **and**<br>• meeting appropriate public health requirements for their workplace, including for workers (e.g. putting up physical barriers), **and**<br>• fulfilling all other health and safety obligations.	People required to work from home unless that is not possible.<br>Workplaces can only open if:<br>• there is only one worker in the premises (or household bubble), **OR**<br>• workers cannot work from home, **and**<br>• they are operating safely, **and**<br>• they are essential services.<br>"Operating safely" means:<br>• complying with Alert Level 4 settings in this table, **and**<br>• meeting appropriate public health requirements for their workplace (e.g. putting up physical barriers), **and**<br>• fulfilling all other health and safety obligations.<br>This means if a business providing an essential service cannot operate safely, workers must not go to work and premises should remain closed.<br>Only supermarkets, dairies and petrol/service stations can open their retail premises to the public. Essential services must also comply with any specific restrictions on how they operate.
**Education**	Any educational facilities connected to a confirmed or probable case of COVID-19 must close temporarily, if advised by the public health unit, to support contact tracing and case and contact management.	**Tertiary education facilities, schools and early learning centres are open for all age groups.**<br>• Early learning centres and schools are all physically open including Years 11–13. Distance learning is available for those unable to attend school (e.g., where there are people self-isolating).<br>• Tertiary education facilities are open, and will maintain the core capability to deliver comprehensive distance learning to students.<br>Any educational facilities connected to a confirmed or probable case of COVID-19 must close temporarily, if advised by the public health unit, to support contact tracing and case and contact management.	**Early learning centres and schools are open for children in Years 1–10, with appropriate health measures in place.**<br>• Early learning centres will be open to provide childcare for people who are working. Children will not be able to attend playcentres and play groups. Home-based care, education and supervision of young children for more than one family in a home if public health control measures can be implemented. Children are encouraged to stay at home, if caregiving is available.<br>• Primary and intermediate schools are open. If there is a parent or caregiver available to look after children at home and school children have access to distance learning, children and young people are encouraged to continue distance learning at home.<br>• Secondary schools are open for young people in Years 9 and 10 who may not be able to stay home by themselves. All young people in Years 11–13 learn from home.<br>• Tertiary education facilities open for limited activities involving small groups (up to 10 people), and with distance learning provision for others.<br>Any educational facilities connected to a confirmed or probable case of COVID-19 must close temporarily, if advised by the public health unit, to support contact tracing and case and contact management.	**All educational facilities closed.**<br>• All schools engaged in some form of distance learning.<br>• Necessary tertiary student and some school hostel (where international students cannot return home and/or it is not safe for domestic students to return home) accommodation can remain open.

GUIDELINES ON THE NATIONWIDE IMPLEMENTATION OF ALERT LEVEL SYSTEM FOR COVID-19 RESPONSE
As of June 04, 2022

WHEREAS, the Coronavirus Disease 2019 (COVID-19), since having been declared as a Public Health Emergency of International Concern (PHEIC), has irreversibly affected millions of lives and families worldwide. Its unprecedented speed of transmission and infectivity has placed a huge burden on essential areas of governance, most importantly in vulnerable sectors such as the economy, education, and healthcare;

WHEREAS, Section 2 of Executive Order No. (E.O.) 112, (s. 2020) provides that provincial governors shall be authorized to impose, lift or extend the Enhanced Community Quarantine (ECQ) in component cities and municipalities upon the concurrence of the relevant regional counterpart body of the Inter-Agency Task Force for the Management of Emerging Infectious Diseases (IATF); and, that the mayors of cities and municipalities are likewise authorized to impose, lift or extend ECQ in barangays, upon the concurrence of the relevant regional counterpart body of the IATF; *provided*, that this is without prejudice to the authority of the IATF to directly impose, lift or extend ECQ in these areas should circumstances call for it;

WHEREAS, the IATF issued the Omnibus Guidelines on the Implementation of Community Quarantine in the Philippines (IATF Omnibus Guidelines), as amended, to harmonize and codify existing guidelines of the IATF and member-agencies pertaining to community quarantine, which shall be applied to all regions, provinces, cities, municipalities, and barangays placed under community quarantine;

WHEREAS, Guidelines for the Pilot Implementation of Alert Level System in the National Capital Region was adopted by the IATF and was published on 13 September 2021.

WHEREAS, the pilot area for implementation of the Alert Level System was expanded to include other provinces, highly urbanized cities, and independent component cities pursuant to IATF Resolution No. 144-D (s.2021) issued on 18 October 2021.

WHEREAS, Executive Order No. 151 (s.2021) issued on 11 November 2021, approved the nationwide rollout of the Alert Level System and adopted these Guidelines as the guidelines to be implemented and enforced in all areas under the Alert Level System.

WHEREAS, Section 6 of Executive Order No. 151 (s.2021) further provides that Executive Order No. 112 (s.2020) shall be deemed repealed once all areas of the country are placed under the Alert Level System.

NOW, THEREFORE, BE IT RESOLVED, as it hereby RESOLVED, that in consideration of the premises set forth herein, the IATF issues these Guidelines to enjoin and

proactively advocate the principles of 3C's (Closed, Crowded, and Close Contact) strategy against COVID-19 to curb the further spread of infection:

For purposes of these Guidelines, the following shall be defined as follows:

1. **Accommodation Establishments** - refers to establishments operating primarily for accommodation purposes including, but not limited to, hotels, resorts, apartment hotels, tourist inns, motels, pension houses, private homes used for homestay, ecolodges, serviced apartments, condotels, and bed and breakfast facilities.

2. **COVID-19** - refers to the Coronavirus Disease 2019 which is caused by the virus known as the severe acute respiratory syndrome coronavirus 2 (SARS-CoV-2).

3. **COVID-19 Alert Level System -** refers to the new Community Quarantine Classifications for dealing with COVID-19 covering entire cities, municipalities and/or regions; aimed to manage and minimize the risk of the disease through System Indicators, Triggers and Thresholds determined by the IATF to specify the public health and social measures to be taken in relation to the COVID-19 response, as may be updated based on new scientific knowledge, information about the effectiveness of control measures in the country and overseas, and its application.

   a. Alert Level 1 - refers to areas wherein case transmission is low and decreasing, total bed utilization rate, and intensive care unit utilization rate is low.

   b. Alert Level 2 - refers to areas wherein case transmission is low and decreasing, healthcare utilization is low, or case counts are low but increasing, or case counts are low and decreasing but total bed utilization rate and intensive care unit utilization rate is increasing.

   c. Alert Level 3 - refers to areas wherein case counts are high and/or increasing, with total bed utilization rate and intensive care unit utilization rate at increasing utilization.

   d. Alert Level 4 - refers to areas wherein case counts are high and/or increasing, with total bed utilization rate and intensive care unit utilization rate at high utilization.

   e. Alert Level 5 - refers to areas wherein case counts are alarming, with total bed utilization rate and intensive care unit utilization rate at critical utilization.

4. **Essential goods and services** - covers health and social services to secure the safety and well-being of persons, such as but not limited to, food, water, medicine, medical devices, public utilities, energy, and others as may be determined by the IATF.

5. **Granular Lockdown** - refers to a micro-level quarantine for areas identified as "critical zones" by the local government unit (LGU) which may be declared regardless of Alert Level.

6. **Health and emergency frontline services** - refers to services provided by public health workers [all employees of the DOH, DOH Hospitals, Hospitals of LGUs, and Provincial, City, and Rural Health Units, and Drug Abuse Treatment and Rehabilitation Centers including those managed by other government agencies (e.g. police and military hospitals/clinics, university medical facilities), uniformed medical personnel], private health workers, such as but not limited to medical professionals, hospital and health facility administrative and maintenance staff, and aides from private health facilities, as well as their service providers, health workers and volunteers of the Philippine Red Cross and the World Health Organization, and employees of Health Maintenance Organizations (HMOs), the Philippine Health Insurance Corporation (PHIC), health insurance providers, disaster risk reduction management officers, and public safety officers.

7. **Minimum public health standards (MPHS)** - refers to the national, local, and sector-specific guidelines on mitigation measures for its COVID-19 response across all settings by implementing non-pharmaceutical interventions (NPIs), consistent with the Department of Health (DOH) Administrative Order No. 2021-0043 or the Omnibus Guidelines on the Minimum Public Health Standards for the Safe Reopening of Institutions. This term shall also encompass specific NPIs of community mitigation strategies or public health measures that do not involve vaccines, medications, or other pharmaceutical interventions, that individuals and communities can carry out in order to reduce transmission rates, contact rates, and the duration of infectiousness of individuals in the population.

8. **On-site capacity** - refers to the number of employees or workers who can be permitted or required to be physically present at their designated workplace outside of their residences.

9. **Skeleton workforce** - refers to the on-site capacity which utilizes the smallest number of people needed for a business or organization to maintain its basic functions.

PART I.
ALERT LEVEL SYSTEM FOR COVID-19 RESPONSE

SECTION [1] GENERAL GUIDELINES

1. The DOH, shall identify the Alert Level of the areas. These areas shall follow the protocols consistent with the declared Alert Level.

2. LGUs shall submit on a daily basis to their respective Regional Inter-Agency Task Force (RIATF) such data as determined by National Government Agencies based on the template provided for by the IATF Sub-Technical Working Group on Data Analytics.

3. MPHS shall be implemented at all times consistent with the DOH Administrative Order No. 2021-0043 or the Omnibus Guidelines on the Minimum Public Health Standards for the Safe Reopening of Institutions.

4. The benefits for hazard pay and special risk allowances for all personnel in health facilities shall be applicable under the Alert Level System for the duration of the state of Public Health Emergency due to COVID-19.

5. Only hotels or accommodation establishments with valid DOT Accreditation shall be allowed to accommodate guests and clients subject to guidelines issued by the Department of Tourism and the IATF.

6. In all areas not under Alert Level 5, establishments permitted to operate under each Alert Level may be allowed additional venue/seating capacity on top of the existing allowable venue/seating capacities, as follows:

    a. An additional twenty percent (20%) if the area where such establishments are located has a vaccination coverage above seventy percent (70%) for both Priority Group A2 (senior citizens) and Priority Group A3 (adults with comorbidities), as determined by the Vaccine Cluster of the National Task Force Against COVID-19; and
    b. An additional ten percent (10%) if said establishments have been awarded Safety Seal Certificates under the Safety Seal Certification Program.

7. LGUs are enjoined to enact the necessary ordinances to enforce protocols contained in these Guidelines and to penalize, in a fair and humane manner, violations of these protocols. Law enforcement agencies are likewise strongly enjoined to observe fair and humane treatment of violators.

8. All national government agencies and instrumentalities, as well as private sector establishments, shall adopt measures to strictly implement and enforce the minimum

public health standards set by DOH and other standards set by relevant government agencies.

9. As a national policy, all LGUs shall ensure unhampered movement by land, air, or sea of all types of goods and cargoes - including their personnel and delivery vehicles - to and from their destination regardless of alert level.

10. Notwithstanding the provisions under the different alert levels as set forth under this Guidelines, the IATF may, in exceptional circumstances, suspend the application of the rules or adopt rules applicable to a different alert level, in order to address the COVID-19 situation in a region, province, city, or municipality.

11. Other COVID-19 measures not specifically provided herein such as those on border control for international travel and those for the implementation of the Philippine National Deployment and Vaccination Plan for COVID-19 Vaccines shall be governed by the appropriate IATF Resolutions.

12. Any violation of these Guidelines may be prosecuted under the appropriate local ordinance or as non-cooperation of the person or entities punishable under Section 9 par. (d) or (e), as the case may be, of Republic Act No. 11332, otherwise known as the Mandatory Reporting of Notifiable Diseases and Health Events of Public Health Concern Act, and its Implementing Rules and Regulations.

SECTION [2] GUIDELINES FOR AREAS UNDER ALERT LEVEL 5. The following protocols shall be observed in areas placed under Alert Level 5, except for portions thereof under granular lockdown:

1. Intrazonal and interzonal movement of all persons shall be limited to accessing goods and services from permitted establishments, for work in such establishments, or for such other activities allowed in this section. Any person below eighteen (18) years old, those who are over sixty-five (65) years of age, those with immunodeficiency, comorbidity, or other health risks, and pregnant women shall be required to remain in their residences at all times, except for obtaining essential goods and services, or for work in industries and offices or such other activities permitted in this Section.

2. Only the following establishments, persons, or activities are allowed to operate, work, or be undertaken for the duration of the Alert Level 5:

    a. With full on-site capacity:

        i. Public and private hospitals;
        ii. Health, emergency, and frontline services, including those provided by dialysis centers, chemotherapy centers, HMOs, health insurance

providers, disaster risk reduction management officers, and public safety officers, and the like;

iii. Manufacturers of medicines and vitamins, medical supplies, devices, and equipment, including suppliers of input, packaging, and distribution;
iv. Industries involved in agriculture (crops, fruits, vegetables, livestock, and poultry), forestry, fishery, and such other components of the food value chain and their workers, including farmers and fisherfolks;
v. Logistics service providers (delivery and courier services; cargo handling; warehousing; trucking; freight forwarding; shipping, port and terminal operators and contractors and ancillary services (i.e. drivers, conductors, terminal workers);
vi. Essential and priority construction projects, whether public or private, in accordance with the guidelines issued by the Department of Public Works and Highways (DPWH) including contractors, subcontractors, and consultants of the Department of Transportation for the construction of Build Build Build flagship infrastructure projects;
vii. Manufacturing related to food and other essential goods such as but not limited to soap and detergents, diapers, personal hygiene products, toilet paper, and wet wipes, and disinfectants;
viii. Companies that manufacture, distribute, and/or supply equipment or products necessary to perform construction or maintenance works, such as cement and steel, or spare parts;
ix. Essential retail trade and service establishments such as public markets, supermarkets, grocery stores, convenience stores, pharmacies or drug stores, hardware, office supplies, bicycle shops, laundry shops, and water-refilling stations;
x. Food preparation establishments such as kiosks, commissaries, restaurants, and eateries, but limited to take-out and delivery;
xi. Public and private financial service providers involved in the distribution of government grants and amelioration subsidies;
xii. Business process outsourcing establishments (BPOs), and export-oriented businesses, including mining and quarrying activities; and
xiii. Public transport providers and operators;

b. At a maximum of fifty percent (50%) on-site capacity:

i. Media establishments and their total permanent staff complement, inclusive of reporters and other field employees.

c. With an on-site skeleton workforce:

i. Dental, rehabilitation, optometry, and other medical clinics for the treatment of illness or injuries. Provided, that there is strict observance of infection prevention and control protocols. Provided, further, that dental procedures shall be limited to emergency cases only and that the wearing of full Personal Protective Equipment (PPEs) by dentists and attendants shall be mandatory. Provided, finally, that home service therapy for Persons with Disabilities (PWDs) shall be allowed;

ii. Veterinary clinics;

iii. Banks, money transfer services, including pawnshops only insofar as performing money transfer functions, microfinance institutions, and credit cooperatives, including their armored vehicle services, if any;

iv. Capital markets, including but not limited to the Bangko Sentral ng Pilipinas, Securities and Exchange Commission, Philippine Stock Exchange, Philippine Dealing and Exchange Corporation, Philippine Securities Settlement Corporation, and Philippine Depository and Trust Corporation;

v. Water supply and janitorial/sanitation services and facilities, including waste disposal services, as well as property management and building utility services;

vi. The energy sector (oil, gas, and power companies), their third-party contractors and service providers, including employees involved in electric transmission and distribution, electric power plant and line maintenance, electricity market and retail suppliers, as well as those involved in the exploration, operations, trading and delivery of coal, oil, crude or petroleum and by-products (gasoline, diesel, liquefied petroleum gas or LPG, jet oil, kerosene, lubricants), including gasoline stations, refineries, LPG stations, and depots or any kind of fuel used to produce power;

vii. Telecommunications companies, internet service providers, cable television providers, including those who perform indirect services such as the technical, sales, and other support personnel, as well as the employees of their third-party contractors doing sales, installation, maintenance, and repair works;

viii. Airline and aircraft maintenance, pilots and crew, and employees of aviation schools for purposes of the pilot's recurrent training for flight proficiency and type rating using simulator facilities; and ship captains and crew, including shipyard operations and repair;

ix. Funeral and embalming services;

x. Security personnel licensed by the PNP - Supervisory Office for Security and Investigation Agencies;

xi. Printing establishments authorized by the Bureau of Internal Revenue and those contracted by other government agencies;

xii. Establishments engaged in repair and maintenance of machinery and equipment, for households and essential permitted establishments;
xiii. Establishments engaged in repair and maintenance of motorized and non-motorized vehicles, including the sale of spare parts;
xiv. Leasing of real and personal properties;
xv. Employment activities that involve the recruitment and placement for permitted sectors;
xvi. Teachers, professors and other staff for purposes of conducting online/offline, and flexible classes, completion of grades, and processing of student credentials, requirements and documents;
xvii. Lawyers only when required to provide on-site legal representation necessary to protect rights of persons, whether natural or juridical; and
xviii. All other establishments, to the extent necessary for the buying and selling of consumer goods or services via the internet.

All other businesses, persons, or activities, shall not be allowed to operate, work, or be undertaken on-site during Alert Level 5.

3. Notwithstanding the foregoing, the Department of Trade and Industry (DTI) is hereby authorized to issue a negative list of other industries that shall remain prohibited in areas under Alert Level 5.

4. Agencies and instrumentalities of the government shall be fully operational, with a skeleton workforce on-site and the remainder under alternative work arrangements as approved by the head of agency unless a greater on-site capacity is required in agencies providing health and emergency frontline services, laboratory and testing services, border control, or other critical services, in accordance with the relevant rules and regulations issued by the Civil Service Commission (CSC).

5. The co-equal or independent authority of the legislature (Senate and the House of Representatives), the judiciary (the Supreme Court, Court of Appeals, Court of Tax Appeals, Sandiganbayan, and the lower courts), the Office of the Ombudsman, and the Constitutional Commissions, to implement any alternative work arrangements, is recognized.

6. Officials and employees of foreign diplomatic missions and international organizations accredited by the Department of Foreign Affairs (DFA), whenever performing diplomatic functions and subject to the guidelines issued by the DFA, may operate with an on-site skeleton workforce.

7. Gatherings outside of residences shall be prohibited. Gatherings at residences with any person outside of one's immediate household shall likewise be prohibited. However, gatherings that are essential for the provision of health services, government services,

or humanitarian activities authorized by the appropriate government agency or instrumentality shall be allowed.

Pastors, priests, rabbis, imams, or other religious ministers and their assistants may conduct religious services performed through online video recording and transmission, necrological services, wakes, inurnment, and funerals, and they shall be allowed to move for such purposes. Corollary, immediate family members of the deceased who died of causes other than COVID-19 and for the cremains of COVID-19 shall be allowed to move from their residences to attend the wake or interment of the deceased upon satisfactory proof of their relationship with the latter, fully complying with the prescribed minimum public health standards for the duration of the activity.

8. Face-to-face or in-person classes at all levels shall be suspended. The education sector shall operate in accordance with the guidelines of the Commission on Higher Education (CHED) for higher education, Technical Educational and Skills Development Authority (TESDA) for technical vocational education and training, and Department of Education (DepEd) for basic education.

9. The road, rail, maritime, and aviation sectors of public transportation shall be allowed to operate at such capacity and protocols in accordance with guidelines issued by the Department of Transportation (DOTr).

10. Law enforcement agencies shall recognize any of the following IDs: (i) IATF IDs issued by the regulatory agencies with jurisdiction over permitted establishments or persons, (ii) valid IDs or other pertinent documentation issued by accrediting organizations or establishments allowed under Alert Level 5, and, (iii) if required by the LGU, local IDs for availing of essential goods and services. No other IDs or passes specifically exempting persons from alert level shall be required of workers of permitted establishments and/or offices without prejudice to requiring the presentation of other documents establishing the nature of their work.

11. The movement of cargo/delivery vehicles, as well as vehicles used by public utility companies, shall be unhampered. Shuttle services of permitted establishments shall not be subject to an ID system but shall maintain compliance with minimum public health standards.

12. Private corporations are encouraged to process payrolls online. Payroll managers and such other employees required for the processing of payroll shall be allowed to travel to their respective offices during Alert Level 5.

13. Uniform curfew hours may be imposed by LGUs, subject to the guidelines issued by the Department of the Interior and Local Government (DILG). Workers, cargo vehicles, public transportation, and operating hours of permitted establishments, however, shall not be restricted by such curfew.

14. (a) The movement of the following authorized persons outside their residences (APOR), by land, sea, or air, within and across areas placed under Alert Level 5 shall be allowed: (1) health and emergency frontline services and uniformed personnel, (2) government officials and employees on official travel, (3) duly-authorized humanitarian assistance actors (HAAs), especially those transporting medical supplies and laboratory specimens related to COVID-19, and other relief and humanitarian assistance, (4) persons traveling for medical or humanitarian reasons, and those leaving their residence to be vaccinated (with proof of schedule), and persons availing of DFA consular services (with confirmed appointments), (5) persons going to and from the airport including Overseas Filipino Workers (OFWs) carrying Overseas Employment Certificates, (6) any person whose purpose of travel is for a work, business, or activity that is also permitted in areas under Alert Level 5, and (7) public utility vehicle operators. The PNP shall be authorized to promulgate and regularly update the list of APORs. Authorized shuttle services shall be allowed to travel within and across areas placed under Alert Level 5, with priority given to persons rendering health and emergency frontline services.

SECTION [3] GUIDELINES FOR AREAS UNDER ALERT LEVEL 4. The following protocols shall be observed in areas placed under Alert Level 4, except for portions thereof under granular lockdown:

1. Intrazonal and interzonal travel shall be allowed subject to the reasonable regulations of the LGU of destination, except for those (i) below eighteen (18) years of age, and (ii) belonging to the vulnerable population, namely, those who are over sixty-five (65) years of age, those with immunodeficiencies, comorbidities, or other health risks, and pregnant women, provided that:

    a. Those below eighteen (18) years of age, and those belonging to the vulnerable population, shall be allowed access to obtain essential goods and services, or for work in permitted industries and offices in accordance with existing labor laws, rules, and regulations.

    b. Fully vaccinated individuals belonging to the vulnerable population and those below eighteen (18) years of age shall be allowed to participate in the following activities:

        i. Specialized markets of the Department of Tourism such as Point-to-Point Travel subject to the reasonable regulations of the LGU of destination, and *Staycations*; and
        ii. Individual outdoor exercises even beyond the general area of their respective residences. Provided that, the minimum public health standards and precautions such as the wearing of face masks and the maintenance of social distancing protocols are observed.

2. Fully vaccinated individuals, including those belonging to the vulnerable population, may participate in the permitted activities under this Section.

3. The following establishments and/or activities characterized as high-risk for transmission **shall not be allowed to operate**, **or be undertaken** in areas classified under Alert Level 4:

    a. Face to face or in-person classes for basic education, except those approved by the IATF and/or the Office of the President;
    b. Face-to-face or in-person classes for higher education or for technical, vocational education and training, except those approved by the IATF and/or the Office of the President;
    c. All contact sports, whether indoor or outdoor;
    d. Cinemas and movie houses;
    e. Funfairs/*peryas* and kid amusement industries such as playgrounds, playroom, and kiddie rides;
    f. Venues with live voice or wind-instrument performers and audiences such as in karaoke bars, bars, clubs, concert halls, and theaters;
    g. Casinos, horse racing, cockfighting and operation of cockpits, lottery and betting shops, and other gaming establishments except as may be authorized by the IATF or the Office of the President; and
    h. Gatherings in residences with individuals not belonging to the same household.

4. The following establishments, or activities, shall be allowed to operate, or be undertaken at a **maximum of 10% indoor venue capacity for fully vaccinated individuals only and 30% outdoor venue capacity provided that they have been issued a Safety Seal Certification**. Provided further, that all on-site workers/employees of these establishments, or organizers of activities are fully vaccinated against COVID-19 and minimum public health standards shall be strictly maintained. Provided further still, that there is no objection from the LGU where these activities may take place:

    a. Venues for meetings, incentives, conferences, and exhibitions (MICE);
    b. Permitted venues for social events such as parties, wedding receptions, engagement parties, wedding anniversaries, debut and birthday parties, family reunions, and bridal or baby showers;
    c. Visitor or tourist attractions such as libraries, archives, museums, galleries, exhibits, parks, plazas, public gardens, scenic viewpoints or overlooks, and the like;
    d. Amusement parks and theme parks; and

e. Recreational venues such as internet cafes, billiard halls, amusement arcades, bowling alleys, skating rinks, archery halls, swimming pools, and similar venues.

5. The following establishments, or activities, shall be allowed to operate, or be undertaken at a **maximum of 10% indoor venue capacity for fully vaccinated individuals only and 30% outdoor venue capacity**. Provided that all on-site workers/employees of these establishments, or organizers of activities are fully vaccinated against COVID-19 and minimum public health standards shall be strictly maintained. Provided further, that there is no objection from the LGU where these activities may take place:

   a. In-person religious gatherings. Provided that gatherings shall be limited to the conduct of religious worship and/or service, and processions and other similar mobile religious gatherings shall not be allowed. Provided further, that pastors, priests, rabbis, imams, or other religious ministers and the assistants of these religious congregations have been fully vaccinated;
   b. Licensure or entrance/qualifying examinations administered by their respective government agency, and specialty examinations authorized by the IATF subject to the health and safety guidelines as approved by the IATF;
   c. Dine-in services in food preparation establishments such as kiosks, commissaries, restaurants, and eateries, subject to DTI sector-specific protocols;
   d. Personal care establishments such as barbershops, hair spas, hair salons, and nail spas, and those offering aesthetic/cosmetic services or procedures, make-up services, salons, spas, reflexology, and other similar procedures including home service options, subject to the sector-specific protocols of the DTI;
   e. Fitness studios, gyms, and venues for non-contact exercise and sports, subject to DTI sector-specific protocols. Provided that patrons/clients and workers/employees wear face masks at all times and that no group activities are conducted; and,
   f. Film, music, and television production, subject to the joint guidelines as may be issued by the DTI, Department of Labor and Employment (DOLE), and the DOH.

6. Gatherings for necrological services, wakes, inurnment, funerals for those who died of causes other than COVID-19, and for the cremains of the COVID-19 deceased, shall be allowed, provided that the same shall be limited to immediate family members, upon satisfactory proof of their relationship with the deceased and with full compliance with the prescribed minimum public health standards.

7. Agencies and instrumentalities of the government shall remain to be fully operational and shall adhere to at least a 40% on-site workforce. Those assigned to work off-site shall be subject to alternative work arrangements as approved by the head of agency in accordance with the relevant rules and regulations issued by the Civil Service Commission (CSC). Heads of agencies providing health and emergency frontline services, laboratory and testing services, border control, or other critical services shall operate at a workforce beyond the minimum as may be necessary to deliver their mandates.

SECTION [4] GUIDELINES FOR AREAS UNDER ALERT LEVEL 3. The following protocols shall be observed in areas placed under Alert Level 3, except for portions thereof under granular lockdown:

1. Intrazonal and interzonal movement shall be allowed. However, reasonable restrictions may be imposed by the LGUs, which should not be stricter as those prescribed under higher alert levels and subject to the oversight, monitoring, and evaluation of their respective RIATF. Provided, that those below eighteen (18) years of age, and those belonging to the vulnerable population, shall be allowed access to obtain essential goods and services, or for work in permitted industries and offices in accordance with existing labor laws, rules, and regulations. Individual outdoor exercises shall also be allowed for all ages regardless of comorbidities or vaccination status.

2. The following establishments and/or activities characterized as high-risk for transmission shall **not be allowed to operate, or be undertaken** in areas classified under Alert Level 3:

    a. Face to face or in-person classes for basic education, except those previously approved by the IATF and/or the Office of the President;
    b. Contact sports, except those conducted under a bubble-type setup as provided for under relevant guidelines adopted by the IATF, Games and Amusement Board, and Philippine Sports Commission, and approved by the LGU where such games shall be held;
    c. Funfairs/*peryas* and kid amusement industries such as playgrounds, playroom, and kiddie rides;
    d. Venues with live voice or wind-instrument performers and audiences such as in karaoke bars, clubs, concert halls, and theaters;
    e. Casinos, horse racing, cockfighting and operation of cockpits, lottery and betting shops, and other gaming establishments except as may be authorized by the IATF or the Office of the President; and
    f. Gatherings in residences with individuals not belonging to the same household.

3. The following establishments, or activities, shall be allowed to operate or be undertaken at a **maximum of 30% indoor venue capacity for fully vaccinated individuals only and 50% outdoor venue capacity**. Provided that all on-site workers/employees of these establishments, and organizers of activities are fully vaccinated against COVID-19 and MPHS shall be strictly maintained. Provided further, that there is no objection from the LGU where these activities may take place.

    a. Venues for meetings, incentives, conferences, and exhibitions (MICE);
    b. Permitted venues for social events such as parties, wedding receptions, engagement parties, wedding anniversaries, debut and birthday parties, family reunions, and bridal or baby showers;
    c. Visitor or tourist attractions such as libraries, archives, museums, galleries, exhibits, parks, plazas, public gardens, scenic viewpoints or overlooks, and the like;
    d. Amusement parks or theme parks;
    e. Recreational venues such as internet cafes, billiard halls, amusement arcades, bowling alleys, skating rinks, archery halls, swimming pools, and similar venues;
    f. Cinemas and movie houses;
    g. Limited face-to-face or in-person classes for higher education and for technical-vocational education and training;
    h. In-person religious gatherings; gatherings for necrological services, wakes, inurnment, and funerals for those who died of causes other than COVID-19 and for the cremains of the COVID-19 deceased;
    i. Licensure or entrance/qualifying examinations administered by their respective government agency, and specialty examinations authorized by the IATF subject to the health and safety guidelines as approved by the IATF;
    j. Dine-in services in food preparation establishments such as kiosks, commissaries, restaurants, and eateries, subject to DTI sector-specific protocols;
    k. Personal care establishments such as barbershops, hair spas, hair salons, and nail spas, and those offering aesthetic/cosmetic services or procedures, make-up services, salons, spas, reflexology, and other similar procedures including home service options, subject to the sector-specific protocols of the DTI;
    l. Fitness studios, gyms, and venues for non-contact exercise and sports, subject to DTI sector-specific protocols. Provided that patrons/clients and workers/employees wear face masks at all times and that no group activities are conducted; and
    m. Film, music, and television production, subject to the joint guidelines as may be issued by the DTI, DOLE, and the DOH.

4. Agencies and instrumentalities of the government shall remain to be fully operational and shall adhere to at least a 60% on-site workforce. Those assigned to work off-site shall be subject to alternative work arrangements as approved by the head of agency in accordance with the relevant rules and regulations issued by the Civil Service Commission (CSC). Heads of agencies providing health and emergency frontline services, laboratory and testing services, border control, or other critical services shall operate at a workforce beyond the minimum as may be necessary to deliver their mandates.

SECTION [5] GUIDELINES FOR AREAS UNDER ALERT LEVEL 2. The following protocols shall be observed in areas placed under Alert Level 2, except for portions thereof under granular lockdown:

1. Intrazonal and interzonal movement shall be allowed. However, reasonable restrictions may be imposed by the LGUs, which should not be stricter as those prescribed under higher alert levels and subject to the oversight, monitoring, and evaluation of their respective RIATF.

2. Casinos, horse racing, cockfighting and operation of cockpits, lottery and betting shops, and other gaming establishments shall **not be allowed to operate, or be undertaken** in areas classified under Alert Level 2 except as may be authorized by the IATF or the Office of the President.

3. The following establishments, or activities, shall be allowed to operate, or be undertaken at a **maximum of 50% indoor venue capacity for fully vaccinated individuals and those below 18 years of age, even if unvaccinated, and 70% outdoor venue capacity**. Provided that all on-site workers/employees of these establishments, or organizers of activities are fully vaccinated against COVID-19 and MPHS shall be strictly maintained. Provided further, that there is no objection from the LGU where these activities may take place.

    a. Venues for meetings, incentives, conferences, and exhibitions (MICE);
    b. Permitted venues for social events such as parties, wedding receptions, engagement parties, wedding anniversaries, debut and birthday parties, family reunions, and bridal or baby showers;
    c. Visitor or tourist attractions such as libraries, archives, museums, galleries, exhibits, parks, plazas, public gardens, scenic viewpoints or overlooks, and the like;
    d. Amusement parks or theme parks;
    e. Recreational venues such as internet cafes, billiard halls, amusement arcades, bowling alleys, skating rinks, archery halls, swimming pools, and similar venues;
    f. Cinemas and movie houses;

g. Limited face-to-face or in-person classes for basic education subject to prior approval of the Office of the President;
h. Limited face-to-face or in-person classes for higher education and for technical-vocational education and training;
i. In-person religious gatherings; gatherings for necrological services, wakes, inurnment, and funerals for those who died of causes other than COVID-19 and for the cremains of the COVID-19 deceased;
j. Licensure or entrance/qualifying examinations administered by their respective government agency, and specialty examinations authorized by the IATF subject to the health and safety guidelines as approved by the IATF;
k. Dine-in services of food preparation establishments such as kiosks, commissaries, restaurants, and eateries, subject to DTI sector-specific protocols;
l. Personal care establishments such as barbershops, hair spas, hair salons, and nail spas, and those offering aesthetic/cosmetic services or procedures, make-up services, salons, spas, reflexology, and other similar procedures including home service options, subject to the sector-specific protocols of the DTI;
m. Fitness studios, gyms, and venues for individual non-contact exercise and sports; Provided that patrons/clients and workers/employees wear face masks at all times and subject to DTI sector-specific protocols.
n. Film, music, and television production subject to the joint guidelines as may be issued by the DTI, DOLE, and the DOH;
o. Contact sports approved by the LGU where such games shall be held;
p. Funfairs/*peryas* or kid amusement industries such as playgrounds, playroom, and kiddie rides;
q. Venues with live voice or wind-instrument performers and audiences such as in karaoke bars, clubs, concert halls, and theaters; and
r. Gatherings in residences with individuals not belonging to the same household.

4. Agencies and instrumentalities of the government shall remain to be fully operational and shall adhere to at least a 80% on-site workforce. Those assigned to work off-site shall be subject to alternative work arrangements as approved by the head of agency in accordance with the relevant rules and regulations issued by the Civil Service Commission (CSC). Heads of agencies providing health and emergency frontline services, laboratory and testing services, border control, or other critical services shall operate at a workforce beyond the minimum as may be necessary to deliver their mandates.

SECTION [6] GUIDELINES FOR AREAS UNDER ALERT LEVEL 1. The following protocols shall be observed in areas placed under Alert Level 1, except for portions thereof

under granular lockdown, and without prejudice to the minimum public health standards and health and safety protocols issued by national government agencies specific to their sector:

I. PREVENT

A. All health facilities shall implement the necessary and appropriate engineering and administrative controls, and use appropriate personal protective equipment in accordance with the latest Infection Prevention & Control (IPC) guidelines for health facilities.

B. All public and private establishments, organizers of events, and local governments shall implement the following protocols:

1. **Administrative controls**.

a. All private offices and workplaces, including public and private construction sites, may operate at full 100% capacity (consistent with national issuances on vaccination requirements for on-site work). However, they may continue to provide flexible and alternative work arrangements as deemed appropriate based on function or individual risk.

b. Agencies and instrumentalities of the government shall adhere to 100% on-site workforce. Off-site work shall be under such work arrangements subject to relevant rules and regulations issued by the Civil Service Commission and the Office of the President.

c. All establishments and/or activities prohibited or allowed to operate and/or be undertaken in limited capacity under Alert Levels 5, 4, 3, and 2 shall be allowed to operate and/or be undertaken at full 100% capacity, subject to the requirement of presentation of proof of full vaccination before participating in mass gatherings or entry into indoor establishments as specified under Item VI of Section [6] of this Guidelines.

d. Public transportation in areas under Alert Level 1 shall be at full seating capacity. For intrazonal and interzonal travels involving public land transportation between an area with a higher alert level classification and an area under Alert Level 1, the passenger capacity shall be that which has the lower passenger capacity rate between the point of origin and point of destination. For aviation, maritime and rail public transport operating in and out of Alert Levels 1 areas, the passenger capacity will be at 100%. The use of acrylic and/or plastic dividers shall not be required. The use of the Safe, Swift and Smart Passage (S-PaSS) travel

management system shall likewise not be required for interzonal travel to areas under Alert Level 1.

e. Designate a Safety and Health Officer/s who shall ensure, monitor, and evaluate proper implementation and strict observance of the minimum public health standards.

f. Prepare a contingency plan for the establishment which shall include coordination with the local government and health care provider networks, management of staff and visitors with COVID-19 symptoms, processes for establishment lockdown and disinfection, and alternative processes in case of escalation of Alert Level status.

g. Regularly disinfect high-risk areas such as but not limited to areas for gatherings, highly touched surfaces, and frequently visited areas such as entrance and exit points, restrooms, hallways, elevators using Food and Drug Administration (FDA)- approved disinfectants.

h. National government agencies and local government units endeavor to include health and safety in routine assessments to be conducted regularly.

2. **Engineering controls**.

a. Ensure adequate air exchange and ventilation, consistent with Department of Labor and Employment (DOLE) Department Order No. 224-21 *Guidelines on Ventilation for Workplaces and Public Transport to Prevent and Control the Spread of COVID-19, viz*:

i. Ensure adequate air quality and ventilation, to wit:

1. If possible, windows shall be open;
   - Ensure that nearby spaces of open windows are free from toxic gases and pollutants;

Whether windows may or may not be opened, supply-only ventilation fans (i.e, floor, table top, pedestal fans) are provided and should be placed where air flow from these fans shall not be from person to person;

2. Exhaust fans should be continuously operating while there are occupants in the area;
3. Additional exhaust fans may be added to improve air exchange to achieve at least 6 air changes per hour; increasing the number of exhaust fans can increase the number of air changes per hour;

4. If possible, establishments are highly encouraged to conduct the following air quality measurements: Air Change per hour -recommend at least 6 air changes per hour; and/or
5. Carbon Dioxide (CO_2) Levels: recommend less than 1000 parts per million or ppm.
6. Air ducts of HVAC systems should be maintained and cleaned regularly as determined by the safety officer or technical expert.

b. All land-based transport terminals are to ensure the compliance to sanitary standards, among others, that are set in accordance with Republic Act No. 11311 otherwise known as An Act to Improve Land Transportation Terminals, Stations, Stops, Rest Areas and Roll-On/Roll-Off Terminals, Appropriating Funds Therefor and for Other Purposes, and its Implementing Rules and Regulations.

c. Install structures enabling active transportation (e.g. bike racks).

d. Maintain smoke-free and vape-free environment.

e. Support mental, and psychosocial needs, especially of vulnerable groups.

f. Make available context-specific and localized health and safety education materials.

g. Set up a mechanism for provision of care and referral for patients with symptoms or other emergency health conditions.

h. Install/maintain hand hygiene and sanitation facilities with the following:
   i. Adequate and safe water supply
   ii. Hand washing station or sink
   iii. Soap and water or 70% Isopropyl (or Ethyl) Alcohol
   iv. Hands-free trash receptacles

i. Install visual cues or signages on mask wearing, hand hygiene, DOH hotlines, and other health and safety reminders (resources downloadable from www.healthypilipinas.ph).

j. Promote and incentivize use of interventions that ensure health and safety in all establishments, such as:
   i. Using outdoor spaces or having outdoor options
   ii. Ensure adequate air quality and ventilation, as specified for under Par. B(2)(a)(i) above.

k. The use of foot baths, disinfection tents, misting chambers, or sanitation booths for preventing and controlling COVID-19 transmission are **not recommended.** Temperature checking prior to the entry in any establishment may be dispensed with.

l. The use of plastic/acrylic barriers/dividers are **optional**, as appropriate.

m. The Safety Seal Certification Program of the national government to mark their compliance with minimum public health standards is optional but all establishments are encouraged to use it as a marketing tool.

3. **Wearing of Face Masks**

a. At all times, well-fitted masks shall be worn properly, whether outdoors or in indoor private or public establishments, including in public transportation by land, air, or sea, except for the following instances:
   i. Eating and drinking;
   ii. Participating in team and individual sports in venues where ventilation standards can be maintained;
   iii. Practicing outdoor sports/exercise activities where physical distance can be maintained.

b. All establishments shall ensure implementation of national protocols on the proper use, handling, and disposing of appropriate personal protective equipment.

II. DETECT

A. Contact Tracing

1. Individuals, who have tested positive for COVID-19, shall inform their close contacts to trigger quarantine for unvaccinated close contacts and symptom monitoring for vaccinated close contacts.

2. Use of health declaration forms / paper-based contact tracing shall **not be required** for all the agencies and establishments.

3. Use of digital contact tracing such as the StaySafe.PH application **is optional** for all the agencies and establishments.

4. Establishments/employers will not have to provide a separate report to DOLE for COVID-19. Establishments/Employers should include COVID-19 cases report as part of work accident illness report form.

5. COVID-19 reporting to the Local Epidemiology and Surveillance Units and the Epidemiology Bureau of the DOH will continue until the establishment of a sentinel surveillance system for workplaces. Guidelines for this system shall follow.

B. Testing Prioritization

1. Implement testing protocols consistent with national guidelines, which may be stricter for individuals who are unvaccinated or have higher exposure risk pursuant to IATF Resolution No. 148-B and No. 149, s. 2021.

2. Testing using RT-PCR shall be recommended and prioritized for:
   a. Priority Groups A2 (persons above 60 years old) and A3 (persons with comorbidities) who are at risk for developing severe disease, especially for instances where the result of testing will affect the clinical management and use of COVID-19 therapeutics.
   b. Priority Group A1 or healthcare workers for groups at highest risk for infection such as deemed necessary.

3. Testing shall be optional for other groups not stated above, including for community level actions wherein case management of probable and confirmed cases remain the same. Specifically:
   a. Testing shall NOT be recommended for asymptomatic close contacts unless symptoms will develop, and should immediately isolate regardless of test results. Instead, symptom monitoring is recommended. Should testing still be used, testing should be done at least 5 days from the day of last exposure.
   b. Testing shall NOT be recommended for screening asymptomatic individuals.

4. Testing using antigen tests shall be recommended only for symptomatic individuals and in instances wherein RT-PCR is not available, consistent with previously issued guidelines.

5. Hospital Infection Prevention and Control Committees (HIPCC) may implement testing protocols in health facilities for health workers and patients based on their assessment of risk and benefit.

III. ISOLATE AND QUARANTINE

A. Updated quarantine protocols for incoming international travelers shall be consistent with IATF Resolution No. 160 A/B (Summary in Annex A).

B. Updated isolation and quarantine protocols for general public & healthcare workers and authorized sectors shall be consistent with DOH Department Memorandum No. **2022-0013** (Summary in Annex B).

C. Establishments are no longer required to set-up isolation facilities within the workplace.

IV. TREAT

A. The management of COVID-19 shall be consistent with the latest recommendation from the Philippine COVID-19 Living Recommendations, which can be accessed through this link: https://www.psmid.org/philippine-covid-19-living-recommendations/

B. Close contacts, asymptomatic confirmed cases, and mild to moderate confirmed cases shall be managed at the primary care level (e.g. health centers, private clinics thru face to face or teleconsultation). Health facilities are encouraged to adopt / scale-up telemedicine to facilitate delivery of appropriate health services.

C. Confirmed cases with severe and critical symptoms shall be managed in the appropriate health care facility.

V. REINTEGRATE

A. Isolation can be discontinued upon completion of the recommended isolation period, provided that they do not have fever for at least 24 hours without the use of any antipyretic medications, and shall have improvement of respiratory signs and symptoms.

B. Repeat tests shall **not be required** for the safe reintegration into the community.

C. For the purpose of claiming sick leave, health benefits, or other relevant processes wherein the proofs of COVID-19 management are necessary, a medical certification may suffice provided it should include the following minimum

information: name of patient, severity of symptoms, diagnosis as probable or confirmed COVID-19, and date of end of quarantine and/or isolation period.

D. To ensure promotion of their psychosocial well-being, individuals in quarantine and isolation are recommended to maintain and continue lines of communication to family and friends. They may also download the DOH Lusog-Isip Mobile Application for free (available in both Apple store or Google play store) or access the National Center for Mental Health (NCMH) Crisis Hotline or the DOH Regional Helplines for mental health and psychosocial support concerns.

VI. VACCINATE

A. Individuals eighteen (18) years old and above will be required to present proof of full vaccination before participating in mass gatherings or entry into indoor establishments, such as but not limited to:
   1. In-person religious gatherings; gatherings for necrological services, wakes, inurnment, and funerals for those who died of causes other than COVID-19 and for the cremains of the COVID-19 deceased;
   2. All indoor dine-in services of food preparation establishments such as kiosks, commissaries, restaurants, and eateries. For outdoor or *al fresco* dining and take out channels, no proof of full vaccination is required;
   3. All indoor personal care establishments such as barbershops, hair spas, hair salons, and nail spas, and those offering aesthetic/cosmetic services or procedures, make-up services, salons, spas, reflexology, and other similar procedures including home service options;
   4. Fitness studios, gyms, and venues for exercise and sports;
   5. All indoor cinemas or movie houses operating at full capacity;
   6. Meetings, incentives, conferences, exhibition events, and permitted venues for social events such as parties, wedding receptions, engagement parties, wedding anniversaries, debut and birthday parties, family reunions, and bridal or baby showers ;
   7. Venues with live voice or wind-instrument performers and audiences such as in karaoke bars, clubs, concert halls, and theaters;
   8. Indoor ancillary establishments in hotels and other accommodation establishments; and
   9. Venues for election-related events.

Proof of full vaccination shall be required before entry in the list of establishments identified under the principles of 3C's (Closed, Crowded, and Close Contact) strategy against COVID-19. (See Annex C). Children ages seventeen (17) and below shall not be required to present proof of full vaccination status.

PART II
GUIDELINES ON THE IMPLEMENTATION OF GRANULAR LOCKDOWNS

Section [1] AUTHORITY TO DECLARE. The authority to impose granular lockdown shall be given to the city and municipal mayors with respect to their component barangays subject to the concurrence of the Regional Inter-Agency Task Force (RIATF), and individual houses where one household member has been confirmed, residential buildings, streets, blocks, *puroks*, subdivisions, and/or villages within their jurisdiction.

SECTION [2] PARAMETERS FOR DECLARATION AND IMPLEMENTATION. The epidemiological parameters in the declaration of a granular lockdown, including specific interventions and activities to be conducted in such areas, shall comply with the National Task Force COVID-19 Memorandum Circular No. 2 dated 15 June 2020 or the Operational Guidelines on the Application of the Zoning Containment Strategy in the Localization of the National Action Plan Against COVID-19 Response.

SECTION [3] DURATION. Granular lockdowns shall be for a period of not less than fourteen (14) days.

SECTION [4] EFFECTIVITY.

1. Declaration of granular lockdowns by local chief executives shall be provisionally effective immediately and shall include due notice to the RIATF. The RIATF shall immediately act on the said declaration.
2. The IATF retains its mandate to impose and/or lift lockdowns on highly urbanized cities and independent component cities.

SECTION [5] SECURITY. The Philippine National Police (PNP) shall ensure peace and order at all times. They shall make sure that security protocols are maintained in lockdown areas.

SECTION [6] MOVEMENT. The privilege to enter and exit areas under Alert Level 5, granted to Authorized Persons Outside Residences as provided under Par. 14 Part I Section of these Guidelines, shall not apply in areas under granular lockdowns. Only health care workers (HCWs) and non-health personnel working in hospitals, laboratories, dialysis facilities, and community healthcare workers if their institutions are unable to provide accommodation for their personnel, and uniformed personnel tasked to enforce the granular lockdown, shall be allowed to move within, into, and out of the area under granular lockdown.

The following shall be allowed to enter and/or exit granular lockdown areas for specific purposes:

1. Overseas Filipino Workers bound for international travel, and those returning to their respective residences after having completed facility-based quarantine upon arrival;
2. Individuals whose home or residence are located within an area under granular lockdown may enter the said area for the sole purpose of returning to their respective homes or residences but must thereafter remain therein for the duration of the granular lockdown;
3. Individuals under exceptional circumstances such as, but not limited to, those seeking urgent medical attention as validated by the municipal/city health officer; and
4. Food and essential items provided that they can only be picked up and/or unloaded at border collection points designated by the LGU.

SECTION [7] ASSISTANCE TO AFFECTED INDIVIDUALS. Households within areas under granular lockdown shall be provided assistance by their respective LGUs and the Department of Social Welfare and Development (DSWD), in accordance with their relevant guidelines.

SECTION [8] REPORTING.

1. LGUs shall report details of their granular lockdowns to the Department of the Interior and Local Government following a prescribed format which shall thereafter be submitted to their respective NTF Regional Task Force.
2. RTFs shall monitor all areas under granular lockdowns in the region, and shall provide monitoring reports to the National Task Force Against COVID-19.

SECTION [9] INCORPORATION. Succeeding issuances of the Department of the Interior and Local Government relative to the imposition of granular lockdowns, if any, shall form an integral part of these Guidelines.

Annex A: Updated Quarantine and Isolation Protocols for Travelers (IATF Resolution No. 160 A/B)

	**Filipino Nationals**	**Foreign Nationals authorized to enter the Philippines**
Fully vaccinated adult	No mandatory facility-based quarantine.<br>Wear masks and self-monitor for any sign or symptom for seven (7) days	No mandatory facility-based quarantine.<br>Wear masks and self-monitor for any sign or symptom for seven (7) days
Unvaccinated, partially vaccinated adult	Facility-based quarantine until the release of their negative RT-PCR test taken on the fifth (5th) day from date of arrival.<br>Home quarantine until their fourteenth (14th) day from date of arrival.	Not applicable since they are not allowed to enter the Philippines.
Unvaccinated minor children*	Follow the quarantine protocols of their parent/s or an accompanying adult/guardian traveling with them	Follow the quarantine protocols of their parent/s or an accompanying adult/guardian traveling with them

* All children in facility quarantine or isolation shall be accompanied by a guardian in the quarantine or isolation facility. Parents or guardians that are not confirmed COVID-19 cases may accompany the COVID-19 confirmed child provided risks and benefits are explained, informed consent is provided, and the adult has no comorbidity putting them at risk for severe disease and death.

Annex B: Updated Quarantine and Isolation Protocols for General Public & Healthcare workers and authorized sectors

		**General Public**	**Healthcare workers and authorized sectors****
**QUARANTINE**			
**Asymptomatic close contact*****	Fully vaccinated	0 days	Same
	Partially Vaccinated or Unvaccinated	At least 14 days from exposure	Same
**ISOLATION**			
**Asymptomatic confirmed case**	Fully vaccinated	At least 7 days* from positive test (sample collection date)	Same<br>IPCC may shorten up to 5 days if with booster
	Partially Vaccinated or Unvaccinated	At least 10 days* from positive test (sample collection date)	Same
**Symptomatic, suspect, probable or confirmed case with MILD symptoms**	Fully vaccinated	At least 7 days* from onset of symptoms	Same<br>IPCC may shorten up to 5 days if with booster
	Partially Vaccinated or Unvaccinated	At least 10 days* from onset of symptoms	Same
**Symptomatic, suspect, probable or confirmed case with MODERATE symptoms**	Regardless of vaccination status	At least 10 days* from onset of symptoms	Same
**Symptomatic, suspect, probable or confirmed case with SEVERE and CRITICAL symptoms**	Regardless of vaccination status	At least 21 days* from onset of symptoms	Same
**Severely Immunocompromised******	Regardless of vaccination status	At least 21 days* from onset of symptoms with negative repeat RT-PCR	Same

*Isolation can be discontinued upon completion of the required days, provided that, they shall not develop fever for at least 24 hours without the use of any antipyretic medications and shall have improvement of respiratory symptoms. Except for immunocompromised individuals, repeat testing nor medical certification is not required for safe reintegration into the community. Time based isolation is sufficient provided the affected individual remains asymptomatic.
** Hospital IPCC, PHO coordinated with provincial HIPCC, and other sectors authorized by the IATF with strict industry standards on IPC shall be authorized to implement further shortening of quarantine and isolation protocols for their fully vaccinated workers with boosters who are close contacts, suspect, probable, and confirmed cases whether asymptomatic, mild, or moderate, based on the institution's individualized risk and needs assessment.
*** All asymptomatic close contacts should continue symptom monitoring for 14 days, strictly observe MPHS which includes wearing well-fitted masks, physical distancing, among others

****Includes (1) individuals receiving active chemotherapy for cancer; (2) Being within one year out from receiving a hematopoietic stem cell or solid organ transplant; (3) Untreated HIV infection with CD4 <200; (4) Primary Immunodeficiency; (5) Taking immunosuppressive medications (e.g., drugs to suppress rejection of transplanted organs or to treat rheumatologic conditions such as mycophenolate and rituximab); (6) Taking more than 20mg a day of prednisone for more than 14 days; (7) The degree of immunocompromise is determined by the health care provider, and preventive actions are adapted to each individual and situation.

ANNEX C: List of establishments identified under the principles of 3C's (Closed, Crowded, and Close Contact) strategy against COVID-19 wherein presentation of proof of full vaccination shall be required before entry:

1. Dine-in services in food preparation establishments such as kiosks, commissaries, restaurants, and eateries.
2. Film, music, and television production.
3. Fitness studios, gyms, and venues for non-contact exercise and sports.
4. Venues for all contact sports approved by the LGU where such games shall be held.
5. Venues for meetings, incentives, conferences, and exhibitions.
6. Permitted venues for social events such as parties, wedding receptions, engagement parties, wedding anniversaries, debut and birthday parties, family reunions, and bridal or baby showers.
7. Visitor or tourist attractions such as libraries, archives, museums, gallerties, exhibits, parks, plazas, public gardens, scenic viewpoints or overlooks, and the like.
8. Amusement parks and theme parks.
9. Recreational venues such as internet cafes, billiard halls, amusement arcades, bowling alleys, skating rinks, archery halls, swimming pools, and similar venues.
10. Cinemas and movie houses.
11. Personal care establishments such as barbershops, hair spas, hair salons, and nail spas, and those offering aesthetic/cosmetic services or procedures, make-up services, salons, spas, reflexology, and other similar procedures including home service options.
12. Venues with live voice or wind-instrument performers and audiences such as in karaoke bars, bars, clubs, concert halls, and theaters.
13. Funfairs/*peryas* and kid amusement industries such as playgrounds, playrooms, and kiddie rides.
14. In-person religious gatherings; gatherings for necrological services, wakes, inurnment, and funerals for those who died of causes other than COVID-19 and for the cremains of the COVID-19 deceased.

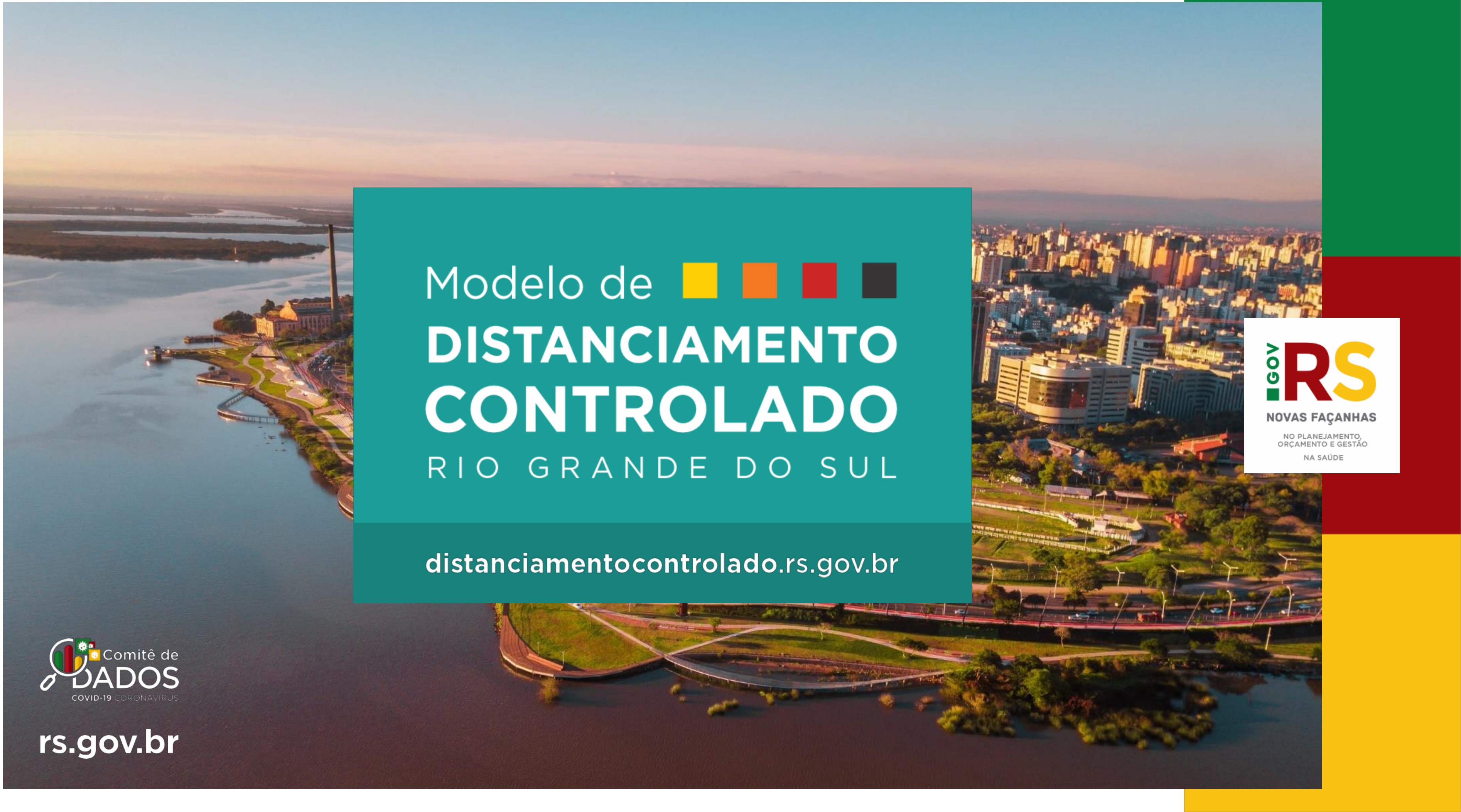
Modelo de
DISTANCIAMENTO
CONTROLADO
RIO GRANDE DO SUL
distanciamentocontrolado.rs.gov.br
GOV RS
NOVAS FAÇANHAS
NO PLANEJAMENTO, ORÇAMENTO E GESTÃO
NA SAÚDE
Comitê de
DADOS
COVID-19 CORONAVIRUS
rs.gov.br

Pilares e princípios

Materialização da estratégia

Estratégia mista, modulada e pactuada, para equilibrar prioridade à **VIDA** com retomada **ECONÔMICA**

Não é flexibilização aleatória
Não é abertura desordenada
Não é volta à normalidade

6 PASSOS

1. **Evitar o pior** – começar cedo. **FOCO NA VIDA**.
2. **Baseada em dados** – sistemas, monitoramento e projeções
3. **Sistema de alerta** – bandeiras de risco
4. **Ponderação econômica** – importância da atividade e nível de risco
5. **Protocolos** – restrição quando e onde é crítico (região e/ou atividade)
6. **Colaboração, diálogo e transparência** – aperfeiçoamento contínuo

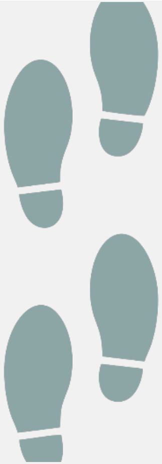

Método e prática

DISTANCIAMENTO CONTROLADO
RS
NOVAS FAÇANHAS

Método pioneiro

Modelo ***pioneiro*** *no mundo na regionalização do risco e dos protocolos*

Benchmarking para as estratégias de **sete** estados

REGIONALIZAÇÃO

Agrupamento das 30 regiões de saúde, com base nos hospitais de referência para leitos de UTI, totalizando **21 regiões Covid**

ALERTA DE RISCO

Alertas regionais por **bandeira**, a partir da **coleta de dados** e da **construção de índice de risco** com base em 7 indicadores de **propagação** doa doença e de 4 indicadores de **capacidade do sistema hospitalar**

PONDERAÇÃO SETORIAL

Criação de **índice setorial** para ponderar **importância da atividade econômica** no estado e **segurança das ocupações**, para calibrar as restrições

PROTOCOLOS COMPARTILHADOS

Rodadas de **consulta** e **diálogo** contínuo com entidades e empresas na construção de 11 **protocolos obrigatórios** e 3 protocolos **segmentados** por atividade e bandeira

COMUNICAÇÃO AMPLIADA

Comunicação direta com a sociedade, por diversos canais (**site** e e-mail exclusivos, Fale Conosco, telefone, chat e **SMS**)

Regionalização

Regionalização

- Existem **30 Regiões de Saúde e 7 Macrorregiões de Saúde** no RS.

- Para o acompanhamento dos indicadores, concatenaram-se algumas regiões de Saúde: **totalizando 21 regiões**.

- Critério de concatenação: as 30 regiões foram concatenadas de tal modo que, dentro de cada uma das novas 20 regiões, existam **hospitais de referência para leitos de UTI**.

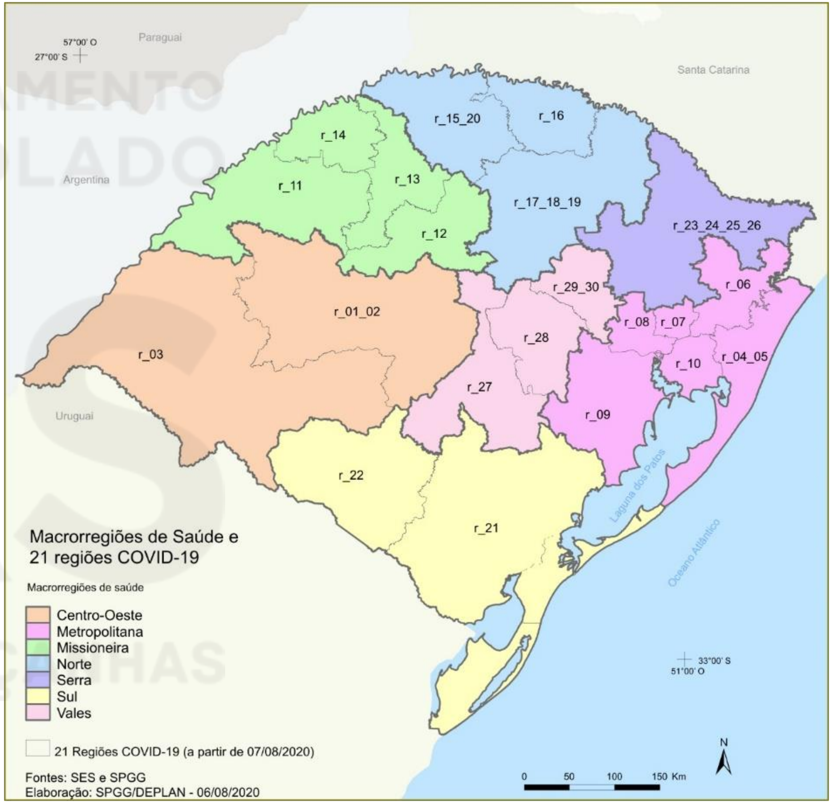

Alerta de Risco

Regionalização e Alerta de Risco

Dimensões de cálculo

- As medidas são dividas em dois grandes grupos: **Propagação da COVID-19** e **Capacidade de Atendimento** do sistema de saúde;
- Cada grupo de medidas possui peso 5/10 (**50%**) para a definição das bandeiras;
- No total, serão acompanhados **11 indicadores**.

Medidas e pesos atribuídos

		PESO DA MEDIDA	Nº DE INDICADORES
PROPAGAÇÃO	Velocidade do avanço	1,5	4
	Estágio da Evolução	1,0	1
	Incidência de novos casos sobre a população	2,5	2
	**SUBTOTAL**	5	7
CAPACIDADE DE ATENDIMENTO	Capacidade de atendimento	2,5	2
	Mudança da Capacidade de atendimento	2,5	2
	**SUBTOTAL**	5	4
	**TOTAL**	10	11

Regionalização e Alerta de Risco

Medidas e pesos a atribuídos

DISTANCIAMENTO CONTROLADO

PROPAGAÇÃO						
VELOCIDADE DO AVANÇO				ESTÁGIO DA EVOLUÇÃO NA REGIÃO	INCIDÊNCIA DE NOVOS CASOS SOBRE A POPULAÇÃO	
20 Regiões	7 Macrorregiões	7 Macrorregiões	7 Macrorregiões	20 Regiões	20 Regiões	20 Regiões
0,375	0,375	0,375	0,375	1	1,25	1,25
**Variação no nº de hospitalizações COVID**	**Variação no n° de internados por SRAG em UTI**	**Variação no n° de confirmados em leitos clínicos**	**Variação no n° de confirmados em UTI**	**Razão entre casos ativos e recuperados**	**Hospitalizações COVID / pop.**	**Projeção de óbitos**
Nº de hospitalizações confirmadas para COVID-19 registradas nos últimos 7 dias / (1 + Nº de hospitalizações confirmadas para COVID-19 registradas nos 7 dias anteriores)	Nº de internados por SRAG* em UTI no último dia / Nº de internados por SRAG há 7 dias atrás	Nº de Pacientes COVID-19 (Confirmados) em leitos clínicos no último dia / Nº de Pacientes COVID-19 (Confirmados) em leitos clinicos há 7 dias atrás	Nº de Pacientes COVID-19 (Confirmados) em leitos UTI no último dia / Nº de Pacientes COVID-19 (Confirmados) em leitos UTI há 7 dias atrás	Ativos na última semana / (1+Recuperados nos 50 dias anteriores ao início da semana)	Nº de hospitalizações confirmadas para COVID-19 registradas nos últimos 7 dias por 100.000 habitantes	Projeção de Óbitos com base nos óbitos dos últimos 7 dias e na variação de pacientes Covid-19 em UTI, para cada 100mil hab.

Regionalização e Alerta de Risco

Medidas e pesos a serem atribuídos

CAPACIDADE DE ATENDIMENTO			
CAPACIDADE DE ATENDIMENTO		MUDANÇA DA CAPACIDADE DE ATENDIMENTO	
7 Macrorregiões	Estado	7 Macrorregiões	Estado
1,25	1,25	1,25	1,25
**Leitos livres de UTI em relação a leitos ocupados por COVID-19 em UTI (Macrorregião)**<br><br>Leitos de UTI Livres / Leitos de UTI ocupados por pacientes COVID	**Leitos livres de UTI em relação a leitos ocupados por COVID-19 em UTI (Estado)**<br><br>Leitos de UTI Livres / Leitos de UTI ocupados por pacientes COVID	**Variação no n° de leitos de UTI disponíveis para atender COVID-19**<br><br>Nº de leitos de UTI disponíveis no último dia para atender COVID / Nº de leitos de UTI disponíveis 7 dias atrás para atender COVID	**Variação no n° de leitos de UTI disponíveis para atender COVID-19**<br><br>Nº de leitos de UTI disponíveis no último dia para atender COVID / Nº de leitos de UTI disponíveis 7 dias atrás para atender COVID

- **Cada indicador** receberá uma bandeira, conforme o valor final aferido e os **pontos de corte definidos**.
- A **Bandeira Final** é obtida por meio do **arredondamento da média ponderada das bandeiras dos indicadores** (conforme os pesos aplicados).
- Para fazer a média das bandeiras, atribuiu-se um valor à bandeira de cada indicador: **Amarela**=0; **Laranja**=1; **Vermelha**=2; **Preta**=3.

Regionalização e Alerta de Risco

AGOSTO	SETEMBRO			
**16ª semana** - vigência entre 25/08 a 31//08	**17ª semana** - vigência entre 01/09 a 07//09	**18ª semana** - vigência entre 08/09 a 14//09	**19ª semana** - vigência entre 15/09 a 21//09	**20ª semana** - vigência entre 22/09 a 28//09

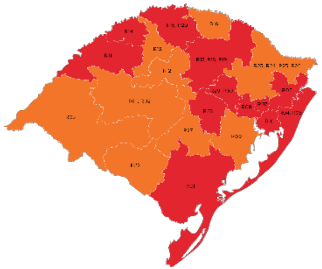
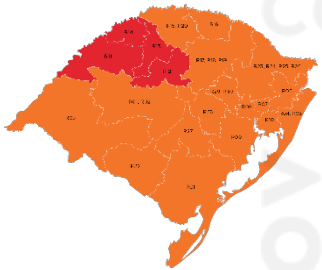
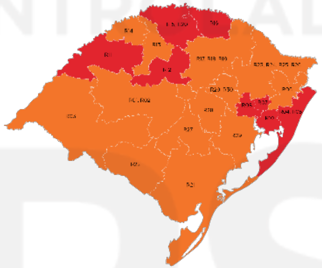
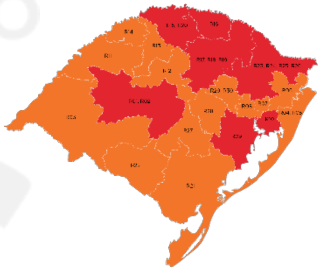
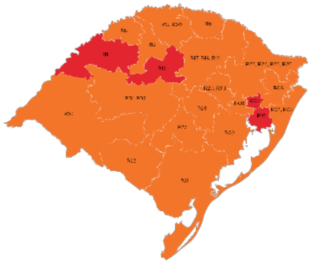

Modelo continuamente aperfeiçoado, em diálogo com regiões e municípios

Sistema de bandeiras

- cálculo **semanal** das bandeiras, com divulgação nas sextas-feiras
- prazo de 36 horas para **recursos** das associações regionais e municípios
- análise dos recursos e **decisão do Gabinete de Crise** nas segundas-feiras
- **vigência** a partir da 00h de terça-feira
- exceções à bandeira vermelha: municípios com 0 óbitos e 0 hospitalizações há 14 dias
- **gestão compartilhada** com 18 regiões, por adesão de 2/3 dos municípios

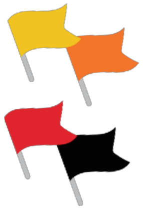

Ponderação Setorial

Ponderação Setorial

- Segmentação em **12 grupos**
- Protocolos para **50 atividades** (CNAE 2 dígitos)
- Informações complementares para **níveis de atividades mais desagregados**:
  - Indicador de Segurança;
  - Emprego;
  - % de Empresas no Simples Nacional para subsetores;
  - Tamanho Médio das Empresas.

Índice Setorial para Distanciamento Controlado do RS

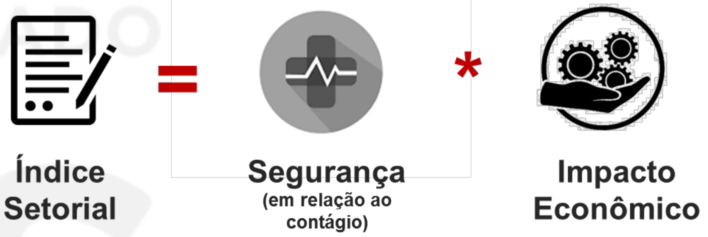

- **Indicador de Segurança:** construído para cada Atividade, a partir do risco calculado para cada uma de suas ocupações (COPPE/UFRJ)
- **Indicador de Impacto Econômico:** a partir do Valor Adicionado Bruto de cada atividade (DEE/SEPLAG)

Ponderação Setorial

- **Indicador de Segurança** construído para cada atividade, a partir de suas **ocupações**
- Cada ocupação está atrelada a um **risco de contágio**:

METODOLOGIA: Baseada na metodologia da COPPE/UFRJ

RISCO DAS OCUPAÇÕES: a partir de **3 perguntas da O*NET** (Departamento Americano de Trabalho e Emprego*)

1) Com qual frequência esse trabalho requer **exposição a doenças e infecções**?

2) O quanto esse trabalho exige de **contato com outros**?

3) Em qual extensão esse trabalho requer a **proximidade física** com outras pessoas?

TRADUÇÃO: da SOC (classificação das ocupações nos EUA) para **Classificação Brasileira de Ocupações (CBO).**

Risco por Ocupação

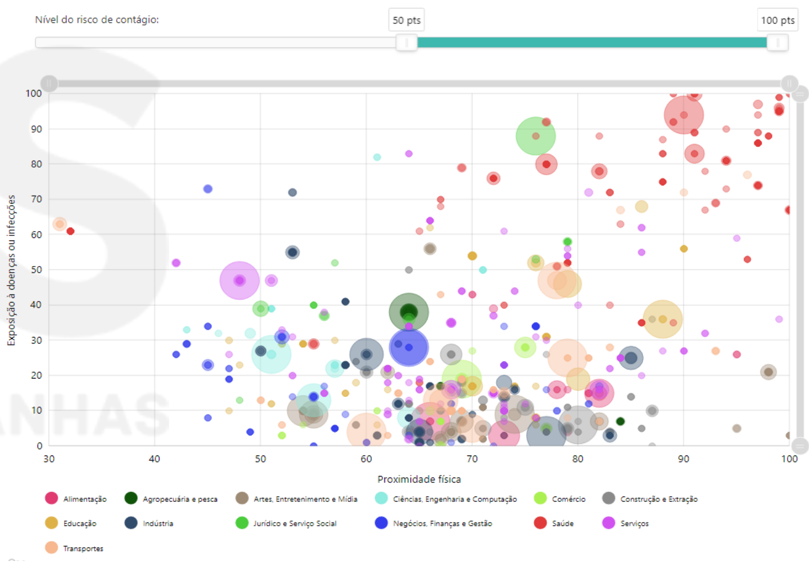

*Fonte: https://impactocovid.com.br/ , https://www.onetonline.org/. *U.S. Department of Labor/Employment and Training Administration)*

Ponderação Setorial

Ponderação:
- **70%** para Segurança
- **30%** para Atividade Eco.

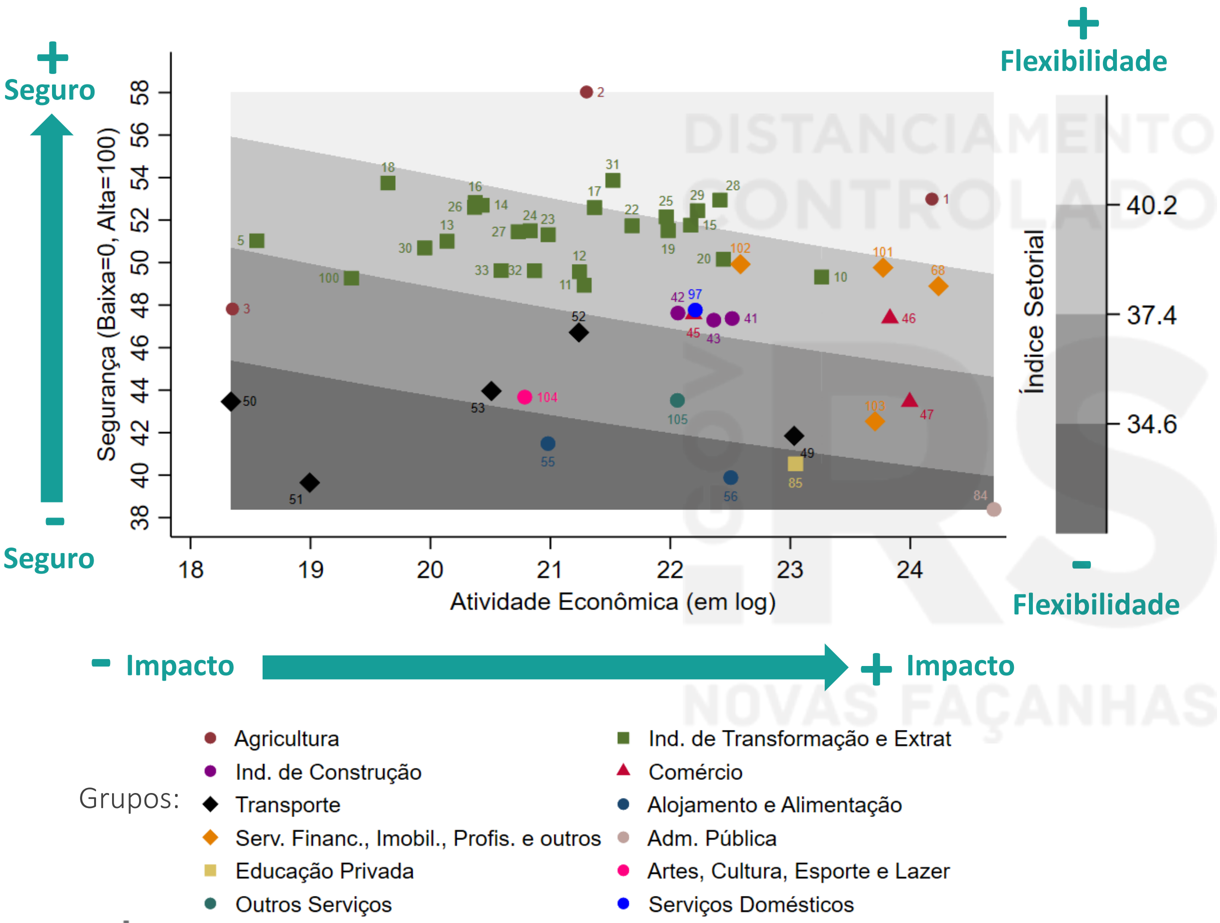

CNAE	Atividade	CNAE	Atividade
1	Agricultura e Pecuária	32	Produtos Diversos
2	Produção Florestal	33	Manut. e Reparação
3	Pesca e Aqüicultura	41	Construção de Edifícios
5	Ext. de Carvão Mineral	42	Obras de Infraestrutura
10	Alimentos	43	Serviços de Construção
11	Bebidas	45	Comércio de Veículos
12	Fumo	46	Comércio Atacadista
13	Têxteis	47	Comércio Varejista
14	Vestuário	49	Transporte Terrestre
15	Couros e Calçados	50	Transporte aquaviário
16	Madeira	51	Transporte aéreo
17	Papel e Celulose	52	Armaz. de Transporte
18	Impressão e Reprod.	53	Correios
19	Derivados Petróleo	55	Alojamento
20	Químicos	56	Alimentação
22	Borracha e Plástico	68	Serv. Imobiliário
23	Minerais não metálicos	84	Adm Pública
24	Metalurgia	85	Educação
25	Produtos de Metal	97	Serv. Domésticos
26	Equip. Informática	100*	Extr. de Petróleo e Minerais
27	Materiais Elétricos	101*	Serv. Financeiros
28	Máquinas e Equip.	102*	Serv. Profis., Cientif. e Técnicas
29	Veículos Automotores	103*	Serv. Admin. e Auxiliares
30	Outros Equipamentos	104*	Artes, Cult., Esportes e Lazer
31	Móveis	105*	Outros Serv.

Atividades Essenciais (Não incluídas na análise Seguranca x Ativ. Econ.)

21	Farmoq. e Farmacêuticos	61	Telecomunicações
35	Eletric., Gás e Outras Util.	62	Serviços de TI
36	Capt., Trat. e Distr. Água	63	Serviços de Informação
37	Esgoto e Ativ. Relac.	80	Ativ.Vigilância, Segurança e Investig.
38	Coleta e Tratam. Resíduos	81	Serv. para Edifícios e Paisagismo
39	Descontam. Resíduos	86	Atenção à Saúde Humana
58	Edição	87	Atenção à Saúde com Assist.Social
59	Atividades Cinematográficas	88	Assistência Social sem Alojam.
60	Atividades de Rádio e de TV		

Nota: (*) Representam agregações de atividades 2 dígitos.

DISTANCIAMENTO CONTROLADO
GOV RS
NOVAS FAÇANHAS

Protocolos compartilhados

Protocolos compartilhados

Protocolos semanalmente aprimorados, em diálogo com prefeituras, entidades e empresas

Construção intersetorial

- *benchamarking* **nacionais e internacionais**.
- duas rodadas de **participação da sociedade** e contínuo canal de comunicação aberto discussão e **construção intersetorial** pelas equipes técnicas do Comitê de Dados e da Secretaria Estadual de Saúde (SES)
- **deliberação** do Gabinete de Crise

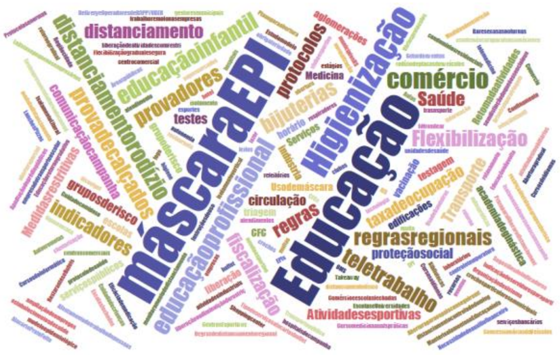

Protocolos por atividade e risco

- Classificação em atividades **essenciais** e **não** essenciais
- Identificação de atividades com tratamento específico (ex.: ILPI)
- **136 atividades** com protocolos **obrigatórios** e **específicos**

Comércio varejista de rua – não essencial

Operação: 50% trabalhadores
Atendimento: Presencial restrito / Telentrega / Pegue e Leve / Drive-thru

Operação: 50% trabalhadores
Atendimento: Presencial restrito / Telentrega / Pegue e Leve / Drive-thru

Operação: 25% trabalhadores
Atendimento: Presencial restrito / Telentrega / Pegue e Leve / Drive-thru

Operação: Fechado
Atendimento: Sem atendimento ao público

Protocolos compartilhados

https://tinyurl.com/ybjsyvjj

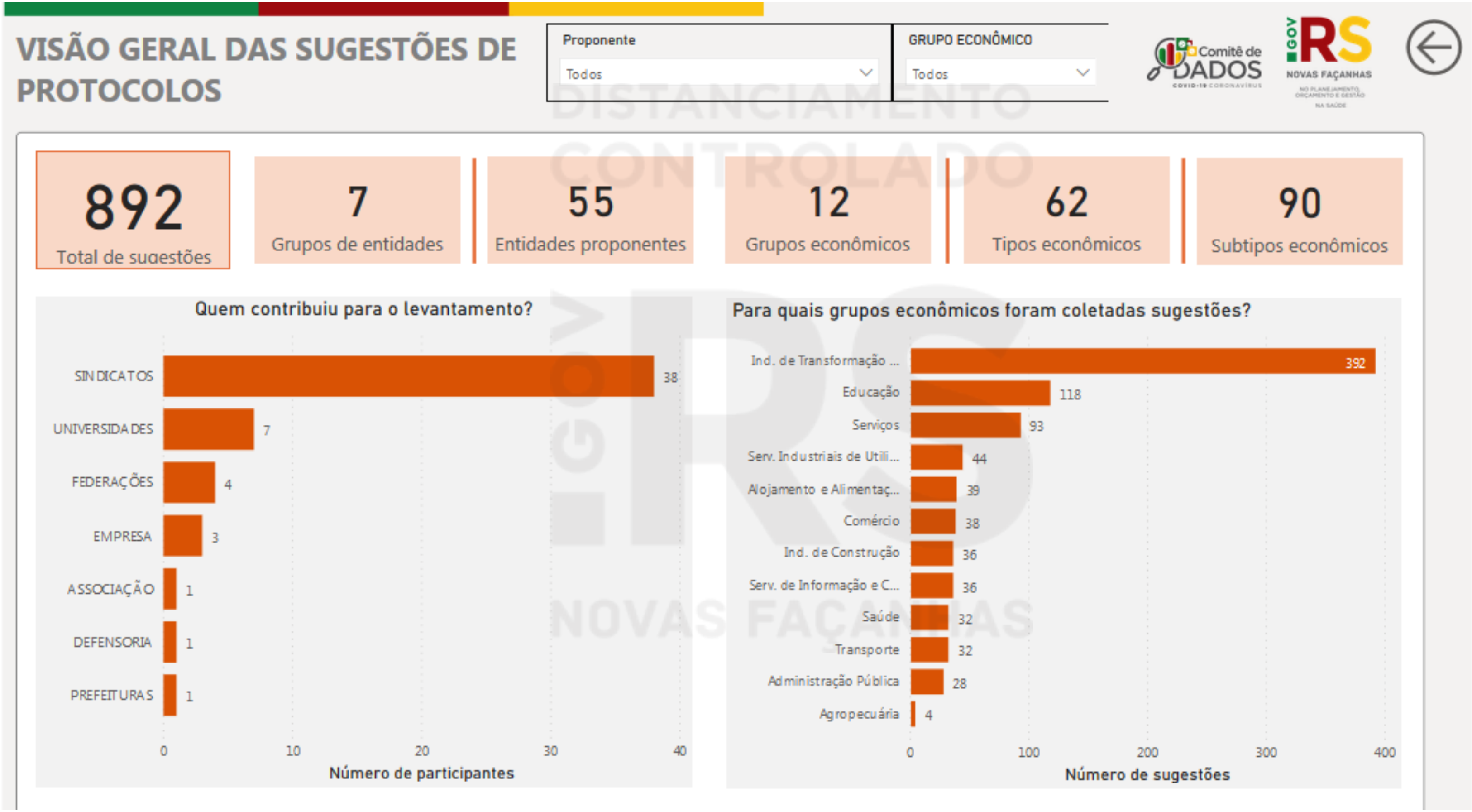

Protocolos compartilhados

https://tinyurl.com/ybjsyvjj

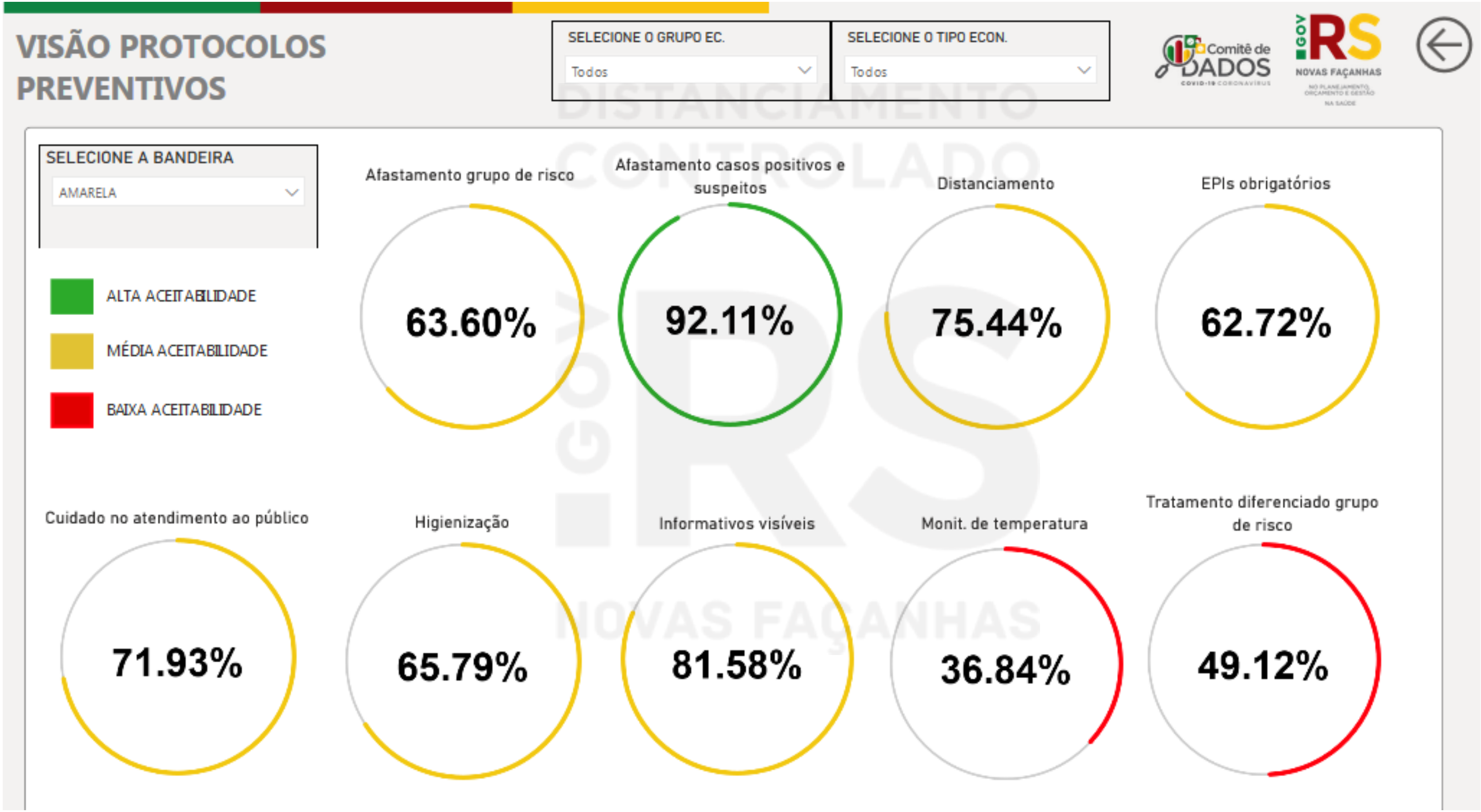

Protocolos compartilhados

https://tinyurl.com/ybjsyvjj

VISÃO POR PROPONENTE

PROPONENTE: FECOMÉRCIO

Proponente		Modo de operação	Horário	Teto de ocupação	Afast. Grupo de Risco	Afast. Positivos e Suspeitos	Cuidado no atend. ao público	Distanciamento	EPIs obrigat.	Higieniz.	Informativo visível	Uso de máscara	Monitor de temp.	Tratamento dif. de grupo de risco
FECOMÉRCIO														
Academia de ginástica														
AMARELA		Presencial com restrições	Mantido	1	✓	✓	✓	✓	✗	✓	✓	✓	✗	✗
LARANJA		Presencial com restrições	Mantido	0,7	✓	✓	✓	✓	✗	✓	✓	✓	✗	✗
VERMELHA		Presencial com restrições	Redução	0,5	✓	✓	✓	✓	✓	✓	✓	✓	✗	✓
PRETA		Presencial com restrições	Redução	0,3	✓	✓	✓	✓	✓	✓	✓	✓	✓	✓
Agência de turismo, passeios e excursões														
AMARELA		Presencial com restrições	Mantido	1	✓	✓	✓	✓	✗	✓	✓	✓	✗	✗
LARANJA		Presencial com restrições	Mantido	0,7	✓	✓	✓	✓	✗	✓	✓	✓	✗	✗
VERMELHA		Presencial com restrições	Redução	0,5	✓	✓	✓	✓	✓	✓	✓	✓	✗	✓
PRETA		Presencial com restrições	Redução	0,3	✓	✓	✓	✓	✓	✓	✓	✓	✓	✓
Armazemento, carga e														
AMARELA		Presencial com restrições	Mantido	1	✓	✓	✓	✓	✗	✓	✓	✓	✗	✗
LARANJA		Presencial com restrições	Mantido	1	✓	✓	✓	✓	✗	✓	✓	✓	✗	✗
VERMELHA		Presencial com restrições	Mantido	1	✓	✓	✓	✓	✓	✓	✓	✓	✗	✓
PRETA		Presencial com restrições	Mantido	0,5	✓	✓	✓	✓	✓	✓	✓	✓	✓	✓
Assistência Social														
AMARELA		Presencial com restrições	Mantido	1	✓	✓	✓	✓	✗	✓	✓	✓	✗	✗
LARANJA		Presencial com restrições	Mantido	1	✓	✓	✓	✓	✗	✓	✓	✓	✗	✗
VERMELHA		Presencial com restrições	Mantido	1	✓	✓	✓	✓	✓	✓	✓	✓	✗	✓
PRETA		Presencial com restrições	Mantido	0,5	✓	✓	✓	✓	✓	✓	✓	✓	✓	✓
Assistência Social sem Alojamento														
AMARELA		Presencial com restrições	Mantido	1	✓	✓	✓	✓	✗	✓	✓	✓	✗	✗
LARANJA		Presencial com restrições	Mantido	1	✓	✓	✓	✓	✗	✓	✓	✓	✗	✗
VERMELHA		Presencial com restrições	Mantido	1	✓	✓	✓	✓	✓	✓	✓	✓	✗	✓
PRETA		Presencial com restrições	Mantido	0,5	✓	✓	✓	✓	✓	✓	✓	✓	✓	✓
[illegible]	[illegible]	[illegible]	[illegible]	[illegible]	[illegible]	[illegible]	[illegible]	[illegible]	[illegible]	[illegible]	[illegible]	[illegible]	[illegible]	[illegible]

Protocolos compartilhados

CRITÉRIOS DE FUNCIONAMENTO (variáveis por bandeira)

- **Teto de operação**
- **Modo de operação**
- Horário de Funcionamento (municípios)

PROTOCOLOS DE PREVENÇÃO OBRIGATÓRIOS (todas as bandeiras)

- **Máscara** (público e trabalhadores)
- **Distanciamento entre pessoas**
- **Teto de ocupação**
- **Higienização** (ambiente, trabalhadores e público)
- **Informativo visível**
- **EPIs obrigatórios**
- **Proteção de grupos de risco no trabalho**
- **Afastamento de casos positivos ou suspeitos**
- **Cuidados no atendimento ao público**
- **Atendimento diferenciado para grupo de riscos**
- **Restrição específica à atividade**

PROTOCOLOS RECOMENDADOS (não obrigatórios, variáveis por bandeiras e atividades)

- **Monitoramento de temperatura**
- **Testagem dos colaboradores**

DISTANCIAMENTO CONTROLADO
NOVAS FAÇANHAS

DISTANCIAMENTO CONTROLADO
.GOV RS
NOVAS FAÇANHAS

Comunicação ampliada

Comunicação ampliada

Site do Distanciamento Controlado

- **mapa das bandeiras** por região
- **protocolos** gerais obrigatórios e protocolos específicos por atividade
- ferramenta de pesquisa por **município** e **setor** econômico
- **vídeos** explicativos
- **metodologia**, **bases de dados** completa e **normas**
- telefone, chat e **Fale Conosco**

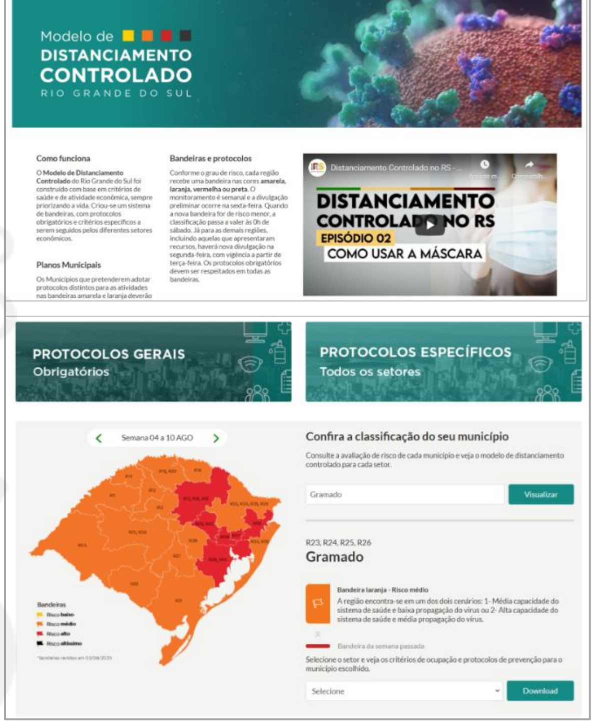

distanciamentocontrolado.rs.gov.br

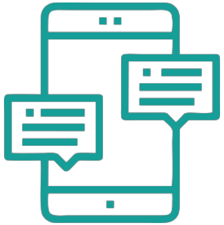

Alertas SMS

- parceria com a Defesa Civil e SECOM
- envios **semanais**, a cada mudança de bandeira
- mensagens com ***nudges*** para promover a mudança de comportamento
- *mais* de **5,7 milhões** de SMS enviados (até 21/set)

Resultados

Adesão dos municípios

O que pensam os prefeitos

A consulta feita aos prefeitos pela Famurs mostrou que a maioria absoluta apoia o modelo de distanciamento controlado: 56,06% concordam com o plano, mas acham que poderia melhorar. Para 33,08%, pode continuar como está. A rejeição se limita a 10,86% dos 396 gestores que responderam ao questionário.

A proposta de mudança no modelo, com maior autonomia aos municípios, divide os prefeitos: 20,2% acham que cada município deve decidir sobre seu território, 18,43% acham que a decisão deve ser das regiões, 27,02% discordam da mudança por entender que o governo apenas transfere responsabilidade aos gestores municipais e 34% acreditam que a decisão deve ser tomada em conjunto pelo governo estadual e pelas prefeituras.

A pesquisa será detalhada hoje, a partir das 9h, em reunião das associações de municípios com o secretário de articulação com as prefeituras, Agostinho Meirelles.

Fonte: Oliveira, Rosanne. GZH. Atualização: 04/08/2020.

90%
CONCORDAM
com o modelo

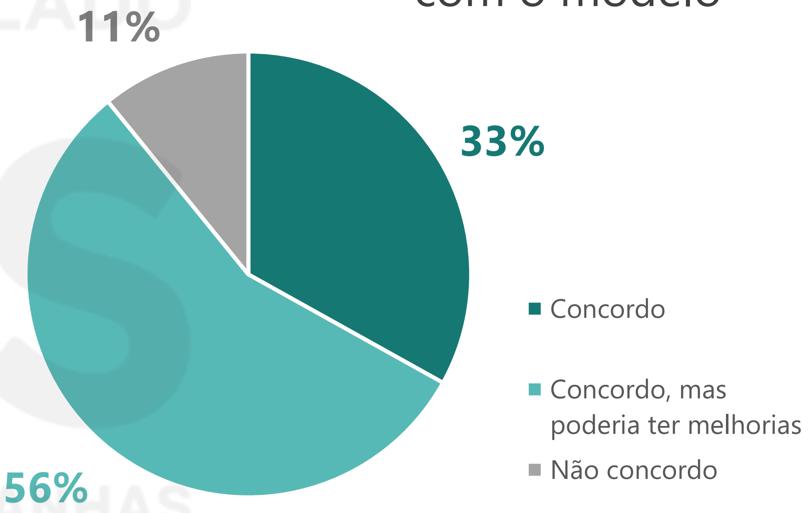

Fonte: FAMURS (2020). Atualização: 04/08/2020.

Proteção à vida

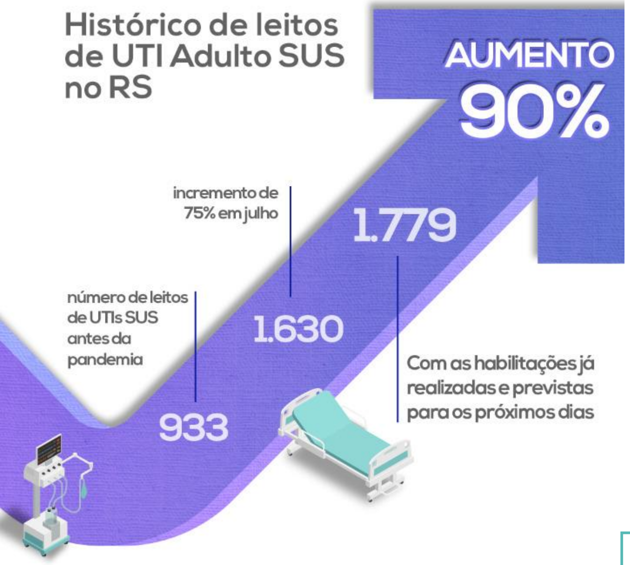

Fonte: SES (2020). Atualização: 27/07/2020.

tempo necessário para
MAIOR CRESCIMENTO
de **leitos no SUS**
na história do estado

Segunda MAIOR taxa de leitos de UTI
por 10 mil habitantes

Distribuição dos leitos de UTI convencionais no SUS, por estado (não inclui para Covid porque são leitos temporários)

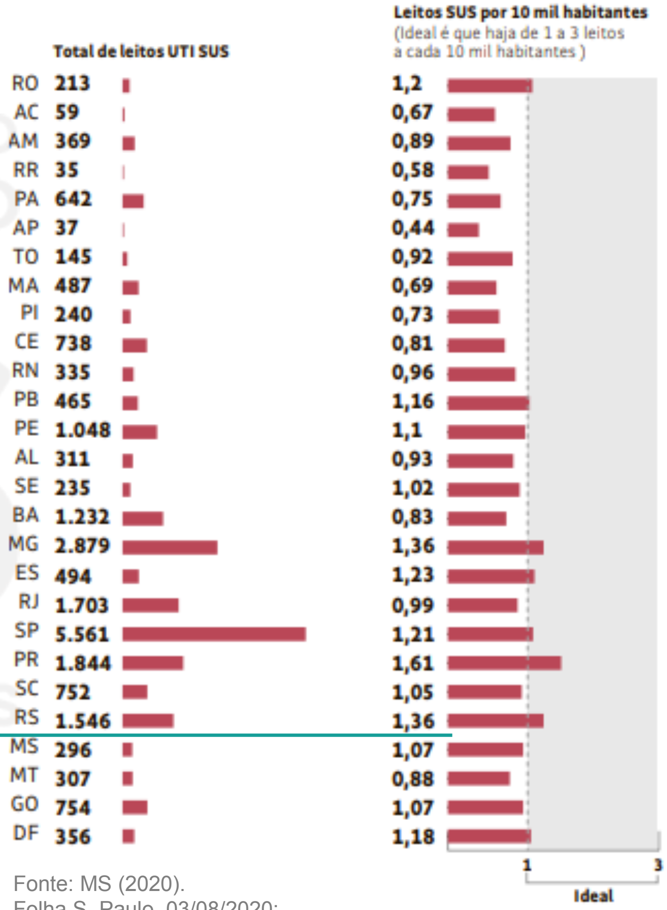

Fonte: MS (2020).
Folha S. Paulo, 03/08/2020:
shorturl.at/quEPX

Proteção à vida

ZERO óbitos
de Covid-19
por falta de leito

Quarta MENOR taxa de óbitos
por 100 mil habitantes

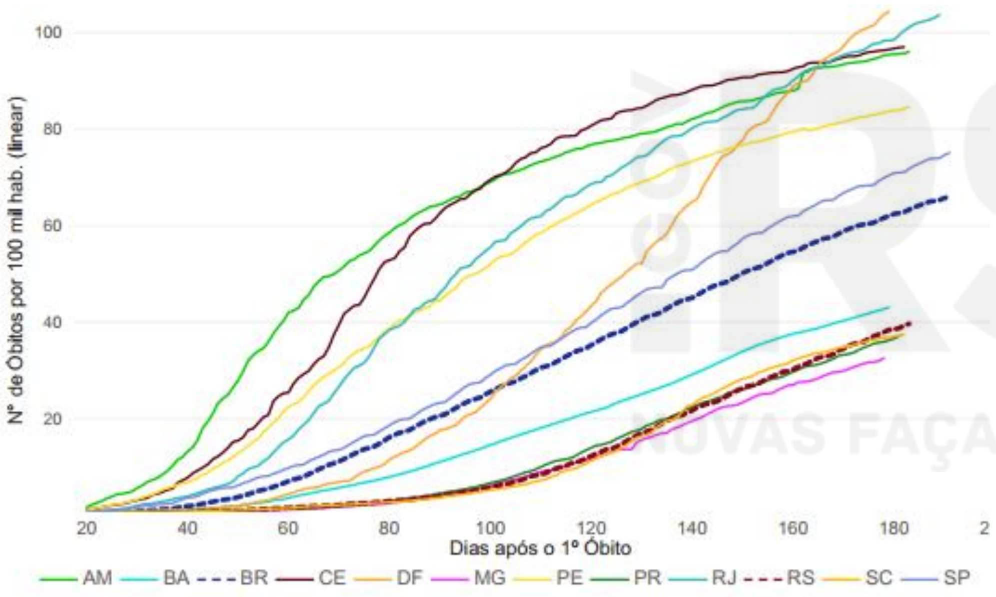

Fonte: MS (2020) e IBGE (2020). Atualização: 24/09/2020.

País/Estado	População	Nº Casos Acumulados	Casos por 100 mil hab.	Nº Óbitos Acumulados	Taxa de Mortalidade por 100 mil hab.
MG	21.168.791	276.314	1.305	6.897	32,58
PR	11.433.957	168.923	1.477	4.244	37,12
SC	7.164.788	208.900	2.916	2.686	37,49
RS	11.377.239	179.436	1.577	4.515	39,68
BA	14.873.064	299.415	2.013	6.408	43,08
MS	2.778.986	65.611	2.361	1.204	43,33
MA	7.075.181	169.690	2.398	3.683	52,06
TO	1.572.866	64.787	4.119	890	56,58
GO	7.018.354	192.157	2.738	4.265	60,77
AL	3.337.357	85.559	2.564	2.034	60,95
PI	3.273.227	92.030	2.812	2.061	62,97
BR	210.147.125	4.624.885	2.201	138.977	66,13
RN	3.506.853	67.761	1.932	2.356	67,18
PB	4.018.127	118.048	2.938	2.741	68,22
AC	881.935	27.397	3.106	652	73,93
RO	1.777.225	64.306	3.618	1.320	74,27
SP	45.919.049	951.973	2.073	34.492	75,11
PA	8.602.865	223.021	2.592	6.489	75,43
AP	845.731	47.364	5.600	697	82,41
PE	9.557.071	143.165	1.498	8.085	84,60
ES	4.018.650	126.606	3.150	3.452	85,90
SE	2.298.696	76.353	3.322	1.999	86,96
MT	3.484.466	116.700	3.349	3.287	94,33
AM	4.144.597	133.413	3.219	3.984	96,13
CE	9.132.078	236.100	2.585	8.861	97,03
RR	605.761	48.919	8.076	616	101,69
RJ	17.264.943	254.885	1.476	17.911	103,74
DF	3.015.268	186.052	6.170	3.148	104,40

Fonte: SES e MS (2020). Atualização: 24/09/2020.

Proteção à vida

Segunda MENOR relação entre total de óbitos
por 100 mil habitantes
com idade **acima de 50 anos**

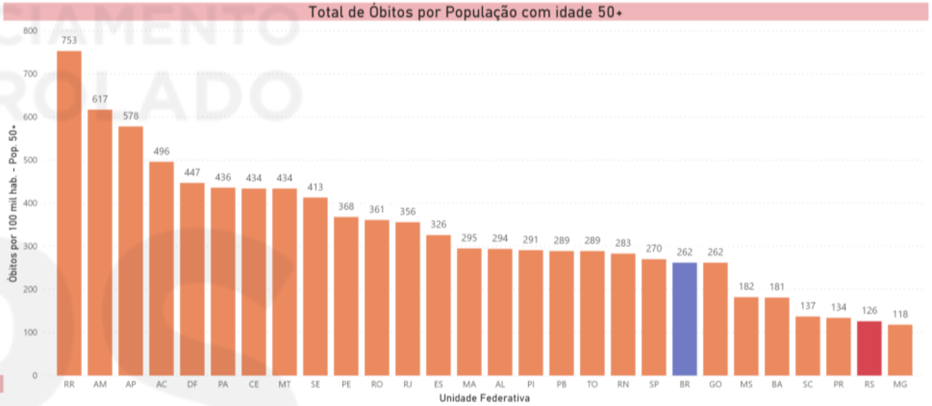

Quarta MENOR
taxa de mortalidade
por 100 mil habitantes

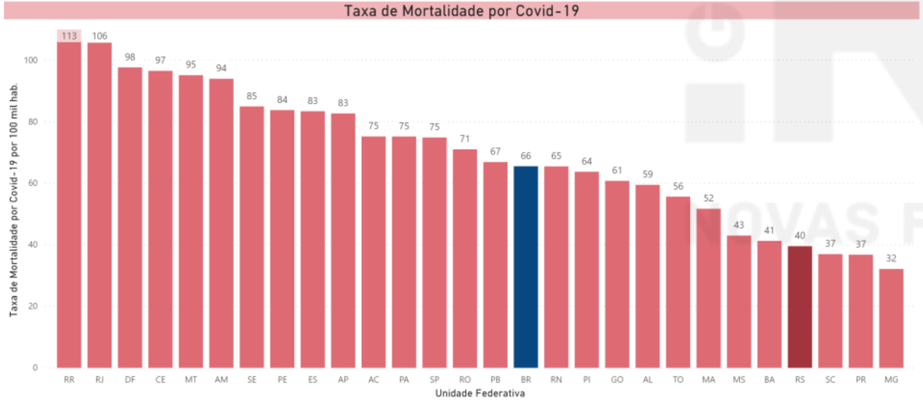

Fonte: Ministério da Saúde (Atualizado com dados até 23 de Setembro de 2020)
IBGE (Projeções Populacionais - Revisão 2018)

Proteção à vida

MENORES TAXAS DE LETALIDADE APARENTE
no cenário nacional e no cenário internacional

Casos Confirmados e Óbitos por 100 mil habitantes por População

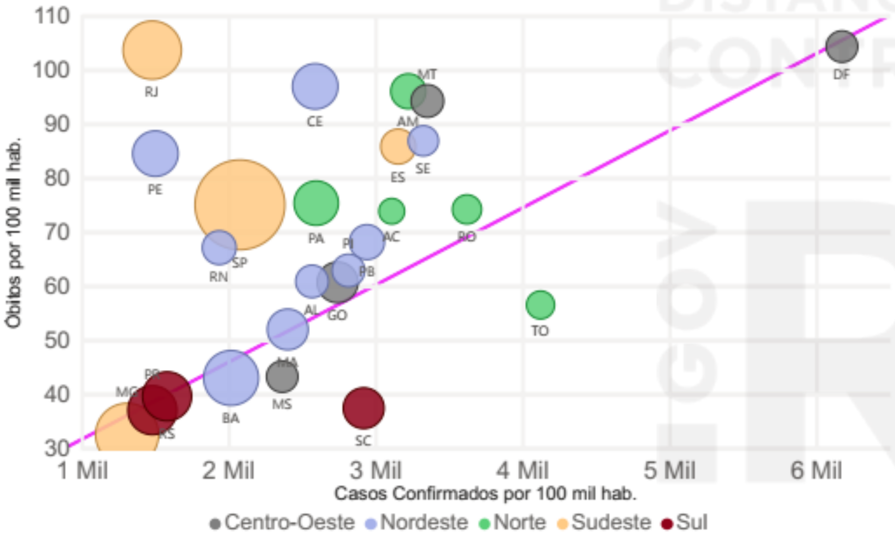

O tamanho do círculo representa a população do estado. Quanto maior o círculo, maior a população.

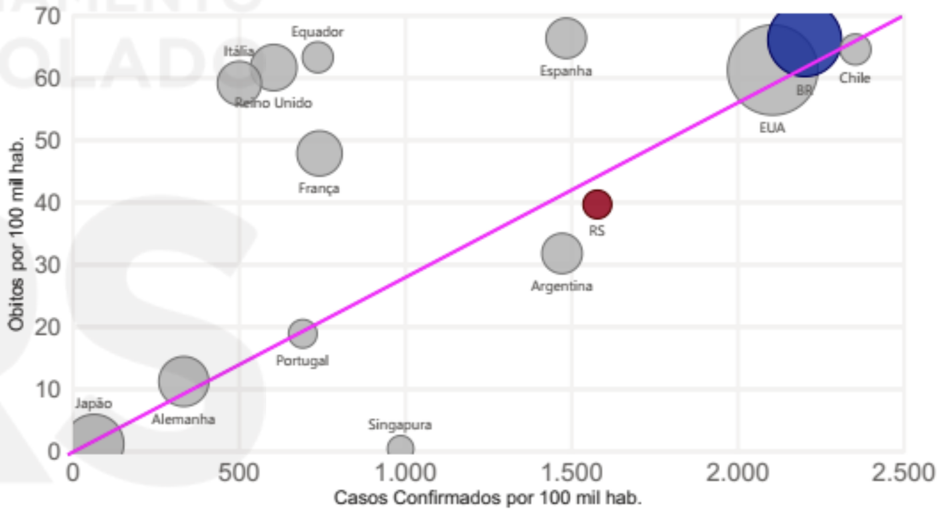

O tamanho do círculo representa a população do estado. Quanto maior o círculo, maior a população.

Fonte: Ministério da Saúde (BRASIL, 2020) e Instituto Brasileiro de Geografia e Estatística (2020) (População estimada em julho/2019). Atualização: 24/09/2020.

O gráfico apresenta três variáveis: o número de casos e óbitos em relação à população e o tamanho da população de cada região. A linha rosa indica uma proporção fixa entre óbitos e número de casos. Estados acima da linha apresentam maior taxa de letalidade aparente – a qual decorre, em parte, da realização de testes em uma parcela menor da população.

Proteção à vida

MENORES TAXAS DE MORTALIDADE
no cenário nacional

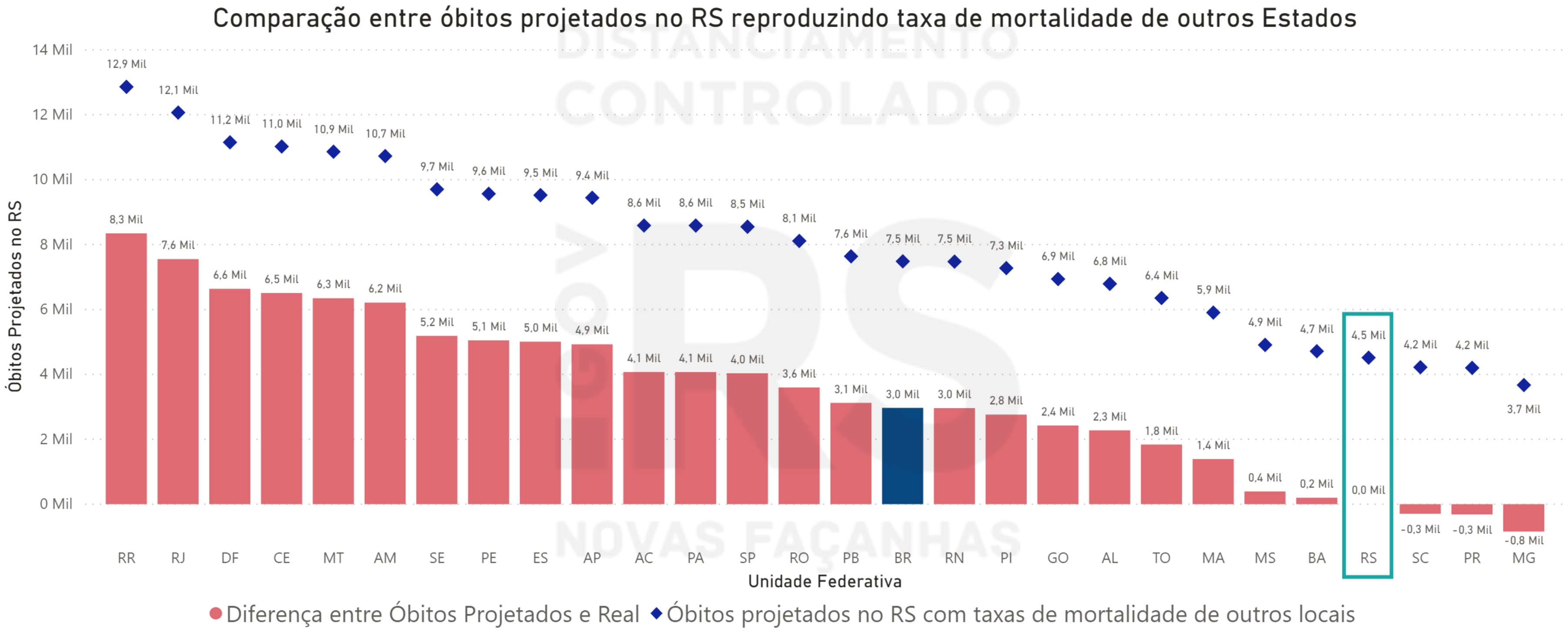

* O exercício não considera as diferentes fases da pandemia que cada estado enfrenta atualmente.

Fonte: MS (2020) e IBGE (2020). Atualização: 24/09/2020.

Proteção à vida

MENOR EXCESSO PROPORCIONAL DE ÓBITOS
no cenário nacional

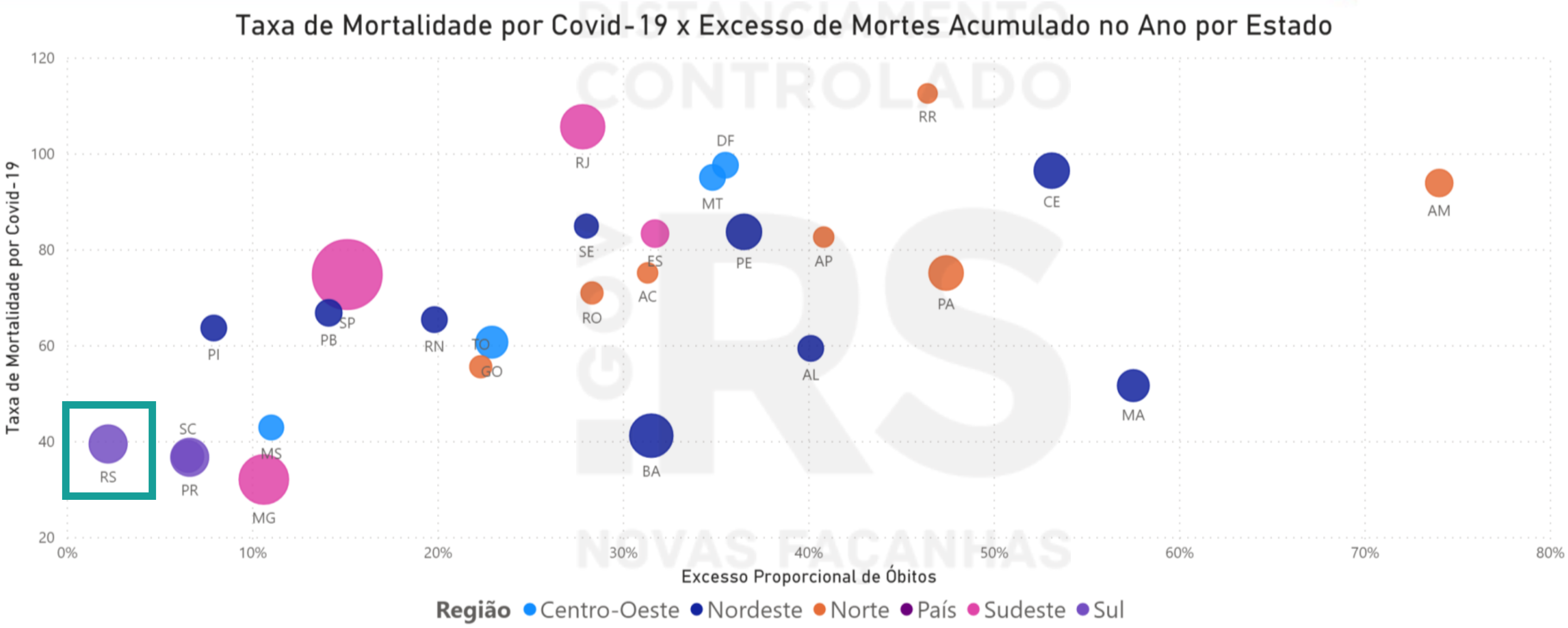

* O **Tamanho do círculo** representa a **População** de cada Unidade Federativa.

Fonte: Ministério da Saúde (Atualizado com dados até 23 de Setembro de 2020)
IBGE (Projeções Populacionais - Revisão 2018)
Conass (excesso de mortes calculado até 15/08)

Retomada econômica

MENOR QUEDA de arrecadação
no Sul e Sudeste

UF	Arrecadação, em R$ bi 1º Sem. 2019**	Arrecadação, em R$ bi 1º Sem. 2020	Variação 1º Sem. 2020
CE	7,6	6,3	-16,5%
RN	2,4	2,0	-15,1%
SE*	1,4	1,2	-14,3%
PR*	13,0	11,6	-10,5%
AP*	0,3	0,4	-9,0%
RJ*	18,4	16,8	-8,7%
PE	9,5	8,8	-7,2%
SP	95,7	89,1	-6,8%
MG	31,1	29,0	-6,8%
GO*	7,6	7,1	-6,8%
ES*	4,4	4,1	-6,3%
SC*	9,1	8,5	-6,1%
RR*	0,5	0,4	-6,0%
BA	12,7	11,9	-5,8%
**RS***	**14,1**	**13,4**	**-5,7%**

*Repasse a municípios já está deduzido na arrecadação.
**Arrecadação de 2019 atualizada pelo IPCA de 07/2019 a 06/2020 |
Fonte: Governos Estaduais Fonte: Folha de S. Paulo, 03/08/2020: shorturl.at/twxT1.

RÁPIDA RECUPERAÇÃO
no **comércio** e na **indústria**

Índice do volume da Indústria e do Comércio do RS - 2020

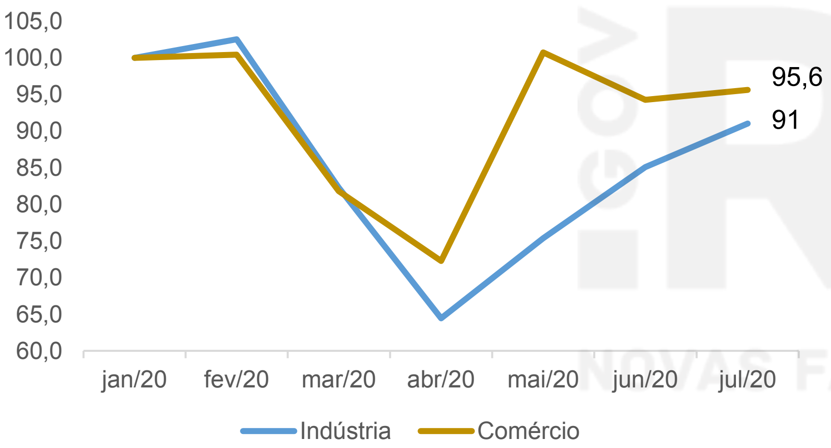

Fonte: IBGE
Nota: Média 2019 = 100

Fonte: Comitê de Dados - RS, 18/09/2020.

Retomada econômica

COMÉRCIO VAREJISTA e INDÚSTRIA DE TRANSFORMAÇÃO em recuperação

Índice de Volume de Vendas do Comércio Varejista Ampliado - 2020
(base fixa com ajuste sazonal)

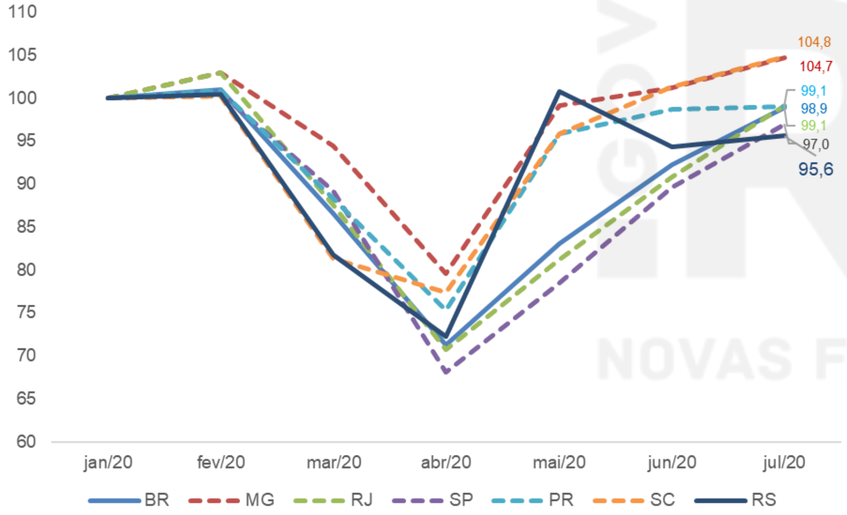

Índice de Produção Industrial por UF - 2020
(base fixa com ajuste sazonal)

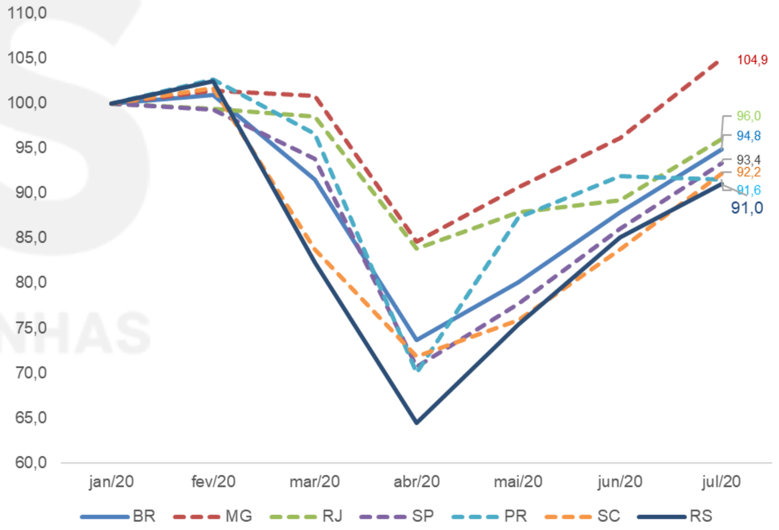

Fonte: IBGE. Comitê de Dados - RS, 18/09/2020. Nota: jan/20 = 100.

Retomada econômica

CRESCIMENTO nas **vendas**
em relação a 2019

EVOLUÇÃO DAS VENDAS POR ATIVIDADE
VARIAÇÃO DA VENDA MÉDIA DIÁRIA NO PERÍODO FRENTE A 2019 [1]

Acumulado
16/3/20 a 18/9/20

-5%	R$ 852,9 milhões
3%	R$ 529,7 milhões
-8%	R$ 463,3 milhões

Indústria
Atacado
Varejo
% Variação do período de 2020 em relação ao de 2019

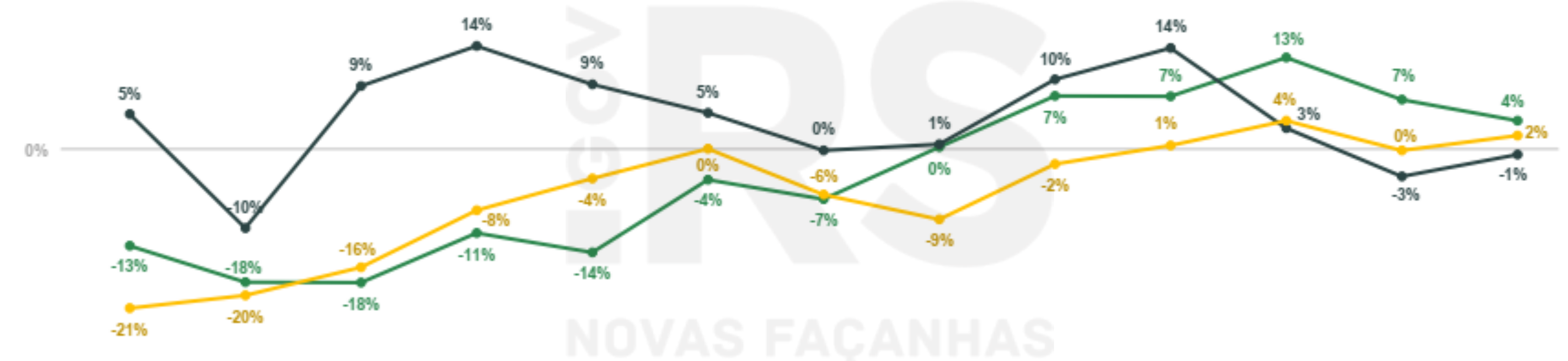

Quinzena	Q 1 * 16/3 a 3/4	Q 2 4/4 a 17/4	Q 3 18/4 a 1/5	Q 4 2/5 a 15/5	Q 5 16/5 a 29/5	Q 6 30/5 a 12/6	Q 7 13/6 a 26/6
Venda Média Diária	R$ 823,8 ml	R$ 679,2 ml	R$ 705,7 ml	R$ 728,2 ml	R$ 762,1 ml	R$ 777,8 ml	R$ 836,7 ml
	R$ 590,8 ml	R$ 612,3 ml	R$ 540,5 ml	R$ 553,6 ml	R$ 533,7 ml	R$ 495,8 ml	R$ 513,7 ml
	R$ 409,5 ml	R$ 414,6 ml	R$ 410,3 ml	R$ 467,5 ml	R$ 449,1 ml	R$ 506,5 ml	R$ 449,1 ml

Quinzena	Q 8 27/6 a 10/7	Q 9 11/7 a 24/7	Q 10 25/7 a 7/8	Q 11 8 a 21/8	Q 12 22/8 a 4/9	Q 13 5 a 18/9
Venda Média Diária	R$ 788,7 ml	R$ 901,2 ml	R$ 991,7 ml	R$ 1,0 bl	R$ 1,1 bl	R$ 962,4 ml
	R$ 478,6 ml	R$ 552,8 ml	R$ 570,9 ml	R$ 548,4 ml	R$ 582,5 ml	R$ 539,9 ml
	R$ 460,4 ml	R$ 456,6 ml	R$ 523,3 ml	R$ 503,5 ml	R$ 537,6 ml	R$ 506,5 ml

 Fonte: Nota Fiscal Eletrônica (NF-e) e a Nota Fiscal de Consumidor Eletrônica (NFC-e). SEFAZ (2020). Atualização: 24/09/2020.

Retomada econômica

QUEDA DE 50% no requerimentos de **seguro-desemprego** de maio a julho

Nº de requerentes de seguro-desemprego (jan-jul 2020)

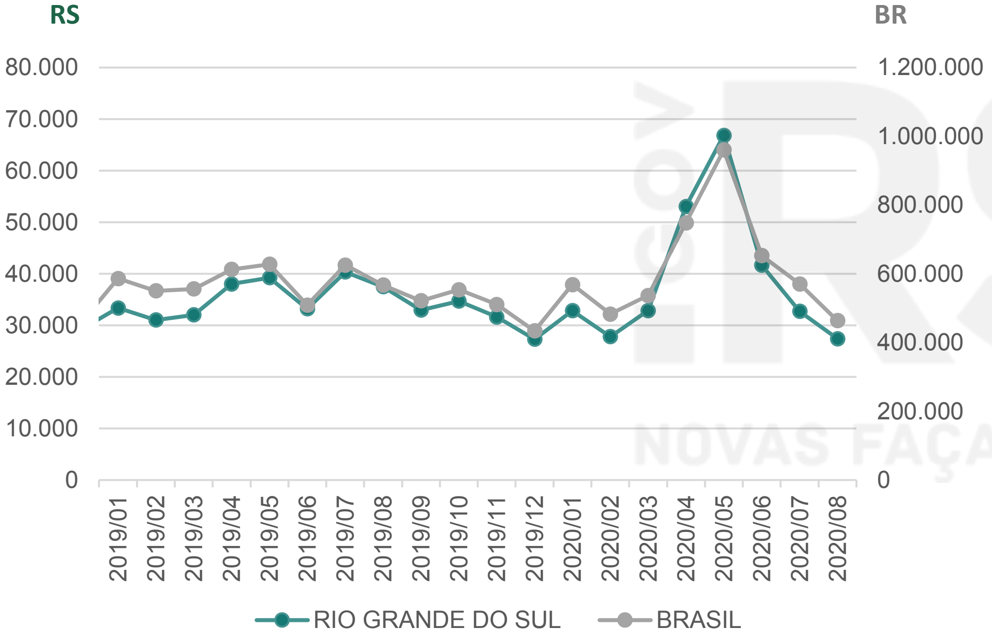

Redução superior à nacional

Var. % do Número de Requerentes

	Ago 20 / Jul 20	Ago 20 /Ago 19
BR	-18,7%	-18,2%
AC	-5,4%	-4,7%
AL	-22,5%	-30,6%
AP	-8,8%	-21,9%
AM	-20,8%	-15,2%
BA	-25,0%	-21,4%
CE	-18,9%	-34,2%
DF	-20,4%	-12,5%
ES	-24,7%	-19,3%
GO	-8,3%	-18,3%
MA	-23,3%	-27,0%
MT	-11,0%	-9,6%
MS	-9,4%	-16,0%
MG	-17,8%	-19,9%
PA	-14,5%	-18,8%
PB	-15,5%	-33,2%
PR	-15,1%	-19,4%
PE	-20,2%	-22,1%
PI	-20,9%	-36,4%
RJ	-20,7%	-17,2%
RN	-18,0%	-18,4%
RS	-16,3%	-26,9%
RO	-16,7%	-16,9%
RR	-9,3%	-11,8%
SC	-11,7%	-12,1%
SP	-22,0%	-14,9%
SE	-16,0%	-14,6%
TO	-12,4%	-18,1%

Fonte: Caixa Econômica Federal. Comitê de Dados - RS, 10/08/2020

Comitê de Dados

DISTANCIAMENTO CONTROLADO
GOV RS
NOVAS FAÇANHAS

Comitê de Dados

Dados, estudos, análises e projeções elaborados por mais de **120 colaboradores**, de 40 instituições

Elaborados colaborativamente apresentados ao Gabinete de Crise amplamente publicizados

Produtos

planejamento.rs.gov.br/comite-de-dados

- **Metodologia, operação e protocolos do Distanciamento Controlado**, em parceria com a Saúde
- **Matriz de risco** de paralisação e impactos da pandemia sobre serviços públicos
- **Estudo pioneiro de prevalência** da Covid-19 desde abril, em parceria com a UFPEL
- **Modelos epidemiológicos** e projeções para amparar protocolos e aumento na oferta de leitos
- **Boletins diários** da evolução da pandemia e das hospitalizações nas regiões
- **Estudos econômicos** sobre impacto no emprego, na atividade econômica, no PIB entre outros.
- **Monitoramentos** dos impactos em infraestrutura (água, energia), mobilidade e isolamento
- **Índices para distribuição** de recursos e renda mínima para populações em vulnerabilidade
- ***Benchmarking* de ações** para mitigar impactos de médio prazo sobre emprego e renda, segurança alimentar, saúde mental, evasão escolar entre outros.

GOVERNO DO ESTADO DO RIO GRANDE DO SUL
Governador: **Eduardo Leite**
Vice-Governador: **Ranolfo Vieira Júnior**

GABINETE DE CRISE PARA O ENFRENTAMENTO DA EPIDEMIA COVID-19
COMITÊ DE DADOS
Coordenadora: **Leany Barreiro de Sousa Lemos**

SECRETARIA DE PLANEJAMENTO, ORÇAMENTO E GESTÃO - **SEPLAG**
Secretário: **Claudio Leite Gastal**
Secretário-Adjunto de Planejamento e Orçamento: **Gilberto Pompilio de Melo Filho**
Secretário-Adjunto de Gestão: **Marcelo Soares Alves**

DEPARTAMENTO DE ECONOMIA E ESTATÍSTICA
DEPARTAMENTO DE PLANEJAMENTO GOVERNAMENTAL
ASSESSORIA DE GABINETE
ASSESSORIA DE COMUNICAÇÃO

SECRETARIA DE ESTADO DA SAÚDE - **SES**
Secretária: **Arita Bergmann**
Secretária-Adjunta: **Aglaé Regina da Silva**

DEPARTAMENTO DE ASSISTÊNCIA HOSPITALAR E AMBULATORIAL
DEPARTAMENTO DE AÇÕES EM SAÚDE
DEPARTAMENTO DE AUDITORIA DO SUS
DEPARTAMENTO DE REGULAÇÃO ESTADUAL
CENTRO ESTADUAL DE VIGILÂNCIA EM SAÚDE

Comitê de DADOS
COVID-19 CORONAVÍRUS

GOV RS
NOVAS FAÇANHAS

Atualizado em:
24/09/2020

MODELO DE DISTANCIAMENTO
CONTROLADO DO RS

Protocolos Gerais e Específicos

Obrigatórios e Setoriais

ULTIMAS ATUALIZAÇÕES

Protocolos Gerais: 05 de janeiro de 2021
Protocolos Específicos e Setoriais: 10 de fevereiro de 2021

Comitê de DADOS
COVID-19 CORONAVÍRUS

GOV RS
NOVAS FAÇANHAS
NO PLANEJAMENTO, GOVERNANÇA E GESTÃO NA SAÚDE

rs.gov.br

Protocolos

MODELO DE DISTANCIAMENTO CONTROLADO DO RS

CRITÉRIOS DE FUNCIONAMENTO (variáveis por bandeira)

- **Teto de Operação**
- **Modo de operação**
- **Horário de Funcionamento**

PROTOCOLOS OBRIGATÓRIOS (todas as bandeiras)

- **Máscara** (público, trabalhadores e alunos)
- **Distanciamento entre pessoas**
- **Teto de ocupação**
- **Higienização** (ambiente, trabalhadores, alunos e público)
- **Informativo visível**
- **EPIs obrigatórios**
- **Proteção de grupos de risco**
- **Afastamento de casos positivos ou suspeitos**
- **Cuidados no atendimento ao público**
- **Atendimento diferenciado para grupo de riscos**
- **Restrições adicionais**

PROTOCOLOS RECOMENDADOS

(não obrigatórios, variáveis por bandeiras e atividades)

- **Monitoramento de temperatura**
- **Testagem dos trabalhadores**

ÚLTIMA ATUALIZAÇÃO
Protocolos Gerais:
05 de janeiro de 2021

DISTANCIAMENTO CONTROLADO
GOV RS NOVAS FAÇANHAS

Os protocolos devem ser observados pelos empregadores, trabalhadores, clientes, alunos ou usuários em todas as bandeiras, sempre que houver qualquer atividade presencial desenvolvida em um ambiente de trabalho ou de ensino.

Regras Gerais

Para a abertura de estabelecimentos para atendimento ao público, deverão ser observadas na íntegra:

- as regras previstas no Decreto Estadual nº 55.240, de 10 de maio de 2020, que institui o Sistema de Distanciamento Controlado;
- as regras previstas no Decreto Estadual nº 55.241, de 10 de maio de 2020, que determina a aplicação das medidas sanitárias segmentadas;
- as Portarias da Secretaria de Saúde (SES-RS) para atividades específicas;
- as regras previstas na Portaria conjunta SES-SEDUC, que determina medidas de prevenção, monitoramento e controle ao novo coronavírus, a serem adotadas por todas as Instituições de Ensino no Estado.
- os atos das autoridades municipais competentes, fundamentados com respaldo em evidências científicas e em análises sobre as informações estratégicas em saúde.

Deverão ser adotadas medidas eficazes de fiscalização do cumprimento das cinco regras acima e dos protocolos delas decorrentes.

Recomenda-se que todos os estabelecimentos e todas as instituições de ensino elaborem **planos de contingência** para a operação das atividades presenciais, em conformidade com os protocolos que seguem.

CRITÉRIOS DE FUNCIONAMENTO (variáveis por bandeira)

Teto de Operação

O **teto de operação** de cada atividade estabelece o número máximo permitido de trabalhadores presentes, ao mesmo tempo, no ambiente de trabalho. É aplicado somente a atividades com quatro (4) ou mais trabalhadores.

O teto de operação também pode sinalizar o número máximo permitido de pessoas atendidas por uma atividade (ex.: 50% dos quartos de hotel disponíveis para operação ou 50% dos alunos presentes).

A finalidade última do teto de operação é reduzir a quantidade de pessoas circulando na cidade, ao mesmo tempo, conforme o maior ou o menor risco representado pelas bandeiras.

Para atender a essas restrições, sugere-se que sejam adotados regimes de escala, rodízio, horários alargados de entrada e saída e/ou turnos alternativos.

Atenção! O teto de operação deverá sempre respeitar o teto de ocupação de um ambiente. Ou seja, a atividade não poderá operar com número de trabalhadores ou público superior ao número máximo de pessoas permitido para o espaço físico livre, respeitando o distanciamento mínimo obrigatório (ver item específico, abaixo).

Por exemplo:
"Uma empresa funcionava em fevereiro de 2020 com **100 trabalhadores** em um **(1) único turno**. Seu galpão de produção contava com 240m^2 de área livre para circulação de pessoas. A empresa localiza-se em município cuja região está com bandeira laranja. Nessa bandeira, a atividade da empresa é limitada a **75% de teto operação**. Logo, somente seriam autorizados a operar ao mesmo tempo 75 trabalhadores nessa bandeira. No entanto, para respeitar o distanciamento mínimo entre as pessoas, a empresa deve obedecer ao limite máximo de pessoas nesse ambiente ao mesmo tempo (teto de ocupação). Esse limite, para uma área livre de 240m^2, é de 60 pessoas ao mesmo tempo. Portanto, **quando o teto de ocupação for menor que o teto de operação, o de ocupação prevalecerá. Nesse caso, se o empregador quiser funcionar em dois (2) turnos**, poderá operar com 50 pessoas em cada: 50 pessoas das 8h às 14h e 50 pessoas das 14h às 20h. Dessa forma, a empresa seguirá operando com a totalidade de sua força de trabalho, de 100 pessoas."

Modo de operação

Indica o modo de operação e/ou de atendimento de uma atividade, se estiver em funcionamento.

A atividade pode ser realizada de modo presencial, mas com as restrições aplicadas pelos protocolos a seguir, e/ou de maneiras alternativas, para que se mantenha funcionando (ex. teletrabalho, tele-atendimento, tele-entrega, pegue e leve, *drive-thru*, ensino remoto, atendimento individualizado, etc.)

Horário de Funcionamento

Critério recomendado para regulamentação municipal, conforme especificidades das atividades no município.

Sinaliza o horário de operação da atividade, se estiver em funcionamento.

Recomenda-se a manutenção dos horários normais para as atividades essenciais e a definição de horários de entrada e saída alternativos e flexíveis para atividades não essenciais, evitando a aglomeração de pessoas nas entradas e saídas dos estabelecimentos, nas ruas e no transporte urbano.

DISTANCIAMENTO CONTROLADO

PROTOCOLOS OBRIGATÓRIOS (todas as bandeiras)

Máscara (público, trabalhadores e alunos)

- É obrigatório utilizar máscara de proteção facial sempre que se estiver em ambiente coletivo fechado ou aberto, destinado à permanência ou circulação de pessoas, incluindo vias públicas, veículos de transporte, elevadores, salas de aula, repartições públicas ou privadas, lojas etc. **Não retirar a máscara para facilitar a comunicação, pois é justamente ao falar que se emitem mais partículas, ampliando as possibilidades de transmissão.**
- É permitido o uso de máscara de proteção facial do tipo cirúrgica descartável ou caseira, fabricada em tecido não tecido (TNT) ou tecido de algodão. Toda máscara é de **uso individual** e deve-se atentar para sua correta utilização, troca e higienização;
- É recomendado o uso de máscara tipo viseira (*face shield*) como uma proteção a mais, não substituindo o uso da máscara de proteção facial. A viseira não protege das menores partículas que percorrem o ar, tampouco desincentiva o hábito de levar as mãos ao nariz ou à boca, que são os maiores veículos de transmissão. Logo, recomenda-se o uso da máscara face *shield* somente quando acompanhada de máscara de proteção facial normal (cirúrgica descartável ou caseira de TNT ou algodão);
- É obrigatório orientar trabalhadores ou alunos quanto à correta utilização, troca e higienização da máscara de proteção facial (assista ao vídeo em: shorturl.at/iky17);
- É obrigatório exigir a utilização de máscara de proteção facial por usuários e clientes para ingresso e permanência no interior de ambiente público ou privado;
- É vedado o uso de máscara de proteção facial por criança menor de dois anos, pessoa que não seja capaz de removê-la sem assistência, assim como por qualquer pessoa durante o período de sono.
- É dever de todos observar a etiqueta respiratória, cobrindo a boca com o antebraço ou usando lenço descartável ao tossir ou espirrar. Descartar o lenço utilizado em uma lixeira fechada imediatamente após o uso.
- Mesmo com máscara de proteção facial, manter o distanciamento mínimo obrigatório (ver item específico).

Distanciamento entre pessoas

Distanciamento mínimo obrigatório entre pessoas em ambientes em geral:

- 2 metros sem máscara ou EPI;
- 1 metro com máscara ou EPI;

Distanciamento mínimo obrigatório entre pessoas em instituições de ensino:

- 2 metros sem máscara ou EPI;
- 1,5 metro com máscara ou EPI;

Nesse sentido:

- priorizar a modalidade de trabalho remoto para todos os trabalhadores que assim possam realizar suas atribuições, sem prejuízo às atividades;
- priorizar a modalidade de atendimento e de ensino remotos para todos os clientes, usuários e alunos que assim possam obter os serviços desejados, sem prejuízos;
- para aquelas atividades que não sejam possíveis de serem desempenhadas remotamente, adotar regimes de escala, revezamento, alteração de jornadas e/ou flexibilização de horários de entrada, saída, almoço ou intervalos, respeitando o teto de operação e o teto de ocupação dos ambientes (ver itens específicos);
- reorganizar as posições das mesas, estações de trabalho ou carteiras escolares para atender a distância mínima entre pessoas, marcando a posição de cada pessoa no chão no caso de atuação em pé;
- caso a mudança de posição das mesas ou estações de trabalho para atendimento do distanciamento mínimo não seja possível, reforçar o uso de EPIs (ver item específico) e/ou utilizar barreiras físicas entre as pessoas, fabricada em material liso, resistente, impermeável e que permita fácil higienização a cada troca de posto;
- vedar a realização de eventos e a realização de reuniões presenciais em áreas fechadas ou abertas. Quando não for possível cancelar ou a realizar as reuniões à distância, reduzir o número de participantes e sua duração, bem como disponibilizar álcool gel 70% e/ou preparações antissépticas ou sanitizantes de efeito similar e exigir o uso de máscara por todos os participantes;
- organizar o mobiliário escolar das salas de aula de forma a respeitar o distanciamento mínimo entre aluno, vedando a organização de classes escolares no formato de duplas ou grupos que desrespeitem o distanciamento mínimo obrigatório;
- evitar o uso de espaços comuns que facilitem a aglomeração de pessoas nas instituições de ensino, como pátios, refeitórios, ginásios, bibliotecas, entre outros, e escalonar os horários de intervalo, refeições, saída e entra de salas de aula, a fim de preservar o distanciamento mínimo obrigatório entre pessoas e evitar a aglomeração de alunos e trabalhadores nas áreas comuns;
- implementar corredores de sentido único para coordenar os fluxos de entrada e de saída dos estabelecimentos e instituições de ensino, respeitando o distanciamento mínimo entre pessoas;

Teto de ocupação

- Indica o número máximo permitido de pessoas presentes, simultaneamente, no interior de um estabelecimento, conforme as Normas de Prevenção e Proteção contra Incêndio e respeitado o distanciamento mínimo obrigatório de 1 metro entre pessoas com máscara ou EPI e 2 metros entre pessoas sem máscara ou EPI.
- Nas instituições de ensino, indica o distanciamento mínimo obrigatório de 1 metro entre pessoas com máscara é de 1,5 metro.
- Para fins de estabelecimento do teto de ocupação, respeitando o distanciamento mínimo obrigatório, recomenda-se o cômputo de **1 pessoa para cada 4m² de área livre**.

Por exemplo:

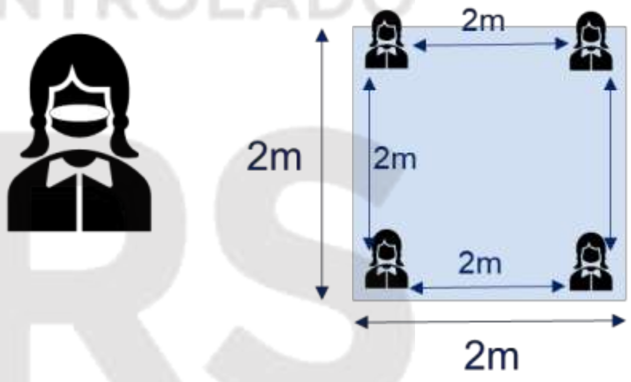

Área = Largura x Comprimento
Área = 2 x 2
Área = 4 m²

Considerando espaço de 40m² de área livre, por exemplo, o teto de ocupação será de 10 pessoas.

- Afixar cartaz com teto de ocupação permitido na entrada do espaço e em locais estratégicos, de fácil visualização, para monitoramento contínuo.

DISTANCIAMENTO CONTROLADO GOV RS NOVAS FAÇANHAS

Higienização (ambiente, trabalhadores, alunos e público)

- No início das atividades e durante o período de funcionamento, no mínimo a cada 2 horas, higienizar as superfícies de toque com álcool gel 70% e/ou preparações antissépticas ou sanitizantes de efeito similar, sob fricção (ex.: terminais de autoatendimento, corrimão de escadas e de acessos, maçanetas, interruptores, botões de elevadores, telefones, alça de carrinhos ou cestinhas de supermercado, etc.);
- Higienizar as máquinas para pagamento com cartão com álcool 70% e/ou preparações antissépticas ou sanitizantes de efeito similar após cada uso;
- Higienizar pisos, paredes, forro de banheiro, refeitórios, vestiários, etc. no mínimo a cada turno e a cada dia nos transportes coletivos, preferencialmente com álcool em 70%, hipoclorito de sódio 0,1% (água sanitária) ou outro desinfetante indicado para este fim;
- Higienizar mesas, cadeiras, teclados, mouses, telefones a cada turno, com álcool 70% e/ou preparações antissépticas ou sanitizantes de efeito similar;
- Nas instituições de ensino, higienizar, a cada uso, materiais e utensílios de uso comum como colchonetes, tatames, trocadores, cadeiras de alimentação, berços entre outros, e desincentivar o compartilhamento de brinquedos e materiais escolares, os quais, na impossibilidade de uso individual, deverão ser higienizados a cada uso;
- Dispor de lixeira com tampa com dispositivo que permita a abertura e fechamento sem o uso das mãos (pedal ou outro tipo de dispositivo) e recolher e descartar os resíduos a cada 2 horas, com segurança;
- Exigir que clientes, trabalhadores, alunos ou usuários higienizem as mãos com álcool em gel 70% e/ou preparações antissépticas ou sanitizantes de efeito similar ao acessarem e ao saírem do estabelecimento.
- Disponibilizar kit completo nos banheiros (álcool gel 70% e/ou preparações antissépticas ou sanitizantes de efeito similar, sabonete líquido e toalhas de papel não reciclado);
- Manter limpos filtros e dutos do ar condicionado;
- Manter portas e janelas abertas, com ventilação adequada, exceto em locais em que não seja permitido por questões sanitárias;
- Instruir trabalhadores e alunos sobre a etiqueta respiratória e de higiene e de prevenção, incentivando a lavagem das mãos a cada 2 horas, com água e sabão, por no mínimo 20 segundos, bem como orientando para não cumprimentar pessoas com apertos de mão, abraços, beijos ou outro tipo de contato físico;
- Recomendar aos trabalhadores que não retornem às suas casas com o uniforme utilizado durante a prestação do serviço;
- Dar preferência à utilização de talheres e copos descartáveis e, na impossibilidade, utilizar talheres higienizados e individualizados (sem contato);
- Substituir os sistemas de autosserviço de bufê em refeitórios, utilizando porções individualizadas ou disponibilizando funcionário(s) específico(s) para servir todos os pratos;
- Eliminar bebedouros verticais ou de jato inclinado e disponibilizar alternativas (dispensadores de água e copos plásticos descartáveis e/ou copos de uso individual, desde que constantemente higienizados).

DISTANCIAMENTO CONTROLADO
GOV RS
NOVAS FAÇANHAS

Informativo visível

- Afixar na entrada do estabelecimento e em locais estratégicos, de fácil visualização do público, dos trabalhadores e/ou dos alunos, cartazes contendo:
  - informações sanitárias sobre higienização e cuidados para a prevenção à COVID-19, tais como necessidade de higienização das mãos, uso de máscara, distanciamento entre as pessoas, limpeza de superfícies, ventilação e limpeza dos ambientes;
  - indicação do teto de ocupação do ambiente;
  - indicação do teto de operação vigente da atividade realizada pelo estabelecimento;
- Nas instituições de ensino, os cartazes informativos deverão ser redigidos com linguagem acessível para toda a comunidade escolar

EPIs obrigatórios

- O empregador deve fornecer e orientar a correta utilização de Equipamentos de Proteção Individual (EPIs) **adequados para a atividade exercida e em quantidade suficiente** para cada trabalhador, conforme especificado nas Normas Regulamentadoras da Secretaria de Trabalho do Ministério da Economia, das normas e recomendações do Ministério da Saúde e da SES-RS, das Normas Regulamentadoras da atividade e das normas ABNT;
- Proibir a reutilização de uniformes e/ou EPIs (capacetes, calçados de segurança, entre outros) quando tais vestimentas/equipamentos não sejam devidamente higienizados com preparações antissépticas ou sanitizantes de efeito similar;
- Caso a atividade não possua protocolo específico de EPIs, o empregador deverá fornecer máscaras descartáveis em quantidades suficientes e/ou no mínimo duas máscaras de tecido não tecido (TNT) ou tecido de algodão para cada trabalhador, que ficará responsável por sua correta utilização, troca e higienização;
- Adotar rotinas de instrução permanente dos trabalhadores quanto à correta utilização, higienização e descarte de EPIs.

Proteção de grupos de risco no trabalho

- Os alunos de grupos de risco devem permanecer em casa, em regime de ensino remoto;
- Os trabalhadores de grupos de risco podem solicitar ao empregador permanecer em casa, em regime de teletrabalho, sempre que possível;
- Quando a permanência do trabalhador de grupos de risco em casa não for possível, deve-se assegurar que suas atividades sejam realizadas em ambiente com menor exposição de risco de contaminação;
- Caso um trabalhador resida com pessoa do grupo de risco, fica a critério do empregador o seu afastamento para regime de teletrabalho, se possível;

Pertencem aos **grupos de risco**, pessoas com:

- Cardiopatias graves ou descompensados (insuficiência cardíaca, cardiopata isquêmica, arritmias)
- Pneumopatias graves ou descompensados (em uso de oxigênio domiciliar; asma moderada/grave, doença pulmonar obstrutiva crônica - DPOC)
- Imunodepressão
- Doenças renais crônicas em estágio avançado (graus 3, 4 e 5)
- Diabetes mellitus, conforme juízo clínico
- Obesidade mórbida (IMC maior ou igual a 40)
- Doenças cromossômicas com estado de fragilidade imunológica (ex.: Síndrome de Down)
- Idade igual ou superior a 60 anos com uma ou mais comorbidades acima relacionadas
- Gestação de alto risco

\+ outras que Ministério da Saúde e/ou a SES-RS definirem.

Afastamento de casos positivos ou suspeitos

- Orientar os trabalhadores e os alunos a informar o estabelecimento caso venham a ter sintomas de síndrome gripal e/ou resultados positivos para a COVID-19;
- Realizar busca ativa, diária, em todos os turnos de trabalho ou de aula, para identificar trabalhadores, alunos ou visitantes com sintomas de síndrome gripal;
- Encaminhar imediatamente para atendimento médico e garantir o imediato afastamento para isolamento domiciliar de 14 dias, a contar do início dos sintomas, ou conforme determinação médica, os trabalhadores e alunos que:
  - testarem positivos para COVID-19;
  - tenham tido contato ou residam com caso confirmado de COVID-19;
  - apresentarem sintomas de síndrome gripal.
- Manter registro atualizado do acompanhamento de todos os trabalhadores e alunos afastados para isolamento domiciliar (quem, quando, suspeito/confirmado, em que data, serviço de saúde onde é acompanhado, se for o caso, etc.)
- Notificar imediatamente os casos suspeitos de síndrome gripal e os confirmados de COVID-19 à Vigilância em Saúde do Município do estabelecimento, bem como à Vigilância em Saúde do Município de residência do trabalhador ou aluno;
- Desenvolver e comunicar planos de continuidade das atividades na ausência de trabalhadores e alunos devido a afastamento por suspeita ou confirmação de COVID-19.
- Coletar os dados de presentes em reuniões presenciais, a fim de facilitar o contato dos órgãos de saúde competentes com o público da reunião, no caso de uma confirmação de COVID-19 dentre os participantes;
- Estabelecer grupos fixos de trabalhadores entre as diferentes áreas da fábrica, a fim de facilitar o contato dos órgãos de saúde competentes com o grupo no caso de uma confirmação de COVID-19 dentre os trabalhadores;

DISTANCIAMENTO CONTROLADO
GOV RS NOVAS FACANHAS

(*) São **sintomas de síndrome gripal**: quadro respiratório agudo, caracterizado por sensação febril ou febre, mesmo que relatada, acompanhada de tosse OU dor de garganta OU coriza OU dificuldade respiratória.

(**) Um surto de síndrome gripal ocorre quando há, pelo menos, 2 (dois) casos suspeitos, sintomáticos, com vínculo temporal de até 7 dias entre as datas de início dos sintomas dos casos. Em caso de suspeita de surto no estabelecimento, notificar a Vigilância em Saúde do Município para que seja desencadeada uma investigação detalhada, a fim de identificar novos casos e interromper o surto.

Para suspeitas de surtos em empresas, confira as orientações da Nota Informativa 08/2020 COE-RS/SES-RS, de 28 de abril de 2020. Para suspeitas de surtos em Instituições de Longa Permanência de Idosos – ILPIs, confira a Nota Informativa COE-RS/SES-RS, de 22 de abril de 2020.

Cuidados no atendimento ao público

- Disponibilizar de álcool gel 70% e/ou preparações antissépticas ou sanitizantes de efeito similar para o público, os trabalhadores e alunos no estabelecimento, em locais estratégicos e de fácil acesso (entrada, saída, corredores, elevadores, mesas, etc.);
- Respeitar o distanciamento mínimo de 2 metros nas filas em frente a balcões de atendimento ou caixas ou no lado externo do estabelecimento, sinalizando no chão a posição a ser ocupada por cada pessoa;
- Assegurar o respeito de distanciamento mínimo de 2 metros no lado externo da instituição de ensino para pais e cuidadores que esperam os alunos na saída, sinalizando no chão a posição a ser ocupada por cada pessoa
- Fazer a utilização, se necessário, do uso de senhas ou outro sistema eficaz para evitar filas ou aglomeração de pessoas;
- Ampliar espaço entre atendimentos agendados, para preservar distanciamento entre pessoas e ter tempo de realizar a higienização de instrumentos de contato, quando aplicável;
- Realizar atendimento de maneira individualizada, restringindo, sempre que possível, a presença de acompanhantes;
- Em serviço de atendimento domiciliar ou agendado, questionar se no local de atendimento há indivíduo que apresenta sintomas respiratórios ou se se encontra em quarentena ou isolamento em decorrência do COVID-19, ficando proibido o atendimento domiciliar em caso afirmativo, exceto em caso de urgência e emergência de saúde;

Atendimento diferenciado para grupo de riscos

Para atendimento de pessoa com idade igual ou superior a 60 anos e aquelas de grupos de risco, conforme autodeclaração:

- estabelecer horários ou setores exclusivos de atendimento;
- conferir atendimento preferencial, garantindo fluxo ágil para que permaneçam o mínimo de tempo possível no estabelecimento.

Restrições adicionais

Além dos protocolos acima, algumas atividades devem atender, na íntegra, os decretos estaduais e as respectivas portarias específicas publicadas pela Secretaria Estadual de Saúde, disponíveis em: https://coronavirus.rs.gov.br/portarias-da-ses

PROTOCOLOS RECOMENDADOS
(não obrigatórios, variáveis por bandeiras e atividades)

Monitoramento de temperatura

- Aferir a temperatura de 100% dos trabalhadores, clientes ou alunos, com termômetro digital infravermelho.
- Monitorar individualmente a temperatura, com termômetro próprio e individual, para evitar contaminação.

Caso a temperatura seja igual ou superior a 37,8 graus, orientar que o trabalhador, o cliente ou o usuário acompanhe seus sintomas e busque um serviço de saúde para investigação diagnóstica.

Recomenda-se vedar a circulação dessas pessoas em ambiente coletivo compartilhado.

Nas instituições de ensino, em caso de aluno(a) febril, o COE-E local deve ser informado imediatamente.

Testagem dos trabalhadores

- Aplicar testagem rápida ou sorológica em trabalhadores que mantiverem rotina de trabalho presencial, frequentando ambientes compartilhados.

Para suspeitas de surtos em empresas, confira as orientações da Nota Informativa 08/2020 COE-RS/SES-RS, de 28 de abril de 2020. Para suspeitas de surtos em Instituições de Longa Permanência de Idosos – ILPIs, confira a Nota Informativa COE-RS/SES-RS, de 22 de abril de 2020.

MODELO DE DISTANCIAMENTO
CONTROLADO DO RS

Protocolos Específicos

Todos os Setores

ULTIMAS ATUALIZAÇÕES

Protocolos Gerais: 05 de janeiro de 2021
Protocolos Específicos e Setoriais: 10 de fevereiro de 2021

rs.gov.br

Comitê de DADOS
COVID-19 CORONAVÍRUS

GOV RS
NOVAS FAÇANHAS
NO PLANEJAMENTO, GOVERNANÇA E GESTÃO NA SAÚDE

MODELO DE DISTANCIAMENTO
CONTROLADO DO RS

DISTANCIAMENTO CONTROLADO
GOV RS
NOVAS FAÇANHAS

Administração Pública

Comitê de DADOS
COVID-19 CORONAVÍRUS
GOV RS
NOVAS FAÇANHAS
NO PLANEJAMENTO, GOVERNANÇA E GESTÃO NA SAÚDE

BANDEIRA AMARELA - Administração Pública

// Atividade				// Critérios específicos de funcionamento (conforme bandeira)				// Protocolos obrigatório (todas as bandeiras)	// Protocolos variáveis (recomendados)		// Restrições adicionais
Grupo	CNAE (2 díg.)	Tipo	Subtipos	**Teto de Operação** Determina o percentual máximo de trabalhadores/público externo presentes no mesmo turno, ao mesmo tempo. Deve respeitar ao nº máximo de pessoas no espaço físico, considerando o distanciamento interpessoal mínimo obrigatório (teto de ocupação).		**Modo de Operação** Forma de operação da atividade, respeitando ao teto de operação, ao teto de ocupação do espaço físico e aos protocolos obrigatórios (ao lado). Trabalhadores	Atendimento	**Decreto nº 55.2540:** - Máscara / EPIs, - Distanciamento, - Teto de ocupação, - Higienização, - Proteção de grupo de risco, - Afastamento de casos, - Cuidados com o público, - Atendimento do grupos de risco - Informativo visível (operação, ocupação e cuidados)	**Monitora-mento de tempera- tura**	**Testagem dos trabalha-dores**	Conteúdo completo das normas obrigatórias específicas à atividade: coronavirus.rs.gov.br/portarias-da-ses
Administração Pública	84	Administração Pública	Administração Pública - Serviços não essenciais		100% trabalhadores (ou normativa municipal)	Teletrabalho / Presencial restrito	Teleatendimento / Presencial restrito	X			Decreto nº 55.240, Capítulo VI (Estadual)
Administração Pública	84	Administração Pública	Locais públicos abertos, sem controle de acesso (ruas, calçadas, parques, praças, faixa de areia, mar, lagoa, rio e similares)		100% lotação	Presencial restrito / Distanciamento interpessoal mínimo de 1m / Uso obrigatório de máscara (cobrindo boca e nariz) / Decreto municipal e fiscalização para coibir aglomeração.		X			
Administração Pública	84	Administração Pública	Segurança e ordem pública		100% trabalhadores	Teletrabalho / Presencial restrito	Teleatendimento / Presencial restrito	X			Decreto nº 55.240, Capítulo VI (Estadual)
Administração Pública	84	Administração Pública	Política e administração de trânsito		100% trabalhadores (ou normativa municipal)	Teletrabalho / Presencial restrito	Teleatendimento / Presencial restrito	X			Decreto nº 55.240, Capítulo VI (Estadual)
Administração Pública	84	Administração Pública	Atividades de fiscalização		100% trabalhadores	Teletrabalho / Presencial restrito	Teleatendimento / Presencial restrito	X			Decreto nº 55.240, Capítulo VI (Estadual)
Administração Pública	84	Administração Pública	Inspeção sanitária		100% trabalhadores	Teletrabalho / Presencial restrito	Teleatendimento / Presencial restrito	X			Decreto nº 55.240, Capítulo VI (Estadual)
Administração Pública	84	Administração Pública	Serviços delegados de habilitação de condutores		100% trabalhadores	Teletrabalho / Presencial restrito	Ensino remoto (aula teórica) / Atendimento individualizado (aula prática)	X			

DISTANCIAMENTO CONTROLADO
GOV RS
NOVAS FAÇANHAS

BANDEIRA LARANJA - Administração Pública

// Atividade				// Critérios específicos de funcionamento (conforme bandeira)			// Protocolos obrigatório (todas as bandeiras)	// Protocolos variáveis (recomendados)		// Restrições adicionais
Grupo	CNAE (2 díg.)	Tipo	Subtipos	**Teto de Operação**<br>Determina o percentual máximo de trabalhadores/público externo presentes no mesmo turno, ao mesmo tempo.<br><br>Deve respeitar ao nº máximo de pessoas no espaço físico, considerando o distanciamento interpessoal mínimo obrigatório (teto de ocupação).	**Modo de Operação**<br>Forma de operação da atividade, respeitando ao teto de operação, ao teto de ocupação do espaço físico e aos protocolos obrigatórios (ao lado).<br><br>Trabalhadores	Atendimento	**Decreto nº 55.2540:**<br>- Máscara / EPIs,<br>- Distanciamento,<br>- Teto de ocupação,<br>- Higienização,<br>- Proteção de grupo de risco,<br>- Afastamento de casos,<br>- Cuidados com o público,<br>- Atendimento do grupos de risco<br>- Informativo visível (operação, ocupação e cuidados)	**Monitora-mento de tempera- tura**	**Testagem dos trabalha-dores**	Conteúdo completo das normas obrigatórias específicas à atividade: coronavirus.rs.gov.br/portarias-da-ses
Administração Pública	84	Administração Pública	Administração Pública - Serviços não essenciais	75% trabalhadores (ou normativa municipal)	Teletrabalho / Presencial restrito	Teleatendimento / Presencial restrito	X			Decreto nº 55.240, Capítulo VI (Estadual)
Administração Pública	84	Administração Pública	Locais públicos abertos, sem controle de acesso (ruas, calçadas, parques, praças, faixa de areia, mar, lagoa, rio e similares)	100% lotação		Presencial restrito / Distanciamento interpessoal mínimo de 1m / Uso obrigatório de máscara (cobrindo boca e nariz) /<br><br>Decreto municipal e fiscalização para coibir aglomeração.	X			
Administração Pública	84	Administração Pública	Segurança e ordem pública	100% trabalhadores	Teletrabalho / Presencial restrito	Teleatendimento / Presencial restrito	X			Decreto nº 55.240, Capítulo VI (Estadual)
Administração Pública	84	Administração Pública	Política e administração de trânsito	75% trabalhadores (ou normativa municipal)	Teletrabalho / Presencial restrito	Teleatendimento / Presencial restrito	X			Decreto nº 55.240, Capítulo VI (Estadual)
Administração Pública	84	Administração Pública	Atividades de fiscalização	100% trabalhadores	Teletrabalho / Presencial restrito	Teleatendimento / Presencial restrito	X			Decreto nº 55.240, Capítulo VI (Estadual)
Administração Pública	84	Administração Pública	Inspeção sanitária	100% trabalhadores	Teletrabalho / Presencial restrito	Teleatendimento / Presencial restrito	X			Decreto nº 55.240, Capítulo VI (Estadual)
Administração Pública	84	Administração Pública	Serviços delegados de habilitação de condutores	75% trabalhadores	Teletrabalho / Presencial restrito	Ensino remoto (aula teórica) / Atendimento individualizado (aula prática)	X			

DISTANCIAMENTO CONTROLADO
iGOV RS
NOVAS FAÇANHAS

BANDEIRA VERMELHA - Administração Pública

// Atividade				// Critérios específicos de funcionamento (conforme bandeira)			// Protocolos obrigatório (todas as bandeiras)	// Protocolos variáveis (recomendados)		// Restrições adicionais
Grupo	CNAE (2 díg.)	Tipo	Subtipos	**Teto de Operação**<br>Determina o percentual máximo de trabalhadores/público presentes no mesmo turno, ao mesmo tempo.<br><br>Deve respeitar ao nº máximo de pessoas no espaço físico, considerando o distanciamento interpessoal mínimo obrigatório (teto de ocupação).	**Modo de Operação**<br>Forma de operação da atividade, respeitando ao teto de operação, ao teto de ocupação do espaço físico e aos protocolos obrigatórios (ao lado).<br><br>**Trabalhadores**	**Atendimento**	**Decreto nº 55.2540:**<br>- Máscara / EPIs,<br>- Distanciamento,<br>- Teto de ocupação,<br>- Higienização,<br>- Proteção de grupo de risco,<br>- Afastamento de casos,<br>- Cuidados com o público,<br>- Atendimento do grupos de risco<br>- Informativo visível (operação, ocupação e cuidados)	**Monitora- mento de tempera- tura**	**Testagem dos trabalha- dores**	Conteúdo completo das normas obrigatórias específicas à atividade: coronavirus.rs.gov.br/portarias-da-ses
Administração Pública	84	Administração Pública	Administração Pública - Serviços não essenciais	50% trabalhadores (ou normativa municipal)	Teletrabalho / Presencial restrito	Teleatendimento / Presencial restrito	X	X		Decreto nº 55.240, Capítulo VI (Estadual)
Administração Pública	84	Administração Pública	Locais públicos abertos, sem controle de acesso (ruas, calçadas, parques, praças, faixa de areia, mar, lagoa, rio e similares)	50% lotação		Proibido permanência / Permitido apenas circulação e realização de exercícios físicos / Distanciamento interpessoal mínimo de 1m / Uso obrigatório e correto de máscara, cobrindo boca e nariz /<br><br>Decreto municipal poderá autorizar permanência, desde que conte com mecanismos para viabilizar fiscalização para coibir aglomeração.	X			
Administração Pública	84	Administração Pública	Segurança e ordem pública	100% trabalhadores	Teletrabalho / Presencial restrito	Teleatendimento / Presencial restrito	X			Decreto nº 55.240, Capítulo VI (Estadual)
Administração Pública	84	Administração Pública	Política e administração de trânsito	75% trabalhadores (ou normativa municipal)	Teletrabalho / Presencial restrito	Teleatendimento / Presencial restrito	X			Decreto nº 55.240, Capítulo VI (Estadual)
Administração Pública	84	Administração Pública	Atividades de fiscalização	100% trabalhadores	Teletrabalho / Presencial restrito	Teleatendimento / Presencial restrito	X			Decreto nº 55.240, Capítulo VI (Estadual)
Administração Pública	84	Administração Pública	Inspeção sanitária	100% trabalhadores	Teletrabalho / Presencial restrito	Teleatendimento / Presencial restrito	X			Decreto nº 55.240, Capítulo VI (Estadual)
Administração Pública	84	Administração Pública	Serviços delegados de habilitação de condutores	50% trabalhadores	Teletrabalho / Presencial restrito	Ensino remoto (aula teórica) / Atendimento individualizado (aula prática)	X	X		

DISTANCIAMENTO CONTROLADO RS NOVAS FAÇANHAS

BANDEIRA PRETA - Administração Pública

// Atividade				// Critérios específicos de funcionamento (conforme bandeira)			// Protocolos obrigatório (todas as bandeiras)	// Protocolos variáveis (recomendados)		// Restrições adicionais
Grupo	CNAE (2 díg.)	Tipo	Subtipos	**Teto de Operação**<br>Determina o percentual máximo de trabalhadores/público presentes no mesmo turno, ao mesmo tempo.<br><br>Deve respeitar ao nº máximo de pessoas no espaço físico, considerando o distanciamento interpessoal mínimo obrigatório (teto de ocupação).	**Modo de Operação**<br>Forma de operação da atividade, respeitando ao teto de operação, ao teto de ocupação do espaço físico e aos protocolos obrigatórios (ao lado).<br><br>**Trabalhadores**	**Atendimento**	**Decreto nº 55.2540:**<br>- Máscara / EPIs,<br>- Distanciamento,<br>- Teto de ocupação,<br>- Higienização,<br>- Proteção de grupo de risco,<br>- Afastamento de casos,<br>- Cuidados com o público,<br>- Atendimento do grupos de risco<br>- Informativo visível (operação, ocupação e cuidados)	**Monitora- mento de tempera- tura**	**Testagem dos trabalha- dores**	Conteúdo completo das normas obrigatórias específicas à atividade: coronavirus.rs.gov.br/portarias-da-ses
Administração Pública	84	Administração Pública	Administração Pública - Serviços não essenciais	25% trabalhadores (ou normativa municipal)	Teletrabalho / Presencial restrito	Teleatendimento / Presencial restrito	X	X		Decreto nº 55.240, Capítulo VI (Estadual)
Administração Pública	84	Administração Pública	Locais públicos abertos, sem controle de acesso (ruas, calçadas, parques, praças, faixa de areia, mar, lagoa, rio e similares)	50% lotação		Proibido permanência<br><br>Permitido apenas circulação / Distanciamento interpessoal mínimo de 1m / Uso obrigatório e correto de máscara, cobrindo boca e nariz /	X			
Administração Pública	84	Administração Pública	Segurança e ordem pública	100% trabalhadores	Teletrabalho / Presencial restrito	Teleatendimento / Presencial restrito	X			Decreto nº 55.240, Capítulo VI (Estadual)
Administração Pública	84	Administração Pública	Política e administração de trânsito	75% trabalhadores (ou normativa municipal)	Teletrabalho / Presencial restrito	Teleatendimento / Presencial restrito	X			Decreto nº 55.240, Capítulo VI (Estadual)
Administração Pública	84	Administração Pública	Atividades de fiscalização	100% trabalhadores	Teletrabalho / Presencial restrito	Teleatendimento / Presencial restrito	X			Decreto nº 55.240, Capítulo VI (Estadual)
Administração Pública	84	Administração Pública	Inspeção sanitária	100% trabalhadores	Teletrabalho / Presencial restrito	Teleatendimento / Presencial restrito	X			Decreto nº 55.240, Capítulo VI (Estadual)
Administração Pública	84	Administração Pública	Serviços delegados de habilitação de condutores	25% trabalhadores	Teletrabalho / Presencial restrito	Ensino remoto (aula teórica) / Atendimento individualizado (aula prática)	X	X		

MODELO DE DISTANCIAMENTO
CONTROLADO DO RS

Agropecuária

Comitê de DADOS
COVID-19 CORONAVÍRUS

GOV RS
NOVAS FAÇANHAS
NO PLANEJAMENTO, GOVERNANÇA E GESTÃO NA SAÚDE

BANDEIRA AMARELA - Agropecuária

// Atividade				// Critérios específicos de funcionamento (conforme bandeira)			// Protocolos obrigatório (todas as bandeiras)	// Protocolos variáveis (recomendados)		// Restrições adicionais
Grupo	CNAE (2 díg.)	Tipo	Subtipos	Teto de Operação (percentual máx. de trabalhadores presentes no turno, ao mesmo tempo, respeitando o teto de ocupação do espaço físico - máx. pessoas)	Modo de Operação (forma de operação, respeitando o teto de operação e o teto de ocupação do espaço físico - máx. pessoas)<br>Trabalhadores	Atendimento	Informativo visível (operação e ocupação) Máscara / EPIs, Distanciamento, Teto de ocupação, Higienização, Proteção de grupo de risco, Afastamento de casos, Cuidados no atendimento ao	Monitora- mento de tempera- tura	Testagem dos trabalha- dores	Normas obrigatórias específicas à atividade https://coronavirus.rs.gov.br/portarias-da-ses
Agropecuária	1	Agricultura, Pecuária e Serv. Relacionados		100% trabalhadores	Teletrabalho / Presencial restrito		X			
Agropecuária	2	Produção Florestal		100% trabalhadores	Teletrabalho / Presencial restrito		X			
Agropecuária	3	Pesca e Aqüicultura		100% trabalhadores	Teletrabalho / Presencial restrito		X			

DISTANCIAMENTO CONTROLADO
GOV RS
NOVAS FAÇANHAS

BANDEIRA LARANJA - Agropecuária

// Atividade				// Critérios específicos de funcionamento (conforme bandeira)			// Protocolos obrigatório (todas as bandeiras)	// Protocolos variáveis (recomendados)		// Restrições adicionais
Grupo	CNAE (2 díg.)	Tipo	Subtipos	Teto de Operação (percentual máx. de trabalhadores presentes no turno, ao mesmo tempo, respeitando o teto de ocupação do espaço físico - máx. pessoas)	Modo de Operação (forma de operação, respeitando o teto de operação e o teto de ocupação do espaço físico - máx. pessoas) Trabalhadores	Atendimento	Informativo visível (operação e ocupação) Máscara / EPIs, Distanciamento, Teto de ocupação, Higienização, Proteção de grupo de risco, Afastamento de casos, Cuidados no atendimento ao	Monitora- mento de tempera- tura	Testagem dos trabalha- dores	Normas obrigatórias específicas à atividade https://coronavirus.rs.gov.br/portarias-da-ses
Agropecuária	1	Agricultura, Pecuária e Serv. Relacionados		100% trabalhadores	Teletrabalho / Presencial restrito		X			
Agropecuária	2	Produção Florestal		100% trabalhadores	Teletrabalho / Presencial restrito		X			
Agropecuária	3	Pesca e Aqüicultura		100% trabalhadores	Teletrabalho / Presencial restrito		X			

DISTANCIAMENTO CONTROLADO
GOV RS
NOVAS FAÇANHAS

BANDEIRA VERMELHA - Agropecuária

// Atividade				// Critérios específicos de funcionamento (conforme bandeira)			// Protocolos obrigatório (todas as bandeiras)	// Protocolos variáveis (recomendados)		// Restrições adicionais
Grupo	CNAE (2 díg.)	Tipo	Subtipos	Teto de Operação (percentual máx. de trabalhadores presentes no turno, ao mesmo tempo, respeitando o teto de ocupação do espaço físico - máx. pessoas)	Modo de Operação (forma de operação, respeitando o teto de operação e o teto de ocupação do espaço físico - máx. pessoas)<br>Trabalhadores	Atendimento	Informativo visível (operação e ocupação) Máscara / EPIs, Distanciamento, Teto de ocupação, Higienização, Proteção de grupo de risco, Afastamento de casos, Cuidados no atendimento ao público, Atendimento do grupos de risco	Monitora- mento de tempera- tura	Testagem dos trabalha- dores	Normas obrigatórias específicas à atividade https://coronavirus.rs.gov.br/portarias-da-ses
Agropecuária	1	Agricultura, Pecuária e Serv. Relacionados		75% trabalhadores	Teletrabalho / Presencial restrito		X			
Agropecuária	2	Produção Florestal		75% trabalhadores	Teletrabalho / Presencial restrito		X			
Agropecuária	3	Pesca e Aqüicultura		75% trabalhadores	Teletrabalho / Presencial restrito		X			

DISTANCIAMENTO CONTROLADO
GOV RS
NOVAS FAÇANHAS

BANDEIRA PRETA - Agropecuária

// Atividade				// Critérios específicos de funcionamento (conforme bandeira)			// Protocolos obrigatório (todas as bandeiras)	// Protocolos variáveis (recomendados)		// Restrições adicionais
Grupo	CNAE (2 díg.)	Tipo	Subtipos	Teto de Operação (percentual máx. de trabalhadores presentes no turno, ao mesmo tempo, respeitando o teto de ocupação do espaço físico - máx. pessoas)	Modo de Operação (forma de operação, respeitando o teto de operação e o teto de ocupação do espaço físico - máx. pessoas)<br>Trabalhadores	Atendimento	Informativo visível (operação e ocupação) Máscara / EPIs, Distanciamento, Teto de ocupação, Higienização, Proteção de grupo de risco, Afastamento de casos, Cuidados no atendimento ao público, Atendimento do grupos de risco	Monitora- mento de tempera- tura	Testagem dos trabalha- dores	Normas obrigatórias específicas à atividade https://coronavirus.rs.gov.br/portarias-da-ses
Agropecuária	1	Agricultura, Pecuária e Serv. Relacionados		75% trabalhadores	Teletrabalho / Presencial restrito		X			
Agropecuária	2	Produção Florestal		75% trabalhadores	Teletrabalho / Presencial restrito		X			
Agropecuária	3	Pesca e Aqüicultura		75% trabalhadores	Teletrabalho / Presencial restrito		X			

DISTANCIAMENTO CONTROLADO
GOV RS
NOVAS FAÇANHAS

MODELO DE DISTANCIAMENTO
CONTROLADO DO RS

Alojamento e Alimentação

DISTANCIAMENTO CONTROLADO
GOV RS NOVAS FAÇANHAS

Comitê de DADOS
COVID-19 CORONAVÍRUS

GOV RS
NOVAS FAÇANHAS
NO PLANEJAMENTO, GOVERNANÇA E GESTÃO NA SAÚDE

BANDEIRA AMARELA - Alojamento e Alimentação

// Atividade				// Critérios específicos de funcionamento (conforme bandeira)			// Protocolos obrigatório (todas as bandeiras)	// Protocolos variáveis (recomendados)		// Restrições adicionais
Grupo	CNAE (2 díg.)	Tipo	Subtipos	Teto de Operação (percentual máx. de trabalhadores presentes no turno, ao mesmo tempo, respeitando o teto de ocupação do espaço físico - máx. pessoas)	Modo de Operação (forma de operação, respeitando o teto de operação e o teto de ocupação do espaço físico - máx. pessoas) Trabalhadores	Atendimento	Informativo visível (operação e ocupação) Máscara / EPIs, Distanciamento, Teto de ocupação, Higienização, Proteção de grupo de risco, Afastamento de casos, Cuidados no atendimento ao	Monitora- mento de tempera- tura	Testagem dos trabalha- dores	Normas obrigatórias específicas à atividade https://coronavirus.rs.gov.br/portarias-da-ses
Alojamento e Alimentação	56	Alimentação	Restaurantes a la carte, prato feito e buffet sem autosserviço	75% trabalhadores<br>75% lotação	Teletrabalho / Presencial restrito	Presencial restrito / Telentrega / Pegue e Leve / Drive-thru	X			Portaria SES nº 319
Alojamento e Alimentação	56	Alimentação	Restaurantes a la carte, prato feito e buffet sem autosserviço (em beira de estradas e rodovias)	75% trabalhadores<br>75% lotação	Teletrabalho / Presencial restrito	Presencial restrito / Telentrega / Pegue e Leve / Drive-thru	X			Portaria SES nº 319
Alojamento e Alimentação	56	Alimentação	Restaurantes de autosserviço (self-service)	75% trabalhadores<br>75% lotação	Teletrabalho / Presencial restrito / Funcionário(a) orientando no início da fila para o correto atendimento dos protocolos (máscara, álcool gel e distanciamento na fila) / Protetor salivar nos buffets / Higienização e troca constante dos talheres e pegadores do buffet / Talheres embalados individualmente / Demais protocolos da Portaria SES nº 319	Telentrega / Pegue e Leve / Drive-thru / Presencial restrito / Utilização obrigatória de máscara ao se servir e ao circular / Permitido retirar a máscara somente para se alimentar, sentado às mesas / Distanciamento mínimo de 1m entre pessoas na fila do buffet, com marcação no chão / Acesso com entrada e sentido único no buffet, com funcionário(a) aplicando e orientando o correto uso do álcool em gel / Uso obrigatório de álcool em gel 70% em fricção imediatamente antes de se servir no buffet / Utilização de prato limpo a cada vez que cliente se servir / Reforço no distanciamento mínimo de 2m entre as mesas	X			Portaria SES nº 319
Alojamento e Alimentação	56	Alimentação	Lanchonetes, lancherias e bares	75% trabalhadores<br>75% lotação	Teletrabalho / Presencial restrito	Presencial restrito / Telentrega / Pegue e Leve / Drive-thru	X			Portaria SES nº 319
Alojamento e Alimentação	56	Alojamento	Hotéis e similares (geral)	Estabelecimentos sem o Selo Turismo Responsável do MTur:<br>**75% de lotação**<br><br>Estabelecimentos com Selo Turismo Responsável do MTur:<br>**90% de lotação**<br><br>Estabelecimentos com **até 10 habitações**/ unidades isoladas (chalés, apartamentos isolados e similares, com banheiros exclusivos e refeições independentes e/ou agendadas):<br>**90% de lotação**	Teletrabalho / Presencial restrito / Restaurantes, bares, lanchonetes e espaços coletivos de alimentação: conforme protocolo de "Restaurantes e Lanchonetes" e Portaria SES nº 319 /	Teleatendimento / Presencial restrito / Equipamentos, espreguiçadeiras, brinquedos infantis: distanciamento mínimo de 4m e higienização constante com álcool 70% ou solução sanitizante similar / Área de piscinas e águas, saunas, academias, quadras etc.: conforme protocolo de "Serviços de educação física (academias, centros de treinamento, estúdios e similares)", "Serviços de educação física em piscina (aberta ou fechada)" e Portaria SES nº 582 e alterações / Eventos: conforme protocolos de "Eventos sociais e de entretenimento em ambiente aberto ou fechado" e Portaria SES nº 617	X	X		Portaria SES nº 319<br><br>Portaria SES nº 582<br><br>Portaria SES nº 617

BANDEIRA AMARELA - Alojamento e Alimentação

// Atividade				// Critérios específicos de funcionamento (conforme bandeira)			// Protocolos obrigatório (todas as bandeiras)	// Protocolos variáveis (recomendados)		// Restrições adicionais
Grupo	CNAE (2 díg.)	Tipo	Subtipos	Teto de Operação (percentual máx. de trabalhadores presentes no turno, ao mesmo tempo, respeitando o teto de ocupação do espaço físico - máx. pessoas)	Modo de Operação (forma de operação, respeitando o teto de operação e o teto de ocupação do espaço físico - máx. pessoas) Trabalhadores	Atendimento	Informativo visível (operação e ocupação) Máscara / EPIs, Distanciamento, Teto de ocupação, Higienização, Proteção de grupo de risco, Afastamento de casos, Cuidados no atendimento ao	Monitora- mento de tempera- tura	Testagem dos trabalha- dores	Normas obrigatórias específicas à atividade https://coronavirus.rs.gov.br/portarias-da-ses
Alojamento e Alimentação	56	Alojamento	Hotéis e similares (em beira de estradas e rodovias)	100% quartos	Teletrabalho / Presencial restrito / Restaurantes, bares, lanchonetes e espaços coletivos de alimentação: conforme protocolo de "Restaurantes e Lanchonetes" e Portaria SES nº 319 /	Teleatendimento / Presencial restrito / Equipamentos, espreguiçadeiras, brinquedos infantis: distanciamento mínimo de 4m e higienização constante com álcool 70% ou solução sanitizante similar / Área de piscinas e águas, saunas, academias, quadras etc.: conforme protocolo de "Serviços de educação física (academias, centros de treinamento, estúdios e similares)", "Serviços de educação física em piscina (aberta ou fechada)" e Portaria SES nº 582 e alterações / Eventos: conforme protocolos de "Eventos sociais e de entretenimento em ambiente aberto ou fechado" e Portaria SES nº 617	X	X		Portaria SES nº 319<br>Portaria SES nº 582<br>Portaria SES nº 617

DISTANCIAMENTO CONTROLADO
GOV RS NOVAS FAÇANHAS

BANDEIRA LARANJA - Alojamento e Alimentação

// Atividade				// Critérios específicos de funcionamento (conforme bandeira)			// Protocolos obrigatório (todas as bandeiras)	// Protocolos variáveis (recomendados)		// Restrições adicionais
Grupo	CNAE (2 díg.)	Tipo	Subtipos	Teto de Operação (percentual máx. de trabalhadores presentes no turno, ao mesmo tempo, respeitando o teto de ocupação do espaço físico - máx. pessoas)	Modo de Operação (forma de operação, respeitando o teto de operação e o teto de ocupação do espaço físico - máx. pessoas)<br>Trabalhadores	Atendimento	Informativo visível (operação e ocupação) Máscara / EPIs, Distanciamento, Teto de ocupação, Higienização, Proteção de grupo de risco, Afastamento de casos, Cuidados no atendimento ao	Monitora- mento de tempera- tura	Testagem dos trabalha- dores	Normas obrigatórias específicas à atividade https://coronavirus.rs.gov.br/portarias-da-ses
Alojamento e Alimentação	56	Alimentação	Restaurantes a la carte, prato feito e buffet sem autosserviço	50% trabalhadores<br>50% lotação	Teletrabalho /<br>Presencial restrito	Presencial restrito /<br>Telentrega /<br>Pegue e Leve /<br>Drive-thru	X			Portaria SES nº 319
Alojamento e Alimentação	56	Alimentação	Restaurantes a la carte, prato feito e buffet sem autosserviço (em beira de estradas e rodovias)	50% trabalhadores<br>50% lotação	Teletrabalho /<br>Presencial restrito	Presencial restrito /<br>Telentrega /<br>Pegue e Leve /<br>Drive-thru	X			Portaria SES nº 319
Alojamento e Alimentação	56	Alimentação	Restaurantes de autosserviço (self-service)	50% trabalhadores<br>50% lotação	Teletrabalho /<br>Presencial restrito /<br>Funcionário(a) orientando no início da fila para o correto atendimento dos protocolos (máscara, álcool gel e distanciamento na fila) /<br>Protetor salivar nos buffets /<br>Higienização e troca constante dos talheres e pegadores do buffet /<br>Talheres embalados individualmente /<br>Demais protocolos da Portaria SES nº 319	Telentrega /<br>Pegue e Leve /<br>Drive-thru /<br>Presencial restrito /<br>Utilização obrigatória de máscara ao se servir e ao circular /<br>Permitido retirar a máscara somente para se alimentar, sentado às mesas /<br>Distanciamento mínimo de 1m entre pessoas na fila do buffet, com marcação no chão /<br>Acesso com entrada e sentido único no buffet, com funcionário(a) aplicando e orientando o correto uso do álcool em gel /<br>Uso obrigatório de álcool em gel 70% em fricção imediatamente antes de se servir no buffet /<br>Utilização de prato limpo a cada vez que cliente se servir /<br>Reforço no distanciamento mínimo de 2m entre as mesas	X			Portaria SES nº 319
Alojamento e Alimentação	56	Alimentação	Lanchonetes, lancherias e bares	50% trabalhadores<br>50% lotação	Teletrabalho /<br>Presencial restrito	Presencial restrito /<br>Telentrega /<br>Pegue e Leve /<br>Drive-thru	X			Portaria SES nº 319
Alojamento e Alimentação	56	Alojamento	Hotéis e similares (geral)	Estabelecimentos sem o Selo Turismo Responsável do MTur:<br>**60% de lotação**<br><br>Estabelecimentos com Selo Turismo Responsável do MTur:<br>**75% de lotação**<br><br>Estabelecimentos com **até 10 habitações**/ unidades isoladas (chalés, apartamentos isolados e similares, com banheiros exclusivos e refeições independentes e/ou agendadas):<br>**75% de lotação**	Teletrabalho /<br>Presencial restrito /<br>Restaurantes, bares, lanchonetes e espaços coletivos de alimentação: conforme protocolo de "Restaurantes e Lanchonetes" e Portaria SES nº 319 /	Teleatendimento /<br>Presencial restrito /<br>Equipamentos, espreguiçadeiras, brinquedos infantis: distanciamento mínimo de 4m e higienização constante com álcool 70% ou solução sanitizante similar /<br>Área de piscinas e águas, saunas, academias, quadras etc.: conforme protocolo de "Serviços de educação física (academias, centros de treinamento, estúdios e similares)", "Serviços de educação física em piscina (aberta ou fechada)" e Portaria SES nº 582 e alterações /<br>Eventos: conforme protocolos de "Eventos sociais e de entretenimento em ambiente aberto ou fechado" e Portaria SES nº 617	X	X		Portaria SES nº 319<br><br>Portaria SES nº 582<br><br>Portaria SES nº 617

DISTANCIAMENTO CONTROLADO

BANDEIRA LARANJA - Alojamento e Alimentação

// Atividade				// Critérios específicos de funcionamento (conforme bandeira)			// Protocolos obrigatório (todas as bandeiras)	// Protocolos variáveis (recomendados)		// Restrições adicionais
Grupo	CNAE (2 díg.)	Tipo	Subtipos	**Teto de Operação** (percentual máx. de trabalhadores presentes no turno, ao mesmo tempo, respeitando o teto de ocupação do espaço físico - máx. pessoas)	**Modo de Operação** (forma de operação, respeitando o teto de operação e o teto de ocupação do espaço físico - máx. pessoas)<br>Trabalhadores	Atendimento	Informativo visível (operação e ocupação)<br>Máscara / EPIs,<br>Distanciamento,<br>Teto de ocupação,<br>Higienização,<br>Proteção de grupo de risco,<br>Afastamento de casos,<br>Cuidados no atendimento ao	Monitora- mento de tempera- tura	Testagem dos trabalha- dores	Normas obrigatórias específicas à atividade<br>https://coronavirus.rs.gov.br/portarias-da-ses
Alojamento e Alimentação	56	Alojamento	Hotéis e similares (em beira de estradas e rodovias)	100% quartos	Teletrabalho /<br>Presencial restrito /<br>Restaurantes, bares, lanchonetes e espaços coletivos de alimentação: conforme protocolo de "Restaurantes e Lanchonetes" e Portaria SES nº 319 /	Teleatendimento /<br>Presencial restrito /<br>Equipamentos, espreguiçadeiras, brinquedos infantis: distanciamento mínimo de 4m e higienização constante com álcool 70% ou solução sanitizante similar /<br>Área de piscinas e águas, saunas, academias, quadras etc.: conforme protocolo de "Serviços de educação física (academias, centros de treinamento, estúdios e similares)", "Serviços de educação física em piscina (aberta ou fechada)" e Portaria SES nº 582 e alterações /<br>Eventos: conforme protocolos de "Eventos sociais e de entretenimento em ambiente aberto ou fechado" e Portaria SES nº 617	X	X		Portaria SES nº 319<br>Portaria SES nº 582<br>Portaria SES nº 617

BANDEIRA VERMELHA - Alojamento e Alimentação

// Atividade				// Critérios específicos de funcionamento (conforme bandeira)			// Protocolos obrigatório (todas as bandeiras)	// Protocolos variáveis (recomendados)		// Restrições adicionais
Grupo	CNAE (2 díg.)	Tipo	Subtipos	Teto de Operação (percentual máx. de trabalhadores presentes no turno, ao mesmo tempo, respeitando o teto de ocupação do espaço físico - máx. pessoas)	Modo de Operação (forma de operação, respeitando o teto de operação e o teto de ocupação do espaço físico - máx. pessoas)<br>Trabalhadores	Atendimento	Informativo visível (operação e ocupação) Máscara / EPIs, Distanciamento, Teto de ocupação, Higienização, Proteção de grupo de risco, Afastamento de casos, Cuidados no atendimento ao público, Atendimento do grupos de risco	Monitora- mento de tempera- tura	Testagem dos trabalha- dores	Normas obrigatórias específicas à atividade https://coronavirus.rs.gov.br/portarias-da-ses
Alojamento e Alimentação	56	Alimentação	Restaurantes a la carte, prato feito e buffet sem autosserviço	50% trabalhadores<br>25% lotação	Teletrabalho /<br>Presencial restrito /<br>Vedado música ao vivo ou mecânica alta, que prejudique a comunicação entre clientes /<br>Ventilação cruzada (janelas e portas abertas)	Presencial restrito (com ingresso até no máximo 22 horas e encerramento 23h) /<br>Grupos de no máximo 6 pessoas por mesa /<br>Distanciamento de 2m entre mesas /<br>Apenas clientes sentados em mesas, sem permanência em pé /<br>Comércio eletrônico, Telentrega, Drive-thru, Pegue e Leve (sem restrição de horário)	X	X		Portaria SES nº 319
Alojamento e Alimentação	56	Alimentação	Restaurantes a la carte, prato feito e buffet sem autosserviço (em beira de estradas e rodovias)	50% trabalhadores<br>50% lotação	Teletrabalho /<br>Presencial restrito /<br>Vedado música ao vivo ou mecânica alta, que prejudique a comunicação entre clientes /<br>Ventilação cruzada (janelas e portas abertas)	Presencial restrito /<br>Grupos de no máximo 6 pessoas por mesa /<br>Distanciamento de 2m entre mesas /<br>Apenas clientes sentados em mesas, sem permanência em pé /<br>Comércio eletrônico, Telentrega, Drive-thru, Pegue e Leve	X	X		Portaria SES nº 319
Alojamento e Alimentação	56	Alimentação	Restaurantes de autosserviço (self-service)	Fechado						
Alojamento e Alimentação	56	Alimentação	Lanchonetes, lancherias e bares	50% trabalhadores<br>25% lotação	Teletrabalho /<br>Presencial restrito /<br>Vedado música ao vivo ou mecânica alta, que prejudique a comunicação entre clientes /<br>Ventilação cruzada (janelas e portas abertas)	Presencial restrito (com ingresso até no máximo 22 horas e encerramento 23h) /<br>Grupos de no máximo 6 pessoas por mesa /<br>Distanciamento de 2m entre mesas /<br>Apenas clientes sentados em mesas, sem permanência em pé /<br>Comércio eletrônico, Telentrega, Drive-thru, Pegue e Leve (sem restrição de horário)	X	X		Portaria SES nº 319
Alojamento e Alimentação	56	Alojamento	Hotéis e similares (geral)	Estabelecimentos sem o Selo Turismo Responsável do MTur: **40% de lotação**<br><br>Estabelecimentos com Selo Turismo Responsável do MTur: **60% de lotação**<br><br>Estabelecimentos com **até 10 habitações**/ unidades isoladas (chalés, apartamentos isolados e similares, com banheiros exclusivos e refeições independentes e/ou agendadas): **60% de lotação**	Teletrabalho /<br>Presencial restrito /<br>Restaurantes, bares, lanchonetes e espaços coletivos de alimentação: conforme protocolo de "Restaurantes e Lanchonetes" e Portaria SES nº 319 /	Teleatendimento /<br>Presencial restrito /<br>Fechamento de áreas comuns como "Equipamentos, espreguiçadeiras, brinquedos infantis", "Área de piscinas e águas, saunas, academias, quadras etc., "Eventos sociais e de entretenimento" /	X	X		Portaria SES nº 319<br><br>Portaria SES nº 582<br><br>Portaria SES nº 617

BANDEIRA VERMELHA - Alojamento e Alimentação

// Atividade				// Critérios específicos de funcionamento (conforme bandeira)			// Protocolos obrigatório (todas as bandeiras)	// Protocolos variáveis (recomendados)		// Restrições adicionais
Grupo	CNAE (2 díg.)	Tipo	Subtipos	Teto de Operação (percentual máx. de trabalhadores presentes no turno, ao mesmo tempo, respeitando o teto de ocupação do espaço físico - máx. pessoas)	Modo de Operação (forma de operação, respeitando o teto de operação e o teto de ocupação do espaço físico - máx. pessoas) Trabalhadores	Atendimento	Informativo visível (operação e ocupação) Máscara / EPIs, Distanciamento, Teto de ocupação, Higienização, Proteção de grupo de risco, Afastamento de casos, Cuidados no atendimento ao público, Atendimento do grupos de risco	Monitora- mento de tempera- tura	Testagem dos trabalha- dores	Normas obrigatórias específicas à atividade https://coronavirus.rs.gov.br/portarias-da-ses
Alojamento e Alimentação	56	Alojamento	Hotéis e similares (em beira de estradas e rodovias)	75% quartos	Teletrabalho / Presencial restrito / Restaurantes, bares, lanchonetes e espaços coletivos de alimentação: conforme protocolo de "Restaurantes" e "Lanchonetes" e Portaria SES nº 319 /	Teleatendimento / Presencial restrito / Fechamento de áreas comuns como "Equipamentos, espreguiçadeiras, brinquedos infantis", "Área de piscinas e águas, saunas, academias, quadras etc., "Eventos sociais e de entretenimento" /	X	X		Portaria SES nº 319<br>Portaria SES nº 582<br>Portaria SES nº 617

DISTANCIAMENTO CONTROLADO
GOV RS
NOVAS FAÇANHAS

BANDEIRA PRETA - Alojamento e Alimentação

// Atividade				// Critérios específicos de funcionamento (conforme bandeira)			// Protocolos obrigatório (todas as bandeiras)	// Protocolos variáveis (recomendados)		// Restrições adicionais
Grupo	CNAE (2 díg.)	Tipo	Subtipos	Teto de Operação (percentual máx. de trabalhadores presentes no turno, ao mesmo tempo, respeitando o teto de ocupação do espaço físico - máx. pessoas)	Modo de Operação (forma de operação, respeitando o teto de operação e o teto de ocupação do espaço físico - máx. pessoas)<br>Trabalhadores	Atendimento	Informativo visível (operação e ocupação)<br>Máscara / EPIs,<br>Distanciamento,<br>Teto de ocupação,<br>Higienização,<br>Proteção de grupo de risco,<br>Afastamento de casos,<br>Cuidados no atendimento ao público,<br>Atendimento do grupos de risco	Monitora-mento de tempera- tura	Testagem dos trabalha- dores	Normas obrigatórias específicas à atividade<br>https://coronavirus.rs.gov.br/portarias-da-ses
Alojamento e Alimentação	56	Alimentação	Restaurantes a la carte, prato feito e buffet sem autosserviço	25% trabalhadores	Teletrabalho / Presencial restrito	(exclusivo) Telentrega / Pegue e Leve / Drive-thru	X	X		Portaria SES nº 319
Alojamento e Alimentação	56	Alimentação	Restaurantes a la carte, prato feito e buffet sem autosserviço (em beira de estradas e rodovias)	25% trabalhadores<br>25% lotação	Teletrabalho / Presencial restrito	Presencial restrito Telentrega / Pegue e Leve / Drive-thru	X	X		Portaria SES nº 319
Alojamento e Alimentação	56	Alimentação	Restaurantes de autosserviço (self-service)	Fechado						
Alojamento e Alimentação	56	Alimentação	Lanchonetes, lancherias e bares	25% trabalhadores	Teletrabalho / Presencial restrito	(exclusivo) Telentrega / Pegue e Leve / Drive-thru	X	X		Portaria SES nº 319
Alojamento e Alimentação	56	Alojamento	Hotéis e similares (geral)	30% quartos	Teletrabalho / Presencial restrito / Restaurantes, bares, lanchonetes e espaços coletivos de alimentação: conforme protocolo de "Restaurantes e Lanchonetes" e Portaria SES nº 319 /	Teleatendimento / Presencial restrito / Fechamento de áreas comuns como "Equipamentos, espreguiçadeiras, brinquedos infantis", "Área de piscinas e águas, saunas, academias, quadras etc., "Eventos sociais e de entretenimento" /	X	X		Portaria SES nº 319<br>Portaria SES nº 582<br>Portaria SES nº 617
Alojamento e Alimentação	56	Alojamento	Hotéis e similares (em beira de estradas e rodovias)	75% quartos	Teletrabalho / Presencial restrito / Restaurantes, bares, lanchonetes e espaços coletivos de alimentação: conforme protocolo de "Restaurantes" e "Lanchonetes" e Portaria SES nº 319 /	Teleatendimento / Presencial restrito / Fechamento de áreas comuns como "Equipamentos, espreguiçadeiras, brinquedos infantis", "Área de piscinas e águas, saunas, academias, quadras etc., "Eventos sociais e de entretenimento" /	X	X		Portaria SES nº 319<br>Portaria SES nº 582<br>Portaria SES nº 617

MODELO DE DISTANCIAMENTO
CONTROLADO DO RS

Comércio

Comitê de DADOS
COVID-19 CORONAVÍRUS

GOV RS
NOVAS FAÇANHAS
NO PLANEJAMENTO, GOVERNANÇA E GESTÃO NA SAÚDE

BANDEIRA AMARELA - Comércio

// Atividade				// Critérios específicos de funcionamento (conforme bandeira)			// Protocolos obrigatório (todas as bandeiras)	// Protocolos variáveis (recomendados)		// Restrições adicionais
Grupo	CNAE (2 díg.)	Tipo	Subtipos	**Teto de Operação**<br>Determina o percentual máximo de trabalhadores/público externo presentes no mesmo turno, ao mesmo tempo.<br><br>Deve respeitar ao nº máximo de pessoas no espaço físico, considerando o distanciamento interpessoal mínimo obrigatório (teto de ocupação).	**Modo de Operação**<br>Forma de operação da atividade, respeitando ao teto de operação, ao teto de ocupação do espaço físico e aos protocolos obrigatórios (ao lado).<br><br>**Trabalhadores**	**Atendimento**	**Decreto nº 55.2540:**<br>- Máscara / EPIs,<br>- Distanciamento,<br>- Teto de ocupação,<br>- Higienização,<br>- Proteção de grupo de risco,<br>- Afastamento de casos,<br>- Cuidados com o público,<br>- Atendimento do grupos de risco<br>- Informativo visível (operação, ocupação e cuidados)	**Monitora-mento de tempera- tura**	**Testagem dos trabalha-dores**	Conteúdo completo das normas obrigatórias específicas à atividade:<br>coronavirus.rs.gov.br/portarias-da-ses
Comércio	45	Comércio de Veículos	Comércio de Veículos (rua)	Lotação (trabalhadores + clientes):<br>1 pessoa, com máscara, para cada 2m² de área útil de circulação, respeitando limite do PPCI	Teletrabalho / Presencial restrito	Teleatendimento / Presencial restrito	X			Portaria SES nº 376
Comércio	45	Comércio de Veículos	Manutenção e Reparação de Veículos Automotores (rua)	Lotação (trabalhadores + clientes):<br>1 pessoa, com máscara, para cada 2m² de área útil de circulação, respeitando limite do PPCI	Teletrabalho / Presencial restrito	Teleatendimento / Presencial restrito	X			Portaria SES nº 376
Comércio	46	Comércio Atacadista	Comércio Atacadista - Não essencial	Lotação (trabalhadores + clientes):<br>1 pessoa, com máscara, para cada 2m² de área útil de circulação, respeitando limite do PPCI	Teletrabalho / Presencial restrito	Presencial restrito / Telentrega / Pegue e Leve / Drive-thru	X			Portaria SES nº 376
Comércio	46	Comércio Atacadista	Comércio Atacadista - Itens Essenciais	Lotação (trabalhadores + clientes):<br>1 pessoa, com máscara, para cada 2m² de área útil de circulação, respeitando limite do PPCI	Teletrabalho / Presencial restrito	Presencial restrito / Telentrega / Pegue e Leve / Drive-thru	X			Portaria SES nº 376
Comércio	47	Comércio Varejista	Comércio Varejista - Não essencial (rua)	Lotação (trabalhadores + clientes):<br>1 pessoa, com máscara, para cada 2m² de área útil de circulação, respeitando limite do PPCI	Teletrabalho / Presencial restrito	Presencial restrito / Telentrega / Pegue e Leve / Drive-thru	X			Portaria SES nº 376
Comércio	47	Comércio Varejista	Comércio Varejista - Não essencial (centro comercial e shopping)	Lotação (trabalhadores + clientes):<br>1 pessoa, com máscara, para cada 2m² de área útil de circulação, respeitando limite do PPCI	Teletrabalho / Presencial restrito	Presencial restrito / Telentrega / Pegue e Leve / Drive-thru	X	X		Portaria SES nº 303 e nº 406

DISTANCIAMENTO CONTROLADO
gov RS
NOVAS FAÇANHAS

BANDEIRA AMARELA - Comércio

// Atividade				// Critérios específicos de funcionamento (conforme bandeira)			// Protocolos obrigatório (todas as bandeiras)	// Protocolos variáveis (recomendados)		// Restrições adicionais
Grupo	CNAE (2 díg.)	Tipo	Subtipos	**Teto de Operação**<br>Determina o percentual máximo de trabalhadores/público externo presentes no mesmo turno, ao mesmo tempo.<br><br>Deve respeitar ao nº máximo de pessoas no espaço físico, considerando o distanciamento interpessoal mínimo obrigatório (teto de ocupação).	**Modo de Operação**<br>Forma de operação da atividade, respeitando ao teto de operação, ao teto de ocupação do espaço físico e aos protocolos obrigatórios (ao lado).<br><br>Trabalhadores	Atendimento	**Decreto nº 55.2540:**<br>- Máscara / EPIs,<br>- Distanciamento,<br>- Teto de ocupação,<br>- Higienização,<br>- Proteção de grupo de risco,<br>- Afastamento de casos,<br>- Cuidados com o público,<br>- Atendimento do grupos de risco<br>- Informativo visível (operação, ocupação e cuidados)	**Monitora-mento de tempera- tura**	**Testagem dos trabalha-dores**	Conteúdo completo das normas obrigatórias específicas à atividade: coronavirus.rs.gov.br/portarias-da-ses
Comércio	47	Comércio Varejista	Comércio Varejista - Itens Essenciais (rua)	Lotação (trabalhadores + clientes):<br>1 pessoa, com máscara, para cada 2m² de área útil de circulação, respeitando limite do PPCI	Teletrabalho / Presencial restrito	Presencial restrito / Telentrega / Pegue e Leve / Drive-thru	X			Portaria SES nº 376
Comércio	46	Comércio Varejista	Comércio Varejista - Itens Essenciais (centro comercial e shopping)	Lotação (trabalhadores + clientes):<br>1 pessoa, com máscara, para cada 2m² de área útil de circulação, respeitando limite do PPCI	Teletrabalho / Presencial restrito	Presencial restrito / Telentrega / Pegue e Leve / Drive-thru	X	X		Portaria SES nº 303 e nº 406
Comércio	47	Comércio Varejista	Comércio Varejista de Produtos Alimentícios (mercados, açougues, fruteiras, padarias e similares)	Lotação (trabalhadores + clientes):<br>1 pessoa, com máscara, para cada 2m² de área útil de circulação, respeitando limite do PPCI	Teletrabalho / Presencial restrito	Presencial restrito / Telentrega / Pegue e Leve / Drive-thru	X			Portaria SES nº 376
Comércio	47	Comércio Varejista	Comércio de Combustíveis para Veículos Automotores	Lotação (trabalhadores + clientes):<br>1 pessoa, com máscara, para cada 2m² de área útil de circulação, respeitando limite do PPCI	Teletrabalho / Presencial restrito	Presencial restrito (vedada aglomeração)	X			Portaria SES nº 376

BANDEIRA LARANJA - Comércio

// Atividade				// Critérios específicos de funcionamento (conforme bandeira)			// Protocolos obrigatório (todas as bandeiras)	// Protocolos variáveis (recomendados)		// Restrições adicionais
Grupo	CNAE (2 díg.)	Tipo	Subtipos	**Teto de Operação**<br>Determina o percentual máximo de trabalhadores/público externo presentes no mesmo turno, ao mesmo tempo.<br><br>Deve respeitar ao nº máximo de pessoas no espaço físico, considerando o distanciamento interpessoal mínimo obrigatório (teto de ocupação).	**Modo de Operação**<br>Forma de operação da atividade, respeitando ao teto de operação, ao teto de ocupação do espaço físico e aos protocolos obrigatórios (ao lado).<br><br>Trabalhadores	Atendimento	**Decreto nº 55.2540:**<br>- Máscara / EPIs,<br>- Distanciamento,<br>- Teto de ocupação,<br>- Higienização,<br>- Proteção de grupo de risco,<br>- Afastamento de casos,<br>- Cuidados com o público,<br>- Atendimento do grupos de risco<br>- Informativo visível (operação, ocupação e cuidados)	**Monitora-mento de tempera- tura**	**Testagem dos trabalha-dores**	Conteúdo completo das normas obrigatórias específicas à atividade: coronavirus.rs.gov.br/portarias-da-ses
Comércio	45	Comércio de Veículos	Comércio de Veículos (rua)	Lotação (trabalhadores + clientes): 1 pessoa, com máscara, para 4m² de área útil de circulação, respeitando limite do PPCI	Teletrabalho / Presencial restrito	Teleatendimento / Presencial restrito	X			Portaria SES nº 376
Comércio	45	Comércio de Veículos	Manutenção e Reparação de Veículos Automotores (rua)	Lotação (trabalhadores + clientes): 1 pessoa, com máscara, para 4m² de área útil de circulação, respeitando limite do PPCI	Teletrabalho / Presencial restrito	Teleatendimento / Presencial restrito	X			Portaria SES nº 376
Comércio	46	Comércio Atacadista	Comércio Atacadista - Não essencial	Lotação (trabalhadores + clientes): 1 pessoa, com máscara, para 4m² de área útil de circulação, respeitando limite do PPCI	Teletrabalho / Presencial restrito	Presencial restrito / Telentrega / Pegue e Leve / Drive-thru	X			Portaria SES nº 376
Comércio	46	Comércio Atacadista	Comércio Atacadista - Itens Essenciais	Lotação (trabalhadores + clientes): 1 pessoa, com máscara, para 4m² de área útil de circulação, respeitando limite do PPCI	Teletrabalho / Presencial restrito	Presencial restrito / Telentrega / Pegue e Leve / Drive-thru	X			Portaria SES nº 376
Comércio	47	Comércio Varejista	Comércio Varejista - Não essencial (rua)	Lotação (trabalhadores + clientes): 1 pessoa, com máscara, para 4m² de área útil de circulação, respeitando limite do PPCI	Teletrabalho / Presencial restrito	Presencial restrito / Telentrega / Pegue e Leve / Drive-thru	X			Portaria SES nº 376
Comércio	47	Comércio Varejista	Comércio Varejista - Não essencial (centro comercial e shopping)	Lotação (trabalhadores + clientes): 1 pessoa, com máscara, para 4m² de área útil de circulação, respeitando limite do PPCI	Teletrabalho / Presencial restrito	Presencial restrito / Telentrega / Pegue e Leve / Drive-thru	X	X		Portaria SES nº 303 e nº 406
Comércio	47	Comércio Varejista	Comércio Varejista - Itens Essenciais (rua)	Lotação (trabalhadores + clientes): 1 pessoa, com máscara, para 4m² de área útil de circulação, respeitando limite do PPCI	Teletrabalho / Presencial restrito	Presencial restrito / Telentrega / Pegue e Leve / Drive-thru	X			Portaria SES nº 376

DISTANCIAMENTO CONTROLADO
GOV RS
NOVAS FAÇANHAS

BANDEIRA LARANJA - Comércio

// Atividade				// Critérios específicos de funcionamento (conforme bandeira)			// Protocolos obrigatório (todas as bandeiras)	// Protocolos variáveis (recomendados)		// Restrições adicionais
Grupo	CNAE (2 díg.)	Tipo	Subtipos	**Teto de Operação**<br>Determina o percentual máximo de trabalhadores/público externo presentes no mesmo turno, ao mesmo tempo.<br><br>Deve respeitar ao nº máximo de pessoas no espaço físico, considerando o distanciamento interpessoal mínimo obrigatório (teto de ocupação).	**Modo de Operação**<br>Forma de operação da atividade, respeitando ao teto de operação, ao teto de ocupação do espaço físico e aos protocolos obrigatórios (ao lado).<br><br>Trabalhadores	Atendimento	**Decreto nº 55.2540:**<br>- Máscara / EPIs,<br>- Distanciamento,<br>- Teto de ocupação,<br>- Higienização,<br>- Proteção de grupo de risco,<br>- Afastamento de casos,<br>- Cuidados com o público,<br>- Atendimento do grupos de risco<br>- Informativo visível (operação, ocupação e cuidados)	**Monitora-mento de tempera- tura**	**Testagem dos trabalha-dores**	Conteúdo completo das normas obrigatórias específicas à atividade: coronavirus.rs.gov.br/portarias-da-ses
Comércio	46	Comércio Varejista	Comércio Varejista - Itens Essenciais (centro comercial e shopping)	Lotação (trabalhadores + clientes): 1 pessoa, com máscara, para 4m² de área útil de circulação, respeitando limite do PPCI	Teletrabalho / Presencial restrito	Presencial restrito / Telentrega / Pegue e Leve / Drive-thru	X	X		Portaria SES nº 303 e nº 406
Comércio	47	Comércio Varejista	Comércio Varejista de Produtos Alimentícios (mercados, açougues, fruteiras, padarias e similares)	Lotação (trabalhadores + clientes): 1 pessoa, com máscara, para 4m² de área útil de circulação, respeitando limite do PPCI	Teletrabalho / Presencial restrito	Presencial restrito / Telentrega / Pegue e Leve / Drive-thru	X			Portaria SES nº 376
Comércio	47	Comércio Varejista	Comércio de Combustíveis para Veículos Automotores	Lotação (trabalhadores + clientes): 1 pessoa, com máscara, para 4m² de área útil de circulação, respeitando limite do PPCI	Teletrabalho / Presencial restrito	Presencial restrito (vedada aglomeração)	X			Portaria SES nº 376

DISTANCIAMENTO CONTROLADO
GOV RS
NOVAS FAÇANHAS

BANDEIRA VERMELHA - Comércio

// Atividade				// Critérios específicos de funcionamento (conforme bandeira)			// Protocolos obrigatório (todas as bandeiras)	// Protocolos variáveis (recomendados)		// Restrições adicionais
Grupo	CNAE (2 díg.)	Tipo	Subtipos	Teto de Operação<br>Determina o percentual máximo de trabalhadores/público presentes no mesmo turno, ao mesmo tempo.<br><br>Deve respeitar ao nº máximo de pessoas no espaço físico, considerando o distanciamento interpessoal mínimo obrigatório (teto de ocupação).	Modo de Operação<br>Forma de operação da atividade, respeitando ao teto de operação, ao teto de ocupação do espaço físico e aos protocolos obrigatórios (ao lado).<br><br>Trabalhadores	Atendimento	Decreto nº 55.2540:<br>- Máscara / EPIs,<br>- Distanciamento,<br>- Teto de ocupação,<br>- Higienização,<br>- Proteção de grupo de risco,<br>- Afastamento de casos,<br>- Cuidados com o público,<br>- Atendimento do grupos de risco<br>- Informativo visível (operação, ocupação e cuidados)	Monitora- mento de tempera- tura	Testagem dos trabalha- dores	Conteúdo completo das normas obrigatórias específicas à atividade: coronavirus.rs.gov.br/portarias-da-ses
Comércio	45	Comércio de Veículos	Comércio de Veículos (rua)	Lotação (trabalhadores + clientes):<br>1 pessoa, com máscara, para 6m² de área útil de circulação, respeitando limite do PPCI	Teletrabalho /<br>Presencial restrito /<br>Respeito ao teto de ocupação e ao distanciamento /<br>Ventilação cruzada (janelas e portas abertas) /	Presencial restrito (com ingresso até no máximo 22 horas e encerramento 23h) /<br>Uso obrigatório e correto de máscara, cobrindo boca e nariz /<br>Horário preferencial para grupo de risco /<br><br>Comércio eletrônico /<br>Telentrega /<br>Drive-thru /<br>Pegue e Leve	X	X		Portaria SES nº 376
Comércio	45	Comércio de Veículos	Manutenção e Reparação de Veículos Automotores (rua)	Lotação (trabalhadores + clientes):<br>1 pessoa, com máscara, para 6m² de área útil de circulação, respeitando limite do PPCI	Teletrabalho /<br>Presencial restrito	Teleatendimento /<br>Presencial restrito	X	X		Portaria SES nº 376
Comércio	46	Comércio Atacadista	Comércio Atacadista - Não essencial	Lotação (trabalhadores + clientes):<br>1 pessoa, com máscara, para 6m² de área útil de circulação, respeitando limite do PPCI	Teletrabalho /<br>Presencial restrito /<br>Respeito ao teto de ocupação e ao distanciamento /<br>Ventilação cruzada (janelas e portas abertas) /	Presencial restrito (com ingresso até no máximo 22 horas e encerramento 23h) /<br>Uso obrigatório e correto de máscara, cobrindo boca e nariz /<br>Horário preferencial para grupo de risco /<br><br>Comércio eletrônico /<br>Telentrega /<br>Drive-thru /<br>Pegue e Leve	X	X		Portaria SES nº 376
Comércio	46	Comércio Atacadista	Comércio Atacadista - Itens Essenciais	Lotação (trabalhadores + clientes):<br>1 pessoa, com máscara, para 6m² de área útil de circulação, respeitando limite do PPCI	Teletrabalho /<br>Presencial restrito	Presencial restrito /<br>Telentrega /<br>Pegue e Leve /<br>Drive-thru	X	X		Portaria SES nº 376
Comércio	47	Comércio Varejista	Comércio Varejista - Não essencial (rua)	Lotação (trabalhadores + clientes):<br>1 pessoa, com máscara, para 6m² de área útil de circulação, respeitando limite do PPCI	Teletrabalho /<br>Presencial restrito /<br>Respeito ao teto de ocupação e ao distanciamento /<br>Ventilação cruzada (janelas e portas abertas) /	Presencial restrito (com ingresso até no máximo 22 horas e encerramento 23h) /<br>Uso obrigatório e correto de máscara, cobrindo boca e nariz /<br>Horário preferencial para grupo de risco /<br><br>Comércio eletrônico /<br>Telentrega /<br>Drive-thru /<br>Pegue e Leve	X	X		Portaria SES nº 376
Comércio	47	Comércio Varejista	Comércio Varejista - Não essencial (centro comercial e shopping)	Lotação (trabalhadores + clientes):<br>1 pessoa, com máscara, para 6m² de área útil de circulação, respeitando limite do PPCI	Teletrabalho /<br>Presencial restrito /<br>Respeito ao teto de ocupação e ao distanciamento /<br>Ventilação cruzada (janelas e portas abertas) /	Presencial restrito (com ingresso até no máximo 22 horas e encerramento 23h) /<br>Uso obrigatório e correto de máscara, cobrindo boca e nariz /<br>Horário preferencial para grupo de risco /<br><br>Comércio eletrônico /<br>Telentrega /<br>Drive-thru /<br>Pegue e Leve	X	X		Portaria SES nº 303 e nº 406

BANDEIRA VERMELHA - Comércio

// Atividade				// Critérios específicos de funcionamento (conforme bandeira)			// Protocolos obrigatório (todas as bandeiras)	// Protocolos variáveis (recomendados)		// Restrições adicionais
Grupo	CNAE (2 díg.)	Tipo	Subtipos	**Teto de Operação**<br>Determina o percentual máximo de trabalhadores/público presentes no mesmo turno, ao mesmo tempo.<br><br>Deve respeitar ao nº máximo de pessoas no espaço físico, considerando o distanciamento interpessoal mínimo obrigatório (teto de ocupação).	**Modo de Operação**<br>Forma de operação da atividade, respeitando ao teto de operação, ao teto de ocupação do espaço físico e aos protocolos obrigatórios (ao lado).<br><br>**Trabalhadores**	**Atendimento**	**Decreto nº 55.2540:**<br>- Máscara / EPIs,<br>- Distanciamento,<br>- Teto de ocupação,<br>- Higienização,<br>- Proteção de grupo de risco,<br>- Afastamento de casos,<br>- Cuidados com o público,<br>- Atendimento do grupos de risco<br>- Informativo visível (operação, ocupação e cuidados)	**Monitora- mento de tempera- tura**	**Testagem dos trabalha- dores**	Conteúdo completo das normas obrigatórias específicas à atividade: coronavirus.rs.gov.br/portarias-da-ses
Comércio	47	Comércio Varejista	Comércio Varejista - Itens Essenciais (rua)	Lotação (trabalhadores + clientes):<br>1 pessoa, com máscara, para 6m² de área útil de circulação, respeitando limite do PPCI	Teletrabalho / Presencial restrito	Presencial restrito / Telentrega / Pegue e Leve / Drive-thru	X	X		Portaria SES nº 376
Comércio	46	Comércio Varejista	Comércio Varejista - Itens Essenciais (centro comercial e shopping)	Lotação (trabalhadores + clientes):<br>1 pessoa, com máscara, para 6m² de área útil de circulação, respeitando limite do PPCI	Teletrabalho / Presencial restrito	Presencial restrito / Telentrega / Pegue e Leve / Drive-thru	X	X		Portaria SES nº 303 e nº 406
Comércio	47	Comércio Varejista	Comércio Varejista de Produtos Alimentícios (mercados, açougues, fruteiras, padarias e similares)	Lotação (trabalhadores + clientes):<br>1 pessoa, com máscara, para 6m² de área útil de circulação, respeitando limite do PPCI	Teletrabalho / Presencial restrito	Presencial restrito / Telentrega / Pegue e Leve / Drive-thru	X	X		Portaria SES nº 376
Comércio	47	Comércio Varejista	Comércio de Combustíveis para Veículos Automotores	Lotação (trabalhadores + clientes):<br>1 pessoa, com máscara, para 6m² de área útil de circulação, respeitando limite do PPCI	Teletrabalho / Presencial restrito	Presencial restrito (vedada aglomeração e vedado consumo de alimentos e bebidas)	X	X		Portaria SES nº 376

DISTANCIAMENTO CONTROLADO RS NOVAS FAÇANHAS

BANDEIRA PRETA - Comércio

// Atividade				// Critérios específicos de funcionamento (conforme bandeira)			// Protocolos obrigatório (todas as bandeiras)	// Protocolos variáveis (recomendados)		// Restrições adicionais
Grupo	CNAE (2 díg.)	Tipo	Subtipos	**Teto de Operação**<br>Determina o percentual máximo de trabalhadores/público presentes no mesmo turno, ao mesmo tempo.<br><br>Deve respeitar ao nº máximo de pessoas no espaço físico, considerando o distanciamento interpessoal mínimo obrigatório (teto de ocupação).	**Modo de Operação**<br>Forma de operação da atividade, respeitando ao teto de operação, ao teto de ocupação do espaço físico e aos protocolos obrigatórios (ao lado).<br><br>**Trabalhadores**	**Atendimento**	**Decreto nº 55.2540:**<br>- Máscara / EPIs,<br>- Distanciamento,<br>- Teto de ocupação,<br>- Higienização,<br>- Proteção de grupo de risco,<br>- Afastamento de casos,<br>- Cuidados com o público,<br>- Atendimento do grupos de risco<br>- Informativo visível (operação, ocupação e cuidados)	**Monitora- mento de tempera- tura**	**Testagem dos trabalha- dores**	Conteúdo completo das normas obrigatórias específicas à atividade: coronavirus.rs.gov.br/portarias-da-ses
Comércio	45	Comércio de Veículos	Comércio de Veículos (rua)	Fechado						
Comércio	45	Comércio de Veículos	Manutenção e Reparação de Veículos Automotores (rua)	Lotação (trabalhadores + clientes): 1 pessoa, com máscara, para 8m² de área útil de circulação, respeitando limite do PPCI	Teletrabalho / Presencial restrito	Teleatendimento / Presencial restrito	X			Portaria SES nº 376
Comércio	46	Comércio Atacadista	Comércio Atacadista - Não essencial	Fechado						
Comércio	46	Comércio Atacadista	Comércio Atacadista - Itens Essenciais	Lotação (trabalhadores + clientes): 1 pessoa, com máscara, para 8m² de área útil de circulação, respeitando limite do PPCI	Teletrabalho / Presencial restrito	Presencial restrito / Telentrega / Pegue e Leve / Drive-thru	X	X		Portaria SES nº 376
Comércio	47	Comércio Varejista	Comércio Varejista - Não essencial (rua)	Fechado						
Comércio	47	Comércio Varejista	Comércio Varejista - Não essencial (centro comercial e shopping)	Fechado						
Comércio	47	Comércio Varejista	Comércio Varejista - Itens Essenciais (rua)	Lotação (trabalhadores + clientes): 1 pessoa, com máscara, para 8m² de área útil de circulação, respeitando limite do PPCI	Teletrabalho / Presencial restrito	Presencial restrito / Telentrega / Pegue e Leve / Drive-thru	X	X		Portaria SES nº 376
Comércio	46	Comércio Varejista	Comércio Varejista - Itens Essenciais (centro comercial e shopping)	Lotação (trabalhadores + clientes): 1 pessoa, com máscara, para 8m² de área útil de circulação, respeitando limite do PPCI	Teletrabalho / Presencial restrito	Presencial restrito / Telentrega / Pegue e Leve / Drive-thru	X	X		Portaria SES nº 303 e nº 406

BANDEIRA PRETA - Comércio

// Atividade				// Critérios específicos de funcionamento (conforme bandeira)			// Protocolos obrigatório (todas as bandeiras)	// Protocolos variáveis (recomendados)		// Restrições adicionais
Grupo	CNAE (2 díg.)	Tipo	Subtipos	**Teto de Operação** Determina o percentual máximo de trabalhadores/público presentes no mesmo turno, ao mesmo tempo. Deve respeitar ao nº máximo de pessoas no espaço físico, considerando o distanciamento interpessoal mínimo obrigatório (teto de ocupação).	**Modo de Operação** Forma de operação da atividade, respeitando ao teto de operação, ao teto de ocupação do espaço físico e aos protocolos obrigatórios (ao lado). **Trabalhadores**	**Atendimento**	**Decreto nº 55.2540:** - Máscara / EPIs, - Distanciamento, - Teto de ocupação, - Higienização, - Proteção de grupo de risco, - Afastamento de casos, - Cuidados com o público, - Atendimento do grupos de risco - Informativo visível (operação, ocupação e cuidados)	**Monitora- mento de tempera- tura**	**Testagem dos trabalha- dores**	Conteúdo completo das normas obrigatórias específicas à atividade: coronavirus.rs.gov.br/portarias-da-ses
Comércio	47	Comércio Varejista	Comércio Varejista de Produtos Alimentícios (mercados, açougues, fruteiras, padarias e similares)	Lotação (trabalhadores + clientes): 1 pessoa, com máscara, para 8m² de área útil de circulação, respeitando limite do PPCI	Teletrabalho / Presencial restrito	Presencial restrito / Telentrega / Pegue e Leve / Drive-thru	X	X		Portaria SES nº 376
Comércio	47	Comércio Varejista	Comércio de Combustíveis para Veículos Automotores	Lotação (trabalhadores + clientes): 1 pessoa, com máscara, para 8m² de área útil de circulação, respeitando limite do PPCI	Teletrabalho / Presencial restrito	Presencial restrito (vedada aglomeração e vedado consumo de alimentos e bebidas)	X			Portaria SES nº 376

DISTANCIAMENTO CONTROLADO
RS
NOVAS FAÇANHAS

MODELO DE DISTANCIAMENTO
CONTROLADO DO RS

Educação

DISTANCIAMENTO CONTROLADO
GOV RS
NOVAS FAÇANHAS

Comitê de DADOS
COVID-19 CORONAVÍRUS

GOV RS
NOVAS FAÇANHAS
NO PLANEJAMENTO, GOVERNANÇA E GESTÃO NA SAÚDE

BANDEIRA AMARELA - Educação

// Atividade				// Critérios específicos de funcionamento (conforme bandeira)			// Protocolos obrigatório (todas as bandeiras)	// Protocolos variáveis (recomendados)		// Restrições adicionais
Grupo	CNAE (2 díg.)	Tipo	Subtipos	**Teto de Operação**<br>Determina o percentual máximo de trabalhadores/público externo presentes no mesmo turno, ao mesmo tempo.<br><br>Deve respeitar ao nº máximo de pessoas no espaço físico, considerando o distanciamento interpessoal mínimo obrigatório (teto de ocupação).	**Modo de Operação**<br>Forma de operação da atividade, respeitando ao teto de operação, ao teto de ocupação do espaço físico e aos protocolos obrigatórios (ao lado).<br><br>**Trabalhadores**	**Atendimento**	**Decreto nº 55.2540:**<br>- Máscara / EPIs,<br>- Distanciamento,<br>- Teto de ocupação,<br>- Higienização,<br>- Proteção de grupo de risco,<br>- Afastamento de casos,<br>- Cuidados com o público,<br>- Atendimento do grupos de risco<br>- Informativo visível (operação, ocupação e cuidados)	**Monitora-mento de tempera- tura**	**Testagem dos trabalha-dores**	Conteúdo completo das normas obrigatórias específicas à atividade: coronavirus.rs.gov.br/portarias-da-ses
Educação	85	Educação Infantil	Creche e Pré-Escola	Regra Geral:<br>Remoto<br><br>Se permitida atividade presencial:<br>50% alunos por sala de aula	Teletrabalho / Presencial restrito	Regra Geral:<br>Ensino Remoto<br><br>Se permitida atividade presencial:<br>Ensino Remoto /<br>Ensino Híbrido (remoto e/ou presencial) /<br>Presencial Restrito /<br>50% alunos por sala de aula /<br>Distanciamento mínimo /<br>Materiais individuais /<br>Vedado atividades coletivas que envolvam aglomeração ou contato físico	X	X		Portaria SES/SEDUC nº 01/2020, Decreto Estadual nº 55.465 (05/09/2020) e demais normativas.
Educação	85	Ensino Fundamental	Ensino Fundamental - Anos Iniciais	Regra Geral:<br>Remoto<br><br>Se permitida atividade presencial:<br>50% alunos por sala de aula	Teletrabalho / Presencial restrito	Regra Geral:<br>Ensino Remoto<br><br>Se permitida atividade presencial:<br>Ensino Remoto /<br>Ensino Híbrido (remoto e/ou presencial) /<br>Presencial Restrito /<br>50% alunos por sala de aula /<br>Distanciamento mínimo /<br>Materiais individuais /<br>Vedado atividades coletivas que envolvam aglomeração ou contato físico	X	X		Portaria SES/SEDUC nº 01/2020, Decreto Estadual nº 55.465 (05/09/2020) e demais normativas.
Educação	85	Ensino Fundamental	Ensino Fundamental - Anos Finais	Regra Geral:<br>Remoto<br><br>Se permitida atividade presencial:<br>50% alunos por sala de aula	Teletrabalho / Presencial restrito	Regra Geral:<br>Ensino Remoto<br><br>Se permitida atividade presencial:<br>Ensino Remoto /<br>Ensino Híbrido (remoto e/ou presencial) /<br>Presencial Restrito /<br>50% alunos por sala de aula /<br>Distanciamento mínimo /<br>Materiais individuais /<br>Vedado atividades coletivas que envolvam aglomeração ou contato físico	X	X		Portaria SES/SEDUC nº 01/2020, Decreto Estadual nº 55.465 (05/09/2020) e demais normativas.
Educação	85	Ensino Médio	Ensino Médio	Regra Geral:<br>Remoto<br><br>Se permitida atividade presencial:<br>50% alunos por sala de aula	Teletrabalho / Presencial restrito	Regra Geral:<br>Ensino Remoto<br><br>Se permitida atividade presencial:<br>Ensino Remoto /<br>Ensino Híbrido (remoto e/ou presencial) /<br>Presencial Restrito /<br>50% alunos por sala de aula /<br>Distanciamento mínimo /<br>Materiais individuais /<br>Vedado atividades coletivas que envolvam aglomeração ou contato físico	X	X		Portaria SES/SEDUC nº 01/2020, Decreto Estadual nº 55.465 (05/09/2020) e demais normativas.

BANDEIRA AMARELA - Educação

// Atividade				// Critérios específicos de funcionamento (conforme bandeira)			// Protocolos obrigatório (todas as bandeiras)	// Protocolos variáveis (recomendados)		// Restrições adicionais
Grupo	CNAE (2 díg.)	Tipo	Subtipos	**Teto de Operação**<br>Determina o percentual máximo de trabalhadores/público externo presentes no mesmo turno, ao mesmo tempo.<br><br>Deve respeitar ao nº máximo de pessoas no espaço físico, considerando o distanciamento interpessoal mínimo obrigatório (teto de ocupação).	**Modo de Operação**<br>Forma de operação da atividade, respeitando ao teto de operação, ao teto de ocupação do espaço físico e aos protocolos obrigatórios (ao lado).<br><br>**Trabalhadores**	**Atendimento**	**Decreto nº 55.2540:**<br>- Máscara / EPIs,<br>- Distanciamento,<br>- Teto de ocupação,<br>- Higienização,<br>- Proteção de grupo de risco,<br>- Afastamento de casos,<br>- Cuidados com o público,<br>- Atendimento do grupos de risco<br>- Informativo visível (operação, ocupação e cuidados)	**Monitora-mento de tempera- tura**	**Testagem dos trabalha-dores**	Conteúdo completo das normas obrigatórias específicas à atividade: coronavirus.rs.gov.br/portarias-da-ses
Educação	85	Ensino Médio	Ensino Técnico de Nível Médio e Normal	Regra Geral:<br>Remoto<br><br>Se permitida atividade presencial:<br>50% alunos por sala de aula	Teletrabalho / Presencial restrito	Regra Geral:<br>Ensino Remoto<br><br>Se permitida atividade presencial:<br>Ensino Remoto /<br>Ensino Híbrido (remoto e/ou presencial) /<br>Presencial Restrito /<br>50% alunos por sala de aula /<br>Distanciamento mínimo /<br>Materiais individuais /<br>Vedado atividades coletivas que envolvam aglomeração ou contato físico	X	X		Portaria SES/SEDUC nº 01/2020, Decreto Estadual nº 55.465 (05/09/2020) e demais normativas.
Educação	85	Ensino Superior	Graduação (Bacharelado, Licenciatura, Tecnólogo) e Pós-graduação (stricto e latu sensu)	Regra Geral:<br>Remoto<br><br>Se permitida atividade presencial:<br>50% alunos por sala de aula	Teletrabalho / Presencial restrito	Regra Geral:<br>Ensino Remoto<br><br>Se permitida atividade presencial:<br>Ensino Remoto /<br>Ensino Híbrido (remoto e/ou presencial) /<br>Presencial Restrito /<br>50% alunos por sala de aula /<br>Distanciamento mínimo /<br>Materiais individuais /<br>Vedado atividades coletivas que envolvam aglomeração ou contato físico	X	X		Portaria SES/SEDUC nº 01/2020, Decreto Estadual nº 55.465 (05/09/2020) e demais normativas.
Educação	85	Ensino Superior	Ensino Médio Técnico Concomitante e Subseqüente, Ensino Superior e Pós-Graduação<br><br>(*somente atividades práticas essenciais para conclusão de curso da área da saúde*: pesquisa, estágio curricular obrigatório, laboratórios e plantão*)	50% alunos	Teletrabalho / Presencial restrito	Regra Geral:<br>Presencial restrito /<br>Atendimento individualizado sob agendamento /<br>Atividades práticas em pequenos grupos, respeitando teto de ocupação /<br>Material individual<br><br>Se permitida atividade presencial do segmento:<br>Ensino Remoto /<br>Ensino Híbrido (remoto e/ou presencial) /<br>Presencial Restrito /<br>50% alunos por sala de aula /<br>Distanciamento mínimo /<br>Materiais individuais /<br>Vedado atividades coletivas que envolvam aglomeração ou contato físico.	X	X		Portaria SES/SEDUC nº 01/2020, Decreto Estadual nº 55.465 (05/09/2020) e demais normativas.
Educação		Ensino Médio e Superior	Ensino Médio Técnico Subseqüente, Ensino Superior e Pós-Graduação (*somente atividades práticas essenciais para conclusão de curso: pesquisa, estágio curricular obrigatório, laboratórios e plantão*)	50% alunos	Teletrabalho / Presencial restrito	Regra Geral:<br>Presencial restrito /<br>Atendimento individualizado sob agendamento /<br>Atividades práticas em pequenos grupos, respeitando teto de ocupação /<br>Material individual<br><br>Se permitida atividade presencial do segmento:<br>Ensino Remoto /<br>Ensino Híbrido (remoto e/ou presencial) /<br>Presencial Restrito /<br>50% alunos por sala de aula /<br>Distanciamento mínimo /<br>Materiais individuais /<br>Vedado atividades coletivas que envolvam aglomeração ou contato físico	X	X		Portaria SES/SEDUC nº 01/2020, Decreto Estadual nº 55.465 (05/09/2020) e demais normativas.

BANDEIRA AMARELA - Educação

// Atividade				// Critérios específicos de funcionamento (conforme bandeira)			// Protocolos obrigatório (todas as bandeiras)	// Protocolos variáveis (recomendados)		// Restrições adicionais
Grupo	CNAE (2 díg.)	Tipo	Subtipos	**Teto de Operação**<br>Determina o percentual máximo de trabalhadores/público externo presentes no mesmo turno, ao mesmo tempo.<br><br>Deve respeitar ao nº máximo de pessoas no espaço físico, considerando o distanciamento interpessoal mínimo obrigatório (teto de ocupação).	**Modo de Operação**<br>Forma de operação da atividade, respeitando ao teto de operação, ao teto de ocupação do espaço físico e aos protocolos obrigatórios (ao lado).<br><br>**Trabalhadores**	**Atendimento**	**Decreto nº 55.2540:**<br>- Máscara / EPIs,<br>- Distanciamento,<br>- Teto de ocupação,<br>- Higienização,<br>- Proteção de grupo de risco,<br>- Afastamento de casos,<br>- Cuidados com o público,<br>- Atendimento do grupos de risco<br>- Informativo visível (operação, ocupação e cuidados)	**Monitora-mento de tempera- tura**	**Testagem dos trabalha-dores**	Conteúdo completo das normas obrigatórias específicas à atividade: coronavirus.rs.gov.br/portarias-da-ses
Educação	85	Educação - Outros	Atividades de Apoio à Educação	50% trabalhadores	Teletrabalho / Presencial restrito	Teleatendimento / Presencial restrito	X	X		Portaria SES/SEDUC nº 01
Educação	85	Outras Atividades de Ensino	Ensino de Idiomas	50% trabalhadores<br>50% alunos	Teletrabalho / Presencial restrito	Ensino remoto / Atendimento individualizado ou em pequenos grupos, respeitando teto de ocupação / Material individual	X	X		Portaria SES/SEDUC nº 01
Educação	85	Outras Atividades de Ensino	Ensino de Música	50% trabalhadores<br>50% alunos	Teletrabalho / Presencial restrito	Ensino remoto / Atendimento individualizado ou em pequenos grupos, respeitando teto de ocupação / Material individual	X	X		Portaria SES/SEDUC nº 01
Educação	85	Outras Atividades de Ensino	Ensino de Esportes, Dança e Artes Cênicas	50% trabalhadores<br>50% alunos	Teletrabalho / Presencial restrito	Ensino remoto / Atendimento individualizado ou coabitantes / Material individual /<br><br>Ficam permitidas as atividades de esportes coletivos exclusivamente em quadras esportivas, sem público, com intervalo de 1 hora entre os jogos e uso intercalado das quadras, para evitar aglomeração e permitir higienização /<br><br>Ensino e Ensaio de Dança e Artes Cênicas coletivas, sem público, com intervalo de 1 hora entre as atividades, com uso intercalado do espaço/pista, para evitar aglomeração e permitir higienização /<br><br>Restaurantes e Lanchonetes em conformidade com o protocolo específico	X	X		Portaria SES nº 582
Educação	85	Outras Atividades de Ensino	Ensino de Arte e Cultura (outros)	50% trabalhadores<br>50% alunos	Teletrabalho / Presencial restrito	Ensino remoto / Atendimento individualizado ou em pequenos grupos, respeitando teto de ocupação / Material individual	X	X		Portaria SES/SEDUC nº 01
Educação	85	Outras Atividades de Ensino	Formação profissional, formação continuada, cursos preparatórios para concurso, treinamentos e similares	50% trabalhadores<br>50% alunos	Teletrabalho / Presencial restrito	Ensino remoto / Atendimento individualizado ou em pequenos grupos, respeitando teto de ocupação / Material individual	X	X		Portaria SES/SEDUC nº 01

BANDEIRA LARANJA - Educação

// Atividade				// Critérios específicos de funcionamento (conforme bandeira)			// Protocolos obrigatório (todas as bandeiras)	// Protocolos variáveis (recomendados)		// Restrições adicionais
Grupo	CNAE (2 díg.)	Tipo	Subtipos	**Teto de Operação** Determina o percentual máximo de trabalhadores/público externo presentes no mesmo turno, ao mesmo tempo. Deve respeitar ao nº máximo de pessoas no espaço físico, considerando o distanciamento interpessoal mínimo obrigatório (teto de ocupação).	**Modo de Operação** Forma de operação da atividade, respeitando ao teto de operação, ao teto de ocupação do espaço físico e aos protocolos obrigatórios (ao lado). Trabalhadores	Atendimento	**Decreto nº 55.2540:** - Máscara / EPIs, - Distanciamento, - Teto de ocupação, - Higienização, - Proteção de grupo de risco, - Afastamento de casos, - Cuidados com o público, - Atendimento do grupos de risco - Informativo visível (operação, ocupação e cuidados)	**Monitora- mento de tempera- tura**	**Testagem dos trabalha- dores**	Conteúdo completo das normas obrigatórias específicas à atividade: coronavirus.rs.gov.br/portarias-da-ses
Educação	85	Educação Infantil	Creche e Pré-Escola	Regra Geral: Remoto<br><br>Se permitida atividade presencial: 50% alunos por sala de aula	Teletrabalho / Presencial restrito	Regra Geral: Ensino Remoto<br><br>Se permitida atividade presencial: Ensino Remoto / Ensino Híbrido (remoto e/ou presencial) / Presencial Restrito / 50% alunos por sala de aula / Distanciamento mínimo / Materiais individuais / Vedado atividades coletivas que envolvam aglomeração ou contato físico	X	X		Portaria SES/SEDUC nº 01/2020, Decreto Estadual nº 55.465 (05/09/2020) e demais normativas.
Educação	85	Ensino Fundamental	Ensino Fundamental - Anos Iniciais	Regra Geral: Remoto<br><br>Se permitida atividade presencial: 50% alunos por sala de aula	Teletrabalho / Presencial restrito	Regra Geral: Ensino Remoto<br><br>Se permitida atividade presencial: Ensino Remoto / Ensino Híbrido (remoto e/ou presencial) / Presencial Restrito / 50% alunos por sala de aula / Distanciamento mínimo / Materiais individuais / Vedado atividades coletivas que envolvam aglomeração ou contato físico	X	X		Portaria SES/SEDUC nº 01/2020, Decreto Estadual nº 55.465 (05/09/2020) e demais normativas.
Educação	85	Ensino Fundamental	Ensino Fundamental - Anos Finais	Regra Geral: Remoto<br><br>Se permitida atividade presencial: 50% alunos por sala de aula	Teletrabalho / Presencial restrito	Regra Geral: Ensino Remoto<br><br>Se permitida atividade presencial: Ensino Remoto / Ensino Híbrido (remoto e/ou presencial) / Presencial Restrito / 50% alunos por sala de aula / Distanciamento mínimo / Materiais individuais / Vedado atividades coletivas que envolvam aglomeração ou contato físico	X	X		Portaria SES/SEDUC nº 01/2020, Decreto Estadual nº 55.465 (05/09/2020) e demais normativas.
Educação	85	Ensino Médio	Ensino Médio	Regra Geral: Remoto<br><br>Se permitida atividade presencial: 50% alunos por sala de aula	Teletrabalho / Presencial restrito	Regra Geral: Ensino Remoto<br><br>Se permitida atividade presencial: Ensino Remoto / Ensino Híbrido (remoto e/ou presencial) / Presencial Restrito / 50% alunos por sala de aula / Distanciamento mínimo / Materiais individuais / Vedado atividades coletivas que envolvam aglomeração ou contato físico	X	X		Portaria SES/SEDUC nº 01/2020, Decreto Estadual nº 55.465 (05/09/2020) e demais normativas.

BANDEIRA LARANJA - Educação

// Atividade				// Critérios específicos de funcionamento (conforme bandeira)			// Protocolos obrigatório (todas as bandeiras)	// Protocolos variáveis (recomendados)		// Restrições adicionais
Grupo	CNAE (2 díg.)	Tipo	Subtipos	**Teto de Operação**<br>Determina o percentual máximo de trabalhadores/público externo presentes no mesmo turno, ao mesmo tempo.<br><br>Deve respeitar ao nº máximo de pessoas no espaço físico, considerando o distanciamento interpessoal mínimo obrigatório (teto de ocupação).	**Modo de Operação**<br>Forma de operação da atividade, respeitando ao teto de operação, ao teto de ocupação do espaço físico e aos protocolos obrigatórios (ao lado).<br><br>Trabalhadores	Atendimento	**Decreto nº 55.2540:**<br>- Máscara / EPIs,<br>- Distanciamento,<br>- Teto de ocupação,<br>- Higienização,<br>- Proteção de grupo de risco,<br>- Afastamento de casos,<br>- Cuidados com o público,<br>- Atendimento do grupos de risco<br>- Informativo visível (operação, ocupação e cuidados)	**Monitora- mento de tempera- tura**	**Testagem dos trabalha- dores**	Conteúdo completo das normas obrigatórias específicas à atividade: <u>coronavirus.rs.gov.br/portarias-da-ses</u>
Educação	85	Ensino Médio	Ensino Técnico de Nível Médio e Normal	Regra Geral:<br>Remoto<br><br>Se permitida atividade presencial:<br>50% alunos por sala de aula	Teletrabalho /<br>Presencial restrito	Regra Geral:<br>Ensino Remoto<br><br>Se permitida atividade presencial:<br>Ensino Remoto /<br>Ensino Híbrido (remoto e/ou presencial) /<br>Presencial Restrito /<br>50% alunos por sala de aula /<br>Distanciamento mínimo /<br>Materiais individuais /<br>Vedado atividades coletivas que envolvam aglomeração ou contato físico	X	X		Portaria SES/SEDUC nº 01/2020, Decreto Estadual nº 55.465 (05/09/2020) e demais normativas.
Educação	85	Ensino Superior	Graduação (Bacharelado, Licenciatura, Tecnólogo) e Pós-graduação (stricto e latu sensu)	Regra Geral:<br>Remoto<br><br>Se permitida atividade presencial:<br>50% alunos por sala de aula	Teletrabalho /<br>Presencial restrito	Regra Geral:<br>Ensino Remoto<br><br>Se permitida atividade presencial:<br>Ensino Remoto /<br>Ensino Híbrido (remoto e/ou presencial) /<br>Presencial Restrito /<br>50% alunos por sala de aula /<br>Distanciamento mínimo /<br>Materiais individuais /<br>Vedado atividades coletivas que envolvam aglomeração ou contato físico	X	X		Portaria SES/SEDUC nº 01/2020, Decreto Estadual nº 55.465 (05/09/2020) e demais normativas.
Educação	85	Ensino Superior	Ensino Médio Técnico <u>Concomitante</u> e Subseqüente, Ensino Superior e Pós-Graduação<br><br>(*somente atividades práticas essenciais para conclusão de curso da <u>área da saúde*</u>: pesquisa, estágio curricular obrigatório, laboratórios e plantão*)	50% trabalhadores<br>50% alunos	Teletrabalho /<br>Presencial restrito	Regra Geral:<br>Presencial restrito /<br>Atendimento individualizado sob agendamento /<br>Atividades práticas em pequenos grupos, respeitando teto de ocupação /<br>Material individual<br><br>Se permitida atividade presencial do segmento:<br>Ensino Remoto /<br>Ensino Híbrido (remoto e/ou presencial) /<br>Presencial Restrito /<br>50% alunos por sala de aula /<br>Distanciamento mínimo /<br>Materiais individuais /<br>Vedado atividades coletivas que envolvam aglomeração ou contato físico.	X	X		Portaria SES/SEDUC nº 01/2020, Decreto Estadual nº 55.465 (05/09/2020) e demais normativas.
Educação		Ensino Médio e Superior	Ensino Médio Técnico Subseqüente, Ensino Superior e Pós-Graduação (*somente atividades práticas essenciais para conclusão de curso: pesquisa, estágio curricular obrigatório, laboratórios e plantão*)	50% trabalhadores<br>50% alunos	Teletrabalho /<br>Presencial restrito	Regra Geral:<br>Presencial restrito /<br>Atendimento individualizado sob agendamento /<br>Atividades práticas em pequenos grupos, respeitando teto de ocupação /<br>Material individual<br><br>Se permitida atividade presencial do segmento:<br>Ensino Remoto /<br>Ensino Híbrido (remoto e/ou presencial) /<br>Presencial Restrito /<br>50% alunos por sala de aula /<br>Distanciamento mínimo /<br>Materiais individuais /<br>Vedado atividades coletivas que envolvam aglomeração ou contato físico	X	X		Portaria SES/SEDUC nº 01/2020, Decreto Estadual nº 55.465 (05/09/2020) e demais normativas.

BANDEIRA LARANJA - Educação

// Atividade				// Critérios específicos de funcionamento (conforme bandeira)			// Protocolos obrigatório (todas as bandeiras)	// Protocolos variáveis (recomendados)		// Restrições adicionais
Grupo	CNAE (2 díg.)	Tipo	Subtipos	**Teto de Operação**<br>Determina o percentual máximo de trabalhadores/público externo presentes no mesmo turno, ao mesmo tempo.<br><br>Deve respeitar ao nº máximo de pessoas no espaço físico, considerando o distanciamento interpessoal mínimo obrigatório (teto de ocupação).	**Modo de Operação**<br>Forma de operação da atividade, respeitando ao teto de operação, ao teto de ocupação do espaço físico e aos protocolos obrigatórios (ao lado).<br><br>Trabalhadores	Atendimento	**Decreto nº 55.2540:**<br>- Máscara / EPIs,<br>- Distanciamento,<br>- Teto de ocupação,<br>- Higienização,<br>- Proteção de grupo de risco,<br>- Afastamento de casos,<br>- Cuidados com o público,<br>- Atendimento do grupos de risco<br>- Informativo visível (operação, ocupação e cuidados)	**Monitora- mento de tempera- tura**	**Testagem dos trabalha- dores**	Conteúdo completo das normas obrigatórias específicas à atividade: coronavirus.rs.gov.br/portarias-da-ses
Educação	85	Educação - Outros	Atividades de Apoio à Educação	50% trabalhadores	Teletrabalho / Presencial restrito	Teleatendimento / Presencial restrito	X	X		Portaria SES/SEDUC nº 01
Educação	85	Outras Atividades de Ensino	Ensino de Idiomas	50% trabalhadores<br>50% alunos	Teletrabalho / Presencial restrito	Ensino remoto / Atendimento individualizado ou em pequenos grupos, respeitando teto de ocupação / Material individual	X	X		Portaria SES/SEDUC nº 01
Educação	85	Outras Atividades de Ensino	Ensino de Música	50% trabalhadores<br>50% alunos	Teletrabalho / Presencial restrito	Ensino remoto / Atendimento individualizado ou em pequenos grupos, respeitando teto de ocupação / Material individual	X	X		Portaria SES/SEDUC nº 01
Educação	85	Outras Atividades de Ensino	Ensino de Esportes, Dança e Artes Cênicas	50% trabalhadores<br>50% alunos	Teletrabalho / Presencial restrito	Ensino remoto / Atendimento individualizado ou coabitantes / Material individual /<br><br>Ficam permitidas as atividades de esportes coletivos exclusivamente em quadras esportivas, sem público, com intervalo de 1 hora entre os jogos e uso intercalado das quadras, para evitar aglomeração e permitir higienização /<br><br>Ensino e Ensaio de Dança e Artes Cênicas coletivas, sem público, com intervalo de 1 hora entre as atividades, com uso intercalado do espaço/pista, para evitar aglomeração e permitir higienização /<br><br>Restaurantes e Lanchonetes em conformidade com o protocolo específico	X	X		Portaria SES nº 582
Educação	85	Outras Atividades de Ensino	Ensino de Arte e Cultura (outros)	50% trabalhadores<br>50% alunos	Teletrabalho / Presencial restrito	Ensino remoto / Atendimento individualizado ou em pequenos grupos, respeitando teto de ocupação / Material individual	X	X		Portaria SES/SEDUC nº 01
Educação	85	Outras Atividades de Ensino	Formação profissional, formação continuada, cursos preparatórios para concurso, treinamentos e similares	50% trabalhadores<br>50% alunos	Teletrabalho / Presencial restrito	Ensino remoto / Atendimento individualizado ou em pequenos grupos, respeitando teto de ocupação / Material individual	X	X		Portaria SES/SEDUC nº 01

BANDEIRA VERMELHA - Educação

// Atividade				// Critérios específicos de funcionamento (conforme bandeira)			// Protocolos obrigatório (todas as bandeiras)	// Protocolos variáveis (recomendados)		// Restrições adicionais
Grupo	CNAE (2 díg.)	Tipo	Subtipos	Teto de Operação<br>Determina o percentual máximo de trabalhadores/público presentes no mesmo turno, ao mesmo tempo.<br><br>Deve respeitar ao nº máximo de pessoas no espaço físico, considerando o distanciamento interpessoal mínimo obrigatório (teto de ocupação).	Modo de Operação<br>Forma de operação da atividade, respeitando ao teto de operação, ao teto de ocupação do espaço físico e aos protocolos obrigatórios (ao lado).<br><br>Trabalhadores	Atendimento	Decreto nº 55.2540:<br>- Máscara / EPIs,<br>- Distanciamento,<br>- Teto de ocupação,<br>- Higienização,<br>- Proteção de grupo de risco,<br>- Afastamento de casos,<br>- Cuidados com o público,<br>- Atendimento do grupos de risco<br>- Informativo visível (operação, ocupação e cuidados)	Monitora-mento de tempera- tura	Testagem dos trabalha- dores	Conteúdo completo das normas obrigatórias específicas à atividade:<br>coronavirus.rs.gov.br/portarias-da-ses
Educação	85	Educação Infantil	Creche e Pré-Escola	Regra Geral:<br>Remoto<br><br>Se permitida atividade presencial:<br>50% alunos por sala de aula	Teletrabalho / Presencial restrito	Regra Geral:<br>Ensino Remoto<br><br>Se permitida atividade presencial:<br>Ensino Remoto /<br>Ensino Híbrido (remoto e/ou presencial) /<br>Presencial Restrito /<br>50% alunos por sala de aula /<br>Distanciamento mínimo /<br>Materiais individuais /<br>Vedado atividades coletivas que envolvam aglomeração ou contato físico.	X	X		Portaria SES/SEDUC nº 01/2020, Decreto Estadual nº 55.465 (05/09/2020) e demais normativas.
Educação	85	Ensino Fundamental	Ensino Fundamental - Anos Iniciais	Regra Geral:<br>Remoto<br><br>Se permitida atividade presencial:<br>50% alunos por sala de aula	Teletrabalho / Presencial restrito	Regra Geral:<br>Ensino Remoto<br><br>Se permitida atividade presencial:<br>Ensino Remoto /<br>Ensino Híbrido (remoto e/ou presencial) /<br>Presencial Restrito /<br>50% alunos por sala de aula /<br>Distanciamento mínimo /<br>Materiais individuais /<br>Vedado atividades coletivas que envolvam aglomeração ou contato físico.	X	X		Portaria SES/SEDUC nº 01/2020, Decreto Estadual nº 55.465 (05/09/2020) e demais normativas.
Educação	85	Ensino Fundamental	Ensino Fundamental - Anos Finais	Regra Geral:<br>Remoto<br><br>Se permitida atividade presencial:<br>50% alunos por sala de aula	Teletrabalho / Presencial restrito	Regra Geral:<br>Ensino Remoto<br><br>Se permitida atividade presencial:<br>Ensino Remoto /<br>Ensino Híbrido (remoto e/ou presencial) /<br>Presencial Restrito /<br>50% alunos por sala de aula /<br>Distanciamento mínimo /<br>Materiais individuais /<br>Vedado atividades coletivas que envolvam aglomeração ou contato físico.	X	X		Portaria SES/SEDUC nº 01/2020, Decreto Estadual nº 55.465 (05/09/2020) e demais normativas.
Educação	85	Ensino Médio	Ensino Médio	Regra Geral:<br>Remoto<br><br>Se permitida atividade presencial:<br>50% alunos por sala de aula	Teletrabalho / Presencial restrito	Regra Geral:<br>Ensino Remoto<br><br>Se permitida atividade presencial:<br>Ensino Remoto /<br>Ensino Híbrido (remoto e/ou presencial) /<br>Presencial Restrito /<br>50% alunos por sala de aula /<br>Distanciamento mínimo /<br>Materiais individuais /<br>Vedado atividades coletivas que envolvam aglomeração ou contato físico.	X	X		Portaria SES/SEDUC nº 01/2020, Decreto Estadual nº 55.465 (05/09/2020) e demais normativas.

BANDEIRA VERMELHA - Educação

// Atividade				// Critérios específicos de funcionamento (conforme bandeira)			// Protocolos obrigatório (todas as bandeiras)	// Protocolos variáveis (recomendados)		// Restrições adicionais
Grupo	CNAE (2 díg.)	Tipo	Subtipos	**Teto de Operação**<br>Determina o percentual máximo de trabalhadores/público presentes no mesmo turno, ao mesmo tempo.<br><br>Deve respeitar ao nº máximo de pessoas no espaço físico, considerando o distanciamento interpessoal mínimo obrigatório (teto de ocupação).	**Modo de Operação**<br>Forma de operação da atividade, respeitando ao teto de operação, ao teto de ocupação do espaço físico e aos protocolos obrigatórios (ao lado).<br><br>Trabalhadores	Atendimento	**Decreto nº 55.2540:**<br>- Máscara / EPIs,<br>- Distanciamento,<br>- Teto de ocupação,<br>- Higienização,<br>- Proteção de grupo de risco,<br>- Afastamento de casos,<br>- Cuidados com o público,<br>- Atendimento do grupos de risco<br>- Informativo visível (operação, ocupação e cuidados)	**Monitora- mento de tempera- tura**	**Testagem dos trabalha- dores**	Conteúdo completo das normas obrigatórias específicas à atividade: <u>coronavirus.rs.gov.br/portarias-da-ses</u>
Educação	85	Ensino Médio	Ensino Técnico de Nível Médio e Normal	Regra Geral:<br>Remoto<br><br>Se permitida atividade presencial:<br>50% alunos por sala de aula	Teletrabalho / Presencial restrito	Regra Geral:<br>Ensino Remoto<br><br>Se permitida atividade presencial:<br>Ensino Remoto /<br>Ensino Híbrido (remoto e/ou presencial) /<br>Presencial Restrito /<br>50% alunos por sala de aula /<br>Distanciamento mínimo /<br>Materiais individuais /<br>Vedado atividades coletivas que envolvam aglomeração ou contato físico.	X	X		Portaria SES/SEDUC nº 01/2020, Decreto Estadual nº 55.465 (05/09/2020) e demais normativas.
Educação	85	Ensino Superior	Graduação (Bacharelado, Licenciatura, Tecnólogo) e Pós-graduação (stricto e latu sensu)	Regra Geral:<br>Remoto<br><br>Se permitida atividade presencial:<br>50% alunos por sala de aula	Teletrabalho / Presencial restrito	Regra Geral:<br>Ensino Remoto<br><br>Se permitida atividade presencial:<br>Ensino Remoto /<br>Ensino Híbrido (remoto e/ou presencial) /<br>Presencial Restrito /<br>50% alunos por sala de aula /<br>Distanciamento mínimo /<br>Materiais individuais /<br>Vedado atividades coletivas que envolvam aglomeração ou contato físico.	X	X		Portaria SES/SEDUC nº 01/2020, Decreto Estadual nº 55.465 (05/09/2020) e demais normativas.
Educação	85	Ensino Superior	Ensino Médio Técnico <u>Concomitante</u> e Subseqüente, Ensino Superior e Pós-Graduação<br><br>(*somente atividades práticas essenciais para conclusão de curso da <u>área da saúde</u>* : pesquisa, estágio curricular obrigatório, laboratórios e plantão*)	50% trabalhadores<br>50% alunos	Teletrabalho / Presencial restrito	Regra Geral:<br>Presencial restrito /<br>Atendimento individualizado sob agendamento /<br>Atividades práticas em pequenos grupos, respeitando teto de ocupação /<br>Material individual<br><br>Se permitida atividade presencial do segmento:<br>Ensino Remoto /<br>Ensino Híbrido (remoto e/ou presencial) /<br>Presencial Restrito /<br>50% alunos por sala de aula /<br>Distanciamento mínimo /<br>Materiais individuais /<br>Vedado atividades coletivas que envolvam aglomeração ou contato físico.	X	X		Portaria SES/SEDUC nº 01/2020, Decreto Estadual nº 55.465 (05/09/2020) e demais normativas.

BANDEIRA VERMELHA - Educação

// Atividade				// Critérios específicos de funcionamento (conforme bandeira)			// Protocolos obrigatório (todas as bandeiras)	// Protocolos variáveis (recomendados)		// Restrições adicionais
Grupo	CNAE (2 díg.)	Tipo	Subtipos	**Teto de Operação**<br>Determina o percentual máximo de trabalhadores/público presentes no mesmo turno, ao mesmo tempo.<br><br>Deve respeitar ao nº máximo de pessoas no espaço físico, considerando o distanciamento interpessoal mínimo obrigatório (teto de ocupação).	**Modo de Operação**<br>Forma de operação da atividade, respeitando ao teto de operação, ao teto de ocupação do espaço físico e aos protocolos obrigatórios (ao lado).<br><br>**Trabalhadores**	**Atendimento**	**Decreto nº 55.2540:**<br>- Máscara / EPIs,<br>- Distanciamento,<br>- Teto de ocupação,<br>- Higienização,<br>- Proteção de grupo de risco,<br>- Afastamento de casos,<br>- Cuidados com o público,<br>- Atendimento do grupos de risco<br>- Informativo visível (operação, ocupação e cuidados)	**Monitora-mento de tempera- tura**	**Testagem dos trabalha- dores**	Conteúdo completo das normas obrigatórias específicas à atividade:<br>coronavirus.rs.gov.br/portarias-da-ses
Educação		Ensino Médio e Superior	Ensino Médio Técnico Subseqüente, Ensino Superior e Pós-Graduação<br>(*somente atividades práticas essenciais para conclusão de curso: pesquisa, estágio curricular obrigatório, laboratórios e plantão*)	Regra Geral:<br>25% trabalhadores<br><br>Se permitida atividade presencial do segmento:<br>50% trabalhadores<br>50% alunos	Teletrabalho / Presencial restrito	Regra Geral:<br>Presencial restrito / Atendimento individualizado sob agendamento (exclusivo para atividades de laboratório, necessárias à manutenção de seres vivos)<br><br>Se permitida atividade presencial do segmento:<br>Ensino Remoto /<br>Ensino Híbrido (remoto e/ou presencial) /<br>Presencial Restrito /<br>50% alunos por sala de aula /<br>Distanciamento mínimo /<br>Materiais individuais /<br>Vedado atividades coletivas que envolvam aglomeração ou contato físico.	X	X		Portaria SES/SEDUC nº 01/2020, Decreto Estadual nº 55.465 (05/09/2020) e demais normativas.
Educação	85	Educação - Outros	Atividades de Apoio à Educação	Regra Geral:<br>25% trabalhadores<br><br>Se permitida atividade presencial do segmento:<br>50% trabalhadores	Teletrabalho / Presencial restrito	Teleatendimento / Presencial restrito	X	X		Portaria SES/SEDUC nº 01
Educação	85	Outras Atividades de Ensino	Ensino de Idiomas	Regra Geral:<br>Remoto<br><br>Se permitida atividade presencial do segmento:<br>50% trabalhadores<br>50% alunos	Teletrabalho / Presencial restrito	Regra Geral:<br>Ensino remoto<br><br>Se permitida atividade presencial do segmento:<br>Ensino remoto /<br>Atendimento individualizado ou em pequenos grupos, respeitando teto de ocupação /<br>Material individual	X	X		Portaria SES/SEDUC nº 01
Educação	85	Outras Atividades de Ensino	Ensino de Música	Regra Geral:<br>Remoto<br><br>Se permitida atividade presencial do segmento:<br>50% trabalhadores<br>50% alunos	Teletrabalho / Presencial restrito	Regra Geral:<br>Ensino remoto<br><br>Se permitida atividade presencial do segmento:<br>Ensino remoto /<br>Atendimento individualizado ou em pequenos grupos, respeitando teto de ocupação /<br>Material individual	X	X		Portaria SES/SEDUC nº 01
Educação	85	Outras Atividades de Ensino	Ensino de Esportes, Dança e Artes Cênicas	Regra Geral:<br>Remoto<br><br>Se permitida atividade presencial do segmento:<br>50% trabalhadores<br>50% alunos	Teletrabalho / Presencial restrito	Regra Geral:<br>Ensino remoto<br><br>Se permitida atividade presencial do segmento:<br>Ensino remoto /<br>Atendimento individualizado ou coabitantes /<br>Material individual /	X	X		Portaria SES nº 582

BANDEIRA VERMELHA - Educação

// Atividade				// Critérios específicos de funcionamento (conforme bandeira)			// Protocolos obrigatório (todas as bandeiras)	// Protocolos variáveis (recomendados)		// Restrições adicionais
Grupo	CNAE (2 díg.)	Tipo	Subtipos	**Teto de Operação** Determina o percentual máximo de trabalhadores/público presentes no mesmo turno, ao mesmo tempo. Deve respeitar ao nº máximo de pessoas no espaço físico, considerando o distanciamento interpessoal mínimo obrigatório (teto de ocupação).	**Modo de Operação** Forma de operação da atividade, respeitando ao teto de operação, ao teto de ocupação do espaço físico e aos protocolos obrigatórios (ao lado). Trabalhadores	Atendimento	**Decreto nº 55.2540:** - Máscara / EPIs, - Distanciamento, - Teto de ocupação, - Higienização, - Proteção de grupo de risco, - Afastamento de casos, - Cuidados com o público, - Atendimento do grupos de risco - Informativo visível (operação, ocupação e cuidados)	**Monitora- mento de tempera- tura**	**Testagem dos trabalha- dores**	Conteúdo completo das normas obrigatórias específicas à atividade: coronavirus.rs.gov.br/portarias-da-ses
Educação	85	Outras Atividades de Ensino	Ensino de Arte e Cultura (outros)	Regra Geral: Remoto<br><br>Se permitida atividade presencial do segmento: 50% trabalhadores 50% alunos	Teletrabalho / Presencial restrito	Regra Geral: Ensino remoto<br><br>Se permitida atividade presencial do segmento: Ensino remoto / Atendimento individualizado ou em pequenos grupos, respeitando teto de ocupação / Material individual	X	X		Portaria SES/SEDUC nº 01
Educação	85	Outras Atividades de Ensino	Formação profissional, formação continuada, cursos preparatórios para concurso, treinamentos e similares	Regra Geral: Remoto<br><br>Se permitida atividade presencial do segmento: 50% trabalhadores 50% alunos	Teletrabalho / Presencial restrito	Regra Geral: Ensino remoto<br><br>Se permitida atividade presencial do segmento: Ensino remoto / Atendimento individualizado ou em pequenos grupos, respeitando teto de ocupação / Material individual	X	X		Portaria SES/SEDUC nº 01

DISTANCIAMENTO CONTROLADO
.GOV RS
NOVAS FAÇANHAS

BANDEIRA PRETA - Educação

// Atividade				// Critérios específicos de funcionamento (conforme bandeira)			// Protocolos obrigatório (todas as bandeiras)	// Protocolos variáveis (recomendados)		// Restrições adicionais
Grupo	CNAE (2 díg.)	Tipo	Subtipos	**Teto de Operação**<br>Determina o percentual máximo de trabalhadores/público presentes no mesmo turno, ao mesmo tempo.<br><br>Deve respeitar ao nº máximo de pessoas no espaço físico, considerando o distanciamento interpessoal mínimo obrigatório (teto de ocupação).	**Modo de Operação**<br>Forma de operação da atividade, respeitando ao teto de operação, ao teto de ocupação do espaço físico e aos protocolos obrigatórios (ao lado).<br><br>**Trabalhadores**	**Atendimento**	**Decreto nº 55.2540:**<br>- Máscara / EPIs,<br>- Distanciamento,<br>- Teto de ocupação,<br>- Higienização,<br>- Proteção de grupo de risco,<br>- Afastamento de casos,<br>- Cuidados com o público,<br>- Atendimento do grupos de risco<br>- Informativo visível (operação, ocupação e cuidados)	**Monitora- mento de tempera- tura**	**Testagem dos trabalha- dores**	Conteúdo completo das normas obrigatórias específicas à atividade: coronavirus.rs.gov.br/portarias-da-ses
Educação	85	Educação Infantil	Creche e Pré-Escola	(remoto)	Teletrabalho	(exclusivo)<br>Ensino remoto				Portaria SES/SEDUC nº 01/2020, Decreto Estadual nº 55.465 (05/09/2020) e demais normativas.
Educação	85	Ensino Fundamental	Ensino Fundamental - Anos Iniciais	(remoto)	Teletrabalho	(exclusivo)<br>Ensino remoto				Portaria SES/SEDUC nº 01/2020, Decreto Estadual nº 55.465 (05/09/2020) e demais normativas.
Educação	85	Ensino Fundamental	Ensino Fundamental - Anos Finais	(remoto)	Teletrabalho	(exclusivo)<br>Ensino remoto				Portaria SES/SEDUC nº 01/2020, Decreto Estadual nº 55.465 (05/09/2020) e demais normativas.
Educação	85	Ensino Médio	Ensino Médio	(remoto)	Teletrabalho	(exclusivo)<br>Ensino remoto				Portaria SES/SEDUC nº 01/2020, Decreto Estadual nº 55.465 (05/09/2020) e demais normativas.
Educação	85	Ensino Médio	Ensino Técnico de Nível Médio e Normal	(remoto)	Teletrabalho	(exclusivo)<br>Ensino remoto				Portaria SES/SEDUC nº 01/2020, Decreto Estadual nº 55.465 (05/09/2020) e demais normativas.
Educação	85	Ensino Superior	Graduação (Bacharelado, Licenciatura, Tecnólogo) e Pós-graduação (stricto e latu sensu)	(remoto)	Teletrabalho	(exclusivo)<br>Ensino remoto				Portaria SES/SEDUC nº 01/2020, Decreto Estadual nº 55.465 (05/09/2020) e demais normativas.
Educação	85	Ensino Superior	Ensino Médio Técnico Concomitante e Subseqüente, Ensino Superior e Pós-Graduação<br><br>*(somente atividades práticas essenciais para conclusão de curso da área da saúde*: pesquisa, estágio curricular obrigatório, laboratórios e plantão)*	50% trabalhadores<br>50% alunos	Teletrabalho / Presencial restrito	Presencial restrito / Atendimento individualizado sob agendamento / Atividades práticas em pequenos grupos, respeitando teto de ocupação / Material individual	X	X		Portaria SES/SEDUC nº 01/2020, Decreto Estadual nº 55.465 (05/09/2020) e demais normativas.

BANDEIRA PRETA - Educação

// Atividade				// Critérios específicos de funcionamento (conforme bandeira)			// Protocolos obrigatório (todas as bandeiras)	// Protocolos variáveis (recomendados)		// Restrições adicionais
Grupo	CNAE (2 díg.)	Tipo	Subtipos	**Teto de Operação**<br>Determina o percentual máximo de trabalhadores/público presentes no mesmo turno, ao mesmo tempo.<br><br>Deve respeitar ao nº máximo de pessoas no espaço físico, considerando o distanciamento interpessoal mínimo obrigatório (teto de ocupação).	**Modo de Operação**<br>Forma de operação da atividade, respeitando ao teto de operação, ao teto de ocupação do espaço físico e aos protocolos obrigatórios (ao lado).<br><br>**Trabalhadores**	 <br><br><br>**Atendimento**	**Decreto nº 55.2540:**<br>- Máscara / EPIs,<br>- Distanciamento,<br>- Teto de ocupação,<br>- Higienização,<br>- Proteção de grupo de risco,<br>- Afastamento de casos,<br>- Cuidados com o público,<br>- Atendimento do grupos de risco<br>- Informativo visível (operação, ocupação e cuidados)	**Monitora- mento de tempera- tura**	**Testagem dos trabalha- dores**	Conteúdo completo das normas obrigatórias específicas à atividade: coronavirus.rs.gov.br/portarias-da-ses
Educação		Ensino Médio e Superior	Ensino Médio Técnico Subseqüente, Ensino Superior e Pós-Graduação (*somente atividades práticas essenciais para conclusão de curso: pesquisa, estágio curricular obrigatório, laboratórios e plantão*)	25% trabalhadores	Teletrabalho	Presencial restrito / Atendimento individualizado sob agendamento (exclusivo para atividades de laboratório, necessárias à manutenção de seres vivos)	X	X		Portaria SES/SEDUC nº 01/2020, Decreto Estadual nº 55.465 (05/09/2020) e demais normativas.
Educação	85	Educação - Outros	Atividades de Apoio à Educação	25% trabalhadores	Teletrabalho	Teleatendimento	X	X		Portaria SES/SEDUC nº 01
Educação	85	Outras Atividades de Ensino	Ensino de Idiomas	(remoto)	Teletrabalho	(exclusivo) Ensino remoto				Portaria SES/SEDUC nº 01
Educação	85	Outras Atividades de Ensino	Ensino de Música	(remoto)	Teletrabalho	(exclusivo) Ensino remoto				Portaria SES/SEDUC nº 01
Educação	85	Outras Atividades de Ensino	Ensino de Esportes, Dança e Artes Cênicas	(remoto)	Teletrabalho	(exclusivo) Ensino remoto				Portaria SES nº 582
Educação	85	Outras Atividades de Ensino	Ensino de Arte e Cultura (outros)	(remoto)	Teletrabalho	(exclusivo) Ensino remoto				Portaria SES/SEDUC nº 01
Educação	85	Outras Atividades de Ensino	Formação profissional, formação continuada, cursos preparatórios para concurso, treinamentos e similares	(remoto)	Teletrabalho	(exclusivo) Ensino remoto				Portaria SES/SEDUC nº 01

MODELO DE DISTANCIAMENTO
CONTROLADO DO RS

Indústria

DISTANCIAMENTO CONTROLADO
GOV RS
NOVAS FAÇANHAS

Comitê de DADOS
COVID-19 CORONAVÍRUS

GOV RS
NOVAS FAÇANHAS
NO PLANEJAMENTO, GOVERNANÇA E GESTÃO NA SAÚDE

BANDEIRA AMARELA - Indústria

// Atividade				// Critérios específicos de funcionamento (conforme bandeira)			// Protocolos obrigatório (todas as bandeiras)	// Protocolos variáveis (recomendados)		// Restrições adicionais
Grupo	CNAE (2 díg.)	Tipo	Subtipos	Teto de Operação (percentual máx. de trabalhadores presentes no turno, ao mesmo tempo, respeitando o teto de ocupação do espaço físico - máx. pessoas)	Modo de Operação (forma de operação, respeitando o teto de operação e o teto de ocupação do espaço físico - máx. pessoas)<br>Trabalhadores	Atendimento	Informativo visível (operação e ocupação) Máscara / EPIs, Distanciamento, Teto de ocupação, Higienização, Proteção de grupo de risco, Afastamento de casos, Cuidados no atendimento ao	Monitora- mento de tempera- tura	Testagem dos trabalha- dores	Normas obrigatórias específicas à atividade https://coronavirus.rs.gov.br/portarias-da-ses
Indústria de Construção	41	Construção de Edifícios		100% trabalhadores	Teletrabalho / Presencial restrito / Ventilação cruzada (portas e janelas abertas) e/ou sistema de renovação de ar / Restaurantes, bares, lanchonetes e espaços coletivos de alimentação: conforme protocolo de "Restaurantes" e "Lanchonetes" e Portaria SES nº 319 /	Uso obrigatório e correto de máscara, cobrindo boca e nariz por todos os presentes/ Distanciamento interpessoal mínimo de 1m /	X			Portaria SES nº 283 e nº 375
Indústria de Construção	42	Obras de Infraestrutura		100% trabalhadores	Teletrabalho / Presencial restrito / Ventilação cruzada (portas e janelas abertas) e/ou sistema de renovação de ar / Restaurantes, bares, lanchonetes e espaços coletivos de alimentação: conforme protocolo de "Restaurantes" e "Lanchonetes" e Portaria SES nº 319 /	Uso obrigatório e correto de máscara, cobrindo boca e nariz por todos os presentes/ Distanciamento interpessoal mínimo de 1m /	X			Portaria SES nº 283 e nº 375
Indústria de Construção	43	Serviços de Construção		100% trabalhadores	Teletrabalho / Presencial restrito / Ventilação cruzada (portas e janelas abertas) e/ou sistema de renovação de ar / Restaurantes, bares, lanchonetes e espaços coletivos de alimentação: conforme protocolo de "Restaurantes" e "Lanchonetes" e Portaria SES nº 319 /	Uso obrigatório e correto de máscara, cobrindo boca e nariz por todos os presentes/ Distanciamento interpessoal mínimo de 1m /	X			Portaria SES nº 283 e nº 375
Indústria de Transformação e Extrativa	5	Extração de Carvão Mineral		100% trabalhadores	Teletrabalho / Presencial restrito / Ventilação cruzada (portas e janelas abertas) e/ou sistema de renovação de ar / Restaurantes, bares, lanchonetes e espaços coletivos de alimentação: conforme protocolo de "Restaurantes" e "Lanchonetes" e Portaria SES nº 319 /	Uso obrigatório e correto de máscara, cobrindo boca e nariz por todos os presentes/ Distanciamento interpessoal mínimo de 1m /	X			Portaria SES nº 283 e nº 375
Indústria de Transformação e Extrativa	100*	Extr. de Petróleo e Minerais	Extração de Petróleo e Gás	100% trabalhadores	Teletrabalho / Presencial restrito / Ventilação cruzada (portas e janelas abertas) e/ou sistema de renovação de ar / Restaurantes, bares, lanchonetes e espaços coletivos de alimentação: conforme protocolo de "Restaurantes" e "Lanchonetes" e Portaria SES nº 319 /	Uso obrigatório e correto de máscara, cobrindo boca e nariz por todos os presentes/ Distanciamento interpessoal mínimo de 1m /	X			Portaria SES nº 283 e nº 375
Indústria de Transformação e Extrativa	100*	Extr. de Petróleo e Minerais	Extr. de Petróleo e Minerais - Outros	100% trabalhadores	Teletrabalho / Presencial restrito / Ventilação cruzada (portas e janelas abertas) e/ou sistema de renovação de ar / Restaurantes, bares, lanchonetes e espaços coletivos de alimentação: conforme protocolo de "Restaurantes" e "Lanchonetes" e Portaria SES nº 319 /	Uso obrigatório e correto de máscara, cobrindo boca e nariz por todos os presentes/ Distanciamento interpessoal mínimo de 1m /	X			Portaria SES nº 283 e nº 375

DISTANCIAMENTO CONTROLADO RS NOVAS FAÇANHAS

Notas:
(*) Representam agregações de atividades 2 dígitos:
100* = 6, 7, 8, 9

BANDEIRA AMARELA - Indústria

// Atividade				// Critérios específicos de funcionamento (conforme bandeira)			// Protocolos obrigatório (todas as bandeiras)	// Protocolos variáveis (recomendados)		// Restrições adicionais
Grupo	CNAE (2 díg.)	Tipo	Subtipos	Teto de Operação (percentual máx. de trabalhadores presentes no turno, ao mesmo tempo, respeitando o teto de ocupação do espaço físico - máx. pessoas)	Modo de Operação (forma de operação, respeitando o teto de operação e o teto de ocupação do espaço físico - máx. pessoas) Trabalhadores	Atendimento	Informativo visível (operação e ocupação) Máscara / EPIs, Distanciamento, Teto de ocupação, Higienização, Proteção de grupo de risco, Afastamento de casos, Cuidados no atendimento ao	Monitora- mento de tempera- tura	Testagem dos trabalha- dores	Normas obrigatórias específicas à atividade https://coronavirus.rs.gov.br/portarias-da-ses
Indústria de Transformação e Extrativa	10	Alimentos		100% trabalhadores	Teletrabalho / Presencial restrito / Ventilação cruzada (portas e janelas abertas) e/ou sistema de renovação de ar / Restaurantes, bares, lanchonetes e espaços coletivos de alimentação: conforme protocolo de "Restaurantes" e "Lanchonetes" e Portaria SES nº 319 /	Uso obrigatório e correto de máscara, cobrindo boca e nariz por todos os presentes/ Distanciamento interpessoal mínimo de 1m /	X			Portaria SES nº 283 e nº 375
Indústria de Transformação e Extrativa	11	Bebidas		100% trabalhadores	Teletrabalho / Presencial restrito / Ventilação cruzada (portas e janelas abertas) e/ou sistema de renovação de ar / Restaurantes, bares, lanchonetes e espaços coletivos de alimentação: conforme protocolo de "Restaurantes" e "Lanchonetes" e Portaria SES nº 319 /	Uso obrigatório e correto de máscara, cobrindo boca e nariz por todos os presentes/ Distanciamento interpessoal mínimo de 1m /	X			Portaria SES nº 283 e nº 375
Indústria de Transformação e Extrativa	12	Fumo		100% trabalhadores	Teletrabalho / Presencial restrito / Ventilação cruzada (portas e janelas abertas) e/ou sistema de renovação de ar / Restaurantes, bares, lanchonetes e espaços coletivos de alimentação: conforme protocolo de "Restaurantes" e "Lanchonetes" e Portaria SES nº 319 /	Uso obrigatório e correto de máscara, cobrindo boca e nariz por todos os presentes/ Distanciamento interpessoal mínimo de 1m /	X			Portaria SES nº 283 e nº 375
Indústria de Transformação e Extrativa	13	Têxteis		100% trabalhadores	Teletrabalho / Presencial restrito / Ventilação cruzada (portas e janelas abertas) e/ou sistema de renovação de ar / Restaurantes, bares, lanchonetes e espaços coletivos de alimentação: conforme protocolo de "Restaurantes" e "Lanchonetes" e Portaria SES nº 319 /	Uso obrigatório e correto de máscara, cobrindo boca e nariz por todos os presentes/ Distanciamento interpessoal mínimo de 1m /	X			Portaria SES nº 283 e nº 375
Indústria de Transformação e Extrativa	14	Vestuário		100% trabalhadores	Teletrabalho / Presencial restrito / Ventilação cruzada (portas e janelas abertas) e/ou sistema de renovação de ar / Restaurantes, bares, lanchonetes e espaços coletivos de alimentação: conforme protocolo de "Restaurantes" e "Lanchonetes" e Portaria SES nº 319 /	Uso obrigatório e correto de máscara, cobrindo boca e nariz por todos os presentes/ Distanciamento interpessoal mínimo de 1m /	X			Portaria SES nº 283 e nº 375
Indústria de Transformação e Extrativa	15	Couros e Calçados		100% trabalhadores	Teletrabalho / Presencial restrito / Ventilação cruzada (portas e janelas abertas) e/ou sistema de renovação de ar / Restaurantes, bares, lanchonetes e espaços coletivos de alimentação: conforme protocolo de "Restaurantes" e "Lanchonetes" e Portaria SES nº 319 /	Uso obrigatório e correto de máscara, cobrindo boca e nariz por todos os presentes/ Distanciamento interpessoal mínimo de 1m /	X			Portaria SES nº 283 e nº 375

DISTANCIAMENTO CONTROLADO
GOV RS
NOVAS FAÇANHAS

Notas:
(*) Representam agregações de atividades 2 dígitos:
100* = 6, 7, 8, 9

BANDEIRA AMARELA - Indústria

// Atividade				// Critérios específicos de funcionamento (conforme bandeira)			// Protocolos obrigatório (todas as bandeiras)	// Protocolos variáveis (recomendados)		// Restrições adicionais
Grupo	CNAE (2 díg.)	Tipo	Subtipos	Teto de Operação (percentual máx. de trabalhadores presentes no turno, ao mesmo tempo, respeitando o teto de ocupação do espaço físico - máx. pessoas)	Modo de Operação (forma de operação, respeitando o teto de operação e o teto de ocupação do espaço físico - máx. pessoas)<br>Trabalhadores	Atendimento	Informativo visível (operação e ocupação) Máscara / EPIs, Distanciamento, Teto de ocupação, Higienização, Proteção de grupo de risco, Afastamento de casos, Cuidados no atendimento ao	Monitora- mento de tempera- tura	Testagem dos trabalha- dores	Normas obrigatórias específicas à atividade https://coronavirus.rs.gov.br/portarias-da-ses
Indústria de Transformação e Extrativa	16	Madeira		100% trabalhadores	Teletrabalho / Presencial restrito / Ventilação cruzada (portas e janelas abertas) e/ou sistema de renovação de ar / Restaurantes, bares, lanchonetes e espaços coletivos de alimentação: conforme protocolo de "Restaurantes" e "Lanchonetes" e Portaria SES nº 319 /	Uso obrigatório e correto de máscara, cobrindo boca e nariz por todos os presentes/ Distanciamento interpessoal mínimo de 1m /	X			Portaria SES nº 283 e nº 375
Indústria de Transformação e Extrativa	17	Papel e Celulose		100% trabalhadores	Teletrabalho / Presencial restrito / Ventilação cruzada (portas e janelas abertas) e/ou sistema de renovação de ar / Restaurantes, bares, lanchonetes e espaços coletivos de alimentação: conforme protocolo de "Restaurantes" e "Lanchonetes" e Portaria SES nº 319 /	Uso obrigatório e correto de máscara, cobrindo boca e nariz por todos os presentes/ Distanciamento interpessoal mínimo de 1m /	X			Portaria SES nº 283 e nº 375
Indústria de Transformação e Extrativa	18	Impressão e Reprodução		100% trabalhadores	Teletrabalho / Presencial restrito / Ventilação cruzada (portas e janelas abertas) e/ou sistema de renovação de ar / Restaurantes, bares, lanchonetes e espaços coletivos de alimentação: conforme protocolo de "Restaurantes" e "Lanchonetes" e Portaria SES nº 319 /	Uso obrigatório e correto de máscara, cobrindo boca e nariz por todos os presentes/ Distanciamento interpessoal mínimo de 1m /	X			Portaria SES nº 283 e nº 375
Indústria de Transformação e Extrativa	19	Derivados Petróleo		100% trabalhadores	Teletrabalho / Presencial restrito / Ventilação cruzada (portas e janelas abertas) e/ou sistema de renovação de ar / Restaurantes, bares, lanchonetes e espaços coletivos de alimentação: conforme protocolo de "Restaurantes" e "Lanchonetes" e Portaria SES nº 319 /	Uso obrigatório e correto de máscara, cobrindo boca e nariz por todos os presentes/ Distanciamento interpessoal mínimo de 1m /	X			Portaria SES nº 283 e nº 375
Indústria de Transformação e Extrativa	20	Químicos		100% trabalhadores	Teletrabalho / Presencial restrito / Ventilação cruzada (portas e janelas abertas) e/ou sistema de renovação de ar / Restaurantes, bares, lanchonetes e espaços coletivos de alimentação: conforme protocolo de "Restaurantes" e "Lanchonetes" e Portaria SES nº 319 /	Uso obrigatório e correto de máscara, cobrindo boca e nariz por todos os presentes/ Distanciamento interpessoal mínimo de 1m /	X			Portaria SES nº 283 e nº 375
Indústria de Transformação e Extrativa	22	Borracha e Plástico		100% trabalhadores	Teletrabalho / Presencial restrito / Ventilação cruzada (portas e janelas abertas) e/ou sistema de renovação de ar / Restaurantes, bares, lanchonetes e espaços coletivos de alimentação: conforme protocolo de "Restaurantes" e "Lanchonetes" e Portaria SES nº 319 /	Uso obrigatório e correto de máscara, cobrindo boca e nariz por todos os presentes/ Distanciamento interpessoal mínimo de 1m /	X			Portaria SES nº 283 e nº 375

DISTANCIAMENTO CONTROLADO
GOV RS
NOVAS FAÇANHAS

Notas:
(*) Representam agregações de atividades 2 dígitos:
100* = 6, 7, 8, 9

BANDEIRA AMARELA - Indústria

// Atividade				// Critérios específicos de funcionamento (conforme bandeira)			// Protocolos obrigatório (todas as bandeiras)	// Protocolos variáveis (recomendados)		// Restrições adicionais
Grupo	CNAE (2 díg.)	Tipo	Subtipos	Teto de Operação (percentual máx. de trabalhadores presentes no turno, ao mesmo tempo, respeitando o teto de ocupação do espaço físico - máx. pessoas)	Modo de Operação (forma de operação, respeitando o teto de operação e o teto de ocupação do espaço físico - máx. pessoas)<br>Trabalhadores	Atendimento	Informativo visível (operação e ocupação)<br>Máscara / EPIs,<br>Distanciamento,<br>Teto de ocupação,<br>Higienização,<br>Proteção de grupo de risco,<br>Afastamento de casos,<br>Cuidados no atendimento ao	Monitora- mento de tempera- tura	Testagem dos trabalha- dores	Normas obrigatórias específicas à atividade<br>https://coronavirus.rs.gov.br/portarias-da-ses
Indústria de Transformação e Extrativa	23	Minerais não metálicos		100% trabalhadores	Teletrabalho /<br>Presencial restrito /<br>Ventilação cruzada (portas e janelas abertas) e/ou sistema de renovação de ar /<br>Restaurantes, bares, lanchonetes e espaços coletivos de alimentação: conforme protocolo de "Restaurantes" e "Lanchonetes" e Portaria SES nº 319 /	Uso obrigatório e correto de máscara, cobrindo boca e nariz por todos os presentes/<br>Distanciamento interpessoal mínimo de 1m /	X			Portaria SES nº 283 e nº 375
Indústria de Transformação e Extrativa	24	Metalurgia		100% trabalhadores	Teletrabalho /<br>Presencial restrito /<br>Ventilação cruzada (portas e janelas abertas) e/ou sistema de renovação de ar /<br>Restaurantes, bares, lanchonetes e espaços coletivos de alimentação: conforme protocolo de "Restaurantes" e "Lanchonetes" e Portaria SES nº 319 /	Uso obrigatório e correto de máscara, cobrindo boca e nariz por todos os presentes/<br>Distanciamento interpessoal mínimo de 1m /	X			Portaria SES nº 283 e nº 375
Indústria de Transformação e Extrativa	25	Produtos de Metal		100% trabalhadores	Teletrabalho /<br>Presencial restrito /<br>Ventilação cruzada (portas e janelas abertas) e/ou sistema de renovação de ar /<br>Restaurantes, bares, lanchonetes e espaços coletivos de alimentação: conforme protocolo de "Restaurantes" e "Lanchonetes" e Portaria SES nº 319 /	Uso obrigatório e correto de máscara, cobrindo boca e nariz por todos os presentes/<br>Distanciamento interpessoal mínimo de 1m /	X			Portaria SES nº 283 e nº 375
Indústria de Transformação e Extrativa	26	Equip. Informática		100% trabalhadores	Teletrabalho /<br>Presencial restrito /<br>Ventilação cruzada (portas e janelas abertas) e/ou sistema de renovação de ar /<br>Restaurantes, bares, lanchonetes e espaços coletivos de alimentação: conforme protocolo de "Restaurantes" e "Lanchonetes" e Portaria SES nº 319 /	Uso obrigatório e correto de máscara, cobrindo boca e nariz por todos os presentes/<br>Distanciamento interpessoal mínimo de 1m /	X			Portaria SES nº 283 e nº 375
Indústria de Transformação e Extrativa	27	Materiais Elétricos		100% trabalhadores	Teletrabalho /<br>Presencial restrito /<br>Ventilação cruzada (portas e janelas abertas) e/ou sistema de renovação de ar /<br>Restaurantes, bares, lanchonetes e espaços coletivos de alimentação: conforme protocolo de "Restaurantes" e "Lanchonetes" e Portaria SES nº 319 /	Uso obrigatório e correto de máscara, cobrindo boca e nariz por todos os presentes/<br>Distanciamento interpessoal mínimo de 1m /	X			Portaria SES nº 283 e nº 375
Indústria de Transformação e Extrativa	28	Máquinas e Equipamentos		100% trabalhadores	Teletrabalho /<br>Presencial restrito /<br>Ventilação cruzada (portas e janelas abertas) e/ou sistema de renovação de ar /<br>Restaurantes, bares, lanchonetes e espaços coletivos de alimentação: conforme protocolo de "Restaurantes" e "Lanchonetes" e Portaria SES nº 319 /	Uso obrigatório e correto de máscara, cobrindo boca e nariz por todos os presentes/<br>Distanciamento interpessoal mínimo de 1m /	X			Portaria SES nº 283 e nº 375

DISTANCIAMENTO CONTROLADO
GOV RS
NOVAS FAÇANHAS

Notas:
(*) Representam agregações de atividades 2 dígitos:
100* = 6, 7, 8, 9

BANDEIRA AMARELA - Indústria

// Atividade				// Critérios específicos de funcionamento (conforme bandeira)			// Protocolos obrigatório (todas as bandeiras)	// Protocolos variáveis (recomendados)		// Restrições adicionais
Grupo	CNAE (2 díg.)	Tipo	Subtipos	Teto de Operação (percentual máx. de trabalhadores presentes no turno, ao mesmo tempo, respeitando o teto de ocupação do espaço físico - máx. pessoas)	Modo de Operação (forma de operação, respeitando o teto de operação e o teto de ocupação do espaço físico - máx. pessoas)<br>Trabalhadores	Atendimento	Informativo visível (operação e ocupação)<br>Máscara / EPIs,<br>Distanciamento,<br>Teto de ocupação,<br>Higienização,<br>Proteção de grupo de risco,<br>Afastamento de casos,<br>Cuidados no atendimento ao	Monitora- mento de tempera- tura	Testagem dos trabalha- dores	Normas obrigatórias específicas à atividade<br>https://coronavirus.rs.gov.br/portarias-da-ses
Indústria de Transformação e Extrativa	29	Veículos Automotores		100% trabalhadores	Teletrabalho /<br>Presencial restrito /<br>Ventilação cruzada (portas e janelas abertas) e/ou sistema de renovação de ar /<br><br>Restaurantes, bares, lanchonetes e espaços coletivos de alimentação: conforme protocolo de "Restaurantes" e "Lanchonetes" e Portaria SES nº 319 /	Uso obrigatório e correto de máscara, cobrindo boca e nariz por todos os presentes/<br><br>Distanciamento interpessoal mínimo de 1m /	X			Portaria SES nº 283 e nº 375
Indústria de Transformação e Extrativa	30	Outros Equipamentos		100% trabalhadores	Teletrabalho /<br>Presencial restrito /<br>Ventilação cruzada (portas e janelas abertas) e/ou sistema de renovação de ar /<br><br>Restaurantes, bares, lanchonetes e espaços coletivos de alimentação: conforme protocolo de "Restaurantes" e "Lanchonetes" e Portaria SES nº 319 /	Uso obrigatório e correto de máscara, cobrindo boca e nariz por todos os presentes/<br><br>Distanciamento interpessoal mínimo de 1m /	X			Portaria SES nº 283 e nº 375
Indústria de Transformação e Extrativa	31	Móveis		100% trabalhadores	Teletrabalho /<br>Presencial restrito /<br>Ventilação cruzada (portas e janelas abertas) e/ou sistema de renovação de ar /<br><br>Restaurantes, bares, lanchonetes e espaços coletivos de alimentação: conforme protocolo de "Restaurantes" e "Lanchonetes" e Portaria SES nº 319 /	Uso obrigatório e correto de máscara, cobrindo boca e nariz por todos os presentes/<br><br>Distanciamento interpessoal mínimo de 1m /	X			Portaria SES nº 283 e nº 375
Indústria de Transformação e Extrativa	32	Produtos Diversos		100% trabalhadores	Teletrabalho /<br>Presencial restrito /<br>Ventilação cruzada (portas e janelas abertas) e/ou sistema de renovação de ar /<br><br>Restaurantes, bares, lanchonetes e espaços coletivos de alimentação: conforme protocolo de "Restaurantes" e "Lanchonetes" e Portaria SES nº 319 /	Uso obrigatório e correto de máscara, cobrindo boca e nariz por todos os presentes/<br><br>Distanciamento interpessoal mínimo de 1m /	X			Portaria SES nº 283 e nº 375
Indústria de Transformação e Extrativa	33	Manut. e Reparação		100% trabalhadores	Teletrabalho /<br>Presencial restrito /<br>Ventilação cruzada (portas e janelas abertas) e/ou sistema de renovação de ar /<br><br>Restaurantes, bares, lanchonetes e espaços coletivos de alimentação: conforme protocolo de "Restaurantes" e "Lanchonetes" e Portaria SES nº 319 /	Uso obrigatório e correto de máscara, cobrindo boca e nariz por todos os presentes/<br><br>Distanciamento interpessoal mínimo de 1m /	X			Portaria SES nº 283 e nº 375
Indústria de Transformação e Extrativa	21	Farmoquímicos e Farmacêuticos		100% trabalhadores	Teletrabalho /<br>Presencial restrito /<br>Ventilação cruzada (portas e janelas abertas) e/ou sistema de renovação de ar /<br><br>Restaurantes, bares, lanchonetes e espaços coletivos de alimentação: conforme protocolo de "Restaurantes" e "Lanchonetes" e Portaria SES nº 319 /	Uso obrigatório e correto de máscara, cobrindo boca e nariz por todos os presentes/<br><br>Distanciamento interpessoal mínimo de 1m /	X			Portaria SES nº 283 e nº 375

DISTANCIAMENTO CONTROLADO
GOV RS
NOVAS FAÇANHAS

Notas:
(*) Representam agregações de atividades 2 dígitos:
100* = 6, 7, 8, 9

BANDEIRA LARANJA - Indústria

// Atividade				// Critérios específicos de funcionamento (conforme bandeira)			// Protocolos obrigatório (todas as bandeiras)	// Protocolos variáveis (recomendados)		// Restrições adicionais
Grupo	CNAE (2 díg.)	Tipo	Subtipos	Teto de Operação (percentual máx. de trabalhadores presentes no turno, ao mesmo tempo, respeitando o teto de ocupação do espaço físico - máx. pessoas)	Modo de Operação (forma de operação, respeitando o teto de operação e o teto de ocupação do espaço físico - máx. pessoas)<br>Trabalhadores	Atendimento	Informativo visível (operação e ocupação) Máscara / EPIs, Distanciamento, Teto de ocupação, Higienização, Proteção de grupo de risco, Afastamento de casos, Cuidados no atendimento ao	Monitora-mento de tempera- tura	Testagem dos trabalha- dores	Normas obrigatórias específicas à atividade https://coronavirus.rs.gov.br/portarias-da-ses
Indústria de Construção	41	Construção de Edifícios		75% trabalhadores	Teletrabalho / Presencial restrito / Ventilação cruzada (portas e janelas abertas) e/ou sistema de renovação de ar / Restaurantes, bares, lanchonetes e espaços coletivos de alimentação: conforme protocolo de "Restaurantes" e "Lanchonetes" e Portaria SES nº 319 /	Uso obrigatório e correto de máscara, cobrindo boca e nariz por todos os presentes/ Distanciamento interpessoal mínimo de 1m /	X			Portaria SES nº 283 e nº 375
Indústria de Construção	42	Obras de Infraestrutura		75% trabalhadores	Teletrabalho / Presencial restrito / Ventilação cruzada (portas e janelas abertas) e/ou sistema de renovação de ar / Restaurantes, bares, lanchonetes e espaços coletivos de alimentação: conforme protocolo de "Restaurantes" e "Lanchonetes" e Portaria SES nº 319 /	Uso obrigatório e correto de máscara, cobrindo boca e nariz por todos os presentes/ Distanciamento interpessoal mínimo de 1m /	X			Portaria SES nº 283 e nº 375
Indústria de Construção	43	Serviços de Construção		75% trabalhadores	Teletrabalho / Presencial restrito / Ventilação cruzada (portas e janelas abertas) e/ou sistema de renovação de ar / Restaurantes, bares, lanchonetes e espaços coletivos de alimentação: conforme protocolo de "Restaurantes" e "Lanchonetes" e Portaria SES nº 319 /	Uso obrigatório e correto de máscara, cobrindo boca e nariz por todos os presentes/ Distanciamento interpessoal mínimo de 1m /	X			Portaria SES nº 283 e nº 375
Indústria de Transformação e Extrativa	5	Extração de Carvão Mineral		100% trabalhadores	Teletrabalho / Presencial restrito / Ventilação cruzada (portas e janelas abertas) e/ou sistema de renovação de ar / Restaurantes, bares, lanchonetes e espaços coletivos de alimentação: conforme protocolo de "Restaurantes" e "Lanchonetes" e Portaria SES nº 319 /	Uso obrigatório e correto de máscara, cobrindo boca e nariz por todos os presentes/ Distanciamento interpessoal mínimo de 1m /	X			Portaria SES nº 283 e nº 375
Indústria de Transformação e Extrativa	100*	Extr. de Petróleo e Minerais	Extração de Petróleo e Gás	100% trabalhadores	Teletrabalho / Presencial restrito / Ventilação cruzada (portas e janelas abertas) e/ou sistema de renovação de ar / Restaurantes, bares, lanchonetes e espaços coletivos de alimentação: conforme protocolo de "Restaurantes" e "Lanchonetes" e Portaria SES nº 319 /	Uso obrigatório e correto de máscara, cobrindo boca e nariz por todos os presentes/ Distanciamento interpessoal mínimo de 1m /	X			Portaria SES nº 283 e nº 375
Indústria de Transformação e Extrativa	100*	Extr. de Petróleo e Minerais	Extr. de Petróleo e Minerais - Outros	100% trabalhadores	Teletrabalho / Presencial restrito / Ventilação cruzada (portas e janelas abertas) e/ou sistema de renovação de ar / Restaurantes, bares, lanchonetes e espaços coletivos de alimentação: conforme protocolo de "Restaurantes" e "Lanchonetes" e Portaria SES nº 319 /	Uso obrigatório e correto de máscara, cobrindo boca e nariz por todos os presentes/ Distanciamento interpessoal mínimo de 1m /	X			Portaria SES nº 283 e nº 375

Notas:
(*) Representam agregações de atividades 2 dígitos:
100* = 6, 7, 8, 9

BANDEIRA LARANJA - Indústria

// Atividade				// Critérios específicos de funcionamento (conforme bandeira)			// Protocolos obrigatório (todas as bandeiras)	// Protocolos variáveis (recomendados)		// Restrições adicionais
Grupo	CNAE (2 díg.)	Tipo	Subtipos	Teto de Operação (percentual máx. de trabalhadores presentes no turno, ao mesmo tempo, respeitando o teto de ocupação do espaço físico - máx. pessoas)	Modo de Operação (forma de operação, respeitando o teto de operação e o teto de ocupação do espaço físico - máx. pessoas)<br>Trabalhadores	Atendimento	Informativo visível (operação e ocupação) Máscara / EPIs, Distanciamento, Teto de ocupação, Higienização, Proteção de grupo de risco, Afastamento de casos, Cuidados no atendimento ao	Monitora- mento de tempera- tura	Testagem dos trabalha- dores	Normas obrigatórias específicas à atividade https://coronavirus.rs.gov.br/portarias-da-ses
Indústria de Transformação e Extrativa	10	Alimentos		100% trabalhadores	Teletrabalho / Presencial restrito / Ventilação cruzada (portas e janelas abertas) e/ou sistema de renovação de ar / Restaurantes, bares, lanchonetes e espaços coletivos de alimentação: conforme protocolo de "Restaurantes" e "Lanchonetes" e Portaria SES nº 319 /	Uso obrigatório e correto de máscara, cobrindo boca e nariz por todos os presentes/ Distanciamento interpessoal mínimo de 1m /	X			Portaria SES nº 283 e nº 375
Indústria de Transformação e Extrativa	11	Bebidas		100% trabalhadores	Teletrabalho / Presencial restrito / Ventilação cruzada (portas e janelas abertas) e/ou sistema de renovação de ar / Restaurantes, bares, lanchonetes e espaços coletivos de alimentação: conforme protocolo de "Restaurantes" e "Lanchonetes" e Portaria SES nº 319 /	Uso obrigatório e correto de máscara, cobrindo boca e nariz por todos os presentes/ Distanciamento interpessoal mínimo de 1m /	X			Portaria SES nº 283 e nº 375
Indústria de Transformação e Extrativa	12	Fumo		100% trabalhadores	Teletrabalho / Presencial restrito / Ventilação cruzada (portas e janelas abertas) e/ou sistema de renovação de ar / Restaurantes, bares, lanchonetes e espaços coletivos de alimentação: conforme protocolo de "Restaurantes" e "Lanchonetes" e Portaria SES nº 319 /	Uso obrigatório e correto de máscara, cobrindo boca e nariz por todos os presentes/ Distanciamento interpessoal mínimo de 1m /	X			Portaria SES nº 283 e nº 375
Indústria de Transformação e Extrativa	13	Têxteis		100% trabalhadores	Teletrabalho / Presencial restrito / Ventilação cruzada (portas e janelas abertas) e/ou sistema de renovação de ar / Restaurantes, bares, lanchonetes e espaços coletivos de alimentação: conforme protocolo de "Restaurantes" e "Lanchonetes" e Portaria SES nº 319 /	Uso obrigatório e correto de máscara, cobrindo boca e nariz por todos os presentes/ Distanciamento interpessoal mínimo de 1m /	X			Portaria SES nº 283 e nº 375
Indústria de Transformação e Extrativa	14	Vestuário		100% trabalhadores	Teletrabalho / Presencial restrito / Ventilação cruzada (portas e janelas abertas) e/ou sistema de renovação de ar / Restaurantes, bares, lanchonetes e espaços coletivos de alimentação: conforme protocolo de "Restaurantes" e "Lanchonetes" e Portaria SES nº 319 /	Uso obrigatório e correto de máscara, cobrindo boca e nariz por todos os presentes/ Distanciamento interpessoal mínimo de 1m /	X			Portaria SES nº 283 e nº 375
Indústria de Transformação e Extrativa	15	Couros e Calçados		100% trabalhadores	Teletrabalho / Presencial restrito / Ventilação cruzada (portas e janelas abertas) e/ou sistema de renovação de ar / Restaurantes, bares, lanchonetes e espaços coletivos de alimentação: conforme protocolo de "Restaurantes" e "Lanchonetes" e Portaria SES nº 319 /	Uso obrigatório e correto de máscara, cobrindo boca e nariz por todos os presentes/ Distanciamento interpessoal mínimo de 1m /	X			Portaria SES nº 283 e nº 375

DISTANCIAMENTO CONTROLADO

Notas:
(*) Representam agregações de atividades 2 dígitos:
100* = 6, 7, 8, 9

BANDEIRA LARANJA - Indústria

// Atividade				// Critérios específicos de funcionamento (conforme bandeira)			// Protocolos obrigatório (todas as bandeiras)	// Protocolos variáveis (recomendados)		// Restrições adicionais
Grupo	CNAE (2 díg.)	Tipo	Subtipos	Teto de Operação (percentual máx. de trabalhadores presentes no turno, ao mesmo tempo, respeitando o teto de ocupação do espaço físico - máx. pessoas)	Modo de Operação (forma de operação, respeitando o teto de operação e o teto de ocupação do espaço físico - máx. pessoas)<br>Trabalhadores	Atendimento	Informativo visível (operação e ocupação) Máscara / EPIs, Distanciamento, Teto de ocupação, Higienização, Proteção de grupo de risco, Afastamento de casos, Cuidados no atendimento ao	Monitora- mento de tempera- tura	Testagem dos trabalha- dores	Normas obrigatórias específicas à atividade https://coronavirus.rs.gov.br/portarias-da-ses
Indústria de Transformação e Extrativa	16	Madeira		100% trabalhadores	Teletrabalho / Presencial restrito / Ventilação cruzada (portas e janelas abertas) e/ou sistema de renovação de ar / Restaurantes, bares, lanchonetes e espaços coletivos de alimentação: conforme protocolo de "Restaurantes" e "Lanchonetes" e Portaria SES nº 319 /	Uso obrigatório e correto de máscara, cobrindo boca e nariz por todos os presentes/ Distanciamento interpessoal mínimo de 1m /	X			Portaria SES nº 283 e nº 375
Indústria de Transformação e Extrativa	17	Papel e Celulose		100% trabalhadores	Teletrabalho / Presencial restrito / Ventilação cruzada (portas e janelas abertas) e/ou sistema de renovação de ar / Restaurantes, bares, lanchonetes e espaços coletivos de alimentação: conforme protocolo de "Restaurantes" e "Lanchonetes" e Portaria SES nº 319 /	Uso obrigatório e correto de máscara, cobrindo boca e nariz por todos os presentes/ Distanciamento interpessoal mínimo de 1m /	X			Portaria SES nº 283 e nº 375
Indústria de Transformação e Extrativa	18	Impressão e Reprodução		100% trabalhadores	Teletrabalho / Presencial restrito / Ventilação cruzada (portas e janelas abertas) e/ou sistema de renovação de ar / Restaurantes, bares, lanchonetes e espaços coletivos de alimentação: conforme protocolo de "Restaurantes" e "Lanchonetes" e Portaria SES nº 319 /	Uso obrigatório e correto de máscara, cobrindo boca e nariz por todos os presentes/ Distanciamento interpessoal mínimo de 1m /	X			Portaria SES nº 283 e nº 375
Indústria de Transformação e Extrativa	19	Derivados Petróleo		100% trabalhadores	Teletrabalho / Presencial restrito / Ventilação cruzada (portas e janelas abertas) e/ou sistema de renovação de ar / Restaurantes, bares, lanchonetes e espaços coletivos de alimentação: conforme protocolo de "Restaurantes" e "Lanchonetes" e Portaria SES nº 319 /	Uso obrigatório e correto de máscara, cobrindo boca e nariz por todos os presentes/ Distanciamento interpessoal mínimo de 1m /	X			Portaria SES nº 283 e nº 375
Indústria de Transformação e Extrativa	20	Químicos		100% trabalhadores	Teletrabalho / Presencial restrito / Ventilação cruzada (portas e janelas abertas) e/ou sistema de renovação de ar / Restaurantes, bares, lanchonetes e espaços coletivos de alimentação: conforme protocolo de "Restaurantes" e "Lanchonetes" e Portaria SES nº 319 /	Uso obrigatório e correto de máscara, cobrindo boca e nariz por todos os presentes/ Distanciamento interpessoal mínimo de 1m /	X			Portaria SES nº 283 e nº 375
Indústria de Transformação e Extrativa	22	Borracha e Plástico		100% trabalhadores	Teletrabalho / Presencial restrito / Ventilação cruzada (portas e janelas abertas) e/ou sistema de renovação de ar / Restaurantes, bares, lanchonetes e espaços coletivos de alimentação: conforme protocolo de "Restaurantes" e "Lanchonetes" e Portaria SES nº 319 /	Uso obrigatório e correto de máscara, cobrindo boca e nariz por todos os presentes/ Distanciamento interpessoal mínimo de 1m /	X			Portaria SES nº 283 e nº 375

Notas:
(*) Representam agregações de atividades 2 dígitos:
100* = 6, 7, 8, 9

BANDEIRA LARANJA - Indústria

// Atividade				// Critérios específicos de funcionamento (conforme bandeira)			// Protocolos obrigatório (todas as bandeiras)	// Protocolos variáveis (recomendados)		// Restrições adicionais
Grupo	CNAE (2 díg.)	Tipo	Subtipos	Teto de Operação<br>(percentual máx. de trabalhadores presentes no turno, ao mesmo tempo,<br>respeitando o teto de ocupação do espaço físico - máx. pessoas)	Modo de Operação<br>(forma de operação, respeitando o teto de operação e o teto de ocupação do espaço físico - máx. pessoas)<br>Trabalhadores	Atendimento	Informativo visível (operação e ocupação)<br>Máscara / EPIs,<br>Distanciamento,<br>Teto de ocupação,<br>Higienização,<br>Proteção de grupo de risco,<br>Afastamento de casos,<br>Cuidados no atendimento ao	Monitora- mento de tempera- tura	Testagem dos trabalha- dores	Normas obrigatórias específicas à atividade<br>https://coronavirus.rs.gov.br/portarias-da-ses
Indústria de Transformação e Extrativa	23	Minerais não metálicos		100% trabalhadores	Teletrabalho /<br>Presencial restrito /<br>Ventilação cruzada (portas e janelas abertas) e/ou sistema de renovação de ar /<br><br>Restaurantes, bares, lanchonetes e espaços coletivos de alimentação: conforme protocolo de "Restaurantes" e "Lanchonetes" e Portaria SES nº 319 /	Uso obrigatório e correto de máscara, cobrindo boca e nariz por todos os presentes/<br><br>Distanciamento interpessoal mínimo de 1m /	X			Portaria SES nº 283 e nº 375
Indústria de Transformação e Extrativa	24	Metalurgia		100% trabalhadores	Teletrabalho /<br>Presencial restrito /<br>Ventilação cruzada (portas e janelas abertas) e/ou sistema de renovação de ar /<br><br>Restaurantes, bares, lanchonetes e espaços coletivos de alimentação: conforme protocolo de "Restaurantes" e "Lanchonetes" e Portaria SES nº 319 /	Uso obrigatório e correto de máscara, cobrindo boca e nariz por todos os presentes/<br><br>Distanciamento interpessoal mínimo de 1m /	X			Portaria SES nº 283 e nº 375
Indústria de Transformação e Extrativa	25	Produtos de Metal		100% trabalhadores	Teletrabalho /<br>Presencial restrito /<br>Ventilação cruzada (portas e janelas abertas) e/ou sistema de renovação de ar /<br><br>Restaurantes, bares, lanchonetes e espaços coletivos de alimentação: conforme protocolo de "Restaurantes" e "Lanchonetes" e Portaria SES nº 319 /	Uso obrigatório e correto de máscara, cobrindo boca e nariz por todos os presentes/<br><br>Distanciamento interpessoal mínimo de 1m /	X			Portaria SES nº 283 e nº 375
Indústria de Transformação e Extrativa	26	Equip. Informática		100% trabalhadores	Teletrabalho /<br>Presencial restrito /<br>Ventilação cruzada (portas e janelas abertas) e/ou sistema de renovação de ar /<br><br>Restaurantes, bares, lanchonetes e espaços coletivos de alimentação: conforme protocolo de "Restaurantes" e "Lanchonetes" e Portaria SES nº 319 /	Uso obrigatório e correto de máscara, cobrindo boca e nariz por todos os presentes/<br><br>Distanciamento interpessoal mínimo de 1m /	X			Portaria SES nº 283 e nº 375
Indústria de Transformação e Extrativa	27	Materiais Elétricos		100% trabalhadores	Teletrabalho /<br>Presencial restrito /<br>Ventilação cruzada (portas e janelas abertas) e/ou sistema de renovação de ar /<br><br>Restaurantes, bares, lanchonetes e espaços coletivos de alimentação: conforme protocolo de "Restaurantes" e "Lanchonetes" e Portaria SES nº 319 /	Uso obrigatório e correto de máscara, cobrindo boca e nariz por todos os presentes/<br><br>Distanciamento interpessoal mínimo de 1m /	X			Portaria SES nº 283 e nº 375
Indústria de Transformação e Extrativa	28	Máquinas e Equipamentos		100% trabalhadores	Teletrabalho /<br>Presencial restrito /<br>Ventilação cruzada (portas e janelas abertas) e/ou sistema de renovação de ar /<br><br>Restaurantes, bares, lanchonetes e espaços coletivos de alimentação: conforme protocolo de "Restaurantes" e "Lanchonetes" e Portaria SES nº 319 /	Uso obrigatório e correto de máscara, cobrindo boca e nariz por todos os presentes/<br><br>Distanciamento interpessoal mínimo de 1m /	X			Portaria SES nº 283 e nº 375

Notas:
(*) Representam agregações de atividades 2 dígitos:
100* = 6, 7, 8, 9

BANDEIRA LARANJA - Indústria

// Atividade				// Critérios específicos de funcionamento (conforme bandeira)			// Protocolos obrigatório (todas as bandeiras)	// Protocolos variáveis (recomendados)		// Restrições adicionais
Grupo	CNAE (2 díg.)	Tipo	Subtipos	Teto de Operação (percentual máx. de trabalhadores presentes no turno, ao mesmo tempo, respeitando o teto de ocupação do espaço físico - máx. pessoas)	Modo de Operação (forma de operação, respeitando o teto de operação e o teto de ocupação do espaço físico - máx. pessoas)<br>Trabalhadores	Atendimento	Informativo visível (operação e ocupação) Máscara / EPIs, Distanciamento, Teto de ocupação, Higienização, Proteção de grupo de risco, Afastamento de casos, Cuidados no atendimento ao	Monitora- mento de tempera- tura	Testagem dos trabalha- dores	Normas obrigatórias específicas à atividade https://coronavirus.rs.gov.br/portarias-da-ses
Indústria de Transformação e Extrativa	29	Veículos Automotores		100% trabalhadores	Teletrabalho / Presencial restrito / Ventilação cruzada (portas e janelas abertas) e/ou sistema de renovação de ar / Restaurantes, bares, lanchonetes e espaços coletivos de alimentação: conforme protocolo de "Restaurantes" e "Lanchonetes" e Portaria SES nº 319 /	Uso obrigatório e correto de máscara, cobrindo boca e nariz por todos os presentes/ Distanciamento interpessoal mínimo de 1m /	X			Portaria SES nº 283 e nº 375
Indústria de Transformação e Extrativa	30	Outros Equipamentos		100% trabalhadores	Teletrabalho / Presencial restrito / Ventilação cruzada (portas e janelas abertas) e/ou sistema de renovação de ar / Restaurantes, bares, lanchonetes e espaços coletivos de alimentação: conforme protocolo de "Restaurantes" e "Lanchonetes" e Portaria SES nº 319 /	Uso obrigatório e correto de máscara, cobrindo boca e nariz por todos os presentes/ Distanciamento interpessoal mínimo de 1m /	X			Portaria SES nº 283 e nº 375
Indústria de Transformação e Extrativa	31	Móveis		100% trabalhadores	Teletrabalho / Presencial restrito / Ventilação cruzada (portas e janelas abertas) e/ou sistema de renovação de ar / Restaurantes, bares, lanchonetes e espaços coletivos de alimentação: conforme protocolo de "Restaurantes" e "Lanchonetes" e Portaria SES nº 319 /	Uso obrigatório e correto de máscara, cobrindo boca e nariz por todos os presentes/ Distanciamento interpessoal mínimo de 1m /	X			Portaria SES nº 283 e nº 375
Indústria de Transformação e Extrativa	32	Produtos Diversos		100% trabalhadores	Teletrabalho / Presencial restrito / Ventilação cruzada (portas e janelas abertas) e/ou sistema de renovação de ar / Restaurantes, bares, lanchonetes e espaços coletivos de alimentação: conforme protocolo de "Restaurantes" e "Lanchonetes" e Portaria SES nº 319 /	Uso obrigatório e correto de máscara, cobrindo boca e nariz por todos os presentes/ Distanciamento interpessoal mínimo de 1m /	X			Portaria SES nº 283 e nº 375
Indústria de Transformação e Extrativa	33	Manut. e Reparação		100% trabalhadores	Teletrabalho / Presencial restrito / Ventilação cruzada (portas e janelas abertas) e/ou sistema de renovação de ar / Restaurantes, bares, lanchonetes e espaços coletivos de alimentação: conforme protocolo de "Restaurantes" e "Lanchonetes" e Portaria SES nº 319 /	Uso obrigatório e correto de máscara, cobrindo boca e nariz por todos os presentes/ Distanciamento interpessoal mínimo de 1m /	X			Portaria SES nº 283 e nº 375
Indústria de Transformação e Extrativa	21	Farmoquímicos e Farmacêuticos		100% trabalhadores	Teletrabalho / Presencial restrito / Ventilação cruzada (portas e janelas abertas) e/ou sistema de renovação de ar / Restaurantes, bares, lanchonetes e espaços coletivos de alimentação: conforme protocolo de "Restaurantes" e "Lanchonetes" e Portaria SES nº 319 /	Uso obrigatório e correto de máscara, cobrindo boca e nariz por todos os presentes/ Distanciamento interpessoal mínimo de 1m /	X			Portaria SES nº 283 e nº 375

Notas:
(*) Representam agregações de atividades 2 dígitos:
100* = 6, 7, 8, 9

BANDEIRA VERMELHA - Indústria

// Atividade				// Critérios específicos de funcionamento (conforme bandeira)			// Protocolos obrigatório (todas as bandeiras)	// Protocolos variáveis (recomendados)		// Restrições adicionais
Grupo	CNAE (2 díg.)	Tipo	Subtipos	Teto de Operação (percentual máx. de trabalhadores presentes no turno, ao mesmo tempo, respeitando o teto de ocupação do espaço físico - máx. pessoas)	Modo de Operação (forma de operação, respeitando o teto de operação e o teto de ocupação do espaço físico - máx. pessoas)<br>Trabalhadores	Atendimento	Informativo visível (operação e ocupação) Máscara / EPIs, Distanciamento, Teto de ocupação, Higienização, Proteção de grupo de risco, Afastamento de casos, Cuidados no atendimento ao público, Atendimento do grupos de risco	Monitora- mento de tempera- tura	Testagem dos trabalha- dores	Normas obrigatórias específicas à atividade https://coronavirus.rs.gov.br/portarias-da-ses
Indústria de Construção	41	Construção de Edifícios		75% trabalhadores	Teletrabalho / Presencial restrito / Ventilação cruzada (portas e janelas abertas) e/ou sistema de renovação de ar / Restaurantes, bares, lanchonetes e espaços coletivos de alimentação: conforme protocolo de "Restaurantes" e "Lanchonetes" e Portaria SES nº 319 /	Uso obrigatório e correto de máscara, cobrindo boca e nariz por todos os presentes/ Distanciamento interpessoal mínimo de 1m /	X	X		Portaria SES nº 283 e nº 375
Indústria de Construção	42	Obras de Infraestrutura		75% trabalhadores	Teletrabalho / Presencial restrito / Ventilação cruzada (portas e janelas abertas) e/ou sistema de renovação de ar / Restaurantes, bares, lanchonetes e espaços coletivos de alimentação: conforme protocolo de "Restaurantes" e "Lanchonetes" e Portaria SES nº 319 /	Uso obrigatório e correto de máscara, cobrindo boca e nariz por todos os presentes/ Distanciamento interpessoal mínimo de 1m /	X	X		Portaria SES nº 283 e nº 375
Indústria de Construção	43	Serviços de Construção		75% trabalhadores	Teletrabalho / Presencial restrito / Ventilação cruzada (portas e janelas abertas) e/ou sistema de renovação de ar / Restaurantes, bares, lanchonetes e espaços coletivos de alimentação: conforme protocolo de "Restaurantes" e "Lanchonetes" e Portaria SES nº 319 /	Uso obrigatório e correto de máscara, cobrindo boca e nariz por todos os presentes/ Distanciamento interpessoal mínimo de 1m /	X	X		Portaria SES nº 283 e nº 375
Indústria de Transformação e Extrativa	5	Extração de Carvão Mineral		75% trabalhadores	Teletrabalho / Presencial restrito / Ventilação cruzada (portas e janelas abertas) e/ou sistema de renovação de ar / Restaurantes, bares, lanchonetes e espaços coletivos de alimentação: conforme protocolo de "Restaurantes" e "Lanchonetes" e Portaria SES nº 319 /	Uso obrigatório e correto de máscara, cobrindo boca e nariz por todos os presentes/ Distanciamento interpessoal mínimo de 1m /	X	X		Portaria SES nº 283 e nº 375
Indústria de Transformação e Extrativa	100*	Extr. de Petróleo e Minerais	Extração de Petróleo e Gás	75% trabalhadores	Teletrabalho / Presencial restrito / Ventilação cruzada (portas e janelas abertas) e/ou sistema de renovação de ar / Restaurantes, bares, lanchonetes e espaços coletivos de alimentação: conforme protocolo de "Restaurantes" e "Lanchonetes" e Portaria SES nº 319 /	Uso obrigatório e correto de máscara, cobrindo boca e nariz por todos os presentes/ Distanciamento interpessoal mínimo de 1m /	X	X		Portaria SES nº 283 e nº 375
Indústria de Transformação e Extrativa	100*	Extr. de Petróleo e Minerais	Extr. de Petróleo e Minerais - Outros	75% trabalhadores	Teletrabalho / Presencial restrito / Ventilação cruzada (portas e janelas abertas) e/ou sistema de renovação de ar / Restaurantes, bares, lanchonetes e espaços coletivos de alimentação: conforme protocolo de "Restaurantes" e "Lanchonetes" e Portaria SES nº 319 /	Uso obrigatório e correto de máscara, cobrindo boca e nariz por todos os presentes/ Distanciamento interpessoal mínimo de 1m /	X	X		Portaria SES nº 283 e nº 375

Notas:
(*) Representam agregações de atividades 2 dígitos:
100* = 6, 7, 8, 9

BANDEIRA VERMELHA - Indústria

// Atividade				// Critérios específicos de funcionamento (conforme bandeira)			// Protocolos obrigatório (todas as bandeiras)	// Protocolos variáveis (recomendados)		// Restrições adicionais
Grupo	CNAE (2 díg.)	Tipo	Subtipos	Teto de Operação (percentual máx. de trabalhadores presentes no turno, ao mesmo tempo, respeitando o teto de ocupação do espaço físico - máx. pessoas)	Modo de Operação (forma de operação, respeitando o teto de operação e o teto de ocupação do espaço físico - máx. pessoas) Trabalhadores	Atendimento	Informativo visível (operação e ocupação) Máscara / EPIs, Distanciamento, Teto de ocupação, Higienização, Proteção de grupo de risco, Afastamento de casos, Cuidados no atendimento ao público, Atendimento do grupos de risco	Monitora-mento de tempera- tura	Testagem dos trabalha- dores	Normas obrigatórias específicas à atividade https://coronavirus.rs.gov.br/portarias-da-ses
Indústria de Transformação e Extrativa	10	Alimentos		75% trabalhadores	Teletrabalho / Presencial restrito / Ventilação cruzada (portas e janelas abertas) e/ou sistema de renovação de ar / Restaurantes, bares, lanchonetes e espaços coletivos de alimentação: conforme protocolo de "Restaurantes" e "Lanchonetes" e Portaria SES nº 319 /	Uso obrigatório e correto de máscara, cobrindo boca e nariz por todos os presentes/ Distanciamento interpessoal mínimo de 1m /	X	X		Portaria SES nº 283 e nº 375
Indústria de Transformação e Extrativa	11	Bebidas		75% trabalhadores	Teletrabalho / Presencial restrito / Ventilação cruzada (portas e janelas abertas) e/ou sistema de renovação de ar / Restaurantes, bares, lanchonetes e espaços coletivos de alimentação: conforme protocolo de "Restaurantes" e "Lanchonetes" e Portaria SES nº 319 /	Uso obrigatório e correto de máscara, cobrindo boca e nariz por todos os presentes/ Distanciamento interpessoal mínimo de 1m /	X	X		Portaria SES nº 283 e nº 375
Indústria de Transformação e Extrativa	12	Fumo		75% trabalhadores	Teletrabalho / Presencial restrito / Ventilação cruzada (portas e janelas abertas) e/ou sistema de renovação de ar / Restaurantes, bares, lanchonetes e espaços coletivos de alimentação: conforme protocolo de "Restaurantes" e "Lanchonetes" e Portaria SES nº 319 /	Uso obrigatório e correto de máscara, cobrindo boca e nariz por todos os presentes/ Distanciamento interpessoal mínimo de 1m /	X	X		Portaria SES nº 283 e nº 375
Indústria de Transformação e Extrativa	13	Têxteis		75% trabalhadores	Teletrabalho / Presencial restrito / Ventilação cruzada (portas e janelas abertas) e/ou sistema de renovação de ar / Restaurantes, bares, lanchonetes e espaços coletivos de alimentação: conforme protocolo de "Restaurantes" e "Lanchonetes" e Portaria SES nº 319 /	Uso obrigatório e correto de máscara, cobrindo boca e nariz por todos os presentes/ Distanciamento interpessoal mínimo de 1m /	X	X		Portaria SES nº 283 e nº 375
Indústria de Transformação e Extrativa	14	Vestuário		75% trabalhadores	Teletrabalho / Presencial restrito / Ventilação cruzada (portas e janelas abertas) e/ou sistema de renovação de ar / Restaurantes, bares, lanchonetes e espaços coletivos de alimentação: conforme protocolo de "Restaurantes" e "Lanchonetes" e Portaria SES nº 319 /	Uso obrigatório e correto de máscara, cobrindo boca e nariz por todos os presentes/ Distanciamento interpessoal mínimo de 1m /	X	X		Portaria SES nº 283 e nº 375
Indústria de Transformação e Extrativa	15	Couros e Calçados		75% trabalhadores	Teletrabalho / Presencial restrito / Ventilação cruzada (portas e janelas abertas) e/ou sistema de renovação de ar / Restaurantes, bares, lanchonetes e espaços coletivos de alimentação: conforme protocolo de "Restaurantes" e "Lanchonetes" e Portaria SES nº 319 /	Uso obrigatório e correto de máscara, cobrindo boca e nariz por todos os presentes/ Distanciamento interpessoal mínimo de 1m /	X	X		Portaria SES nº 283 e nº 375

DISTANCIAMENTO CONTROLADO
GOV RS
NOVAS FACANHAS

Notas:
(*) Representam agregações de atividades 2 dígitos:
100* = 6, 7, 8, 9

BANDEIRA VERMELHA - Indústria

// Atividade				// Critérios específicos de funcionamento (conforme bandeira)			// Protocolos obrigatório (todas as bandeiras)	// Protocolos variáveis (recomendados)		// Restrições adicionais
Grupo	CNAE (2 díg.)	Tipo	Subtipos	Teto de Operação (percentual máx. de trabalhadores presentes no turno, ao mesmo tempo, respeitando o teto de ocupação do espaço físico - máx. pessoas)	Modo de Operação (forma de operação, respeitando o teto de operação e o teto de ocupação do espaço físico - máx. pessoas)<br>Trabalhadores	Atendimento	Informativo visível (operação e ocupação) Máscara / EPIs, Distanciamento, Teto de ocupação, Higienização, Proteção de grupo de risco, Afastamento de casos, Cuidados no atendimento ao público, Atendimento do grupos de risco	Monitora- mento de tempera- tura	Testagem dos trabalha- dores	Normas obrigatórias específicas à atividade https://coronavirus.rs.gov.br/portarias-da-ses
Indústria de Transformação e Extrativa	16	Madeira		75% trabalhadores	Teletrabalho / Presencial restrito / Ventilação cruzada (portas e janelas abertas) e/ou sistema de renovação de ar / Restaurantes, bares, lanchonetes e espaços coletivos de alimentação: conforme protocolo de "Restaurantes" e "Lanchonetes" e Portaria SES nº 319 /	Uso obrigatório e correto de máscara, cobrindo boca e nariz por todos os presentes/ Distanciamento interpessoal mínimo de 1m /	X	X		Portaria SES nº 283 e nº 375
Indústria de Transformação e Extrativa	17	Papel e Celulose		75% trabalhadores	Teletrabalho / Presencial restrito / Ventilação cruzada (portas e janelas abertas) e/ou sistema de renovação de ar / Restaurantes, bares, lanchonetes e espaços coletivos de alimentação: conforme protocolo de "Restaurantes" e "Lanchonetes" e Portaria SES nº 319 /	Uso obrigatório e correto de máscara, cobrindo boca e nariz por todos os presentes/ Distanciamento interpessoal mínimo de 1m /	X	X		Portaria SES nº 283 e nº 375
Indústria de Transformação e Extrativa	18	Impressão e Reprodução		75% trabalhadores	Teletrabalho / Presencial restrito / Ventilação cruzada (portas e janelas abertas) e/ou sistema de renovação de ar / Restaurantes, bares, lanchonetes e espaços coletivos de alimentação: conforme protocolo de "Restaurantes" e "Lanchonetes" e Portaria SES nº 319 /	Uso obrigatório e correto de máscara, cobrindo boca e nariz por todos os presentes/ Distanciamento interpessoal mínimo de 1m /	X	X		Portaria SES nº 283 e nº 375
Indústria de Transformação e Extrativa	19	Derivados Petróleo		75% trabalhadores	Teletrabalho / Presencial restrito / Ventilação cruzada (portas e janelas abertas) e/ou sistema de renovação de ar / Restaurantes, bares, lanchonetes e espaços coletivos de alimentação: conforme protocolo de "Restaurantes" e "Lanchonetes" e Portaria SES nº 319 /	Uso obrigatório e correto de máscara, cobrindo boca e nariz por todos os presentes/ Distanciamento interpessoal mínimo de 1m /	X	X		Portaria SES nº 283 e nº 375
Indústria de Transformação e Extrativa	20	Químicos		75% trabalhadores	Teletrabalho / Presencial restrito / Ventilação cruzada (portas e janelas abertas) e/ou sistema de renovação de ar / Restaurantes, bares, lanchonetes e espaços coletivos de alimentação: conforme protocolo de "Restaurantes" e "Lanchonetes" e Portaria SES nº 319 /	Uso obrigatório e correto de máscara, cobrindo boca e nariz por todos os presentes/ Distanciamento interpessoal mínimo de 1m /	X	X		Portaria SES nº 283 e nº 375
Indústria de Transformação e Extrativa	22	Borracha e Plástico		100% trabalhadores	Teletrabalho / Presencial restrito / Ventilação cruzada (portas e janelas abertas) e/ou sistema de renovação de ar / Restaurantes, bares, lanchonetes e espaços coletivos de alimentação: conforme protocolo de "Restaurantes" e "Lanchonetes" e Portaria SES nº 319 /	Uso obrigatório e correto de máscara, cobrindo boca e nariz por todos os presentes/ Distanciamento interpessoal mínimo de 1m /	X	X		Portaria SES nº 283 e nº 375

Notas:
(*) Representam agregações de atividades 2 dígitos:
100* = 6, 7, 8, 9

BANDEIRA VERMELHA - Indústria

// Atividade				// Critérios específicos de funcionamento (conforme bandeira)			// Protocolos obrigatório (todas as bandeiras)	// Protocolos variáveis (recomendados)		// Restrições adicionais
Grupo	CNAE (2 díg.)	Tipo	Subtipos	Teto de Operação (percentual máx. de trabalhadores presentes no turno, ao mesmo tempo, respeitando o teto de ocupação do espaço físico - máx. pessoas)	Modo de Operação (forma de operação, respeitando o teto de operação e o teto de ocupação do espaço físico - máx. pessoas)<br>Trabalhadores	Atendimento	Informativo visível (operação e ocupação) Máscara / EPIs, Distanciamento, Teto de ocupação, Higienização, Proteção de grupo de risco, Afastamento de casos, Cuidados no atendimento ao público, Atendimento do grupos de risco	Monitora- mento de tempera- tura	Testagem dos trabalha- dores	Normas obrigatórias específicas à atividade https://coronavirus.rs.gov.br/portarias-da-ses
Indústria de Transformação e Extrativa	23	Minerais não metálicos		75% trabalhadores	Teletrabalho / Presencial restrito / Ventilação cruzada (portas e janelas abertas) e/ou sistema de renovação de ar / Restaurantes, bares, lanchonetes e espaços coletivos de alimentação: conforme protocolo de "Restaurantes" e "Lanchonetes" e Portaria SES nº 319 /	Uso obrigatório e correto de máscara, cobrindo boca e nariz por todos os presentes/ Distanciamento interpessoal mínimo de 1m /	X	X		Portaria SES nº 283 e nº 375
Indústria de Transformação e Extrativa	24	Metalurgia		75% trabalhadores	Teletrabalho / Presencial restrito / Ventilação cruzada (portas e janelas abertas) e/ou sistema de renovação de ar / Restaurantes, bares, lanchonetes e espaços coletivos de alimentação: conforme protocolo de "Restaurantes" e "Lanchonetes" e Portaria SES nº 319 /	Uso obrigatório e correto de máscara, cobrindo boca e nariz por todos os presentes/ Distanciamento interpessoal mínimo de 1m /	X	X		Portaria SES nº 283 e nº 375
Indústria de Transformação e Extrativa	25	Produtos de Metal		75% trabalhadores	Teletrabalho / Presencial restrito / Ventilação cruzada (portas e janelas abertas) e/ou sistema de renovação de ar / Restaurantes, bares, lanchonetes e espaços coletivos de alimentação: conforme protocolo de "Restaurantes" e "Lanchonetes" e Portaria SES nº 319 /	Uso obrigatório e correto de máscara, cobrindo boca e nariz por todos os presentes/ Distanciamento interpessoal mínimo de 1m /	X	X		Portaria SES nº 283 e nº 375
Indústria de Transformação e Extrativa	26	Equip. Informática		75% trabalhadores	Teletrabalho / Presencial restrito / Ventilação cruzada (portas e janelas abertas) e/ou sistema de renovação de ar / Restaurantes, bares, lanchonetes e espaços coletivos de alimentação: conforme protocolo de "Restaurantes" e "Lanchonetes" e Portaria SES nº 319 /	Uso obrigatório e correto de máscara, cobrindo boca e nariz por todos os presentes/ Distanciamento interpessoal mínimo de 1m /	X	X		Portaria SES nº 283 e nº 375
Indústria de Transformação e Extrativa	27	Materiais Elétricos		75% trabalhadores	Teletrabalho / Presencial restrito / Ventilação cruzada (portas e janelas abertas) e/ou sistema de renovação de ar / Restaurantes, bares, lanchonetes e espaços coletivos de alimentação: conforme protocolo de "Restaurantes" e "Lanchonetes" e Portaria SES nº 319 /	Uso obrigatório e correto de máscara, cobrindo boca e nariz por todos os presentes/ Distanciamento interpessoal mínimo de 1m /	X	X		Portaria SES nº 283 e nº 375
Indústria de Transformação e Extrativa	28	Máquinas e Equipamentos		75% trabalhadores	Teletrabalho / Presencial restrito / Ventilação cruzada (portas e janelas abertas) e/ou sistema de renovação de ar / Restaurantes, bares, lanchonetes e espaços coletivos de alimentação: conforme protocolo de "Restaurantes" e "Lanchonetes" e Portaria SES nº 319 /	Uso obrigatório e correto de máscara, cobrindo boca e nariz por todos os presentes/ Distanciamento interpessoal mínimo de 1m /	X	X		Portaria SES nº 283 e nº 375

Notas:
(*) Representam agregações de atividades 2 dígitos:
100* = 6, 7, 8, 9

BANDEIRA VERMELHA - Indústria

// Atividade				// Critérios específicos de funcionamento (conforme bandeira)			// Protocolos obrigatório (todas as bandeiras)	// Protocolos variáveis (recomendados)		// Restrições adicionais
Grupo	CNAE (2 díg.)	Tipo	Subtipos	Teto de Operação (percentual máx. de trabalhadores presentes no turno, ao mesmo tempo, respeitando o teto de ocupação do espaço físico - máx. pessoas)	Modo de Operação (forma de operação, respeitando o teto de operação e o teto de ocupação do espaço físico - máx. pessoas) Trabalhadores	Atendimento	Informativo visível (operação e ocupação) Máscara / EPIs, Distanciamento, Teto de ocupação, Higienização, Proteção de grupo de risco, Afastamento de casos, Cuidados no atendimento ao público, Atendimento do grupos de risco	Monitora- mento de tempera- tura	Testagem dos trabalha- dores	Normas obrigatórias específicas à atividade https://coronavirus.rs.gov.br/portarias-da-ses
Indústria de Transformação e Extrativa	29	Veículos Automotores		75% trabalhadores	Teletrabalho / Presencial restrito / Ventilação cruzada (portas e janelas abertas) e/ou sistema de renovação de ar / Restaurantes, bares, lanchonetes e espaços coletivos de alimentação: conforme protocolo de "Restaurantes" e "Lanchonetes" e Portaria SES nº 319 /	Uso obrigatório e correto de máscara, cobrindo boca e nariz por todos os presentes/ Distanciamento interpessoal mínimo de 1m /	X	X		Portaria SES nº 283 e nº 375
Indústria de Transformação e Extrativa	30	Outros Equipamentos		75% trabalhadores	Teletrabalho / Presencial restrito / Ventilação cruzada (portas e janelas abertas) e/ou sistema de renovação de ar / Restaurantes, bares, lanchonetes e espaços coletivos de alimentação: conforme protocolo de "Restaurantes" e "Lanchonetes" e Portaria SES nº 319 /	Uso obrigatório e correto de máscara, cobrindo boca e nariz por todos os presentes/ Distanciamento interpessoal mínimo de 1m /	X	X		Portaria SES nº 283 e nº 375
Indústria de Transformação e Extrativa	31	Móveis		75% trabalhadores	Teletrabalho / Presencial restrito / Ventilação cruzada (portas e janelas abertas) e/ou sistema de renovação de ar / Restaurantes, bares, lanchonetes e espaços coletivos de alimentação: conforme protocolo de "Restaurantes" e "Lanchonetes" e Portaria SES nº 319 /	Uso obrigatório e correto de máscara, cobrindo boca e nariz por todos os presentes/ Distanciamento interpessoal mínimo de 1m /	X	X		Portaria SES nº 283 e nº 375
Indústria de Transformação e Extrativa	32	Produtos Diversos		75% trabalhadores	Teletrabalho / Presencial restrito / Ventilação cruzada (portas e janelas abertas) e/ou sistema de renovação de ar / Restaurantes, bares, lanchonetes e espaços coletivos de alimentação: conforme protocolo de "Restaurantes" e "Lanchonetes" e Portaria SES nº 319 /	Uso obrigatório e correto de máscara, cobrindo boca e nariz por todos os presentes/ Distanciamento interpessoal mínimo de 1m /	X	X		Portaria SES nº 283 e nº 375
Indústria de Transformação e Extrativa	33	Manut. e Reparação		75% trabalhadores	Teletrabalho / Presencial restrito / Ventilação cruzada (portas e janelas abertas) e/ou sistema de renovação de ar / Restaurantes, bares, lanchonetes e espaços coletivos de alimentação: conforme protocolo de "Restaurantes" e "Lanchonetes" e Portaria SES nº 319 /	Uso obrigatório e correto de máscara, cobrindo boca e nariz por todos os presentes/ Distanciamento interpessoal mínimo de 1m /	X	X		Portaria SES nº 283 e nº 375
Indústria de Transformação e Extrativa	21	Farmoquímicos e Farmacêuticos		100% trabalhadores	Teletrabalho / Presencial restrito / Ventilação cruzada (portas e janelas abertas) e/ou sistema de renovação de ar / Restaurantes, bares, lanchonetes e espaços coletivos de alimentação: conforme protocolo de "Restaurantes" e "Lanchonetes" e Portaria SES nº 319 /	Uso obrigatório e correto de máscara, cobrindo boca e nariz por todos os presentes/ Distanciamento interpessoal mínimo de 1m /	X	X		Portaria SES nº 283 e nº 375

DISTANCIAMENTO CONTROLADO
GOV RS
NOVAS FACANHAS

Notas:
(*) Representam agregações de atividades 2 dígitos:
100* = 6, 7, 8, 9

BANDEIRA PRETA - Indústria

// Atividade				// Critérios específicos de funcionamento (conforme bandeira)			// Protocolos obrigatório (todas as bandeiras)	// Protocolos variáveis (recomendados)		// Restrições adicionais
Grupo	CNAE (2 díg.)	Tipo	Subtipos	Teto de Operação (percentual máx. de trabalhadores presentes no turno, ao mesmo tempo, respeitando o teto de ocupação do espaço físico - máx. pessoas)	Modo de Operação (forma de operação, respeitando o teto de operação e o teto de ocupação do espaço físico - máx. pessoas)<br>Trabalhadores	Atendimento	Informativo visível (operação e ocupação) Máscara / EPIs, Distanciamento, Teto de ocupação, Higienização, Proteção de grupo de risco, Afastamento de casos, Cuidados no atendimento ao público, Atendimento do grupos de risco	Monitora- mento de tempera- tura	Testagem dos trabalha- dores	Normas obrigatórias específicas à atividade https://coronavirus.rs.gov.br/portarias-da-ses
Indústria de Construção	41	Construção de Edifícios		75% trabalhadores	Teletrabalho / Presencial restrito (exclusivo emergência Covid-19) / Ventilação cruzada (portas e janelas abertas) e/ou sistema de renovação de ar / Restaurantes, bares, lanchonetes e espaços coletivos de alimentação: conforme protocolo de "Restaurantes" e "Lanchonetes" e Portaria SES nº 319 /	Uso obrigatório e correto de máscara, cobrindo boca e nariz por todos os presentes/ Distanciamento interpessoal mínimo de 1m /	X	X		Portaria SES nº 283 e nº 375
Indústria de Construção	42	Obras de Infraestrutura		75% trabalhadores	Teletrabalho / Presencial restrito (exclusivo emergência Covid-19) / Ventilação cruzada (portas e janelas abertas) e/ou sistema de renovação de ar / Restaurantes, bares, lanchonetes e espaços coletivos de alimentação: conforme protocolo de "Restaurantes" e "Lanchonetes" e Portaria SES nº 319 /	Uso obrigatório e correto de máscara, cobrindo boca e nariz por todos os presentes/ Distanciamento interpessoal mínimo de 1m /	X	X		Portaria SES nº 283 e nº 375
Indústria de Construção	43	Serviços de Construção		75% trabalhadores	Teletrabalho / Presencial restrito (exclusivo emergência Covid-19) / Ventilação cruzada (portas e janelas abertas) e/ou sistema de renovação de ar / Restaurantes, bares, lanchonetes e espaços coletivos de alimentação: conforme protocolo de "Restaurantes" e "Lanchonetes" e Portaria SES nº 319 /	Uso obrigatório e correto de máscara, cobrindo boca e nariz por todos os presentes/ Distanciamento interpessoal mínimo de 1m /	X	X		Portaria SES nº 283 e nº 375

Notas:
(*) Representam agregações de atividades 2 dígitos:
100* = 6, 7, 8, 9

BANDEIRA PRETA - Indústria

// Atividade				// Critérios específicos de funcionamento (conforme bandeira)			// Protocolos obrigatório (todas as bandeiras)	// Protocolos variáveis (recomendados)		// Restrições adicionais
Grupo	CNAE (2 díg.)	Tipo	Subtipos	Teto de Operação (percentual máx. de trabalhadores presentes no turno, ao mesmo tempo, respeitando o teto de ocupação do espaço físico - máx. pessoas)	Modo de Operação (forma de operação, respeitando o teto de operação e o teto de ocupação do espaço físico - máx. pessoas)<br>Trabalhadores	Atendimento	Informativo visível (operação e ocupação)<br>Máscara / EPIs,<br>Distanciamento,<br>Teto de ocupação,<br>Higienização,<br>Proteção de grupo de risco,<br>Afastamento de casos,<br>Cuidados no atendimento ao público,<br>Atendimento do grupos de risco	Monitora- mento de tempera- tura	Testagem dos trabalha- dores	Normas obrigatórias específicas à atividade https://coronavirus.rs.gov.br/portarias-da-ses
Indústria de Transformação e Extrativa	5	Extração de Carvão Mineral		75% trabalhadores	Teletrabalho no máximo possível / Presencial restrito / Ventilação cruzada (portas e janelas abertas) e/ou sistema de renovação de ar /<br>Restaurantes, bares, lanchonetes e espaços coletivos de alimentação: conforme protocolo de "Restaurantes" e "Lanchonetes" e Portaria SES nº 319 /	Uso obrigatório e correto de máscara, cobrindo boca e nariz por todos os presentes/<br>Distanciamento interpessoal mínimo de 1m /	X	X		Portaria SES nº 283 e nº 375
Indústria de Transformação e Extrativa	100*	Extr. de Petróleo e Minerais	Extração de Petróleo e Gás	75% trabalhadores	Teletrabalho no máximo possível / Presencial restrito / Ventilação cruzada (portas e janelas abertas) e/ou sistema de renovação de ar /<br>Restaurantes, bares, lanchonetes e espaços coletivos de alimentação: conforme protocolo de "Restaurantes" e "Lanchonetes" e Portaria SES nº 319 /	Uso obrigatório e correto de máscara, cobrindo boca e nariz por todos os presentes/<br>Distanciamento interpessoal mínimo de 1m /	X	X		Portaria SES nº 283 e nº 375
Indústria de Transformação e Extrativa	100*	Extr. de Petróleo e Minerais	Extr. de Petróleo e Minerais - Outros	75% trabalhadores	Teletrabalho no máximo possível / Presencial restrito / Ventilação cruzada (portas e janelas abertas) e/ou sistema de renovação de ar /<br>Restaurantes, bares, lanchonetes e espaços coletivos de alimentação: conforme protocolo de "Restaurantes" e "Lanchonetes" e Portaria SES nº 319 /	Uso obrigatório e correto de máscara, cobrindo boca e nariz por todos os presentes/<br>Distanciamento interpessoal mínimo de 1m /	X	X		Portaria SES nº 283 e nº 375

Notas:
(*) Representam agregações de atividades 2 dígitos:
100* = 6, 7, 8, 9

BANDEIRA PRETA - Indústria

// Atividade				// Critérios específicos de funcionamento (conforme bandeira)			// Protocolos obrigatório (todas as bandeiras)	// Protocolos variáveis (recomendados)		// Restrições adicionais
Grupo	CNAE (2 díg.)	Tipo	Subtipos	Teto de Operação (percentual máx. de trabalhadores presentes no turno, ao mesmo tempo, respeitando o teto de ocupação do espaço físico - máx. pessoas)	Modo de Operação (forma de operação, respeitando o teto de operação e o teto de ocupação do espaço físico - máx. pessoas)<br>Trabalhadores	Atendimento	Informativo visível (operação e ocupação)<br>Máscara / EPIs,<br>Distanciamento,<br>Teto de ocupação,<br>Higienização,<br>Proteção de grupo de risco,<br>Afastamento de casos,<br>Cuidados no atendimento ao público,<br>Atendimento do grupos de risco	Monitora- mento de tempera- tura	Testagem dos trabalha- dores	Normas obrigatórias específicas à atividade<br>https://coronavirus.rs.gov.br/portarias-da-ses
Indústria de Transformação e Extrativa	10	Alimentos		75% trabalhadores	Teletrabalho no máximo possível /<br>Presencial restrito /<br>Ventilação cruzada (portas e janelas abertas) e/ou sistema de renovação de ar /<br><br>Restaurantes, bares, lanchonetes e espaços coletivos de alimentação: conforme protocolo de "Restaurantes" e "Lanchonetes" e Portaria SES nº 319 /	Uso obrigatório e correto de máscara, cobrindo boca e nariz por todos os presentes/<br><br>Distanciamento interpessoal mínimo de 1m /	X	X		Portaria SES nº 283 e nº 375
Indústria de Transformação e Extrativa	11	Bebidas		75% trabalhadores	Teletrabalho no máximo possível /<br>Presencial restrito /<br>Ventilação cruzada (portas e janelas abertas) e/ou sistema de renovação de ar /<br><br>Restaurantes, bares, lanchonetes e espaços coletivos de alimentação: conforme protocolo de "Restaurantes" e "Lanchonetes" e Portaria SES nº 319 /	Uso obrigatório e correto de máscara, cobrindo boca e nariz por todos os presentes/<br><br>Distanciamento interpessoal mínimo de 1m /	X	X		Portaria SES nº 283 e nº 375
Indústria de Transformação e Extrativa	12	Fumo		75% trabalhadores	Teletrabalho no máximo possível /<br>Presencial restrito /<br>Ventilação cruzada (portas e janelas abertas) e/ou sistema de renovação de ar /<br><br>Restaurantes, bares, lanchonetes e espaços coletivos de alimentação: conforme protocolo de "Restaurantes" e "Lanchonetes" e Portaria SES nº 319 /	Uso obrigatório e correto de máscara, cobrindo boca e nariz por todos os presentes/<br><br>Distanciamento interpessoal mínimo de 1m /	X	X		Portaria SES nº 283 e nº 375

Notas:
(*) Representam agregações de atividades 2 dígitos:
100* = 6, 7, 8, 9

BANDEIRA PRETA - Indústria

// Atividade				// Critérios específicos de funcionamento (conforme bandeira)			// Protocolos obrigatório (todas as bandeiras)	// Protocolos variáveis (recomendados)		// Restrições adicionais
Grupo	CNAE (2 díg.)	Tipo	Subtipos	Teto de Operação (percentual máx. de trabalhadores presentes no turno, ao mesmo tempo, respeitando o teto de ocupação do espaço físico - máx. pessoas)	Modo de Operação (forma de operação, respeitando o teto de operação e o teto de ocupação do espaço físico - máx. pessoas) Trabalhadores	Atendimento	Informativo visível (operação e ocupação) Máscara / EPIs, Distanciamento, Teto de ocupação, Higienização, Proteção de grupo de risco, Afastamento de casos, Cuidados no atendimento ao público, Atendimento do grupos de risco	Monitora- mento de tempera- tura	Testagem dos trabalha- dores	Normas obrigatórias específicas à atividade https://coronavirus.rs.gov.br/portarias-da-ses
Indústria de Transformação e Extrativa	13	Têxteis		75% trabalhadores	Teletrabalho no máximo possível / Presencial restrito / Ventilação cruzada (portas e janelas abertas) e/ou sistema de renovação de ar / Restaurantes, bares, lanchonetes e espaços coletivos de alimentação: conforme protocolo de "Restaurantes" e "Lanchonetes" e Portaria SES nº 319 /	Uso obrigatório e correto de máscara, cobrindo boca e nariz por todos os presentes/ Distanciamento interpessoal mínimo de 1m /	X	X		Portaria SES nº 283 e nº 375
Indústria de Transformação e Extrativa	14	Vestuário		75% trabalhadores	Teletrabalho no máximo possível / Presencial restrito / Ventilação cruzada (portas e janelas abertas) e/ou sistema de renovação de ar / Restaurantes, bares, lanchonetes e espaços coletivos de alimentação: conforme protocolo de "Restaurantes" e "Lanchonetes" e Portaria SES nº 319 /	Uso obrigatório e correto de máscara, cobrindo boca e nariz por todos os presentes/ Distanciamento interpessoal mínimo de 1m /	X	X		Portaria SES nº 283 e nº 375
Indústria de Transformação e Extrativa	15	Couros e Calçados		75% trabalhadores	Teletrabalho no máximo possível / Presencial restrito / Ventilação cruzada (portas e janelas abertas) e/ou sistema de renovação de ar / Restaurantes, bares, lanchonetes e espaços coletivos de alimentação: conforme protocolo de "Restaurantes" e "Lanchonetes" e Portaria SES nº 319 /	Uso obrigatório e correto de máscara, cobrindo boca e nariz por todos os presentes/ Distanciamento interpessoal mínimo de 1m /	X	X		Portaria SES nº 283 e nº 375

Notas:
(*) Representam agregações de atividades 2 dígitos:
100* = 6, 7, 8, 9

BANDEIRA PRETA - Indústria

// Atividade				// Critérios específicos de funcionamento (conforme bandeira)			// Protocolos obrigatório (todas as bandeiras)	// Protocolos variáveis (recomendados)		// Restrições adicionais
Grupo	CNAE (2 díg.)	Tipo	Subtipos	Teto de Operação (percentual máx. de trabalhadores presentes no turno, ao mesmo tempo, respeitando o teto de ocupação do espaço físico - máx. pessoas)	Modo de Operação (forma de operação, respeitando o teto de operação e o teto de ocupação do espaço físico - máx. pessoas)<br>Trabalhadores	Atendimento	Informativo visível (operação e ocupação) Máscara / EPIs, Distanciamento, Teto de ocupação, Higienização, Proteção de grupo de risco, Afastamento de casos, Cuidados no atendimento ao público, Atendimento do grupos de risco	Monitora- mento de tempera- tura	Testagem dos trabalha- dores	Normas obrigatórias específicas à atividade https://coronavirus.rs.gov.br/portarias-da-ses
Indústria de Transformação e Extrativa	16	Madeira		75% trabalhadores	Teletrabalho no máximo possível / Presencial restrito / Ventilação cruzada (portas e janelas abertas) e/ou sistema de renovação de ar / Restaurantes, bares, lanchonetes e espaços coletivos de alimentação: conforme protocolo de "Restaurantes" e "Lanchonetes" e Portaria SES nº 319 /	Uso obrigatório e correto de máscara, cobrindo boca e nariz por todos os presentes/ Distanciamento interpessoal mínimo de 1m /	X	X		Portaria SES nº 283 e nº 375
Indústria de Transformação e Extrativa	17	Papel e Celulose		75% trabalhadores	Teletrabalho no máximo possível / Presencial restrito / Ventilação cruzada (portas e janelas abertas) e/ou sistema de renovação de ar / Restaurantes, bares, lanchonetes e espaços coletivos de alimentação: conforme protocolo de "Restaurantes" e "Lanchonetes" e Portaria SES nº 319 /	Uso obrigatório e correto de máscara, cobrindo boca e nariz por todos os presentes/ Distanciamento interpessoal mínimo de 1m /	X	X		Portaria SES nº 283 e nº 375
Indústria de Transformação e Extrativa	18	Impressão e Reprodução		75% trabalhadores	Teletrabalho no máximo possível / Presencial restrito / Ventilação cruzada (portas e janelas abertas) e/ou sistema de renovação de ar / Restaurantes, bares, lanchonetes e espaços coletivos de alimentação: conforme protocolo de "Restaurantes" e "Lanchonetes" e Portaria SES nº 319 /	Uso obrigatório e correto de máscara, cobrindo boca e nariz por todos os presentes/ Distanciamento interpessoal mínimo de 1m /	X	X		Portaria SES nº 283 e nº 375

Notas:
(*) Representam agregações de atividades 2 dígitos:
100* = 6, 7, 8, 9

BANDEIRA PRETA - Indústria

// Atividade				// Critérios específicos de funcionamento (conforme bandeira)			// Protocolos obrigatório (todas as bandeiras)	// Protocolos variáveis (recomendados)		// Restrições adicionais
Grupo	CNAE (2 díg.)	Tipo	Subtipos	Teto de Operação (percentual máx. de trabalhadores presentes no turno, ao mesmo tempo, respeitando o teto de ocupação do espaço físico - máx. pessoas)	Modo de Operação (forma de operação, respeitando o teto de operação e o teto de ocupação do espaço físico - máx. pessoas)<br>Trabalhadores	Atendimento	Informativo visível (operação e ocupação) Máscara / EPIs, Distanciamento, Teto de ocupação, Higienização, Proteção de grupo de risco, Afastamento de casos, Cuidados no atendimento ao público, Atendimento do grupos de risco	Monitora- mento de tempera- tura	Testagem dos trabalha- dores	Normas obrigatórias específicas à atividade https://coronavirus.rs.gov.br/portarias-da-ses
Indústria de Transformação e Extrativa	19	Derivados Petróleo		75% trabalhadores	Teletrabalho no máximo possível / Presencial restrito / Ventilação cruzada (portas e janelas abertas) e/ou sistema de renovação de ar / Restaurantes, bares, lanchonetes e espaços coletivos de alimentação: conforme protocolo de "Restaurantes" e "Lanchonetes" e Portaria SES nº 319 /	Uso obrigatório e correto de máscara, cobrindo boca e nariz por todos os presentes/ Distanciamento interpessoal mínimo de 1m /	X	X		Portaria SES nº 283 e nº 375
Indústria de Transformação e Extrativa	20	Químicos		75% trabalhadores	Teletrabalho no máximo possível / Presencial restrito / Ventilação cruzada (portas e janelas abertas) e/ou sistema de renovação de ar / Restaurantes, bares, lanchonetes e espaços coletivos de alimentação: conforme protocolo de "Restaurantes" e "Lanchonetes" e Portaria SES nº 319 /	Uso obrigatório e correto de máscara, cobrindo boca e nariz por todos os presentes/ Distanciamento interpessoal mínimo de 1m /	X	X		Portaria SES nº 283 e nº 375
Indústria de Transformação e Extrativa	22	Borracha e Plástico		75% trabalhadores	Teletrabalho no máximo possível / Presencial restrito / Ventilação cruzada (portas e janelas abertas) e/ou sistema de renovação de ar / Restaurantes, bares, lanchonetes e espaços coletivos de alimentação: conforme protocolo de "Restaurantes" e "Lanchonetes" e Portaria SES nº 319 /	Uso obrigatório e correto de máscara, cobrindo boca e nariz por todos os presentes/ Distanciamento interpessoal mínimo de 1m /	X	X		Portaria SES nº 283 e nº 375

Notas:
(*) Representam agregações de atividades 2 dígitos:
100* = 6, 7, 8, 9

BANDEIRA PRETA - Indústria

// Atividade				// Critérios específicos de funcionamento (conforme bandeira)			// Protocolos obrigatório (todas as bandeiras)	// Protocolos variáveis (recomendados)		// Restrições adicionais
Grupo	CNAE (2 díg.)	Tipo	Subtipos	Teto de Operação (percentual máx. de trabalhadores presentes no turno, ao mesmo tempo, respeitando o teto de ocupação do espaço físico - máx. pessoas)	Modo de Operação (forma de operação, respeitando o teto de operação e o teto de ocupação do espaço físico - máx. pessoas)<br>Trabalhadores	Atendimento	Informativo visível (operação e ocupação)<br>Máscara / EPIs,<br>Distanciamento,<br>Teto de ocupação,<br>Higienização,<br>Proteção de grupo de risco,<br>Afastamento de casos,<br>Cuidados no atendimento ao público,<br>Atendimento do grupos de risco	Monitora- mento de tempera- tura	Testagem dos trabalha- dores	Normas obrigatórias específicas à atividade https://coronavirus.rs.gov.br/portarias-da-ses
Indústria de Transformação e Extrativa	23	Minerais não metálicos		75% trabalhadores	Teletrabalho no máximo possível /<br>Presencial restrito /<br>Ventilação cruzada (portas e janelas abertas) e/ou sistema de renovação de ar /<br><br>Restaurantes, bares, lanchonetes e espaços coletivos de alimentação: conforme protocolo de "Restaurantes" e "Lanchonetes" e Portaria SES nº 319 /	Uso obrigatório e correto de máscara, cobrindo boca e nariz por todos os presentes/<br><br>Distanciamento interpessoal mínimo de 1m /	X	X		Portaria SES nº 283 e nº 375
Indústria de Transformação e Extrativa	24	Metalurgia		75% trabalhadores	Teletrabalho no máximo possível /<br>Presencial restrito /<br>Ventilação cruzada (portas e janelas abertas) e/ou sistema de renovação de ar /<br><br>Restaurantes, bares, lanchonetes e espaços coletivos de alimentação: conforme protocolo de "Restaurantes" e "Lanchonetes" e Portaria SES nº 319 /	Uso obrigatório e correto de máscara, cobrindo boca e nariz por todos os presentes/<br><br>Distanciamento interpessoal mínimo de 1m /	X	X		Portaria SES nº 283 e nº 375
Indústria de Transformação e Extrativa	25	Produtos de Metal		75% trabalhadores	Teletrabalho no máximo possível /<br>Presencial restrito /<br>Ventilação cruzada (portas e janelas abertas) e/ou sistema de renovação de ar /<br><br>Restaurantes, bares, lanchonetes e espaços coletivos de alimentação: conforme protocolo de "Restaurantes" e "Lanchonetes" e Portaria SES nº 319 /	Uso obrigatório e correto de máscara, cobrindo boca e nariz por todos os presentes/<br><br>Distanciamento interpessoal mínimo de 1m /	X	X		Portaria SES nº 283 e nº 375

Notas:
(*) Representam agregações de atividades 2 dígitos:
100* = 6, 7, 8, 9

BANDEIRA PRETA - Indústria

// Atividade				// Critérios específicos de funcionamento (conforme bandeira)			// Protocolos obrigatório (todas as bandeiras)	// Protocolos variáveis (recomendados)		// Restrições adicionais
Grupo	CNAE (2 díg.)	Tipo	Subtipos	Teto de Operação (percentual máx. de trabalhadores presentes no turno, ao mesmo tempo, respeitando o teto de ocupação do espaço físico - máx. pessoas)	Modo de Operação (forma de operação, respeitando o teto de operação e o teto de ocupação do espaço físico - máx. pessoas)<br>Trabalhadores	Atendimento	Informativo visível (operação e ocupação) Máscara / EPIs, Distanciamento, Teto de ocupação, Higienização, Proteção de grupo de risco, Afastamento de casos, Cuidados no atendimento ao público, Atendimento do grupos de risco	Monitora- mento de tempera- tura	Testagem dos trabalha- dores	Normas obrigatórias específicas à atividade https://coronavirus.rs.gov.br/portarias-da-ses
Indústria de Transformação e Extrativa	26	Equip. Informática		75% trabalhadores	Teletrabalho no máximo possível / Presencial restrito / Ventilação cruzada (portas e janelas abertas) e/ou sistema de renovação de ar /<br><br>Restaurantes, bares, lanchonetes e espaços coletivos de alimentação: conforme protocolo de "Restaurantes" e "Lanchonetes" e Portaria SES nº 319 /	Uso obrigatório e correto de máscara, cobrindo boca e nariz por todos os presentes/<br><br>Distanciamento interpessoal mínimo de 1m /	X	X		Portaria SES nº 283 e nº 375
Indústria de Transformação e Extrativa	27	Materiais Elétricos		75% trabalhadores	Teletrabalho no máximo possível / Presencial restrito / Ventilação cruzada (portas e janelas abertas) e/ou sistema de renovação de ar /<br><br>Restaurantes, bares, lanchonetes e espaços coletivos de alimentação: conforme protocolo de "Restaurantes" e "Lanchonetes" e Portaria SES nº 319 /	Uso obrigatório e correto de máscara, cobrindo boca e nariz por todos os presentes/<br><br>Distanciamento interpessoal mínimo de 1m /	X	X		Portaria SES nº 283 e nº 375
Indústria de Transformação e Extrativa	28	Máquinas e Equipamentos		75% trabalhadores	Teletrabalho no máximo possível / Presencial restrito / Ventilação cruzada (portas e janelas abertas) e/ou sistema de renovação de ar /<br><br>Restaurantes, bares, lanchonetes e espaços coletivos de alimentação: conforme protocolo de "Restaurantes" e "Lanchonetes" e Portaria SES nº 319 /	Uso obrigatório e correto de máscara, cobrindo boca e nariz por todos os presentes/<br><br>Distanciamento interpessoal mínimo de 1m /	X	X		Portaria SES nº 283 e nº 375

DISTANCIAMENTO CONTROLADO
NOVAS BANDEIRAS

Notas:
(*) Representam agregações de atividades 2 dígitos:
100* = 6, 7, 8, 9

BANDEIRA PRETA - Indústria

// Atividade				// Critérios específicos de funcionamento (conforme bandeira)			// Protocolos obrigatório (todas as bandeiras)	// Protocolos variáveis (recomendados)		// Restrições adicionais
Grupo	CNAE (2 díg.)	Tipo	Subtipos	Teto de Operação (percentual máx. de trabalhadores presentes no turno, ao mesmo tempo, respeitando o teto de ocupação do espaço físico - máx. pessoas)	Modo de Operação (forma de operação, respeitando o teto de operação e o teto de ocupação do espaço físico - máx. pessoas)<br>Trabalhadores	Atendimento	Informativo visível (operação e ocupação)<br>Máscara / EPIs,<br>Distanciamento,<br>Teto de ocupação,<br>Higienização,<br>Proteção de grupo de risco,<br>Afastamento de casos,<br>Cuidados no atendimento ao público,<br>Atendimento do grupos de risco	Monitora- mento de tempera- tura	Testagem dos trabalha- dores	Normas obrigatórias específicas à atividade https://coronavirus.rs.gov.br/portarias-da-ses
Indústria de Transformação e Extrativa	29	Veículos Automotores		75% trabalhadores	Teletrabalho no máximo possível / Presencial restrito / Ventilação cruzada (portas e janelas abertas) e/ou sistema de renovação de ar /<br><br>Restaurantes, bares, lanchonetes e espaços coletivos de alimentação: conforme protocolo de "Restaurantes" e "Lanchonetes" e Portaria SES nº 319 /	Uso obrigatório e correto de máscara, cobrindo boca e nariz por todos os presentes/<br><br>Distanciamento interpessoal mínimo de 1m /	X	X		Portaria SES nº 283 e nº 375
Indústria de Transformação e Extrativa	30	Outros Equipamentos		75% trabalhadores	Teletrabalho no máximo possível / Presencial restrito / Ventilação cruzada (portas e janelas abertas) e/ou sistema de renovação de ar /<br><br>Restaurantes, bares, lanchonetes e espaços coletivos de alimentação: conforme protocolo de "Restaurantes" e "Lanchonetes" e Portaria SES nº 319 /	Uso obrigatório e correto de máscara, cobrindo boca e nariz por todos os presentes/<br><br>Distanciamento interpessoal mínimo de 1m /	X	X		Portaria SES nº 283 e nº 375
Indústria de Transformação e Extrativa	31	Móveis		75% trabalhadores	Teletrabalho no máximo possível / Presencial restrito / Ventilação cruzada (portas e janelas abertas) e/ou sistema de renovação de ar /<br><br>Restaurantes, bares, lanchonetes e espaços coletivos de alimentação: conforme protocolo de "Restaurantes" e "Lanchonetes" e Portaria SES nº 319 /	Uso obrigatório e correto de máscara, cobrindo boca e nariz por todos os presentes/<br><br>Distanciamento interpessoal mínimo de 1m /	X	X		Portaria SES nº 283 e nº 375

DISTANCIAMENTO CONTROLADO
.RS.GOV
NOVAS BANDEIRAS

Notas:
(*) Representam agregações de atividades 2 dígitos:
100* = 6, 7, 8, 9

BANDEIRA PRETA - Indústria

// Atividade				// Critérios específicos de funcionamento (conforme bandeira)			// Protocolos obrigatório (todas as bandeiras)	// Protocolos variáveis (recomendados)		// Restrições adicionais
Grupo	CNAE (2 díg.)	Tipo	Subtipos	Teto de Operação (percentual máx. de trabalhadores presentes no turno, ao mesmo tempo, respeitando o teto de ocupação do espaço físico - máx. pessoas)	Modo de Operação (forma de operação, respeitando o teto de operação e o teto de ocupação do espaço físico - máx. pessoas) Trabalhadores	Atendimento	Informativo visível (operação e ocupação) Máscara / EPIs, Distanciamento, Teto de ocupação, Higienização, Proteção de grupo de risco, Afastamento de casos, Cuidados no atendimento ao público, Atendimento do grupos de risco	Monitora- mento de tempera- tura	Testagem dos trabalha- dores	Normas obrigatórias específicas à atividade https://coronavirus.rs.gov.br/portarias-da-ses
Indústria de Transformação e Extrativa	32	Produtos Diversos		75% trabalhadores	Teletrabalho no máximo possível / Presencial restrito / Ventilação cruzada (portas e janelas abertas) e/ou sistema de renovação de ar / Restaurantes, bares, lanchonetes e espaços coletivos de alimentação: conforme protocolo de "Restaurantes" e "Lanchonetes" e Portaria SES nº 319 /	Uso obrigatório e correto de máscara, cobrindo boca e nariz por todos os presentes/ Distanciamento interpessoal mínimo de 1m /	X	X		Portaria SES nº 283 e nº 375
Indústria de Transformação e Extrativa	33	Manut. e Reparação		75% trabalhadores	Teletrabalho no máximo possível / Presencial restrito / Ventilação cruzada (portas e janelas abertas) e/ou sistema de renovação de ar / Restaurantes, bares, lanchonetes e espaços coletivos de alimentação: conforme protocolo de "Restaurantes" e "Lanchonetes" e Portaria SES nº 319 /	Uso obrigatório e correto de máscara, cobrindo boca e nariz por todos os presentes/ Distanciamento interpessoal mínimo de 1m /	X	X		Portaria SES nº 283 e nº 375
Indústria de Transformação e Extrativa	21	Farmoquímicos e Farmacêuticos		75% trabalhadores	Teletrabalho no máximo possível / Presencial restrito / Ventilação cruzada (portas e janelas abertas) e/ou sistema de renovação de ar / Restaurantes, bares, lanchonetes e espaços coletivos de alimentação: conforme protocolo de "Restaurantes" e "Lanchonetes" e Portaria SES nº 319 /	Uso obrigatório e correto de máscara, cobrindo boca e nariz por todos os presentes/ Distanciamento interpessoal mínimo de 1m /	X	X		Portaria SES nº 283 e nº 375

Notas:
(*) Representam agregações de atividades 2 dígitos:
100* = 6, 7, 8, 9

MODELO DE DISTANCIAMENTO
CONTROLADO DO RS

Saúde e Assistência

Comitê de DADOS
COVID-19 CORONAVÍRUS

GOV RS
NOVAS FAÇANHAS
NO PLANEJAMENTO, GOVERNANÇA E GESTÃO NA SAÚDE

BANDEIRA AMARELA - Saúde e Assistência

// Atividade				// Critérios específicos de funcionamento (conforme bandeira)			// Protocolos obrigatório (todas as bandeiras)	// Protocolos variáveis (recomendados)		// Restrições adicionais
Grupo	CNAE (2 díg.)	Tipo	Subtipos	Teto de Operação (percentual máx. de trabalhadores presentes no turno, ao mesmo tempo, respeitando o teto de ocupação do espaço físico - máx. pessoas)	Modo de Operação (forma de operação, respeitando o teto de operação e o teto de ocupação do espaço físico - máx. pessoas) Trabalhadores	Atendimento	Informativo visível (operação e ocupação) Máscara / EPIs, Distanciamento, Teto de ocupação, Higienização, Proteção de grupo de risco, Afastamento de casos, Cuidados no atendimento ao	Monitora- mento de tempera- tura	Testagem dos trabalha- dores	Normas obrigatórias específicas à atividade https://coronavirus.rs.gov.br/portarias-da-ses
Saúde e Assistência	86	Atenção à Saúde Humana		100% trabalhadores	Teletrabalho / Presencial restrito / Restaurantes, bares, lanchonetes e espaços coletivos de alimentação: conforme protocolo de "Restaurantes" e "Lanchonetes" e Portaria SES nº 319 /	Presencial restrito / Teleatendimento	X			Portaria SES nº 274, nº 284, nº 300 e nº 374
Saúde e Assistência	87	Assistência Social		100% trabalhadores	Teletrabalho / Presencial restrito / Restaurantes, bares, lanchonetes e espaços coletivos de alimentação: conforme protocolo de "Restaurantes" e "Lanchonetes" e Portaria SES nº 319 /	Presencial restrito / Teleatendimento	X			Portaria SES nº 289 e nº 352
Saúde e Assistência	75	Assistência Veterinária		75% trabalhadores	Teletrabalho / Presencial restrito	Presencial restrito / Teleatendimento	X			

DISTANCIAMENTO CONTROLADO
GOV RS NOVAS FAÇANHAS

BANDEIRA LARANJA - Saúde e Assistência

// Atividade				// Critérios específicos de funcionamento (conforme bandeira)			// Protocolos obrigatório (todas as bandeiras)	// Protocolos variáveis (recomendados)		// Restrições adicionais
Grupo	CNAE (2 díg.)	Tipo	Subtipos	Teto de Operação (percentual máx. de trabalhadores presentes no turno, ao mesmo tempo, respeitando o teto de ocupação do espaço físico - máx. pessoas)	Modo de Operação (forma de operação, respeitando o teto de operação e o teto de ocupação do espaço físico - máx. pessoas) Trabalhadores	Atendimento	Informativo visível (operação e ocupação) Máscara / EPIs, Distanciamento, Teto de ocupação, Higienização, Proteção de grupo de risco, Afastamento de casos, Cuidados no atendimento ao	Monitora- mento de tempera- tura	Testagem dos trabalha- dores	Normas obrigatórias específicas à atividade https://coronavirus.rs.gov.br/portarias-da-ses
Saúde e Assistência	86	Atenção à Saúde Humana		100% trabalhadores	Teletrabalho / Presencial restrito / Restaurantes, bares, lanchonetes e espaços coletivos de alimentação: conforme protocolo de "Restaurantes" e "Lanchonetes" e Portaria SES nº 319 /	Presencial restrito / Teleatendimento	X			Portaria SES nº 274, nº 284, nº 300 e nº 374
Saúde e Assistência	87	Assistência Social		100% trabalhadores	Teletrabalho / Presencial restrito / Restaurantes, bares, lanchonetes e espaços coletivos de alimentação: conforme protocolo de "Restaurantes" e "Lanchonetes" e Portaria SES nº 319 /	Presencial restrito / Teleatendimento	X			Portaria SES nº 289 e nº 352
Saúde e Assistência	75	Assistência Veterinária		75% trabalhadores	Teletrabalho / Presencial restrito	Presencial restrito / Teleatendimento	X			

DISTANCIAMENTO CONTROLADO
GOV RS NOVAS FAÇANHAS

BANDEIRA VERMELHA - Saúde e Assistência

// Atividade				// Critérios específicos de funcionamento (conforme bandeira)			// Protocolos obrigatório (todas as bandeiras)	// Protocolos variáveis (recomendados)		// Restrições adicionais
Grupo	CNAE (2 díg.)	Tipo	Subtipos	Teto de Operação (percentual máx. de trabalhadores presentes no turno, ao mesmo tempo, respeitando o teto de ocupação do espaço físico - máx. pessoas)	Modo de Operação (forma de operação, respeitando o teto de operação e o teto de ocupação do espaço físico - máx. pessoas) Trabalhadores	Atendimento	Informativo visível (operação e ocupação) Máscara / EPIs, Distanciamento, Teto de ocupação, Higienização, Proteção de grupo de risco, Afastamento de casos, Cuidados no atendimento ao público, Atendimento do grupos de risco	Monitora- mento de tempera- tura	Testagem dos trabalha- dores	Normas obrigatórias específicas à atividade https://coronavirus.rs.gov.br/portarias-da-ses
Saúde e Assistência	86	Atenção à Saúde Humana		100% trabalhadores	Teletrabalho / Presencial restrito / Restaurantes, bares, lanchonetes e espaços coletivos de alimentação: conforme protocolo de "Restaurantes" e "Lanchonetes" e Portaria SES nº 319 /	Presencial restrito / Teleatendimento	X	X		Portaria SES nº 274, nº 284, nº 300 e nº 374
Saúde e Assistência	87	Assistência Social		100% trabalhadores	Teletrabalho / Presencial restrito / Restaurantes, bares, lanchonetes e espaços coletivos de alimentação: conforme protocolo de "Restaurantes" e "Lanchonetes" e Portaria SES nº 319 /	Presencial restrito / Teleatendimento	X	X		Portaria SES nº 289 e nº 352
Saúde e Assistência	75	Assistência Veterinária		50% trabalhadores	Teletrabalho / Presencial restrito	Presencial restrito / Teleatendimento	X	X		

DISTANCIAMENTO CONTROLADO
GOV RS
NOVAS FAÇANHAS

BANDEIRA PRETA - Saúde e Assistência

// Atividade				// Critérios específicos de funcionamento (conforme bandeira)			// Protocolos obrigatório (todas as bandeiras)	// Protocolos variáveis (recomendados)		// Restrições adicionais
Grupo	CNAE (2 díg.)	Tipo	Subtipos	Teto de Operação (percentual máx. de trabalhadores presentes no turno, ao mesmo tempo, respeitando o teto de ocupação do espaço físico - máx. pessoas)	Modo de Operação (forma de operação, respeitando o teto de operação e o teto de ocupação do espaço físico - máx. pessoas) Trabalhadores	Atendimento	Informativo visível (operação e ocupação) Máscara / EPIs, Distanciamento, Teto de ocupação, Higienização, Proteção de grupo de risco, Afastamento de casos, Cuidados no atendimento ao público, Atendimento do grupos de risco	Monitora- mento de tempera- tura	Testagem dos trabalha- dores	Normas obrigatórias específicas à atividade https://coronavirus.rs.gov.br/portarias-da-ses
Saúde e Assistência	86	Atenção à Saúde Humana		100% trabalhadores	Teletrabalho / Presencial restrito / Restaurantes, bares, lanchonetes e espaços coletivos de alimentação: conforme protocolo de "Restaurantes" e "Lanchonetes" e Portaria SES nº 319 /	Presencial restrito / Teleatendimento	X	X		Portaria SES nº 274, nº 284, nº 300 e nº 374
Saúde e Assistência	87	Assistência Social		100% trabalhadores	Teletrabalho / Presencial restrito / Restaurantes, bares, lanchonetes e espaços coletivos de alimentação: conforme protocolo de "Restaurantes" e "Lanchonetes" e Portaria SES nº 319 /	Presencial restrito / Teleatendimento	X	X		Portaria SES nº 289 e nº 352
Saúde e Assistência	75	Assistência Veterinária		50% trabalhadores	Teletrabalho / Presencial restrito	Presencial restrito / Teleatendimento	X	X		

DISTANCIAMENTO CONTROLADO
.GOV RS
NOVAS FAÇANHAS

MODELO DE DISTANCIAMENTO
CONTROLADO DO RS

DISTANCIAMENTO CONTROLADO

Serviços

GOV RS NOVAS FAÇANHAS

BANDEIRA AMARELA - Serviços

<table>
<tr><th colspan="4">// Atividade</th><th colspan="3">// Critérios específicos de funcionamento
(conforme bandeira)</th><th>// Protocolos obrigatório
(todas as bandeiras)</th><th colspan="2">// Protocolos variáveis
(recomendados)</th><th>// Restrições adicionais</th></tr>
<tr><td>Grupo</td><td>CNAE
(2 díg.)</td><td>Tipo</td><td>Subtipos</td><td>Teto de Operação
Determina o percentual máximo de trabalhadores/público externo presentes no mesmo turno, ao mesmo tempo.

Deve respeitar ao nº máximo de pessoas no espaço físico, considerando o distanciamento interpessoal mínimo obrigatório (teto de ocupação).</td><td colspan="2">Modo de Operação
Forma de operação da atividade, respeitando ao teto de operação, ao teto de ocupação do espaço físico e aos protocolos obrigatórios (ao lado).

Trabalhadores | Atendimento</td><td>Decreto nº 55.2540:
- Máscara / EPIs,
- Distanciamento,
- Teto de ocupação,
- Higienização,
- Proteção de grupo de risco,
- Afastamento de casos,
- Cuidados com o público,
- Atendimento do grupos de risco
- Informativo visível (operação, ocupação e cuidados)</td><td>Monitora-mento de tempera- tura</td><td>Testagem dos trabalha- dores</td><td>Conteúdo completo das normas obrigatórias específicas à atividade: coronavirus.rs.gov.br/portarias-da-ses</td></tr>
<tr><td>Serviços</td><td>104*</td><td>Artes, Cultura, Esportes e Lazer</td><td>Parques Temáticos, Parques de Diversão, Parques de Aventura, Parques Aquáticos, Atrativos Turísticos e Similares - fixos ou itinerantes</td><td>50% trabalhadores
50% público</td><td>Teletrabalho /
Presencial restrito /
Restaurantes, bares, lanchonetes e espaços coletivos de alimentação: conforme protocolo de "Restaurantes" e "Lanchonetes" e Portaria SES nº 319 /</td><td>Teleatendimento /
Presencial restrito
(exclusivo locais com Selo Turismo Responsável do MTur)</td><td>X</td><td>X</td><td></td><td>Selo Turismo Responsável - Ministério do Turismo</td></tr>
<tr><td>Serviços</td><td>104*</td><td>Artes, Cultura, Esportes e Lazer</td><td>Parques e reservas naturais, jardins botânicos e zoológicos</td><td>50% trabalhadores
50% público</td><td>Teletrabalho /
Presencial restrito /
Restaurantes, bares, lanchonetes e espaços coletivos de alimentação: conforme protocolo de "Restaurantes" e "Lanchonetes" e Portaria SES nº 319 /</td><td>Teleatendimento /
Presencial restrito
(somentes áreas externas, com demarcação no chão de áreas de permanência distanciada de grupos - máx. 8 pessoas)</td><td>X</td><td>X</td><td></td><td>Selo Turismo Responsável - Ministério do Turismo</td></tr>
<tr><td>Serviços</td><td>104*</td><td>Artes, Cultura, Esportes e Lazer</td><td>Teatros, auditórios, casas de espetáculos, casas de show, circos e similares

(em ambiente aberto ou fechado, com público exclusivamente sentado e restrito ao período da apresentação)</td><td>Ambientes FECHADOS E ABERTOS: permitidos.

Respeitando à lotação, ao distanciamento e à necessidade de autorização, conforme número total de pessoas (ver "Restrições Adicionais").

Local permite consumo de alimentos/ bebidas:

- PERMITE - 40% de lotação, com distanciamento de 2m

- NÃO PERMITE - 50% de lotação, com distanciamento de 1m</td><td>Teletrabalho /
Presencial restrito /
Para ambiente aberto, permitido instalação de toldo ou cobertura, desde que as laterais sejam inteiramente abertas, para plena circulação de ar /
Elaboração de projeto (croqui e protocolos), disponível para fiscalização e/ou autorização, quando exigido /
Circulação de ar cruzada ou sistema de renovação de ar /
Início e término de programações não concomitantes, quando houver multissalas /
Intervalo mín. de 1 hora entre as apresentações com troca de público, para permitir higienização e evitar aglomerações /
Restaurantes, bares, lanchonetes e espaços coletivos de alimentação: conforme protocolo de "Restaurantes" e "Lanchonetes" e Portaria SES nº 319 /</td><td>Presencial restrito /
Máscara de uso obrigatório /
Reforço na comunicação sonora e visual dos protocolos de higiene e distanciamento para público e colaboradores /
Circulação em pé somente para uso dos sanitários, com uso de máscara e fila com distanciamento demarcado /
Vedado interação física entre artistas e público /

PERMITE ALIMENTAÇÃO/ BEBIDA: distanciamento mínimo de 2m entre pessoas e/ou grupos de coabitantes OU ocupação intercalada de assentos (sim/não/não/sim) e ocupação intercalada das fileiras /

NÃO PERMITE ALIMENTAÇÃO/ BEBIDA: distanciamento mínimo de 1m entre pessoas e/ou grupos de coabitantes OU ocupação intercalada de assentos (sim/não/não/sim), sem ocupação de assento(s) imediatamente à frente e atrás /</td><td>X</td><td>X</td><td></td><td>Portaria SES nº 617

Portaria SES nº 319

Decreto Estadual nº 55.240, Art. 21, § 7º e §8º

Pedido de autorização, conforme número de pessoas (trabalhadores e público) presentes ao mesmo tempo:
- Até 300: protocolos estaduais;
- 300 a 600: protocolos estaduais (+) pedido de autorização do município sede, encaminhado pela organização do evento;
- 600 a 1.200: protocolos estaduais (+) pedido de autorização da(s) associação(ões) de municípios da Região Covid, encaminhado pelo município sede (aprovação por no mín. 2/3 dos municípios da Região)
- 1.200 a 2.500, no máx.: protocolos estaduais (+) pedido de autorização do Gabinete de Crise, encaminhado pela(s) associação(ões) de municípios da Região Covid, após aprovação dessa(s)</td></tr>
<tr><td>Serviços</td><td>104*</td><td>Artes, Cultura, Esportes e Lazer</td><td>Espetáculos tipo drive-in (cinema, shows, etc.)</td><td>100% vagas,

com distanciamento</td><td>Teletrabalho /
Presencial restrito /
Sem contato físico /
Alimentos e bebidas solicitados por aplicativo e entregues no carro</td><td>Teleatendimento /
Presencial restrito /
Público somente nos automóveis /
Vedada abertura de portas e circulação externa aos automóveis /
Circulação somente para uso dos sanitários, com uso de máscara e fila com distanciamento demarcado</td><td>X</td><td></td><td></td><td></td></tr>
</table>

Notas:
(*) Representam agregações de atividades 2 dígitos:
101* = 64, 65, 66 | 104* = 90, 91, 92, 93
102* = 69, 70, 71, 72, 73, 74, 75 | 105* = 94, 95, 96, 99
103* = 77, 78, 79, 82

DISTANCIAMENTO CONTROLADO
NOVAS FAÇANHAS

BANDEIRA AMARELA - Serviços

// Atividade				// Critérios específicos de funcionamento (conforme bandeira)			// Protocolos obrigatório (todas as bandeiras)	// Protocolos variáveis (recomendados)		// Restrições adicionais
Grupo	CNAE (2 díg.)	Tipo	Subtipos	**Teto de Operação**<br>Determina o percentual máximo de trabalhadores/público externo presentes no mesmo turno, ao mesmo tempo.<br><br>Deve respeitar ao nº máximo de pessoas no espaço físico, considerando o distanciamento interpessoal mínimo obrigatório (teto de ocupação).	**Modo de Operação**<br>Forma de operação da atividade, respeitando ao teto de operação, ao teto de ocupação do espaço físico e aos protocolos obrigatórios (ao lado).<br><br>**Trabalhadores**	**Atendimento**	**Decreto nº 55.2540:**<br>- Máscara / EPIs,<br>- Distanciamento,<br>- Teto de ocupação,<br>- Higienização,<br>- Proteção de grupo de risco,<br>- Afastamento de casos,<br>- Cuidados com o público,<br>- Atendimento do grupos de risco<br>- Informativo visível (operação, ocupação e cuidados)	**Monitora-mento de tempera- tura**	**Testagem dos trabalha- dores**	Conteúdo completo das normas obrigatórias específicas à atividade: coronavirus.rs.gov.br/portarias-da-ses
Serviços	104*	Artes, Cultura, Esportes e Lazer	Cinemas	**Permitido,** respeitando à **lotação** e ao **distanciamento:**<br><br>Estabelecimento permite CONSUMO DE ALIMENTOS OU BEBIDAS:<br><br>- PERMITE - **40% de lotação**, com distanciamento de 2m<br><br>- NÃO PERMITE - **50% de lotação**, com distanciamento de 1m	Teletrabalho /<br>Presencial restrito /<br>Elaboração de projeto (croqui e protocolos), disponível para fiscalização e/ou autorização, quando exigido /<br>Circulação de ar cruzada ou sistema de renovação de ar /<br>Início e término de programações não concomitantes, quando houver multissalas /<br>Intervalo mín. de 1 hora entre as apresentações com troca de público, para permitir higienização e evitar aglomerações /<br>Restaurantes, bares, lanchonetes e espaços coletivos de alimentação: conforme protocolo de "Restaurantes" e "Lanchonetes" e Portaria SES nº 319 /	Presencial restrito /<br>Máscara de uso obrigatório /<br>Reforço na comunicação sonora e visual dos protocolos de higiene e distanciamento para público e colaboradores /<br>Circulação em pé somente para uso dos sanitários, com uso de máscara e fila com distanciamento demarcado /<br><br>PERMITE ALIMENTAÇÃO/BEBIDA: distanciamento mínimo de 2m entre pessoas e/ou grupos de coabitantes OU ocupação intercalada de assentos (sim/não/não/sim) e ocupação intercalada das fileiras /<br><br>NÃO PERMITE ALIMENTAÇÃO/BEBIDA: distanciamento mínimo de 1m entre pessoas e/ou grupos de coabitantes OU ocupação intercalada de assentos (sim/não/não/sim), sem ocupação de assento(s) imediatamente à frente e atrás /	X	X		Portaria SES nº 319<br><br>Decreto Estadual nº 55.240, Art. 21, §8º
Serviços	104*	Artes, Cultura, Esportes e Lazer	Museus, centros culturais e similares	50% trabalhadores<br><br>50% público	Teletrabalho /<br>Presencial restrito /<br>Restaurantes, bares, lanchonetes e espaços coletivos de alimentação: conforme protocolo de "Restaurantes" e "Lanchonetes" e Portaria SES nº 319 /	Teleatendimento /<br>Presencial restrito	X	X		Recomendações aos Museus em Tempos de Covid-19, do Instituto Brasileiro de Museus (Ibram)
Serviços	104*	Artes, Cultura, Esportes e Lazer	Bibliotecas, arquivos, acervos e similares	50% trabalhadores<br><br>50% público	Teletrabalho/<br>Presencial restrito	Teleatendimento /<br>Presencial restrito	X			
Serviços	104*	Artes, Cultura, Esportes e Lazer	Ateliês (artes plásticas, restauração de obras de arte, escrita, artistas independentes e similares)	50% trabalhadores	Teletrabalho/<br>Presencial restrito	Teleatendimento /<br>Atendimento individualizado, com agendamento	X			
Serviços	104*	Artes, Cultura, Esportes e Lazer	Atividades de organizações associativas ligadas à arte e à cultura (MTG e similares)	50% trabalhadores	Teletrabalho/<br>Presencial restrito	Teleatendimento /<br>Atendimento individualizado, com agendamento	X			
Serviços	104*	Artes, Cultura, Esportes e Lazer	Convenções partidárias	50%<br>lotação<br><br>Máx. 100 pessoas, ao mesmo tempo	Teletrabalho /<br>Presencial restrito /<br>Circulação de ar cruzada /<br>Credenciamento e check-in online	Presencial restrito /<br>Cadeiras intercalados (sim/não/não/sim) /<br>Filas intercaladas /<br>6m² por pessoa /<br>Entrada e saída escalonada por filas previamente demarcadas /<br>Material individual (canetas)	X	X		

DISTANCIAMENTO CONTROLADO
.GOV RS
NOVAS FAÇANHAS

Notas:
(*) Representam agregações de atividades 2 dígitos:
101* = 64, 65, 66
102* = 69, 70, 71, 72, 73, 74, 75
103* = 77, 78, 79, 82
104* = 90, 91, 92, 93
105* = 94, 95, 96, 99

BANDEIRA AMARELA - Serviços

// Atividade				// Critérios específicos de funcionamento (conforme bandeira)			// Protocolos obrigatório (todas as bandeiras)	// Protocolos variáveis (recomendados)		// Restrições adicionais
Grupo	CNAE (2 díg.)	Tipo	Subtipos	**Teto de Operação**<br>Determina o percentual máximo de trabalhadores/público externo presentes no mesmo turno, ao mesmo tempo.<br><br>Deve respeitar ao nº máximo de pessoas no espaço físico, considerando o distanciamento interpessoal mínimo obrigatório (teto de ocupação).	**Modo de Operação**<br>Forma de operação da atividade, respeitando ao teto de operação, ao teto de ocupação do espaço físico e aos protocolos obrigatórios (ao lado).<br><br>**Trabalhadores**	**Atendimento**	**Decreto nº 55.2540:**<br>- Máscara / EPIs,<br>- Distanciamento,<br>- Teto de ocupação,<br>- Higienização,<br>- Proteção de grupo de risco,<br>- Afastamento de casos,<br>- Cuidados com o público,<br>- Atendimento do grupos de risco<br>- Informativo visível (operação, ocupação e cuidados)	**Monitora- mento de tempera- tura**	**Testagem dos trabalha- dores**	Conteúdo completo das normas obrigatórias específicas à atividade: coronavirus.rs.gov.br/portarias-da-ses
Serviços	104*	Artes, Cultura, Esportes e Lazer	Feiras e Exposições corporativas e comerciais	**Permitido**, respeitando à **lotação**, ao **distanciamento** e à necessidade de **autorização**, conforme número total de pessoas (ver "Restrições Adicionais").	Teletrabalho /<br>Presencial restrito /<br>Elaboração de projeto (croqui e protocolos), disponível para fiscalização e/ou autorização, quando couber /<br>Módulos de estandes distanciados 4 metros um do outro /<br>Circulação de ar cruzada /<br>Credenciamento e check-in online /<br>Início e término de programações não concomitantes, quando houver multissalas /<br>Restaurantes, bares, lanchonetes e espaços coletivos de alimentação: conforme protocolo de "Restaurantes" e "Lanchonetes" e Portaria SES nº 319 /	Teleatendimento /<br>Presencial restrito /<br>Ambientes (estandes, salas, corredores, etc.) com circulação em pé: contabilizar mínimo de 8m² por pessoa /<br>Ambientes com público sentado: contabilizar mínimo de 4m² por pessoa, considerando se o local permite alimentação ou bebida:<br><br>PERMITE ALIMENTAÇÃO/BEBIDA: distanciamento mínimo de 2m entre pessoas e/ou grupos de coabitantes OU ocupação intercalada de assentos (sim/não/não/sim) e ocupação intercalada das fileiras /<br><br>NÃO PERMITE ALIMENTAÇÃO/BEBIDA: distanciamento mínimo de 1m entre pessoas e/ou grupos de coabitantes OU ocupação intercalada de assentos (sim/não/não/sim), sem ocupação de assento(s) imediatamente à frente e atrás /	X	X		Portaria SES nº 617<br><br>Portaria SES nº 319<br><br>Decreto Estadual nº 55.240, Art. 21, § 7º e §8º<br><br>Pedido de autorização, conforme número de pessoas (trabalhadores e público) presentes ao mesmo tempo:<br>- **Até 300**: protocolos estaduais;<br>- **300 a 600**: protocolos estaduais (+) pedido de autorização do município sede, encaminhado pela organização do evento;<br>- **600 a 1.200**: protocolos estaduais (+) pedido de autorização da(s) associação(ões) de municípios da Região Covid, encaminhado pelo município sede (aprovação por no mín. 2/3 dos municípios da Região)<br>- **1.200 a 2.500**, no máx.: protocolos estaduais (+) pedido de autorização do Gabinete de Crise, encaminhado pela(s) associação(ões) de municípios da Região Covid, após aprovação dessa(s)

DISTANCIAMENTO CONTROLADO
GOV RS
NOVAS FAÇANHAS

Notas:
(*) Representam agregações de atividades 2 dígitos:
101* = 64, 65, 66
102* = 69, 70, 71, 72, 73, 74, 75
103* = 77, 78, 79, 82
104* = 90, 91, 92, 93
105* = 94, 95, 96, 99

BANDEIRA AMARELA - Serviços

// Atividade				// Critérios específicos de funcionamento (conforme bandeira)			// Protocolos obrigatório (todas as bandeiras)	// Protocolos variáveis (recomendados)		// Restrições adicionais
Grupo	CNAE (2 díg.)	Tipo	Subtipos	**Teto de Operação**<br>Determina o percentual máximo de trabalhadores/público externo presentes no mesmo turno, ao mesmo tempo.<br><br>Deve respeitar ao nº máximo de pessoas no espaço físico, considerando o distanciamento interpessoal mínimo obrigatório (teto de ocupação).	**Modo de Operação**<br>Forma de operação da atividade, respeitando ao teto de operação, ao teto de ocupação do espaço físico e aos protocolos obrigatórios (ao lado).<br><br>**Trabalhadores**	**Atendimento**	**Decreto nº 55.2540:**<br>- Máscara / EPIs,<br>- Distanciamento,<br>- Teto de ocupação,<br>- Higienização,<br>- Proteção de grupo de risco,<br>- Afastamento de casos,<br>- Cuidados com o público,<br>- Atendimento do grupos de risco<br>- Informativo visível (operação, ocupação e cuidados)	**Monitora-mento de tempera- tura**	**Testagem dos trabalha- dores**	Conteúdo completo das normas obrigatórias específicas à atividade: coronavirus.rs.gov.br/portarias-da-ses
Serviços	104*	Artes, Cultura, Esportes e Lazer	Seminários, congressos, convenções, simpósios e similares	**Permitido**, respeitando à **lotação**, ao **distanciamento** e à necessidade de **autorização**, conforme número total de pessoas (ver "Restrições Adicionais").	Teletrabalho /<br>Presencial restrito /<br>Elaboração de projeto (croqui e protocolos), disponível para fiscalização e/ou autorização, quando couber /<br>Módulos de estandes distanciados 4 metros um do outro /<br>Circulação de ar cruzada /<br>Credenciamento e check-in online /<br>Início e término de programações não concomitantes, quando houver multissalas /<br>Restaurantes, bares, lanchonetes e espaços coletivos de alimentação: conforme protocolo de "Restaurantes" e "Lanchonetes" e Portaria SES nº 319 /	Teleatendimento /<br>Presencial restrito /<br>Ambientes (estandes, salas, corredores, etc.) com circulação em pé: contabilizar mínimo de 8m² por pessoa /<br>Ambientes com público sentado: contabilizar mínimo de 4m² por pessoa, considerando se o local permite alimentação ou bebida:<br><br>PERMITE ALIMENTAÇÃO/BEBIDA: distanciamento mínimo de 2m entre pessoas e/ou grupos de coabitantes OU ocupação intercalada de assentos (sim/não/não/sim) e ocupação intercalada das fileiras /<br><br>NÃO PERMITE ALIMENTAÇÃO/BEBIDA: distanciamento mínimo de 1m entre pessoas e/ou grupos de coabitantes OU ocupação intercalada de assentos (sim/não/não/sim), sem ocupação de assento(s) imediatamente à frente e atrás /	X	X		Portaria SES nº 617<br><br>Portaria SES nº 319<br><br>Decreto Estadual nº 55.240, Art. 21, § 7º e §8º<br><br>Pedido de autorização, conforme número de pessoas (trabalhadores e público) presentes ao mesmo tempo:<br>- **Até 300**: protocolos estaduais;<br>- **300 a 600**: protocolos estaduais (+) pedido de autorização do município sede, encaminhado pela organização do evento;<br>- **600 a 1.200**: protocolos estaduais (+) pedido de autorização da(s) associação(ões) de municípios da Região Covid, encaminhado pelo município sede (aprovação por no mín. 2/3 dos municípios da Região)<br>- **1.200 a 2.500**, no máx.: protocolos estaduais (+) pedido de autorização do Gabinete de Crise, encaminhado pela(s) associação(ões) de municípios da Região Covid, após aprovação dessa(s)
Serviços	104*	Artes, Cultura, Esportes e Lazer	Reuniões corporativas, oficinas, treinamentos e cursos corporativos	**Máximo de 100 pessoas** (trabalhadores e públicos), respeitando ao **teto de ocupação** e ao **distanciamento** estabelecido no Modo de Atendimento	Teletrabalho /<br>Presencial restrito /<br>Elaboração de projeto (croqui e protocolos), disponível para fiscalização e/ou autorização, quando couber /<br>Módulos de estandes distanciados 4 metros um do outro /<br>Circulação de ar cruzada /<br>Credenciamento e check-in online /<br>Início e término de programações não concomitantes, quando houver multissalas /<br>Restaurantes, bares, lanchonetes e espaços coletivos de alimentação: conforme protocolo de "Restaurantes" e "Lanchonetes" e Portaria SES nº 319 /	Teleatendimento /<br>Presencial restrito /<br>Material individual /<br>Ambientes (estandes, salas, corredores, etc.) com circulação em pé: contabilizar mínimo de 8m² por pessoa /<br>Ambientes com público sentado: contabilizar mínimo de 4m² por pessoa, considerando se o local permite alimentação ou bebida:<br><br>PERMITE ALIMENTAÇÃO/BEBIDA: distanciamento mínimo de 2m entre pessoas e/ou grupos de coabitantes OU ocupação intercalada de assentos (sim/não/não/sim) e ocupação intercalada das fileiras /<br><br>NÃO PERMITE ALIMENTAÇÃO/BEBIDA: distanciamento mínimo de 1m entre pessoas e/ou grupos de coabitantes OU ocupação intercalada de assentos (sim/não/não/sim), sem ocupação de assento(s) imediatamente à frente e atrás /	X	X		Portaria SES nº 617<br><br>Portaria SES nº 319

DISTANCIAMENTO CONTROLADO

Notas:
(*) Representam agregações de atividades 2 dígitos:
101* = 64, 65, 66
102* = 69, 70, 71, 72, 73, 74, 75
103* = 77, 78, 79, 82
104* = 90, 91, 92, 93
105* = 94, 95, 96, 99

BANDEIRA AMARELA - Serviços

// Atividade				// Critérios específicos de funcionamento (conforme bandeira)			// Protocolos obrigatório (todas as bandeiras)	// Protocolos variáveis (recomendados)		// Restrições adicionais
Grupo	CNAE (2 díg.)	Tipo	Subtipos	**Teto de Operação**<br>Determina o percentual máximo de trabalhadores/público externo presentes no mesmo turno, ao mesmo tempo.<br><br>Deve respeitar ao nº máximo de pessoas no espaço físico, considerando o distanciamento interpessoal mínimo obrigatório (teto de ocupação).	**Modo de Operação**<br>Forma de operação da atividade, respeitando ao teto de operação, ao teto de ocupação do espaço físico e aos protocolos obrigatórios (ao lado).<br><br>**Trabalhadores**	**Atendimento**	**Decreto nº 55.2540:**<br>- Máscara / EPIs,<br>- Distanciamento,<br>- Teto de ocupação,<br>- Higienização,<br>- Proteção de grupo de risco,<br>- Afastamento de casos,<br>- Cuidados com o público,<br>- Atendimento do grupos de risco<br>- Informativo visível (operação, ocupação e cuidados)	**Monitora- mento de tempera- tura**	**Testagem dos trabalha- dores**	Conteúdo completo das normas obrigatórias específicas à atividade: coronavirus.rs.gov.br/portarias-da-ses
Serviços	104*	Artes, Cultura, Esportes e Lazer	Eventos infantis em buffets, casas de festas ou similares<br><br>(em ambiente aberto ou fechado)	**Máximo de 100 pessoas** (trabalhadores e públicos), respeitando **teto de ocupação** e **distanciamento** estabelecido no Modo de Operação	Teletrabalho /<br>Presencial restrito /<br>Teto de ocupação: mínimo de 8m² por pessoa, respeitando a lotação máxima da bandeira /<br>Elaboração de projeto (croqui e protocolos), disponível para fiscalização e/ou autorização, quando couber /<br>Distanciamento mínimo de 2m entre mesas /<br>Ventilação forçada ou circulação de ar cruzada, com manutenção de janelas e portas abertas, independente do uso de equipamento de climatização /<br>Adesivagem do piso demarcando distanciamento mín. 1m nas filas /<br>Fluxo único de entrada, saída e circulação /<br>Abertura antecipada e ingresso escalonado ao evento /<br>Reforço constante na comunicação visual e sonora dos protocolos de higiene e distanciamento /<br>Distanciamento mín. 2m entre artistas e público, vedado o contato físico /<br>Tapetes sanitizantes em todas as entradas /<br>Higienização de camarins, camarotes e todas as áreas comuns (corredores, portas, elevadores, banheiros, vestiários, mesas, assentos e superfícies de contato) antes da abertura do evento e após seu término /<br>Higienização a cada 1 hora de superfícies de contato (mesas, corrimão, balcões etc) /<br>Higienização a cada 2 horas de banheiro e áreas comuns de maior circulação /<br>Higienização dos brinquedos a cada uso, com álcool 70% e/ou solução sanitizante similar /<br>Intervalo mín. de 1 hora entre as apresentações com troca de público, para permitir higienização e evitar aglomerações /<br>Início e término não concomitantes de programações com troca de público /<br>Reforço nos EPIs de colaboradores (máscara e faceshield) e higienização constante das mãos /<br>Organização e escalonamento da equipe de trabalhadores em grupos únicos (bolhas) /<br>Instrumentos musicais de uso individual, vedado o compartilhamento /<br>Vedados alimentos e bebidas expostos (mesa de doces, salgados e bebidas) /<br>Serviços de alimentação e bebidas conforme Portaria SES nº 319 conforme protocolo de "Restaurantes" e "Lanchonetes" e Portaria SES nº 319 /	Duração máxima do evento (para o público): 4 horas<br>Máscara de uso obrigatório sempre, à exceção do momento do consumo de alimentos ou bebidas, repondo imediatamente depois /<br>Priorização da venda e conferência de ingressos ou convites por meio visual ou digital, sem contato /<br>Registro dos contatos de todos os presentes (trabalhadores e público) e documento jurídico autorizativo de contato para rastreabilidade em caso de posterior confirmação ou suspeita de Covid-19 /<br>Disponibilização de totens e dispensers de álcool em gel com acionamento automático, sem contato, e em diferentes locais estratégicos /<br>Disponibilizar álcool em gel e monitor para orientar sobre o uso da máscara e a correta higienização das mãos antes e depois de acessar os brinquedos /<br>Kit completo nos banheiros (álcool gel 70%, sabonete líquido, toalhas de papel e lixeira com tampa de acionamento sem uso das mãos) /<br>Vedado consumo de alimentos e de bebidas em pé /<br>Vedado uso de bebedouros verticais /<br>Vedado uso de pista de dança /<br>Suspensão de todas as atividades em caso de detecção de surto /	X	X		Portaria SES nº 617<br><br>Portaria SES nº 319<br><br>Decreto Estadual nº 55.240, Art. 21, §8º

DISTANCIAMENTO CONTROLADO
GOV RS
NOVAS FAÇANHAS

Notas:
(*) Representam agregações de atividades 2 dígitos:
101* = 64, 65, 66
102* = 69, 70, 71, 72, 73, 74, 75
103* = 77, 78, 79, 82
104* = 90, 91, 92, 93
105* = 94, 95, 96, 99

BANDEIRA AMARELA - Serviços

// Atividade				// Critérios específicos de funcionamento (conforme bandeira)			// Protocolos obrigatório (todas as bandeiras)	// Protocolos variáveis (recomendados)		// Restrições adicionais
Grupo	CNAE (2 díg.)	Tipo	Subtipos	**Teto de Operação**<br>Determina o percentual máximo de trabalhadores/público externo presentes no mesmo turno, ao mesmo tempo.<br><br>Deve respeitar ao nº máximo de pessoas no espaço físico, considerando o distanciamento interpessoal mínimo obrigatório (teto de ocupação).	**Modo de Operação**<br>Forma de operação da atividade, respeitando ao teto de operação, ao teto de ocupação do espaço físico e aos protocolos obrigatórios (ao lado).<br><br>**Trabalhadores**	**Atendimento**	**Decreto nº 55.2540:**<br>- Máscara / EPIs,<br>- Distanciamento,<br>- Teto de ocupação,<br>- Higienização,<br>- Proteção de grupo de risco,<br>- Afastamento de casos,<br>- Cuidados com o público,<br>- Atendimento do grupos de risco<br>- Informativo visível (operação, ocupação e cuidados)	**Monitora- mento de tempera- tura**	**Testagem dos trabalha- dores**	Conteúdo completo das normas obrigatórias específicas à atividade: coronavirus.rs.gov.br/portarias-da-ses
Serviços	104*	Artes, Cultura, Esportes e Lazer	Eventos sociais e de entretenimento em buffets, casas de festas, casas de shows, casas noturnas, bares e pubs ou similares<br><br>(em ambiente fechado, com público em pé)	**Máximo de 100 pessoas** (trabalhadores e públicos), respeitando **teto de ocupação** e **distanciamento** estabelecido no Modo de Operação	Teletrabalho /<br>Presencial restrito /<br>Teto de ocupação: mínimo de 8m² por pessoa, respeitando a lotação máxima da bandeira /<br>Elaboração de projeto (croqui e protocolos), disponível para fiscalização e/ou autorização, quando couber /<br>Distanciamento mínimo de 2m entre mesas /<br>Áreas exclusivas para até 8 coabitantes, com distanciamento mín. 4m e demarcadas no chão (não permite bebida/alimentação) ou por barreira física (permite bebida/alimentação) /<br>Ventilação forçada ou circulação de ar cruzada, com manutenção de janelas e portas abertas, independente do uso de equipamento de climatização /<br>Adesivagem do piso demarcando distanciamento mín. 1m nas filas /<br>Fluxo único de entrada, saída e circulação /<br>Abertura antecipada e ingresso escalonado ao evento /<br>Reforço constante na comunicação visual e sonora dos protocolos de higiene e distanciamento /<br>Distanciamento mín. 2m entre artistas e público, vedado o contato físico /<br>Tapetes sanitizantes em todas as entradas /<br>Higienização de camarins, camarotes e todas as áreas comuns (corredores, portas, elevadores, banheiros, vestiários, grades, mesas, assentos e superfícies de contato) antes da abertura do evento e após seu término /<br>Higienização a cada 1 hora de superfícies de contato (mesas, maçanetas, corrimão, balcões etc)<br>Higienização a cada 2 horas de banheiro e áreas comuns de maior circulação /<br>Intervalo mín. de 1 hora entre as apresentações com troca de público, para permitir higienização e evitar aglomerações /<br>Início e término não concomitantes de programações com troca de público /<br>Reforço nos EPIs de colaboradores (máscara e faceshield) e higienização constante das mãos /<br>Organização e escalonamento da equipe de trabalhadores em grupos únicos (bolhas) /<br>Instrumentos musicais de uso individual, vedado o compartilhamento /<br>Vedados alimentos e bebidas expostos (mesa de doces, salgados e bebidas) /<br>Serviços de alimentação e bebidas conforme Portaria SES nº 319 conforme protocolo de "Restaurantes" e "Lanchonetes" e Portaria SES nº 319 /	Duração máxima do evento (para o público): **4 horas /**<br>Máscara de uso obrigatório sempre, à exceção do momento do consumo de alimentos ou bebidas, repondo imediatamente depois /<br>Priorização da venda e conferência de ingressos ou convites por meio visual ou digital, sem contato /<br>Registro dos contatos de todos os presentes (trabalhadores e público) e documento jurídico autorizativo de contato para rastreabilidade em caso de posterior confirmação ou suspeita de Covid-19 /<br>Priorização de pagamentos sem contato (contactless) e/ou higienização a cada uso das máquinas de pagamento de cartão com álcool 70% /<br>Disponibilização de totens e dispensers de álcool em gel com acionamento automático, sem contato, e em diferentes locais estratégicos /<br>Kit completo nos banheiros (álcool gel 70%, sabonete líquido, toalhas de papel e lixeira com tampa de acionamento sem uso das mãos) /<br>Vedado consumo de alimentos e de bebidas em pé /<br>Vedado uso de pista de dança /<br>Suspensão de todas as atividades em caso de detecção de surto /	X	X		Portaria SES nº 617<br><br>Portaria SES nº 319<br><br>Decreto Estadual nº 55.240, Art. 21, §8º

Notas:
(*) Representam agregações de atividades 2 dígitos:

101* = 64, 65, 66
102* = 69, 70, 71, 72, 73, 74, 75
103* = 77, 78, 79, 82
104* = 90, 91, 92, 93
105* = 94, 95, 96, 99

BANDEIRA AMARELA - Serviços

// Atividade				// Critérios específicos de funcionamento (conforme bandeira)			// Protocolos obrigatório (todas as bandeiras)	// Protocolos variáveis (recomendados)		// Restrições adicionais
Grupo	CNAE (2 díg.)	Tipo	Subtipos	**Teto de Operação**<br>Determina o percentual máximo de trabalhadores/público externo presentes no mesmo turno, ao mesmo tempo.<br><br>Deve respeitar ao nº máximo de pessoas no espaço físico, considerando o distanciamento interpessoal mínimo obrigatório (teto de ocupação).	**Modo de Operação**<br>Forma de operação da atividade, respeitando ao teto de operação, ao teto de ocupação do espaço físico e aos protocolos obrigatórios (ao lado).<br><br>**Trabalhadores**	**Atendimento**	**Decreto nº 55.2540:**<br>- Máscara / EPIs,<br>- Distanciamento,<br>- Teto de ocupação,<br>- Higienização,<br>- Proteção de grupo de risco,<br>- Afastamento de casos,<br>- Cuidados com o público,<br>- Atendimento do grupos de risco<br>- Informativo visível (operação, ocupação e cuidados)	**Monitora-mento de tempera- tura**	**Testagem dos trabalha- dores**	Conteúdo completo das normas obrigatórias específicas à atividade: coronavirus.rs.gov.br/portarias-da-ses
Serviços	104*	Artes, Cultura, Esportes e Lazer	Eventos sociais e de entretenimento em ambiente aberto, com público em pé	**Permitido**, respeitando à **lotação**, ao **distanciamento** e à necessidade de **autorização, conforme número total de pessoas (ver "Restrições Adicionais").**<br><br>Local permite CONSUMO DE ALIMENTOS OU BEBIDAS:<br><br>- PERMITE - **40% de lotação** do PPCI, respeitando à lotação, ao distanciamento e à necessidade de autorização, conforme número total de pessoas (ver "Restrições Adicionais").<br><br>- NÃO PERMITE - **50% de lotação** do PPCI, respeitando à lotação, ao distanciamento e à necessidade de autorização, conforme número total de pessoas (ver "Restrições Adicionais").	Teletrabalho /<br>Presencial restrito /<br>Teto de ocupação: mínimo de 8m² por pessoa, respeitando a lotação máxima da bandeira /<br>Elaboração de projeto (croqui e protocolos), disponível para fiscalização e/ou autorização, quando couber /<br>Controle de acesso à área do evento /<br>Distanciamento mínimo de 2m entre mesas /<br>Áreas exclusivas para até 8 coabitantes, com distanciamento mín. 4m e demarcadas no chão (não permite bebida/alimentação) ou por barreira física (permite bebida/alimentação) /<br>Adesivagem do piso demarcando distanciamento mín. 1m nas filas /<br>Fluxo único de entrada, saída e circulação /<br>Abertura antecipada e ingresso escalonado ao evento /<br>Reforço constante na comunicação visual e sonora dos protocolos de higiene e distanciamento /<br>Distanciamento mín. 2m entre artistas e público, vedado o contato físico /<br>Tapetes sanitizantes em todas as entradas /<br>Higienização de camarins, camarotes e todas as áreas comuns (corredores, portas, elevadores, banheiros, vestiários, grades, mesas, assentos e superfícies de contato) antes da abertura do evento e após seu término /<br>Higienização a cada 1 hora de superfícies de contato (mesas, corrimão, balcões etc)<br>Higienização a cada 2 horas de banheiro e áreas comuns de maior circulação /<br>Intervalo mín. de 1 hora entre as apresentações com troca de público, para permitir higienização e evitar aglomerações /<br>Início e término não concomitantes de programações com troca de público /<br>Reforço nos EPIs de colaboradores (máscara e faceshield) e higienização constante das mãos /<br>Organização e escalonamento da equipe de trabalhadores em grupos únicos (bolhas) /<br>Instrumentos musicais de uso individual, vedado o compartilhamento /<br>Vedados alimentos e bebidas expostos (mesa de doces, salgados e bebidas) /<br>Serviços de alimentação e bebidas conforme Portaria SES nº 319 conforme protocolo de "Restaurantes" e "Lanchonetes" e Portaria SES nº 319 /	Duração máxima do evento (para o público): **4 horas /**<br>Máscara de uso obrigatório sempre, à exceção do momento do consumo de alimentos ou bebidas, repondo imediatamente depois /<br>Priorização da venda e conferência de ingressos ou convites por meio visual ou digital, sem contato /<br>Registro dos contatos de todos os presentes (trabalhadores e público) e documento jurídico autorizativo de contato para rastreabilidade em caso de posterior confirmação ou suspeita de Covid-19 /<br>Priorização de pagamentos sem contato (contactless) e/ou higienização a cada uso das máquinas de pagamento de cartão com álcool 70% /<br>Disponibilização de totens e dispensers de álcool em gel com acionamento automático, sem contato, e em diferentes locais estratégicos /<br>Kit completo nos banheiros (álcool gel 70%, sabonete líquido, toalhas de papel e lixeira com tampa de acionamento sem uso das mãos) /<br>Vedado consumo de alimentos e de bebidas em pé /<br>Vedado uso de pista de dança /<br>Suspensão de todas as atividades em caso de detecção de surto /	X	X		Portaria SES nº 617<br><br>Portaria SES nº 319<br><br>Decreto Estadual nº 55.240, Art. 21, § 7º e §8º<br><br>Pedido de autorização, conforme número de pessoas (trabalhadores e público) presentes ao mesmo tempo:<br>- **Até 300**: protocolos estaduais;<br>- **300 a 600**: protocolos estaduais (+) pedido de autorização do município sede, encaminhado pela organização do evento;<br>- **600 a 1.200**: protocolos estaduais (+) pedido de autorização da(s) associação(ões) de municípios da Região Covid, encaminhado pelo município sede (aprovação por no mín. 2/3 dos municípios da Região)<br>- **1.200 a 2.500**, no máx.: protocolos estaduais (+) pedido de autorização do Gabinete de Crise, encaminhado pela(s) associação(ões) de municípios da Região Covid, após aprovação dessa(s)
Serviços	104*	Artes, Cultura, Esportes e Lazer	Demais tipos de eventos, em ambiente fechado ou aberto	Fechado						
Serviços	104*	Artes, Cultura, Esportes e Lazer	Serviços de educação física (academias, centros de treinamento, estúdios e similares)	60% trabalhadores	Teletrabalho / Presencial restrito	Presencial restrito,<br>com distanciamento,<br>sem contato físico,<br>material individual /<br>Ocupação de 1 pessoa para cada 6m² de área útil (piscina, academia etc.) /	X	X		Portaria SES nº 582<br><br>Decreto Estadual nº 55.240, Art. 21, §8º
Serviços	104*	Artes, Cultura, Esportes e Lazer	Serviços de educação física em piscina (aberta ou fechada)	60% trabalhadores	Teletrabalho / Presencial restrito	Presencial restrito,<br>com distanciamento,<br>sem contato físico,<br>material individual /<br>Ocupação de 1 pessoa para cada 6m² de área útil (piscina, academia etc.) /	X	X		Portaria SES nº 582

Notas:
(*) Representam agregações de atividades 2 dígitos:
101* = 64, 65, 66
102* = 69, 70, 71, 72, 73, 74, 75
103* = 77, 78, 79, 82
104* = 90, 91, 92, 93
105* = 94, 95, 96, 99

BANDEIRA AMARELA - Serviços

// Atividade				// Critérios específicos de funcionamento (conforme bandeira)			// Protocolos obrigatório (todas as bandeiras)	// Protocolos variáveis (recomendados)		// Restrições adicionais
Grupo	CNAE (2 díg.)	Tipo	Subtipos	**Teto de Operação**<br>Determina o percentual máximo de trabalhadores/público externo presentes no mesmo turno, ao mesmo tempo.<br><br>Deve respeitar ao nº máximo de pessoas no espaço físico, considerando o distanciamento interpessoal mínimo obrigatório (teto de ocupação).	**Modo de Operação**<br>Forma de operação da atividade, respeitando ao teto de operação, ao teto de ocupação do espaço físico e aos protocolos obrigatórios (ao lado).<br><br>**Trabalhadores**	**Atendimento**	**Decreto nº 55.2540:**<br>- Máscara / EPIs,<br>- Distanciamento,<br>- Teto de ocupação,<br>- Higienização,<br>- Proteção de grupo de risco,<br>- Afastamento de casos,<br>- Cuidados com o público,<br>- Atendimento do grupos de risco<br>- Informativo visível (operação, ocupação e cuidados)	**Monitora-mento de tempera- tura**	**Testagem dos trabalha- dores**	Conteúdo completo das normas obrigatórias específicas à atividade: coronavirus.rs.gov.br/portarias-da-ses
Serviços	104*	Artes, Cultura, Esportes e Lazer	Clubes sociais, esportivos e similares	60% trabalhadores<br><br>60% lotação	Teletrabalho /<br>Presencial restrito /<br>Restaurantes, bares, lanchonetes e espaços coletivos de alimentação: conforme protocolo de "Restaurantes" e "Lanchonetes" e Portaria SES nº 319 /	Presencial restrito,<br>com distanciamento,<br>sem contato físico,<br>material individual /<br>Ocupação de 1 pessoa para cada **6m²** de área útil (piscina, academia etc.) /<br><br>Esportes coletivos (duas ou mais pessoas) exclusivamente em quadras esportivas, sem público, com intervalo de 1 hora entre os jogos e uso intercalado das quadras, para evitar aglomeração e permitir higienização /<br><br>Equipamentos, espreguiçadeiras, brinquedos infantis: distanciamento mínimo de 4m e higienização constante com álcool 70% ou solução sanitizante similar /<br>Área de piscinas e águas, saunas, academias, quadras etc.: conforme protocolo de "Serviços de educação física (academias, centros de treinamento, estúdios e similares)", "Serviços de educação física em piscina (aberta ou fechada)" e Portaria SES nº 582 e alterações /<br>Eventos: conforme protocolos de "Eventos sociais e de entretenimento em ambiente aberto ou fechado" e Portaria SES nº 617 /<br>Atividades tradicionalistas de dança e ensaios conforme protocolo de "Ensino de Esportes, Dança e Artes Cênicas"	X	X		Portaria SES nº 319<br><br>Portaria SES nº 582<br><br>Portaria SES nº 617<br><br>Decreto Estadual nº 55.240, Art. 21, §8º

DISTANCIAMENTO CONTROLADO
.GOV RS
NOVAS FAÇANHAS

Notas:
(*) Representam agregações de atividades 2 dígitos:
101* = 64, 65, 66
102* = 69, 70, 71, 72, 73, 74, 75
103* = 77, 78, 79, 82
104* = 90, 91, 92, 93
105* = 94, 95, 96, 99

BANDEIRA AMARELA - Serviços

// Atividade				// Critérios específicos de funcionamento (conforme bandeira)			// Protocolos obrigatório (todas as bandeiras)	// Protocolos variáveis (recomendados)		// Restrições adicionais
Grupo	CNAE (2 díg.)	Tipo	Subtipos	**Teto de Operação**<br>Determina o percentual máximo de trabalhadores/público externo presentes no mesmo turno, ao mesmo tempo.<br><br>Deve respeitar ao nº máximo de pessoas no espaço físico, considerando o distanciamento interpessoal mínimo obrigatório (teto de ocupação).	**Modo de Operação**<br>Forma de operação da atividade, respeitando ao teto de operação, ao teto de ocupação do espaço físico e aos protocolos obrigatórios (ao lado).		**Decreto nº 55.2540:**<br>- Máscara / EPIs,<br>- Distanciamento,<br>- Teto de ocupação,<br>- Higienização,<br>- Proteção de grupo de risco,<br>- Afastamento de casos,<br>- Cuidados com o público,<br>- Atendimento do grupos de risco<br>- Informativo visível (operação, ocupação e cuidados)	**Monitora-mento de tempera- tura**	**Testagem dos trabalha- dores**	Conteúdo completo das normas obrigatórias específicas à atividade: coronavirus.rs.gov.br/portarias-da-ses
					**Trabalhadores**	**Atendimento**				
Serviços	104*	Artes, Cultura, Esportes e Lazer	Clubes de futebol profissional em disputa no Campeonato Gaúcho (Gauchão Ipiranga 2020), no Campeonato Brasileiro 2020 e na Copa Libertadores (Conmebol Libertadores 2020)	25% trabalhadores	Teletrabalho /<br>Presencial restrito,<br>com atendimento integral dos protocolos da FGF, da CBF, da Conmebol e<br>das recomendações do Comitê Científico (Nota Resposta de 08/07/2020)	Treinos e jogos coletivos, exclusivos de atletas profissionais / Sem público	X	X	X	Protocolos da Federação Gaúcha de Futebol (FGF), Recomendações do Comitê Científico (Nota Resposta de 08/07/2020), Guia Médico de Sugestões Protetivas Para o Retorno às Atividades do Futebol Brasileiro (CBF), Diretriz Técnico Operacional de Retorno das Competições (CBF), Protocolo de operações para o reinício das competições de clubes da Conmebol; Protocolo de recomendações médicas para treinamentos, viagens e competições durante a pandemia COVID-19 da Conmebol; Concentração Sanitária: disposições da Conmebol para diminuir o contágio - com risco médico aceitável - do Coronavírus (COVID-19) durante a reativação do futebol Sul-Americano.
Serviços	104*	Artes, Cultura, Esportes e Lazer	Competições esportivas	50% trabalhadores	Teletrabalho /<br>Presencial restrito,<br>com atendimento integral da Nota Informativa nº 18 COE SES-RS de 13/08/2020 (+)<br>Autorização do(s) município(s) sede	Atendimento coletivo exclusivo de atletas /<br>Sem público	X	X	X	Nota Informativa nº 18 COE SES-RS de 13/08/2020
Serviços	105*	Outros Serviços	Outros Serviços - Outros	25% trabalhadores	Teletrabalho /<br>Presencial restrito	Teleatendimento /<br>Presencial restrito	X			
Serviços	105*	Outros Serviços	Reparação e manutenção de objetos e equipamentos	75% trabalhadores	Teletrabalho /<br>Presencial restrito	Teleatendimento /<br>Presencial restrito	X			
Serviços	105*	Outros Serviços	Lavanderias e similares	75% trabalhadores	Teletrabalho /<br>Presencial restrito	Presencial restrito /<br>Tele-entrega /<br>Pegue e leve	X			
Serviços	105*	Outros Serviços	Serviços de higiene pessoal (cabelereiro e barbeiro)	25% trabalhadores	Teletrabalho /<br>Presencial restrito	Atendimento individualizado,<br>por ambiente	X			
Serviços	105*	Outros Serviços	Serviços de higiene e alojamento de animais domésticos (*petshop*)	25% trabalhadores	Teletrabalho /<br>Presencial restrito	Teleatendimento /<br>Presencial restrito	X			

Notas:

(*) Representam agregações de atividades 2 dígitos:

101* = 64, 65, 66
102* = 69, 70, 71, 72, 73, 74, 75
103* = 77, 78, 79, 82
104* = 90, 91, 92, 93
105* = 94, 95, 96, 99

BANDEIRA AMARELA - Serviços

// Atividade				// Critérios específicos de funcionamento (conforme bandeira)			// Protocolos obrigatório (todas as bandeiras)	// Protocolos variáveis (recomendados)		// Restrições adicionais
Grupo	CNAE (2 díg.)	Tipo	Subtipos	**Teto de Operação**<br>Determina o percentual máximo de trabalhadores/público externo presentes no mesmo turno, ao mesmo tempo.<br><br>Deve respeitar ao nº máximo de pessoas no espaço físico, considerando o distanciamento interpessoal mínimo obrigatório (teto de ocupação).	**Modo de Operação**<br>Forma de operação da atividade, respeitando ao teto de operação, ao teto de ocupação do espaço físico e aos protocolos obrigatórios (ao lado).<br><br>**Trabalhadores**	**Atendimento**	**Decreto nº 55.2540:**<br>- Máscara / EPIs,<br>- Distanciamento,<br>- Teto de ocupação,<br>- Higienização,<br>- Proteção de grupo de risco,<br>- Afastamento de casos,<br>- Cuidados com o público,<br>- Atendimento do grupos de risco<br>- Informativo visível (operação, ocupação e cuidados)	**Monitora- mento de tempera- tura**	**Testagem dos trabalha- dores**	Conteúdo completo das normas obrigatórias específicas à atividade: <u>coronavirus.rs.gov.br/portarias-da-ses</u>
Serviços	105*	Outros Serviços	Missas e serviços religiosos	50% público	Teletrabalho /<br>Presencial restrito /<br>Restaurantes, bares, lanchonetes e espaços coletivos de alimentação: conforme protocolo de "Restaurantes" e "Lanchonetes" e Portaria SES nº 319 /	Presencial restrito /<br>SE PERMITIDO o consumo de bebidas ou alimentos, ocupação intercalada de assentos, respeitando distanciamento mínimo de 2m entre pessoas e/ou grupos de coabitantes /<br>SE NÃO PERMITIDO o consumo de bebidas ou alimentos, ocupação intercalada de assentos, respeitando distanciamento mínimo de 1m entre pessoas e/ou grupos de coabitantes /<br>Obrigatória a utilização de máscaras /<br>Atendimento individualizado	X			
Serviços	105*	Outros Serviços	Festas, festejos e processões religiosas ou similares, em ambiente púbico ou privado, aberto ou fechado	<u>Vedada</u> qualquer atividade que não esteja de acordo com o Modo de Operação deste subtipo.		Permitido apenas manifestações <u>individuais</u> ou em grupos de no <u>máx. 10 pessoas</u>, com uso obrigatório de máscara e distanciamento interpessoal de no mín. 1 metro.<br><br><u>Carreatas</u> permitidas, com permanência das pessoas exclusivamente no interior dos veículos.<br><br><u>Vedada qualquer aglomeração</u>, sujeita à fiscalização e à dispersão pelas autoridades.	X			
Serviços	105*	Outros Serviços	Funerária	100% trabalhadores	Teletrabalho /<br>Presencial restrito	Teleatendimento /<br>Presencial restrito<br>(máx. 10, se Covid-19)	X			
Serviços	105*	Outros Serviços	Organizações sindicais, patronais, empresariais e profissionais	50% trabalhadores	Teletrabalho /<br>Presencial restrito	Teleatendimento /<br>Presencial restrito	X			
Serviços	105*	Outros Serviços	Atividades administrativas dos serviços sociais autônomos	50% trabalhadores	Teletrabalho /<br>Presencial restrito	Teleatendimento /<br>Presencial restrito	X			Nas atividades-fim, observar protocolos específicos conforme medidas sanitárias segmentadas neste decreto.
Serviços	101*	Serv. Financeiros	Bancos, lotéricas e similares	75% trabalhadores	Teletrabalho /<br>Presencial restrito	Teleatendimento /<br>Presencial restrito	X			
Serviços	68	Serv. Imobiliário	Imobiliárias e similares	50% trabalhadores	Teletrabalho /<br>Presencial restrito	Teleatendimento /<br>Presencial restrito	X			
Serviços	102*	Serv. Profissionais, Científicas e Técnicas	Serviços de auditoria, consultoria, engenharia, arquitetura, publicidade e outros	50% trabalhadores	Teletrabalho /<br>Presencial restrito	Teleatendimento /<br>Presencial restrito	X			
Serviços	102*	Serv. Profissionais, Científicas e Técnicas	Serviços profissionais de advocacia e de contabilidade	75% trabalhadores	Teletrabalho /<br>Presencial restrito	Teleatendimento /<br>Presencial restrito	X			
Serviços	103*	Serv. Admin. e Auxiliares	Serv. Admin. e Auxiliares - Outros	50% trabalhadores	Teletrabalho /<br>Presencial restrito	Teleatendimento /<br>Presencial restrito	X			
Serviços	103*	Serv. Admin. e Auxiliares	Agência de turismo, passeios e excursões	50% trabalhadores	Teletrabalho /<br>Presencial restrito	Teleatendimento /<br>Presencial restrito<br>(grupos <u>exclusivo</u> para agências com Selo Turismo Responsável do MTur)	X	X		Selo Turismo Responsável - Ministério do Turismo
Serviços	80	Vigilância, Segurança e Investigação	Vigilância, Segurança e Investigação	75% trabalhadores	Teletrabalho /<br>Presencial restrito		X			

Notas:
(*) Representam agregações de atividades 2 dígitos:
101* = 64, 65, 66
102* = 69, 70, 71, 72, 73, 74, 75
103* = 77, 78, 79, 82
104* = 90, 91, 92, 93
105* = 94, 95, 96, 99

BANDEIRA AMARELA - Serviços

// Atividade				// Critérios específicos de funcionamento (conforme bandeira)			// Protocolos obrigatório (todas as bandeiras)	// Protocolos variáveis (recomendados)		// Restrições adicionais
Grupo	CNAE (2 díg.)	Tipo	Subtipos	**Teto de Operação**<br>Determina o percentual máximo de trabalhadores/público externo presentes no mesmo turno, ao mesmo tempo.<br><br>Deve respeitar ao nº máximo de pessoas no espaço físico, considerando o distanciamento interpessoal mínimo obrigatório (teto de ocupação).	**Modo de Operação**<br>Forma de operação da atividade, respeitando ao teto de operação, ao teto de ocupação do espaço físico e aos protocolos obrigatórios (ao lado).<br><br>**Trabalhadores**	**Atendimento**	**Decreto nº 55.2540:**<br>- Máscara / EPIs,<br>- Distanciamento,<br>- Teto de ocupação,<br>- Higienização,<br>- Proteção de grupo de risco,<br>- Afastamento de casos,<br>- Cuidados com o público,<br>- Atendimento do grupos de risco<br>- Informativo visível (operação, ocupação e cuidados)	**Monitora-mento de tempera- tura**	**Testagem dos trabalha- dores**	Conteúdo completo das normas obrigatórias específicas à atividade: coronavirus.rs.gov.br/portarias-da-ses
Serviços	97	Serv. Domésticos	Faxineiros, cozinheiros, motoristas, babás, jardineiros e similares	50% trabalhadores	Presencial restrito /<br>Obrigatório uso correto da máscara por empregado(s) e empregador(es) durante a prestação do serviço, para proteção de ambos /<br>Circulação de ar cruzada (janelas abertas)		X			
Serviços	81	Condomínios prediais, residenciais e comerciais	*Áreas comuns*	75% trabalhadores	Teletrabalho /<br>Presencial restrito	Teleatendimento /<br>Presencial restrito /<br>Equipamentos, espreguiçadeiras, brinquedos infantis: distanciamento mínimo de 4m e higienização constante com álcool 70% ou solução sanitizante similar /<br>Área de piscinas e águas, saunas, academias, quadras etc.: conforme protocolo de "Serviços de educação física (academias, centros de treinamento, estúdios e similares)", "Serviços de educação física em piscina (aberta ou fechada)" e Portaria SES nº 582 e alterações /<br>Eventos: conforme protocolos de "Eventos sociais e de entretenimento em ambiente aberto ou fechado" e Portaria SES nº 617 /<br>Restaurantes, lanchonetes e espaços coletivos de alimentação: conforme protocolo de "Restaurantes e Lanchonetes" e Portaria SES nº 319 /	X			Portaria SES nº 319<br><br>Portaria SES nº 582<br><br>Portaria SES nº 617
Serviços	81	Condomínios prediais, residenciais e comerciais	Serviços de Limpeza e Manutenção de eficícios e condomínios	75% trabalhadores	Teletrabalho /<br>Presencial restrito		X			
Serviços	72	Serv. Profissionais, Científicas e Técnicas	Pesquisa científica e laboratórios (pandemia)	100% trabalhadores	Teletrabalho /<br>Presencial restrito		X			
Serviços	82	Serv. Admin. e Auxiliares	Call-center	50% trabalhadores	Teletrabalho /<br>Presencial restrito	Teleatendimento	X			

Notas:
(*) Representam agregações de atividades 2 dígitos:
101* = 64, 65, 66
102* = 69, 70, 71, 72, 73, 74, 75
103* = 77, 78, 79, 82
104* = 90, 91, 92, 93
105* = 94, 95, 96, 99

BANDEIRA LARANJA - Serviços

// Atividade				// Critérios específicos de funcionamento (conforme bandeira)			// Protocolos obrigatório (todas as bandeiras)	// Protocolos variáveis (recomendados)		// Restrições adicionais
Grupo	CNAE (2 díg.)	Tipo	Subtipos	**Teto de Operação**<br>Determina o percentual máximo de trabalhadores/público externo presentes no mesmo turno, ao mesmo tempo.<br><br>Deve respeitar ao nº máximo de pessoas no espaço físico, considerando o distanciamento interpessoal mínimo obrigatório (teto de ocupação).	**Modo de Operação**<br>Forma de operação da atividade, respeitando ao teto de operação, ao teto de ocupação do espaço físico e aos protocolos obrigatórios (ao lado).<br><br>**Trabalhadores**	**Atendimento**	**Decreto nº 55.2540:**<br>- Máscara / EPIs,<br>- Distanciamento,<br>- Teto de ocupação,<br>- Higienização,<br>- Proteção de grupo de risco,<br>- Afastamento de casos,<br>- Cuidados com o público,<br>- Atendimento do grupos de risco<br>- Informativo visível (operação, ocupação e cuidados)	**Monitora-mento de tempera- tura**	**Testagem dos trabalha- dores**	Conteúdo completo das normas obrigatórias específicas à atividade: coronavirus.rs.gov.br/portarias-da-ses
Serviços	104*	Artes, Cultura, Esportes e Lazer	Parques Temáticos, Parques de Diversão, Parques de Aventura, Parques Aquáticos, Atrativos Turísticos e Similares - fixos ou itinerantes	50% trabalhadores<br><br>25% público	Teletrabalho /<br>Presencial restrito /<br>Restaurantes, bares, lanchonetes e espaços coletivos de alimentação: conforme protocolo de "Restaurantes" e "Lanchonetes" e Portaria SES nº 319 /	Teleatendimento /<br>Presencial restrito<br>(exclusivo locais com Selo Turismo Responsável do MTur)	X	X		Selo Turismo Responsável - Ministério do Turismo
Serviços	104*	Artes, Cultura, Esportes e Lazer	Parques e reservas naturais, jardins botânicos e zoológicos	50% trabalhadores<br><br>25% público	Teletrabalho /<br>Presencial restrito /<br>Restaurantes, bares, lanchonetes e espaços coletivos de alimentação: conforme protocolo de "Restaurantes" e "Lanchonetes" e Portaria SES nº 319 /	Teleatendimento /<br>Presencial restrito<br>(somentes áreas externas, com demarcação no chão de áreas de permanência distanciada de grupos - máx. 8 pessoas)	X	X		Selo Turismo Responsável - Ministério do Turismo
Serviços	104*	Artes, Cultura, Esportes e Lazer	Teatros, auditórios, casas de espetáculos, casas de show, circos e similares<br><br>(em ambiente aberto ou fechado, com público exclusivamente sentado e restrito ao período da apresentação)	**Ambientes FECHADOS E ABERTOS**: permitidos.<br><br>Respeitando à **lotação**, ao **distanciamento** e à necessidade de **autorização**, conforme número total de pessoas (ver "Restrições Adicionais").<br><br>Local permite consumo de alimentos/ bebidas:<br><br>- PERMITE - **40% de lotação, com** distanciamento de 2m<br><br>- NÃO PERMITE - **50% de lotação**, com distanciamento de 1m	Teletrabalho /<br>Presencial restrito /<br>Para ambiente aberto, permitido instalação de toldo ou cobertura, desde que as laterais sejam inteiramente abertas, para plena circulação de ar /<br>Elaboração de projeto (croqui e protocolos), disponível para fiscalização e/ou autorização, quando exigido /<br>Circulação de ar cruzada ou sistema de renovação de ar /<br>Início e término de programações não concomitantes, quando houver multissalas /<br>Intervalo mín. de 1 hora entre as apresentações com troca de público, para permitir higienização e evitar aglomerações /<br>Restaurantes, bares, lanchonetes e espaços coletivos de alimentação: conforme protocolo de "Restaurantes" e "Lanchonetes" e Portaria SES nº 319 /	Presencial restrito /<br>Máscara de uso obrigatório /<br>Reforço na comunicação sonora e visual dos protocolos de higiene e distanciamento para público e colaboradores /<br>Circulação em pé somente para uso dos sanitários, com uso de máscara e fila com distanciamento demarcado /<br>Vedado interação física entre artistas e público /<br><br>PERMITE ALIMENTAÇÃO/ BEBIDA: distanciamento mínimo de 2m entre pessoas e/ou grupos de coabitantes OU ocupação intercalada de assentos (sim/não/não/sim) e ocupação intercalada das fileiras /<br><br>NÃO PERMITE ALIMENTAÇÃO/ BEBIDA: distanciamento mínimo de 1m entre pessoas e/ou grupos de coabitantes OU ocupação intercalada de assentos (sim/não/não/sim), sem ocupação de assento(s) imediatamente à frente e atrás /	X	X		Portaria SES nº 617<br><br>Portaria SES nº 319<br><br>Decreto Estadual nº 55.240, Art. 21, § 7º e §8º<br><br>Pedido de autorização, conforme número de pessoas (trabalhadores e público) presentes ao mesmo tempo:<br>- **Até 300**: protocolos estaduais;<br>- **300 a 600**: protocolos estaduais (+) pedido de autorização do município sede, encaminhado pela organização do evento;<br>- **600 a 1.200**: protocolos estaduais (+) pedido de autorização da(s) associação(ões) de municípios da Região Covid, encaminhado pelo município sede (aprovação por no mín. 2/3 dos municípios da Região)<br>- **1.200 a 2.500**, no máx.: protocolos estaduais (+) pedido de autorização do Gabinete de Crise, encaminhado pela(s) associação(ões) de municípios da Região Covid, após aprovação dessa(s)
Serviços	104*	Artes, Cultura, Esportes e Lazer	Espetáculos tipo drive-in (cinema, shows, etc.)	75% vagas,<br><br>com distanciamento	Teletrabalho /<br>Presencial restrito /<br>Sem contato físico /<br>Alimentos e bebidas solicitados por aplicativo e entregues no carro	Teleatendimento /<br>Presencial restrito /<br>Público somente nos automóveis /<br>Vedada abertura de portas e circulação externa aos automóveis /<br>Circulação somente para uso dos sanitários, com uso de máscara e fila com distanciamento demarcado	X	X		Portaria SES nº 319<br><br>Decreto Estadual nº 55.240, Art. 21, §8º

Notas:
(*) Representam agregações de atividades 2 dígitos:
101* = 64, 65, 66
102* = 69, 70, 71, 72, 73, 74, 75
103* = 77, 78, 79, 82
104* = 90, 91, 92, 93
105* = 94, 95, 96, 99

BANDEIRA LARANJA - Serviços

// Atividade				// Critérios específicos de funcionamento (conforme bandeira)			// Protocolos obrigatório (todas as bandeiras)	// Protocolos variáveis (recomendados)		// Restrições adicionais
Grupo	CNAE (2 díg.)	Tipo	Subtipos	**Teto de Operação**<br>Determina o percentual máximo de trabalhadores/público externo presentes no mesmo turno, ao mesmo tempo.<br><br>Deve respeitar ao nº máximo de pessoas no espaço físico, considerando o distanciamento interpessoal mínimo obrigatório (teto de ocupação).	**Modo de Operação**<br>Forma de operação da atividade, respeitando ao teto de operação, ao teto de ocupação do espaço físico e aos protocolos obrigatórios (ao lado).<br><br>**Trabalhadores**	**Atendimento**	**Decreto nº 55.2540:**<br>- Máscara / EPIs,<br>- Distanciamento,<br>- Teto de ocupação,<br>- Higienização,<br>- Proteção de grupo de risco,<br>- Afastamento de casos,<br>- Cuidados com o público,<br>- Atendimento do grupos de risco<br>- Informativo visível (operação, ocupação e cuidados)	**Monitora-mento de tempera- tura**	**Testagem dos trabalha- dores**	Conteúdo completo das normas obrigatórias específicas à atividade: coronavirus.rs.gov.br/portarias-da-ses
Serviços	104*	Artes, Cultura, Esportes e Lazer	Cinemas	**Permitido,** respeitando à **lotação** e ao **distanciamento:**<br><br>Estabelecimento permite CONSUMO DE ALIMENTOS OU BEBIDAS:<br><br>- PERMITE - **40% de lotação**, com distanciamento de 2m<br><br>- NÃO PERMITE - **50% de lotação**, com distanciamento de 1m	Teletrabalho /<br>Presencial restrito /<br>Elaboração de projeto (croqui e protocolos), disponível para fiscalização e/ou autorização, quando exigido /<br>Circulação de ar cruzada ou sistema de renovação de ar /<br>Início e término de programações não concomitantes, quando houver multissalas /<br>Intervalo mín. de 1 hora entre as apresentações com troca de público, para permitir higienização e evitar aglomerações /<br>Restaurantes, bares, lanchonetes e espaços coletivos de alimentação: conforme protocolo de "Restaurantes" e "Lanchonetes" e Portaria SES nº 319 /	Presencial restrito /<br>Máscara de uso obrigatório /<br>Reforço na comunicação sonora e visual dos protocolos de higiene e distanciamento para público e colaboradores /<br>Circulação em pé somente para uso dos sanitários, com uso de máscara e fila com distanciamento demarcado /<br><br>PERMITE ALIMENTAÇÃO/BEBIDA: distanciamento mínimo de 2m entre pessoas e/ou grupos de coabitantes OU ocupação intercalada de assentos (sim/não/não/sim) e ocupação intercalada das fileiras /<br><br>NÃO PERMITE ALIMENTAÇÃO/BEBIDA: distanciamento mínimo de 1m entre pessoas e/ou grupos de coabitantes OU ocupação intercalada de assentos (sim/não/não/sim), sem ocupação de assento(s) imediatamente à frente e atrás /	X	X		Portaria SES nº 319<br><br>Decreto Estadual nº 55.240, Art. 21, §8º
Serviços	104*	Artes, Cultura, Esportes e Lazer	Museus, centros culturais e similares	50% trabalhadores<br><br>25% público	Teletrabalho /<br>Presencial restrito /<br>Restaurantes, bares, lanchonetes e espaços coletivos de alimentação: conforme protocolo de "Restaurantes" e "Lanchonetes" e Portaria SES nº 319 /	Teleatendimento /<br>Presencial restrito	X	X		Recomendações aos Museus em Tempos de Covid-19, do Instituto Brasileiro de Museus (Ibram)
Serviços	104*	Artes, Cultura, Esportes e Lazer	Bibliotecas, arquivos, acervos e similares	50% trabalhadores<br><br>25% público	Teletrabalho/<br>Presencial restrito	Teleatendimento /<br>Presencial restrito	X			
Serviços	104*	Artes, Cultura, Esportes e Lazer	Ateliês (artes plásticas, restauração de obras de arte, escrita, artistas independentes e similares)	25% trabalhadores	Teletrabalho/<br>Presencial restrito	Teleatendimento /<br>Atendimento individualizado, com agendamento	X			
Serviços	104*	Artes, Cultura, Esportes e Lazer	Atividades de organizações associativas ligadas à arte e à cultura (MTG e similares)	25% trabalhadores	Teletrabalho/<br>Presencial restrito	Teleatendimento /<br>Atendimento individualizado, com agendamento	X	X		
Serviços	104*	Artes, Cultura, Esportes e Lazer	Convenções partidárias	30% lotação<br><br>Máx. 70 pessoas, ao mesmo tempo	Teletrabalho /<br>Presencial restrito /<br>Circulação de ar cruzada /<br>Credenciamento e check-in online	Presencial restrito /<br>Cadeiras intercalados (sim/não/não/sim) /<br>Filas intercaladas /<br>10m² por pessoa /<br>Entrada e saída escalonada por filas previamente demarcadas /<br>Material individual (canetas)	X			

Notas:
(*) Representam agregações de atividades 2 dígitos:
101* = 64, 65, 66
102* = 69, 70, 71, 72, 73, 74, 75
103* = 77, 78, 79, 82
104* = 90, 91, 92, 93
105* = 94, 95, 96, 99

BANDEIRA LARANJA - Serviços

// Atividade				// Critérios específicos de funcionamento (conforme bandeira)			// Protocolos obrigatório (todas as bandeiras)	// Protocolos variáveis (recomendados)		// Restrições adicionais
Grupo	CNAE (2 díg.)	Tipo	Subtipos	**Teto de Operação**<br>Determina o percentual máximo de trabalhadores/público externo presentes no mesmo turno, ao mesmo tempo.<br><br>Deve respeitar ao nº máximo de pessoas no espaço físico, considerando o distanciamento interpessoal mínimo obrigatório (teto de ocupação).	**Modo de Operação**<br>Forma de operação da atividade, respeitando ao teto de operação, ao teto de ocupação do espaço físico e aos protocolos obrigatórios (ao lado).<br><br>**Trabalhadores**	**Atendimento**	**Decreto nº 55.2540:**<br>- Máscara / EPIs,<br>- Distanciamento,<br>- Teto de ocupação,<br>- Higienização,<br>- Proteção de grupo de risco,<br>- Afastamento de casos,<br>- Cuidados com o público,<br>- Atendimento do grupos de risco<br>- Informativo visível (operação, ocupação e cuidados)	**Monitora- mento de tempera- tura**	**Testagem dos trabalha- dores**	Conteúdo completo das normas obrigatórias específicas à atividade: coronavirus.rs.gov.br/portarias-da-ses
Serviços	104*	Artes, Cultura, Esportes e Lazer	Feiras e Exposições corporativas e comerciais	**Permitido**, respeitando à **lotação**, ao **distanciamento** e à necessidade de **autorização**, conforme número total de pessoas (ver "Restrições Adicionais").	Teletrabalho /<br>Presencial restrito /<br>Elaboração de projeto (croqui e protocolos), disponível para fiscalização e/ou autorização, quando couber /<br>Módulos de estandes distanciados 4 metros um do outro /<br>Circulação de ar cruzada /<br>Credenciamento e check-in online /<br>Início e término de programações não concomitantes, quando houver multissalas /<br>Restaurantes, bares, lanchonetes e espaços coletivos de alimentação: conforme protocolo de "Restaurantes" e "Lanchonetes" e Portaria SES nº 319 /	Teleatendimento /<br>Presencial restrito /<br>Ambientes (estandes, salas, corredores, etc.) com circulação em pé: contabilizar mínimo de 8m² por pessoa /<br>Ambientes com público sentado: contabilizar mínimo de 4m² por pessoa, considerando se o local permite alimentação ou bebida:<br><br>PERMITE ALIMENTAÇÃO/BEBIDA: distanciamento mínimo de 2m entre pessoas e/ou grupos de coabitantes OU ocupação intercalada de assentos (sim/não/não/sim) e ocupação intercalada das fileiras /<br><br>NÃO PERMITE ALIMENTAÇÃO/BEBIDA: distanciamento mínimo de 1m entre pessoas e/ou grupos de coabitantes OU ocupação intercalada de assentos (sim/não/não/sim), sem ocupação de assento(s) imediatamente à frente e atrás /	X	X		Portaria SES nº 617<br><br>Portaria SES nº 319<br><br>Decreto Estadual nº 55.240, Art. 21, § 7º e §8º<br><br>Pedido de autorização, conforme número de pessoas (trabalhadores e público) presentes ao mesmo tempo:<br>- **Até 300**: protocolos estaduais;<br>- **300 a 600**: protocolos estaduais (+) pedido de autorização do município sede, encaminhado pela organização do evento;<br>- **600 a 1.200**: protocolos estaduais (+) pedido de autorização da(s) associação(ões) de municípios da Região Covid, encaminhado pelo município sede (aprovação por no mín. 2/3 dos municípios da Região)<br>- **1.200 a 2.500**, no máx.: protocolos estaduais (+) pedido de autorização do Gabinete de Crise, encaminhado pela(s) associação(ões) de municípios da Região Covid, após aprovação dessa(s)

DISTANCIAMENTO CONTROLADO
GOV RS
NOVAS FAÇANHAS

Notas:
(*) Representam agregações de atividades 2 dígitos:
101* = 64, 65, 66
102* = 69, 70, 71, 72, 73, 74, 75
103* = 77, 78, 79, 82
104* = 90, 91, 92, 93
105* = 94, 95, 96, 99

BANDEIRA LARANJA - Serviços

// Atividade				// Critérios específicos de funcionamento (conforme bandeira)			// Protocolos obrigatório (todas as bandeiras)	// Protocolos variáveis (recomendados)		// Restrições adicionais
Grupo	CNAE (2 díg.)	Tipo	Subtipos	Teto de Operação<br>Determina o percentual máximo de trabalhadores/público externo presentes no mesmo turno, ao mesmo tempo.<br><br>Deve respeitar ao nº máximo de pessoas no espaço físico, considerando o distanciamento interpessoal mínimo obrigatório (teto de ocupação).	Modo de Operação<br>Forma de operação da atividade, respeitando ao teto de operação, ao teto de ocupação do espaço físico e aos protocolos obrigatórios (ao lado).<br><br>Trabalhadores	Atendimento	Decreto nº 55.2540:<br>- Máscara / EPIs,<br>- Distanciamento,<br>- Teto de ocupação,<br>- Higienização,<br>- Proteção de grupo de risco,<br>- Afastamento de casos,<br>- Cuidados com o público,<br>- Atendimento do grupos de risco<br>- Informativo visível (operação, ocupação e cuidados)	Monitora-mento de tempera- tura	Testagem dos trabalha- dores	Conteúdo completo das normas obrigatórias específicas à atividade: coronavirus.rs.gov.br/portarias-da-ses
Serviços	104*	Artes, Cultura, Esportes e Lazer	Seminários, congressos, convenções, simpósios e similares	**Permitido**, respeitando à **lotação**, ao **distanciamento** e à necessidade de **autorização**, conforme número total de pessoas (ver "Restrições Adicionais").	Teletrabalho /<br>Presencial restrito /<br>Elaboração de projeto (croqui e protocolos), disponível para fiscalização e/ou autorização, quando couber /<br>Módulos de estandes distanciados 4 metros um do outro /<br>Circulação de ar cruzada /<br>Credenciamento e check-in online /<br>Início e término de programações não concomitantes, quando houver multissalas /<br>Restaurantes, bares, lanchonetes e espaços coletivos de alimentação: conforme protocolo de "Restaurantes" e "Lanchonetes" e Portaria SES nº 319 /	Teleatendimento /<br>Presencial restrito /<br>Ambientes (estandes, salas, corredores, etc.) com circulação em pé: contabilizar mínimo de 8m² por pessoa /<br>Ambientes com público sentado: contabilizar mínimo de 4m² por pessoa, considerando se o local permite alimentação ou bebida:<br><br>PERMITE ALIMENTAÇÃO/BEBIDA: distanciamento mínimo de 2m entre pessoas e/ou grupos de coabitantes OU ocupação intercalada de assentos (sim/não/não/sim) e ocupação intercalada das fileiras /<br><br>NÃO PERMITE ALIMENTAÇÃO/BEBIDA: distanciamento mínimo de 1m entre pessoas e/ou grupos de coabitantes OU ocupação intercalada de assentos (sim/não/não/sim), sem ocupação de assento(s) imediatamente à frente e atrás /	X	X		Portaria SES nº 617<br><br>Portaria SES nº 319<br><br>Decreto Estadual nº 55.240, Art. 21, § 7º e §8º<br><br>Pedido de autorização, conforme número de pessoas (trabalhadores e público) presentes ao mesmo tempo:<br>- **Até 300**: protocolos estaduais;<br>- **300 a 600**: protocolos estaduais (+) pedido de autorização do município sede, encaminhado pela organização do evento;<br>- **600 a 1.200**: protocolos estaduais (+) pedido de autorização da(s) associação(ões) de municípios da Região Covid, encaminhado pelo município sede (aprovação por no mín. 2/3 dos municípios da Região)<br>- **1.200 a 2.500**, no máx.: protocolos estaduais (+) pedido de autorização do Gabinete de Crise, encaminhado pela(s) associação(ões) de municípios da Região Covid, após aprovação dessa(s)
Serviços	104*	Artes, Cultura, Esportes e Lazer	Reuniões corporativas, oficinas, treinamentos e cursos corporativos	**Máximo de 70 pessoas** (trabalhadores e públicos), respeitando ao **teto de ocupação** e ao **distanciamento** estabelecido no Modo de Atendimento	Teletrabalho /<br>Presencial restrito /<br>Elaboração de projeto (croqui e protocolos), disponível para fiscalização e/ou autorização, quando couber /<br>Módulo de estandes distanciados 4 metros um do outro /<br>Circulação de ar cruzada /<br>Credenciamento e check-in online /<br>Início e término de programações não concomitantes, quando houver multissalas /<br>Restaurantes, bares, lanchonetes e espaços coletivos de alimentação: conforme protocolo de "Restaurantes" e "Lanchonetes" e Portaria SES nº 319 /	Teleatendimento /<br>Presencial restrito /<br>Material individual /<br>Ambientes (estandes, salas, corredores, etc.) com circulação em pé: contabilizar mínimo de 8m² por pessoa /<br>Ambientes com público sentado: contabilizar mínimo de 4m² por pessoa, considerando se o local permite alimentação ou bebida:<br><br>PERMITE ALIMENTAÇÃO/BEBIDA: distanciamento mínimo de 2m entre pessoas e/ou grupos de coabitantes OU ocupação intercalada de assentos (sim/não/não/sim) e ocupação intercalada das fileiras /<br><br>NÃO PERMITE ALIMENTAÇÃO/BEBIDA: distanciamento mínimo de 1m entre pessoas e/ou grupos de coabitantes OU ocupação intercalada de assentos (sim/não/não/sim), sem ocupação de assento(s) imediatamente à frente e atrás /	X	X		Portaria SES nº 617<br><br>Portaria SES nº 319

DISTANCIAMENTO CONTROLADO
GOV RS
NOVAS FAÇANHAS

Notas:
(*) Representam agregações de atividades 2 dígitos:
101* = 64, 65, 66 104* = 90, 91, 92, 93
102* = 69, 70, 71, 72, 73, 74, 75 105* = 94, 95, 96, 99
103* = 77, 78, 79, 82

BANDEIRA LARANJA - Serviços

// Atividade				// Critérios específicos de funcionamento (conforme bandeira)			// Protocolos obrigatório (todas as bandeiras)	// Protocolos variáveis (recomendados)		// Restrições adicionais
Grupo	CNAE (2 díg.)	Tipo	Subtipos	**Teto de Operação**<br>Determina o percentual máximo de trabalhadores/público externo presentes no mesmo turno, ao mesmo tempo.<br><br>Deve respeitar ao nº máximo de pessoas no espaço físico, considerando o distanciamento interpessoal mínimo obrigatório (teto de ocupação).	**Modo de Operação**<br>Forma de operação da atividade, respeitando ao teto de operação, ao teto de ocupação do espaço físico e aos protocolos obrigatórios (ao lado).<br><br>**Trabalhadores**	**Atendimento**	**Decreto nº 55.2540:**<br>- Máscara / EPIs,<br>- Distanciamento,<br>- Teto de ocupação,<br>- Higienização,<br>- Proteção de grupo de risco,<br>- Afastamento de casos,<br>- Cuidados com o público,<br>- Atendimento do grupos de risco<br>- Informativo visível (operação, ocupação e cuidados)	**Monitora-mento de tempera- tura**	**Testagem dos trabalha- dores**	Conteúdo completo das normas obrigatórias específicas à atividade: coronavirus.rs.gov.br/portarias-da-ses
Serviços	104*	Artes, Cultura, Esportes e Lazer	Eventos infantis em buffets, casas de festas ou similares<br><br>(em ambiente aberto ou fechado)	**Máximo de 70 pessoas** (trabalhadores e públicos), respeitando **teto de ocupação** e **distanciamento** estabelecido no Modo de Operação	Teletrabalho /<br>Presencial restrito /<br>Teto de ocupação: mínimo de 8m² por pessoa, respeitando a lotação máxima da bandeira /<br>Elaboração de projeto (croqui e protocolos), disponível para fiscalização e/ou autorização, quando couber /<br>Distanciamento mínimo de 2m entre mesas /<br>Ventilação forçada ou circulação de ar cruzada, com manutenção de janelas e portas abertas, independente do uso de equipamento de climatização /<br>Adesivagem do piso demarcando distanciamento mín. 1m nas filas /<br>Fluxo único de entrada, saída e circulação /<br>Abertura antecipada e ingresso escalonado ao evento /<br>Reforço constante na comunicação visual e sonora dos protocolos de higiene e distanciamento /<br>Distanciamento mín. 2m entre artistas e público, vedado o contato físico /<br>Tapetes sanitizantes em todas as entradas /<br>Higienização de camarins, camarotes e todas as áreas comuns (corredores, portas, elevadores, banheiros, vestiários, mesas, assentos e superfícies de contato) antes da abertura do evento e após seu término /<br>Higienização a cada 1 hora de superfícies de contato (mesas, corrimão, balcões etc) /<br>Higienização a cada 2 horas de banheiro e áreas comuns de maior circulação /<br>Higienização dos brinquedos a cada uso, com álcool 70% e/ou solução sanitizante similar /<br>Intervalo mín. de 1 hora entre as apresentações com troca de público, para permitir higienização e evitar aglomerações /<br>Início e término não concomitantes de programações com troca de público /<br>Reforço nos EPIs de colaboradores (máscara e faceshield) e higienização constante das mãos /<br>Organização e escalonamento da equipe de trabalhadores em grupos únicos (bolhas) /<br>Instrumentos musicais de uso individual, vedado o compartilhamento /<br>Vedados alimentos e bebidas expostos (mesa de doces, salgados e bebidas) /<br>Serviços de alimentação e bebidas conforme Portaria SES nº 319 conforme protocolo de "Restaurantes" e "Lanchonetes" e Portaria	Duração máxima do evento (para o público): **4 horas** /<br>Máscara de uso obrigatório sempre, à exceção do momento do consumo de alimentos ou bebidas, repondo imediatamente depois /<br>Priorização da venda e conferência de ingressos ou convites por meio visual ou digital, sem contato /<br>Registro dos contatos de todos os presentes (trabalhadores e público) e documento jurídico autorizativo de contato para rastreabilidade em caso de posterior confirmação ou suspeita de Covid-19 /<br>Disponibilização de totens e dispensers de álcool em gel com acionamento automático, sem contato, e em diferentes locais estratégicos /<br>Disponibilizar álcool em gel e monitor para orientar sobre o uso da máscara e a correta higienização das mãos antes e depois de acessar os brinquedos /<br>Kit completo nos banheiros (álcool gel 70%, sabonete líquido, toalhas de papel e lixeira com tampa de acionamento sem uso das mãos) /<br>Vedado consumo de alimentos e de bebidas em pé /<br>Vedado uso de bebedouros verticais /<br>Vedado uso de pista de dança /<br>Suspensão de todas as atividades em caso de detecção de surto	X	X		Portaria SES nº 617<br><br>Portaria SES nº 319<br><br>Decreto Estadual nº 55.240, Art. 21, §8º

Notas:
(*) Representam agregações de atividades 2 dígitos:
101* = 64, 65, 66
102* = 69, 70, 71, 72, 73, 74, 75
103* = 77, 78, 79, 82
104* = 90, 91, 92, 93
105* = 94, 95, 96, 99

BANDEIRA LARANJA - Serviços

// Atividade				// Critérios específicos de funcionamento (conforme bandeira)			// Protocolos obrigatório (todas as bandeiras)	// Protocolos variáveis (recomendados)		// Restrições adicionais
Grupo	CNAE (2 díg.)	Tipo	Subtipos	**Teto de Operação**<br>Determina o percentual máximo de trabalhadores/público externo presentes no mesmo turno, ao mesmo tempo.<br><br>Deve respeitar ao nº máximo de pessoas no espaço físico, considerando o distanciamento interpessoal mínimo obrigatório (teto de ocupação).	**Modo de Operação**<br>Forma de operação da atividade, respeitando ao teto de operação, ao teto de ocupação do espaço físico e aos protocolos obrigatórios (ao lado).<br><br>**Trabalhadores**	**Atendimento**	**Decreto nº 55.2540:**<br>- Máscara / EPIs,<br>- Distanciamento,<br>- Teto de ocupação,<br>- Higienização,<br>- Proteção de grupo de risco,<br>- Afastamento de casos,<br>- Cuidados com o público,<br>- Atendimento do grupos de risco<br>- Informativo visível (operação, ocupação e cuidados)	**Monitora- mento de tempera- tura**	**Testagem dos trabalha- dores**	Conteúdo completo das normas obrigatórias específicas à atividade: coronavirus.rs.gov.br/portarias-da-ses
Serviços	104*	Artes, Cultura, Esportes e Lazer	Eventos sociais e de entretenimento em buffets, casas de festas, casas de shows, casas noturnas, bares e pubs ou similares<br><br>(em ambiente fechado, com público em pé)	**Máximo de 70 pessoas** (trabalhadores e públicos), respeitando **teto de ocupação** e **distanciamento** estabelecido no Modo de Operação	Teletrabalho /<br>Presencial restrito /<br>Teto de ocupação: mínimo de 8m² por pessoa, respeitando a lotação máxima da bandeira /<br>Elaboração de projeto (croqui e protocolos), disponível para fiscalização e/ou autorização, quando couber /<br>Distanciamento mínimo de 2m entre mesas /<br>Áreas exclusivas para até 8 coabitantes, com distanciamento mín. 4m e demarcadas no chão (não permite bebida/alimentação) ou por barreira física (permite bebida/alimentação) /<br>Ventilação forçada ou circulação de ar cruzada, com manutenção de janelas e portas abertas, independente do uso de equipamento de climatização /<br>Adesivagem do piso demarcando distanciamento mín. 1m nas filas /<br>Fluxo único de entrada, saída e circulação /<br>Abertura antecipada e ingresso escalonado ao evento /<br>Reforço constante na comunicação visual e sonora dos protocolos de higiene e distanciamento /<br>Distanciamento mín. 2m entre artistas e público, vedado o contato físico /<br>Tapetes sanitizantes em todas as entradas /<br>Higienização de camarins, camarotes e todas as áreas comuns (corredores, portas, elevadores, banheiros, vestiários, grades, mesas, assentos e superfícies de contato) antes da abertura do evento e após seu término /<br>Higienização a cada 1 hora de superfícies de contato (mesas, maçanetas, corrimão, balcões etc)<br>Higienização a cada 2 horas de banheiro e áreas comuns de maior circulação /<br>Intervalo mín. de 1 hora entre as apresentações com troca de público, para permitir higienização e evitar aglomerações /<br>Início e término não concomitantes de programações com troca de público /<br>Reforço nos EPIs de colaboradores (máscara e faceshield) e higienização constante das mãos /<br>Organização e escalonamento da equipe de trabalhadores em grupos únicos (bolhas) /<br>Instrumentos musicais de uso individual, vedado o compartilhamento /<br>Vedados alimentos e bebidas expostos (mesa de doces, salgados e bebidas) /<br>Serviços de alimentação e bebidas conforme Portaria SES nº 319 conforme protocolo de "Restaurantes" e "Lanchonetes" e Portaria SES nº 319 /	Duração máxima do evento (para o público): **4 horas /**<br>Máscara de uso obrigatório sempre, à exceção do momento do consumo de alimentos ou bebidas, repondo imediatamente depois /<br>Priorização da venda e conferência de ingressos ou convites por meio visual ou digital, sem contato /<br>Registro dos contatos de todos os presentes (trabalhadores e público) e documento jurídico autorizativo de contato para rastreabilidade em caso de posterior confirmação ou suspeita de Covid-19 /<br>Priorização de pagamentos sem contato (contactless) e/ou higienização a cada uso das máquinas de pagamento de cartão com álcool 70% /<br>Disponibilização de totens e dispensers de álcool em gel com acionamento automático, sem contato, e em diferentes locais estratégicos /<br>Kit completo nos banheiros (álcool gel 70%, sabonete líquido, toalhas de papel e lixeira com tampa de acionamento sem uso das mãos) /<br>Vedado consumo de alimentos e de bebidas em pé /<br>Vedado uso de pista de dança /<br>Suspensão de todas as atividades em caso de detecção de surto	X	X		Portaria SES nº 617<br><br>Portaria SES nº 319<br><br>Decreto Estadual nº 55.240, Art. 21, §8º

Notas:

(*) Representam agregações de atividades 2 dígitos:

101* = 64, 65, 66
102* = 69, 70, 71, 72, 73, 74, 75
103* = 77, 78, 79, 82
104* = 90, 91, 92, 93
105* = 94, 95, 96, 99

BANDEIRA LARANJA - Serviços

// Atividade				// Critérios específicos de funcionamento (conforme bandeira)			// Protocolos obrigatório (todas as bandeiras)	// Protocolos variáveis (recomendados)		// Restrições adicionais
Grupo	CNAE (2 díg.)	Tipo	Subtipos	Teto de Operação<br>Determina o percentual máximo de trabalhadores/público externo presentes no mesmo turno, ao mesmo tempo.<br><br>Deve respeitar ao nº máximo de pessoas no espaço físico, considerando o distanciamento interpessoal mínimo obrigatório (teto de ocupação).	Modo de Operação<br>Forma de operação da atividade, respeitando ao teto de operação, ao teto de ocupação do espaço físico e aos protocolos obrigatórios (ao lado).<br><br>**Trabalhadores**	**Atendimento**	Decreto nº 55.2540:<br>- Máscara / EPIs,<br>- Distanciamento,<br>- Teto de ocupação,<br>- Higienização,<br>- Proteção de grupo de risco,<br>- Afastamento de casos,<br>- Cuidados com o público,<br>- Atendimento do grupos de risco<br>- Informativo visível (operação, ocupação e cuidados)	Monitora- mento de tempera- tura	Testagem dos trabalha- dores	Conteúdo completo das normas obrigatórias específicas à atividade: coronavirus.rs.gov.br/portarias-da-ses
Serviços	104*	Artes, Cultura, Esportes e Lazer	Eventos sociais e de entretenimento em ambiente aberto, com público em pé	**Permitido**, respeitando à **lotação**, ao **distanciamento** e à necessidade de **autorização, conforme número total de pessoas (ver "Restrições Adicionais").**<br><br>Local permite CONSUMO DE ALIMENTOS OU BEBIDAS:<br><br>- PERMITE - **40% de lotação** do PPCI, respeitando à lotação, ao distanciamento e à necessidade de autorização, conforme número total de pessoas (ver "Restrições Adicionais").<br><br>- NÃO PERMITE - **50% de lotação** do PPCI, respeitando à lotação, ao distanciamento e à necessidade de autorização, conforme número total de pessoas (ver "Restrições Adicionais").	Teletrabalho /<br>Presencial restrito /<br>Teto de ocupação: mínimo de 8m² por pessoa, respeitando a lotação máxima da bandeira /<br>Elaboração de projeto (croqui e protocolos), disponível para fiscalização e/ou autorização, quando couber /<br>Controle de acesso à área do evento /<br>Distanciamento mínimo de 2m entre mesas /<br>Áreas exclusivas para até 8 coabitantes, com distanciamento mín. 4m e demarcadas no chão (não permite bebida/alimentação) ou por barreira física (permite bebida/alimentação) /<br>Adesivagem do piso demarcando distanciamento mín. 1m nas filas /<br>Fluxo único de entrada, saída e circulação /<br>Abertura antecipada e ingresso escalonado ao evento /<br>Reforço constante na comunicação visual e sonora dos protocolos de higiene e distanciamento /<br>Distanciamento mín. 2m entre artistas e público, vedado o contato físico /<br>Tapetes sanitizantes em todas as entradas /<br>Higienização de camarins, camarotes e todas as áreas comuns (corredores, portas, elevadores, banheiros, vestiários, grades, mesas, assentos e superfícies de contato) antes da abertura do evento e após seu término /<br>Higienização a cada 1 hora de superfícies de contato (mesas, corrimão, balcões etc)<br>Higienização a cada 2 horas de banheiro e áreas comuns de maior circulação /<br>Intervalo mín. de 1 hora entre as apresentações com troca de público, para permitir higienização e evitar aglomerações /<br>Início e término não concomitantes de programações com troca de público /<br>Reforço nos EPIs de colaboradores (máscara e faceshield) e higienização constante das mãos /<br>Organização e escalonamento da equipe de trabalhadores em grupos únicos (bolhas) /<br>Instrumentos musicais de uso individual, vedado o compartilhamento /<br>Vedados alimentos e bebidas expostos (mesa de doces, salgados e bebidas) /<br>Serviços de alimentação e bebidas conforme Portaria SES nº 319 conforme protocolo de "Restaurantes" e "Lanchonetes" e Portaria SES nº 319 /	Duração máxima do evento (para o público): **4 horas /**<br>Máscara de uso obrigatório sempre, à exceção do momento do consumo de alimentos ou bebidas, repondo imediatamente depois /<br>Priorização da venda e conferência de ingressos ou convites por meio visual ou digital, sem contato /<br>Registro dos contatos de todos os presentes (trabalhadores e público) e documento jurídico autorizativo de contato para rastreabilidade em caso de posterior confirmação ou suspeita de Covid-19 /<br>Priorização de pagamentos sem contato (contactless) e/ou higienização a cada uso das máquinas de pagamento de cartão com álcool 70% /<br>Disponibilização de totens e dispensers de álcool em gel com acionamento automático, sem contato, e em diferentes locais estratégicos /<br>Kit completo nos banheiros (álcool gel 70%, sabonete líquido, toalhas de papel e lixeira com tampa de acionamento sem uso das mãos) /<br>Vedado consumo de alimentos e de bebidas em pé /<br>Vedado uso de pista de dança /<br>Suspensão de todas as atividades em caso de detecção de surto /		X	X	Portaria SES nº 617<br><br>Portaria SES nº 319<br><br>Decreto Estadual nº 55.240, Art. 21, § 7º e §8º<br><br>Pedido de autorização, conforme número de pessoas (trabalhadores e público) presentes ao mesmo tempo:<br>- **Até 300**: protocolos estaduais;<br>- **300 a 600**: protocolos estaduais (+) pedido de autorização do município sede, encaminhado pela organização do evento;<br>- **600 a 1.200**: protocolos estaduais (+) pedido de autorização da(s) associação(ões) de municípios da Região Covid, encaminhado pelo município sede (aprovação por no mín. 2/3 dos municípios da Região)<br>- **1.200 a 2.500**, no máx.: protocolos estaduais (+) pedido de autorização do Gabinete de Crise, encaminhado pela(s) associação(ões) de municípios da Região Covid, após aprovação dessa(s)
Serviços	104*	Artes, Cultura, Esportes e Lazer	Demais tipos de eventos, em ambiente fechado ou aberto	Fechado						
Serviços	104*	Artes, Cultura, Esportes e Lazer	Serviços de educação física (academias, centros de treinamento, estúdios e similares)	50% trabalhadores	Teletrabalho /<br>Presencial restrito	Presencial restrito,<br>com distanciamento,<br>sem contato físico,<br>material individual /<br>Ocupação de 1 pessoa para cada 10m² de área útil (piscina, academia etc.) /		X	X	Portaria SES nº 582<br><br>Decreto Estadual nº 55.240, Art. 21, §8º
Serviços	104*	Artes, Cultura, Esportes e Lazer	Serviços de educação física em piscina (aberta ou fechada)	50% trabalhadores	Teletrabalho / Presencial restrito	Presencial restrito,<br>com distanciamento,<br>sem contato físico,<br>material individual /<br>Ocupação de 1 pessoa para cada 10m² de área útil (piscina, academia etc.) /		X	X	Portaria SES nº 582

Notas:
(*) Representam agregações de atividades 2 dígitos:
101* = 64, 65, 66
102* = 69, 70, 71, 72, 73, 74, 75
103* = 77, 78, 79, 82
104* = 90, 91, 92, 93
105* = 94, 95, 96, 99

DISTANCIAMENTO CONTROLADO
NOVAS FAÇANHAS

BANDEIRA LARANJA - Serviços

// Atividade				// Critérios específicos de funcionamento (conforme bandeira)			// Protocolos obrigatório (todas as bandeiras)	// Protocolos variáveis (recomendados)		// Restrições adicionais
Grupo	CNAE (2 díg.)	Tipo	Subtipos	**Teto de Operação** Determina o percentual máximo de trabalhadores/público externo presentes no mesmo turno, ao mesmo tempo. Deve respeitar ao nº máximo de pessoas no espaço físico, considerando o distanciamento interpessoal mínimo obrigatório (teto de ocupação).	**Modo de Operação** Forma de operação da atividade, respeitando ao teto de operação, ao teto de ocupação do espaço físico e aos protocolos obrigatórios (ao lado). **Trabalhadores**	**Atendimento**	**Decreto nº 55.2540:** - Máscara / EPIs, - Distanciamento, - Teto de ocupação, - Higienização, - Proteção de grupo de risco, - Afastamento de casos, - Cuidados com o público, - Atendimento do grupos de risco - Informativo visível (operação, ocupação e cuidados)	**Monitora- mento de tempera- tura**	**Testagem dos trabalha- dores**	Conteúdo completo das normas obrigatórias específicas à atividade: coronavirus.rs.gov.br/portarias-da-ses
Serviços	104*	Artes, Cultura, Esportes e Lazer	Clubes sociais, esportivos e similares	50% trabalhadores<br>50% lotação	Teletrabalho / Presencial restrito / Restaurantes, bares, lanchonetes e espaços coletivos de alimentação: conforme protocolo de "Restaurantes" e "Lanchonetes" e Portaria SES nº 319 /	Presencial restrito, com distanciamento, sem contato físico, material individual / Ocupação de 1 pessoa para cada **10m²** de área útil (piscina, academia etc.) /<br><br>Esportes coletivos (duas ou mais pessoas) exclusivamente em quadras esportivas, sem público, com intervalo de 1 hora entre os jogos e uso intercalado das quadras, para evitar aglomeração e permitir higienização /<br><br>Equipamentos, espreguiçadeiras, brinquedos infantis: distanciamento mínimo de 4m e higienização constante com álcool 70% ou solução sanitizante similar / Área de piscinas e águas, saunas, academias, quadras etc.: conforme protocolo de "Serviços de educação física (academias, centros de treinamento, estúdios e similares)", "Serviços de educação física em piscina (aberta ou fechada)" e Portaria SES nº 582 e alterações / Eventos: conforme protocolos de "Eventos sociais e de entretenimento em ambiente aberto ou fechado" e Portaria SES nº 617 / Atividades tradicionalistas de dança e ensaios conforme protocolo de "Ensino de Esportes, Dança e Artes Cênicas"		X	X	Portaria SES nº 319<br><br>Portaria SES nº 582<br><br>Portaria SES nº 617<br><br>Decreto Estadual nº 55.240, Art. 21, §8º

DISTANCIAMENTO CONTROLADO
.GOV RS
NOVAS FAÇANHAS

Notas:
(*) Representam agregações de atividades 2 dígitos:
101* = 64, 65, 66
102* = 69, 70, 71, 72, 73, 74, 75
103* = 77, 78, 79, 82
104* = 90, 91, 92, 93
105* = 94, 95, 96, 99

BANDEIRA LARANJA - Serviços

// Atividade				// Critérios específicos de funcionamento (conforme bandeira)			// Protocolos obrigatório (todas as bandeiras)	// Protocolos variáveis (recomendados)		// Restrições adicionais
Grupo	CNAE (2 díg.)	Tipo	Subtipos	**Teto de Operação**<br>Determina o percentual máximo de trabalhadores/público externo presentes no mesmo turno, ao mesmo tempo.<br><br>Deve respeitar ao nº máximo de pessoas no espaço físico, considerando o distanciamento interpessoal mínimo obrigatório (teto de ocupação).	**Modo de Operação**<br>Forma de operação da atividade, respeitando ao teto de operação, ao teto de ocupação do espaço físico e aos protocolos obrigatórios (ao lado).<br><br>**Trabalhadores**	**Atendimento**	**Decreto nº 55.2540:**<br>- Máscara / EPIs,<br>- Distanciamento,<br>- Teto de ocupação,<br>- Higienização,<br>- Proteção de grupo de risco,<br>- Afastamento de casos,<br>- Cuidados com o público,<br>- Atendimento do grupos de risco<br>- Informativo visível (operação, ocupação e cuidados)	**Monitora- mento de tempera- tura**	**Testagem dos trabalha- dores**	Conteúdo completo das normas obrigatórias específicas à atividade: coronavirus.rs.gov.br/portarias-da-ses
Serviços	104*	Artes, Cultura, Esportes e Lazer	Clubes de futebol profissional em disputa no Campeonato Gaúcho (Gauchão Ipiranga 2020), no Campeonato Brasileiro 2020 e na Copa Libertadores (Conmebol Libertadores 2020)	25% trabalhadores	Teletrabalho /<br>Presencial restrito,<br>com atendimento integral dos protocolos da FGF, da CBF, da Conmebol e<br>das recomendações do Comitê Científico (Nota Resposta de 08/07/2020)	Treinos e jogos coletivos, exclusivos de atletas profissionais<br>/<br>Sem público	X	X	X	Protocolos da Federação Gaúcha de Futebol (FGF), Recomendações do Comitê Científico (Nota Resposta de 08/07/2020), Guia Médico de Sugestões Protetivas Para o Retorno às Atividades do Futebol Brasileiro (CBF), Diretriz Técnico Operacional de Retorno das Competições (CBF), Protocolo de operações para o reinício das competições de clubes da Conmebol; Protocolo de recomendações médicas para treinamentos, viagens e competições durante a pandemia COVID-19 da Conmebol; Concentração Sanitária: disposições da Conmebol para diminuir o contágio - com risco médico aceitável - do Coronavírus (COVID-19) durante a reativação do futebol Sul-Americano.
Serviços	104*	Artes, Cultura, Esportes e Lazer	Competições esportivas	50% trabalhadores	Teletrabalho /<br>Presencial restrito,<br>com atendimento integral da Nota Informativa nº 18 COE SES-RS de 13/08/2020 (+)<br>Autorização do(s) município(s) sede	Atendimento coletivo exclusivo de atletas /<br>Sem público	X	X	X	Nota Informativa nº 18 COE SES-RS de 13/08/2020
Serviços	105*	Outros Serviços	Outros Serviços - Outros	25% trabalhadores	Teletrabalho /<br>Presencial restrito	Teleatendimento /<br>Presencial restrito	X			
Serviços	105*	Outros Serviços	Reparação e manutenção de objetos e equipamentos	50% trabalhadores	Teletrabalho /<br>Presencial restrito	Teleatendimento /<br>Presencial restrito	X			
Serviços	105*	Outros Serviços	Lavanderias e similares	50% trabalhadores	Teletrabalho /<br>Presencial restrito	Presencial restrito /<br>Tele-entrega /<br>Pegue e leve	X			
Serviços	105*	Outros Serviços	Serviços de higiene pessoal (cabelereiro e barbeiro)	25% trabalhadores	Teletrabalho /<br>Presencial restrito	Atendimento individualizado,<br>por ambiente	X			
Serviços	105*	Outros Serviços	Serviços de higiene e alojamento de animais domésticos (*petshop*)	25% trabalhadores	Teletrabalho /<br>Presencial restrito	Teleatendimento /<br>Presencial restrito	X			

DISTANCIAMENTO CONTROLADO
GOV RS
NOVAS FAÇANHAS

Notas:
(*) Representam agregações de atividades 2 dígitos:
101* = 64, 65, 66
102* = 69, 70, 71, 72, 73, 74, 75
103* = 77, 78, 79, 82
104* = 90, 91, 92, 93
105* = 94, 95, 96, 99

BANDEIRA LARANJA - Serviços

// Atividade				// Critérios específicos de funcionamento (conforme bandeira)			// Protocolos obrigatório (todas as bandeiras)	// Protocolos variáveis (recomendados)		// Restrições adicionais
Grupo	CNAE (2 díg.)	Tipo	Subtipos	Teto de Operação<br>Determina o percentual máximo de trabalhadores/público externo presentes no mesmo turno, ao mesmo tempo.<br><br>Deve respeitar ao nº máximo de pessoas no espaço físico, considerando o distanciamento interpessoal mínimo obrigatório (teto de ocupação).	Modo de Operação<br>Forma de operação da atividade, respeitando ao teto de operação, ao teto de ocupação do espaço físico e aos protocolos obrigatórios (ao lado).<br><br>**Trabalhadores**	**Atendimento**	**Decreto nº 55.2540:**<br>- Máscara / EPIs,<br>- Distanciamento,<br>- Teto de ocupação,<br>- Higienização,<br>- Proteção de grupo de risco,<br>- Afastamento de casos,<br>- Cuidados com o público,<br>- Atendimento do grupos de risco<br>- Informativo visível (operação, ocupação e cuidados)	**Monitora- mento de tempera- tura**	**Testagem dos trabalha- dores**	Conteúdo completo das normas obrigatórias específicas à atividade: coronavirus.rs.gov.br/portarias-da-ses
Serviços	105*	Outros Serviços	Missas e serviços religiosos	30% público	Teletrabalho /<br>Presencial restrito /<br>Restaurantes, bares, lanchonetes e espaços coletivos de alimentação: conforme protocolo de "Restaurantes" e "Lanchonetes" e Portaria SES nº 319 /	Presencial restrito /<br>SE PERMITIDO o consumo de bebidas ou alimentos, ocupação intercalada de assentos, respeitando distanciamento mínimo de 2m entre pessoas e/ou grupos de coabitantes /<br>SE NÃO PERMITIDO o consumo de bebidas ou alimentos, ocupação intercalada de assentos, respeitando distanciamento mínimo de 1m entre pessoas e/ou grupos de coabitantes /<br>Obrigatória a utilização de máscaras /<br>Atendimento individualizado	X			
Serviços	105*	Outros Serviços	Festas, festejos e procissões religiosas ou similares, em ambiente público ou privado, aberto ou fechado	Vedada qualquer atividade que não esteja de acordo com o Modo de Operação deste subtipo.		Permitido apenas manifestações individuais ou em grupos de no máx. 10 pessoas, com uso obrigatório de máscara e distanciamento interpessoal de no mín. 1 metro.<br><br>Carreatas permitidas, com permanência das pessoas exclusivamente no interior dos veículos.<br><br>Vedada qualquer aglomeração, sujeita à fiscalização e à dispersão pelas autoridades.	X			
Serviços	105*	Outros Serviços	Funerária	100% trabalhadores	Teletrabalho /<br>Presencial restrito	Teleatendimento /<br>Presencial restrito<br>(máx. 10, se Covid-19)	X			
Serviços	105*	Outros Serviços	Organizações sindicais, patronais, empresariais e profissionais	50% trabalhadores	Teletrabalho /<br>Presencial restrito	Teleatendimento /<br>Presencial restrito	X			
Serviços	105*	Outros Serviços	Atividades administrativas dos serviços sociais autônomos	50% trabalhadores	Teletrabalho /<br>Presencial restrito	Teleatendimento /<br>Presencial restrito	X			Nas atividades-fim, observar protocolos específicos conforme medidas sanitárias segmentadas neste decreto.
Serviços	101*	Serv. Financeiros	Bancos, lotéricas e similares	75% trabalhadores	Teletrabalho /<br>Presencial restrito	Teleatendimento /<br>Presencial restrito	X			
Serviços	68	Serv. Imobiliário	Imobiliárias e similares	50% trabalhadores	Teletrabalho /<br>Presencial restrito	Teleatendimento /<br>Presencial restrito	X			
Serviços	102*	Serv. Profissionais, Científicas e Técnicas	Serviços de auditoria, consultoria, engenharia, arquitetura, publicidade e outros	50% trabalhadores	Teletrabalho /<br>Presencial restrito	Teleatendimento /<br>Presencial restrito	X			
Serviços	102*	Serv. Profissionais, Científicas e Técnicas	Serviços profissionais de advocacia e de contabilidade	75% trabalhadores	Teletrabalho /<br>Presencial restrito	Teleatendimento /<br>Presencial restrito	X			
Serviços	103*	Serv. Admin. e Auxiliares	Serv. Admin. e Auxiliares - Outros	50% trabalhadores	Teletrabalho /<br>Presencial restrito	Teleatendimento /<br>Presencial restrito	X			
Serviços	103*	Serv. Admin. e Auxiliares	Agência de turismo, passeios e excursões	50% trabalhadores	Teletrabalho /<br>Presencial restrito	Teleatendimento /<br>Presencial restrito<br>(grupos exclusivo para agências com Selo Turismo Responsável do MTur)	X	X		Selo Turismo Responsável - Ministério do Turismo
Serviços	80	Vigilância, Segurança e Investigação	Vigilância, Segurança e Investigação	75% trabalhadores	Teletrabalho /<br>Presencial restrito		X			

DISTANCIAMENTO CONTROLADO iGOV RS NOVAS FAÇANHAS

Notas:
(*) Representam agregações de atividades 2 dígitos:
101* = 64, 65, 66
102* = 69, 70, 71, 72, 73, 74, 75
103* = 77, 78, 79, 82
104* = 90, 91, 92, 93
105* = 94, 95, 96, 99

BANDEIRA LARANJA - Serviços

// Atividade				// Critérios específicos de funcionamento (conforme bandeira)			// Protocolos obrigatório (todas as bandeiras)	// Protocolos variáveis (recomendados)		// Restrições adicionais
Grupo	CNAE (2 díg.)	Tipo	Subtipos	**Teto de Operação**<br>Determina o percentual máximo de trabalhadores/público externo presentes no mesmo turno, ao mesmo tempo.<br><br>Deve respeitar ao nº máximo de pessoas no espaço físico, considerando o distanciamento interpessoal mínimo obrigatório (teto de ocupação).	**Modo de Operação**<br>Forma de operação da atividade, respeitando ao teto de operação, ao teto de ocupação do espaço físico e aos protocolos obrigatórios (ao lado).<br><br>**Trabalhadores**	**Atendimento**	**Decreto nº 55.2540:**<br>- Máscara / EPIs,<br>- Distanciamento,<br>- Teto de ocupação,<br>- Higienização,<br>- Proteção de grupo de risco,<br>- Afastamento de casos,<br>- Cuidados com o público,<br>- Atendimento do grupos de risco<br>- Informativo visível (operação, ocupação e cuidados)	**Monitora- mento de tempera- tura**	**Testagem dos trabalha- dores**	Conteúdo completo das normas obrigatórias específicas à atividade: coronavirus.rs.gov.br/portarias-da-ses
Serviços	97	Serv. Domésticos	Faxineiros, cozinheiros, motoristas, babás, jardineiros e similares	50% trabalhadores	Presencial restrito / Obrigatório uso correto da máscara por empregado(s) e empregador(es) durante a prestação do serviço, para proteção de ambos / Circulação de ar cruzada (janelas abertas)		X			
Serviços	81	Condomínios prediais, residenciais e comerciais	*Áreas comuns*	75% trabalhadores	Teletrabalho / Presencial restrito	Teleatendimento / Presencial restrito / Equipamentos, espreguiçadeiras, brinquedos infantis: distanciamento mínimo de 4m e higienização constante com álcool 70% ou solução sanitizante similar / Área de piscinas e águas, saunas, academias, quadras etc.: conforme protocolo de "Serviços de educação física (academias, centros de treinamento, estúdios e similares)", "Serviços de educação física em piscina (aberta ou fechada)" e Portaria SES nº 582 e alterações / Eventos: conforme protocolos de "Eventos sociais e de entretenimento em ambiente aberto ou fechado" e Portaria SES nº 617 / Restaurantes, lanchonetes e espaços coletivos de alimentação: conforme protocolo de "Restaurantes e Lanchonetes" e Portaria SES nº 319 /	X			Portaria SES nº 319<br><br>Portaria SES nº 582<br><br>Portaria SES nº 617
Serviços	81	Condomínios prediais, residenciais e comerciais	Serviços de Limpeza e Manutenção de eficícios e condomínios	75% trabalhadores	Teletrabalho / Presencial restrito		X			
Serviços	72	Serv. Profissionais, Científicas e Técnicas	Pesquisa científica e laboratórios (pandemia)	100% trabalhadores	Teletrabalho / Presencial restrito		X			
Serviços	82	Serv. Admin. e Auxiliares	Call-center	50% trabalhadores	Teletrabalho / Presencial restrito	Teleatendimento	X			

Notas:
(*) Representam agregações de atividades 2 dígitos:
101* = 64, 65, 66
102* = 69, 70, 71, 72, 73, 74, 75
103* = 77, 78, 79, 82
104* = 90, 91, 92, 93
105* = 94, 95, 96, 99

BANDEIRA VERMELHA - Serviços

// Atividade				// Critérios específicos de funcionamento (conforme bandeira)			// Protocolos obrigatório (todas as bandeiras)	// Protocolos variáveis (recomendados)		// Restrições adicionais
Grupo	CNAE (2 díg.)	Tipo	Subtipos	**Teto de Operação**<br>Determina o percentual máximo de trabalhadores/público presentes no mesmo turno, ao mesmo tempo.<br><br>Deve respeitar ao nº máximo de pessoas no espaço físico, considerando o distanciamento interpessoal mínimo obrigatório (teto de ocupação).	**Modo de Operação**<br>Forma de operação da atividade, respeitando ao teto de operação, ao teto de ocupação do espaço físico e aos protocolos obrigatórios (ao lado).<br><br>**Trabalhadores**	**Atendimento**	**Decreto nº 55.2540:**<br>- Máscara / EPIs,<br>- Distanciamento,<br>- Teto de ocupação,<br>- Higienização,<br>- Proteção de grupo de risco,<br>- Afastamento de casos,<br>- Cuidados com o público,<br>- Atendimento do grupos de risco<br>- Informativo visível (operação, ocupação e cuidados)	**Monitora-mento de tempera- tura**	**Testagem dos trabalha- dores**	Conteúdo completo das normas obrigatórias específicas à atividade: coronavirus.rs.gov.br/portarias-da-ses
Serviços	104*	Artes, Cultura, Esportes e Lazer	Parques Temáticos, Parques de Diversão, Parques de Aventura, Parques Aquáticos, Atrativos Turísticos e Similares - fixos ou itinerantes	Permitido exclusivamente para locais com **Selo Turismo Responsável** do MTur e em **ambiente ABERTO**, com **controle de acesso:**<br><br>50% trabalhadores<br><br>25% público	Teletrabalho / Presencial restrito / Restaurantes, bares, lanchonetes e espaços coletivos de alimentação: conforme protocolo de "Restaurantes" e "Lanchonetes" e Portaria SES nº 319 /	Teleatendimento / Presencial restrito (somentes áreas externas, com demarcação no chão de áreas de permanência distanciada de grupos - máx. 8 pessoas)	X	X		Selo Turismo Responsável - Ministério do Turismo
Serviços	104*	Artes, Cultura, Esportes e Lazer	Parques e reservas naturais, jardins botânicos e zoológicos	Permitido exclusivamente em **ambientes ABERTOS**, com **controle de acesso:**<br><br>50% trabalhadores<br><br>25% público	Teletrabalho / Presencial restrito / Restaurantes, bares, lanchonetes e espaços coletivos de alimentação: conforme protocolo de "Restaurantes" e "Lanchonetes" e Portaria SES nº 319 /	Teleatendimento / Presencial restrito (somentes áreas externas, com demarcação no chão de áreas de permanência distanciada de grupos - máx. 8 pessoas)	X	X		Selo Turismo Responsável - Ministério do Turismo

DISTANCIAMENTO CONTROLADO
GOV RS
NOVAS FAÇANHAS

Notas:
(*) Representam agregações de atividades 2 dígitos:
101* = 64, 65, 66
102* = 69, 70, 71, 72, 73, 74, 75
103* = 77, 78, 79, 82
104* = 90, 91, 92, 93
105* = 94, 95, 96, 99

BANDEIRA VERMELHA - Serviços

// Atividade				// Critérios específicos de funcionamento (conforme bandeira)			// Protocolos obrigatório (todas as bandeiras)	// Protocolos variáveis (recomendados)		// Restrições adicionais
Grupo	CNAE (2 díg.)	Tipo	Subtipos	**Teto de Operação**<br>Determina o percentual máximo de trabalhadores/público presentes no mesmo turno, ao mesmo tempo.<br><br>Deve respeitar ao nº máximo de pessoas no espaço físico, considerando o distanciamento interpessoal mínimo obrigatório (teto de ocupação).	**Modo de Operação**<br>Forma de operação da atividade, respeitando ao teto de operação, ao teto de ocupação do espaço físico e aos protocolos obrigatórios (ao lado).<br><br>**Trabalhadores**	**Atendimento**	**Decreto nº 55.2540:**<br>- Máscara / EPIs,<br>- Distanciamento,<br>- Teto de ocupação,<br>- Higienização,<br>- Proteção de grupo de risco,<br>- Afastamento de casos,<br>- Cuidados com o público,<br>- Atendimento do grupos de risco<br>- Informativo visível (operação, ocupação e cuidados)	**Monitora-mento de tempera- tura**	**Testagem dos trabalha- dores**	Conteúdo completo das normas obrigatórias específicas à atividade: coronavirus.rs.gov.br/portarias-da-ses
Serviços	104*	Artes, Cultura, Esportes e Lazer	Teatros, auditórios, casas de espetáculos, casas de show, circos e similares<br><br>(em ambiente aberto ou fechado, com público exclusivamente sentado e restrito ao período da apresentação)	**Ambiente FECHADO**: não permitido.<br><br>**Ambiente ABERTO**, com controle de acesso: permitido, SEM consumo de alimentos/bebidas, respeitando à lotação, ao distanciamento e à necessidade de autorização, conforme número total de pessoas (ver "Restrições Adicionais").<br><br>**50% de lotação**, com distanciamento de 1m	Teletrabalho /<br>Presencial restrito, exclusiamente em ambiente aberto /<br>Permitido instalação de toldo ou cobertura, desde que as laterais sejam inteiramente abertas, para plena circulação de ar /<br>Elaboração de projeto (croqui e protocolos), disponível para fiscalização e/ou autorização, quando exigido /<br>Início e término de programações não concomitantes, quando houver multissalas /<br>Intervalo mín. de 1 hora entre as apresentações com troca de público, para permitir higienização e evitar aglomerações	Presencial restrito /<br>Máscara de uso obrigatório /<br>Reforço na comunicação sonora e visual dos protocolos de higiene e distanciamento para público e colaboradores /<br>Circulação em pé somente para uso dos sanitários, com uso de máscara e fila com distanciamento demarcado /<br>Vedado interação física entre artistas e público /<br>PROIBIDO consumo de alimentos ou bebidas /<br>Distanciamento mínimo de 1m entre pessoas e/ou grupos de coabitantes OU ocupação intercalada de assentos (sim/não/não/sim), sem ocupação de assento(s) imediatamente à frente e atrás /	X			Portaria SES nº 617<br><br>Portaria SES nº 319<br><br>Decreto Estadual nº 55.240, Art. 21, § 7º e §8º<br><br>Pedido de autorização, conforme número de pessoas (trabalhadores e público) presentes ao mesmo tempo:<br>- **Até 300**: protocolos estaduais;<br>- **300 a 600**: protocolos estaduais (+) pedido de autorização do município sede, encaminhado pela organização do evento;<br>- **600 a 1.200**: protocolos estaduais (+) pedido de autorização da(s) associação(ões) de municípios da Região Covid, encaminhado pelo município sede (aprovação por no mín. 2/3 dos municípios da Região)<br>- **1.200 a 2.500**, no máx.: protocolos estaduais (+) pedido de autorização do Gabinete de Crise, encaminhado pela(s) associação(ões) de municípios da Região Covid, após aprovação dessa(s)
Serviços	104*	Artes, Cultura, Esportes e Lazer	Espetáculos tipo drive-in (cinema, shows, etc.)	50% vagas,<br><br>com distanciamento	Teletrabalho /<br>Presencial restrito /<br>Sem contato físico /	Teleatendimento /<br>Presencial restrito /<br>Público somente nos automóveis /<br>Vedada abertura de portas e circulação externa aos automóveis /<br>Circulação somente para uso dos sanitários, com uso de máscara e fila com distanciamento demarcado /<br>PROIBIDO consumo de alimentos ou bebidas /	X			
Serviços	104*	Artes, Cultura, Esportes e Lazer	Cinemas	Fechado						
Serviços	104*	Artes, Cultura, Esportes e Lazer	Museus, centros culturais e similares	50% trabalhadores<br>25% público	Teletrabalho /<br>Presencial restrito /<br>Restaurantes, bares, lanchonetes e espaços coletivos de alimentação: conforme protocolo de "Restaurantes" e "Lanchonetes" e Portaria SES nº 319 /	Teleatendimento /<br>Presencial restrito /<br>Grupos de no máximo 6 pessoas, sob agendamento	X	X		
Serviços	104*	Artes, Cultura, Esportes e Lazer	Bibliotecas, arquivos, acervos e similares	25% trabalhadores	Teletrabalho/<br>Presencial restrito	Teleatendimento /<br>Atendimento individualizado, com agendamento (consulta local ou pegue e leve)	X			

Notas:
(*) Representam agregações de atividades 2 dígitos:
101* = 64, 65, 66
102* = 69, 70, 71, 72, 73, 74, 75
103* = 77, 78, 79, 82
104* = 90, 91, 92, 93
105* = 94, 95, 96, 99

BANDEIRA VERMELHA - Serviços

// Atividade				// Critérios específicos de funcionamento (conforme bandeira)			// Protocolos obrigatório (todas as bandeiras)	// Protocolos variáveis (recomendados)		// Restrições adicionais
Grupo	CNAE (2 díg.)	Tipo	Subtipos	**Teto de Operação**<br>Determina o percentual máximo de trabalhadores/público presentes no mesmo turno, ao mesmo tempo.<br><br>Deve respeitar ao nº máximo de pessoas no espaço físico, considerando o distanciamento interpessoal mínimo obrigatório (teto de ocupação).	**Modo de Operação**<br>Forma de operação da atividade, respeitando ao teto de operação, ao teto de ocupação do espaço físico e aos protocolos obrigatórios (ao lado).<br><br>**Trabalhadores**	**Atendimento**	**Decreto nº 55.2540:**<br>- Máscara / EPIs,<br>- Distanciamento,<br>- Teto de ocupação,<br>- Higienização,<br>- Proteção de grupo de risco,<br>- Afastamento de casos,<br>- Cuidados com o público,<br>- Atendimento do grupos de risco<br>- Informativo visível (operação, ocupação e cuidados)	**Monitora- mento de tempera- tura**	**Testagem dos trabalha- dores**	Conteúdo completo das normas obrigatórias específicas à atividade: coronavirus.rs.gov.br/portarias-da-ses
Serviços	104*	Artes, Cultura, Esportes e Lazer	Ateliês (artes plásticas, restauração de obras de arte, escrita, artistas independentes e similares)	Fechado						
Serviços	104*	Artes, Cultura, Esportes e Lazer	Atividades de organizações associativas ligadas à arte e à cultura (MTG e similares)	Fechado						
Serviços	104*	Artes, Cultura, Esportes e Lazer	Convenções partidárias	10% lotação<br><br>Máx. 30 pessoas, ao mesmo tempo	Teletrabalho /<br>Presencial restrito /<br>Circulação de ar cruzada /<br>Credenciamento e check-in online	Presencial restrito /<br>Cadeiras intercalados (sim/não/não/sim) /<br>Filas intercaladas /<br>16m² por pessoa /<br>Entrada e saída escalonada por filas previamente demarcadas /<br>Material individual (canetas)	X			
Serviços	104*	Artes, Cultura, Esportes e Lazer	Feiras e Exposições corporativas e comerciais	Fechado						
Serviços	104*	Artes, Cultura, Esportes e Lazer	Seminários, congressos, convenções, simpósios e similares	Fechado						
Serviços	104*	Artes, Cultura, Esportes e Lazer	Reuniões corporativas, oficinas, treinamentos e cursos corporativos	Fechado						
Serviços	104*	Artes, Cultura, Esportes e Lazer	Eventos infantis em buffets, casas de festas ou similares<br><br>(em ambiente aberto ou fechado)	Fechado						
Serviços	104*	Artes, Cultura, Esportes e Lazer	Eventos sociais e de entretenimento em buffets, casas de festas, casas de shows, casas noturnas, bares e pubs ou similares<br><br>(em ambiente fechado, com público em pé)	Fechado						
Serviços	104*	Artes, Cultura, Esportes e Lazer	Eventos sociais e de entretenimento em ambiente aberto, com público em pé	Fechado						
Serviços	104*	Artes, Cultura, Esportes e Lazer	Demais tipos de eventos, em ambiente fechado ou aberto	Fechado						
Serviços	104*	Artes, Cultura, Esportes e Lazer	Serviços de educação física (academias, centros de treinamento, estúdios e similares)	25% trabalhadores<br><br>25% lotação	Teletrabalho /<br>Aberto somente para atividades físicas vinculada à manutenção da saúde /<br>Fechado para lazer	Presencial restrito,<br>com distanciamento, sem contato físico,<br>material individual /<br>Ocupação de 1 pessoa para cada 16m² de área útil (piscina, academia etc.) /<br>Esportes coletivos (dois ou mais atletas) exclusivo para atletas profissionais, sem público	X			Portaria SES nº 582<br><br>Decreto Estadual nº 55.240, Art. 21, §8º

Notas:
(*) Representam agregações de atividades 2 dígitos:
101* = 64, 65, 66
102* = 69, 70, 71, 72, 73, 74, 75
103* = 77, 78, 79, 82
104* = 90, 91, 92, 93
105* = 94, 95, 96, 99

BANDEIRA VERMELHA - Serviços

// Atividade				// Critérios específicos de funcionamento (conforme bandeira)			// Protocolos obrigatório (todas as bandeiras)	// Protocolos variáveis (recomendados)		// Restrições adicionais
Grupo	CNAE (2 díg.)	Tipo	Subtipos	**Teto de Operação**<br>Determina o percentual máximo de trabalhadores/público presentes no mesmo turno, ao mesmo tempo.<br><br>Deve respeitar ao nº máximo de pessoas no espaço físico, considerando o distanciamento interpessoal mínimo obrigatório (teto de ocupação).	**Modo de Operação**<br>Forma de operação da atividade, respeitando ao teto de operação, ao teto de ocupação do espaço físico e aos protocolos obrigatórios (ao lado).<br><br>**Trabalhadores**	**Atendimento**	**Decreto nº 55.2540:**<br>- Máscara / EPIs,<br>- Distanciamento,<br>- Teto de ocupação,<br>- Higienização,<br>- Proteção de grupo de risco,<br>- Afastamento de casos,<br>- Cuidados com o público,<br>- Atendimento do grupos de risco<br>- Informativo visível (operação, ocupação e cuidados)	**Monitora- mento de tempera- tura**	**Testagem dos trabalha- dores**	Conteúdo completo das normas obrigatórias específicas à atividade: coronavirus.rs.gov.br/portarias-da-ses
Serviços	104*	Artes, Cultura, Esportes e Lazer	Serviços de educação física em piscina (aberta ou fechada)	25% trabalhadores<br><br>25% lotação	Teletrabalho /<br>Piscinas abertas somente para atividades físicas vinculada à manutenção da saúde (natação, hidroginástica e fisioterapia) /<br>Fechado para lazer	Presencial restrito,<br>com distanciamento, sem contato físico,<br>material individual /<br>Ocupação de 1 pessoa para cada 16m² de área útil (piscina, academia etc.) /<br>Esportes coletivos (dois ou mais atletas) exclusivo para atletas profissionais, sem público	X			
Serviços	104*	Artes, Cultura, Esportes e Lazer	Clubes sociais, esportivos e similares	25% trabalhadores<br><br>25% lotação	Teletrabalho /<br>Aberto somente para atividades físicas /<br>Piscinas abertas somente para atividades físicas vinculadas à manutenção da saúde (natação, hidroginástica e fisioterapia) /<br>Fechado para lazer /<br>Restaurantes, bares, lanchonetes e espaços coletivos de alimentação: conforme protocolo de "Restaurantes" e "Lanchonetes" e Portaria SES nº 319 /	Presencial restrito,<br>com distanciamento, sem contato físico,<br>material individual<br>/<br>Ocupação de 1 pessoa para cada **16m²** de área útil (piscina, academia etc.) /<br><br>Esportes coletivos (dois ou mais atletas) exclusivo para atletas profissionais, sem público<br><br>Fechamento de áreas comuns, tais como espreguiçadeiras, brinquedos infantis, saunas, quadras, salões de festas, churrasqueiras compartilhadas e demais locais para eventos sociais e de entretenimento /<br>Academias e piscinas conforme protocolo de "Serviços de educação física (academias, centros de treinamento, estúdios e similares)", "Serviços de educação física em piscina (aberta ou fechada)" e Portaria SES nº 582 e alterações /<br>Atividades tradicionalistas de dança e ensaios conforme protocolo de "Ensino de Esportes, Dança e Artes Cênicas"	X			Portaria SES nº 319<br><br>Portaria SES nº 582<br><br>Portaria SES nº 617<br><br>Decreto Estadual nº 55.240, Art. 21, §8º
Serviços	104*	Artes, Cultura, Esportes e Lazer	Clubes de futebol profissional em disputa no Campeonato Gaúcho (Gauchão Ipiranga 2020), no Campeonato Brasileiro 2020 e na Copa Libertadores (Conmebol Libertadores 2020)	25% trabalhadores	Teletrabalho /<br>Presencial restrito,<br>com atendimento integral dos protocolos da FGF, da CBF, da Commembol e<br>das recomendações do Comitê Científico (Nota Resposta de 08/07/2020)	Treinos e jogos coletivos, exclusivos de atletas profissionais /<br>Sem público	X		X	Protocolos da Federação Gaúcha de Futebol (FGF),<br>Recomendações do Comitê Científico (Nota Resposta de 08/07/2020),<br>Guia Médico de Sugestões Protetivas Para o Retorno às Atividades do Futebol Brasileiro (CBF),<br>Diretriz Técnico Operacional de Retorno das Competições (CBF),<br>Protocolo de operações para o reinício das competições de clubes da Conmebol;<br>Protocolo de recomendações médicas para treinamentos, viagens e competições durante a pandemia COVID-19 da Conmebol;<br>Concentração Sanitária: disposições da Conmebol para diminuir o contágio - com risco médico aceitável - do Coronavírus (COVID-19) durante a reativação do futebol Sul-Americano.

Notas:
(*) Representam agregações de atividades 2 dígitos:
101* = 64, 65, 66
102* = 69, 70, 71, 72, 73, 74, 75
103* = 77, 78, 79, 82
104* = 90, 91, 92, 93
105* = 94, 95, 96, 99

BANDEIRA VERMELHA - Serviços

// Atividade				// Critérios específicos de funcionamento (conforme bandeira)			// Protocolos obrigatório (todas as bandeiras)	// Protocolos variáveis (recomendados)		// Restrições adicionais
Grupo	CNAE (2 díg.)	Tipo	Subtipos	**Teto de Operação**<br>Determina o percentual máximo de trabalhadores/público presentes no mesmo turno, ao mesmo tempo.<br><br>Deve respeitar ao nº máximo de pessoas no espaço físico, considerando o distanciamento interpessoal mínimo obrigatório (teto de ocupação).	**Modo de Operação**<br>Forma de operação da atividade, respeitando ao teto de operação, ao teto de ocupação do espaço físico e aos protocolos obrigatórios (ao lado).<br><br>**Trabalhadores**	**Atendimento**	**Decreto nº 55.2540:**<br>- Máscara / EPIs,<br>- Distanciamento,<br>- Teto de ocupação,<br>- Higienização,<br>- Proteção de grupo de risco,<br>- Afastamento de casos,<br>- Cuidados com o público,<br>- Atendimento do grupos de risco<br>- Informativo visível (operação, ocupação e cuidados)	**Monitora- mento de tempera- tura**	**Testagem dos trabalha- dores**	Conteúdo completo das normas obrigatórias específicas à atividade: coronavirus.rs.gov.br/portarias-da-ses
Serviços	104*	Artes, Cultura, Esportes e Lazer	Competições esportivas	50% trabalhadores<br><br>Exclusivo campeonatos esportivos chancelados por ligas estaduais e nacionais, federações e confederações nacionais e internacionais reconhecidas pelo Sistema do Desporto Nacional	Teletrabalho / Presencial restrito, com atendimento integral da Nota Informativa nº 18 COE SES-RS de 13/08/2020 (+) Autorização do(s) município(s) sede	Atendimento coletivo exclusivo de atletas profissionais / Sem público /<br><br>Vedadas competições de atletas amadores	X			
Serviços	105*	Outros Serviços	Outros Serviços - Outros	Fechado						
Serviços	105*	Outros Serviços	Reparação e manutenção de objetos e equipamentos	25% trabalhadores	Teletrabalho / Presencial restrito	Teleatendimento / Presencial restrito	X			
Serviços	105*	Outros Serviços	Lavanderias e similares	25% trabalhadores	Teletrabalho / Presencial restrito	Presencial restrito / Tele-entrega / Pegue e leve	X	X		
Serviços	105*	Outros Serviços	Serviços de higiene pessoal (cabelereiro e barbeiro)	25% trabalhadores	Teletrabalho / Presencial restrito	Atendimento individualizado, por ambiente (distanciamento de 4m entre clientes)	X			
Serviços	105*	Outros Serviços	Serviços de higiene e alojamento de animais domésticos (*petshop*)	25% trabalhadores	Teletrabalho / Presencial restrito	Teleatendimento / Atendimento individual, sob agendamento tipo Pegue e leve	X			
Serviços	105*	Outros Serviços	Missas e serviços religiosos	ou máx. 30 pessoas, ou 20% público	Teletrabalho / Presencial restrito	Presencial restrito / Proibido o consumo de alimentos e bebidas, exceto o estritamente necessário para a realização do ritual ou celebração (por ex.: eucaristia ou comunhão), recolocando a máscara imediatamente depois) / Ocupação intercalada de assentos, respeitando distanciamento mínimo de 1m entre pessoas e/ou grupos de coabitantes / Obrigatória a utilização de máscaras / Atendimento individualizado	X	X		
Serviços	105*	Outros Serviços	Festas, festejos e procissões religiosas ou similares, em ambiente púbico ou privado, aberto ou fechado	Vedada qualquer atividade que não esteja de acordo com o Modo de Operação deste subtipo.		Permitido apenas manifestações individuais ou em grupos de no máx. 10 pessoas, com uso obrigatório de máscara e distanciamento interpessoal de no mín. 1 metro.<br><br>Carreatas permitidas, com permanência das pessoas exclusivamente no interior dos veículos.<br><br>Vedada qualquer aglomeração, sujeita à fiscalização e à dispersão pelas autoridades.	X			
Serviços	105*	Outros Serviços	Funerária	100% trabalhadores	Teletrabalho / Presencial restrito	Teleatendimento / Presencial restrito (máx. 10, se Covid-19)	X			
Serviços	105*	Outros Serviços	Organizações sindicais, patronais, empresariais e profissionais	25% trabalhadores	Teletrabalho / Presencial restrito	Teleatendimento / Atendimento individual, sob agendamento	X	X		

Notas:
(*) Representam agregações de atividades 2 dígitos:
101* = 64, 65, 66
102* = 69, 70, 71, 72, 73, 74, 75
103* = 77, 78, 79, 82
104* = 90, 91, 92, 93
105* = 94, 95, 96, 99

BANDEIRA VERMELHA - Serviços

// Atividade				// Critérios específicos de funcionamento (conforme bandeira)			// Protocolos obrigatório (todas as bandeiras)	// Protocolos variáveis (recomendados)		// Restrições adicionais
Grupo	CNAE (2 díg.)	Tipo	Subtipos	**Teto de Operação**<br>Determina o percentual máximo de trabalhadores/público presentes no mesmo turno, ao mesmo tempo.<br><br>Deve respeitar ao nº máximo de pessoas no espaço físico, considerando o distanciamento interpessoal mínimo obrigatório (teto de ocupação).	**Modo de Operação**<br>Forma de operação da atividade, respeitando ao teto de operação, ao teto de ocupação do espaço físico e aos protocolos obrigatórios (ao lado).<br><br>**Trabalhadores**	**Atendimento**	**Decreto nº 55.2540:**<br>- Máscara / EPIs,<br>- Distanciamento,<br>- Teto de ocupação,<br>- Higienização,<br>- Proteção de grupo de risco,<br>- Afastamento de casos,<br>- Cuidados com o público,<br>- Atendimento do grupos de risco<br>- Informativo visível (operação, ocupação e cuidados)	**Monitora- mento de tempera- tura**	**Testagem dos trabalha- dores**	Conteúdo completo das normas obrigatórias específicas à atividade: coronavirus.rs.gov.br/portarias-da-ses
Serviços	105*	Outros Serviços	Atividades administrativas dos serviços sociais autônomos	25% trabalhadores	Teletrabalho / Presencial restrito	Teleatendimento	X	X		Nas atividades-fim, observar protocolos específicos conforme medidas sanitárias segmentadas neste decreto.
Serviços	101*	Serv. Financeiros	Bancos, lotéricas e similares	50% trabalhadores (ou normativa municipal)	Teletrabalho / Presencial restrito	Teleatendimento / Presencial restrito	X	X		
Serviços	68	Serv. Imobiliário	Imobiliárias e similares	25% trabalhadores	Teletrabalho / Presencial restrito	Teleatendimento	X	X		
Serviços	102*	Serv. Profissionais, Científicas e Técnicas	Serviços de auditoria, consultoria, engenharia, arquitetura, publicidade e outros	25% trabalhadores	Teletrabalho / Presencial restrito	Teleatendimento	X	X		
Serviços	102*	Serv. Profissionais, Científicas e Técnicas	Serviços profissionais de advocacia e de contabilidade	50% trabalhadores	Teletrabalho / Presencial restrito	Teleatendimento / Presencial restrito	X	X		
Serviços	103*	Serv. Admin. e Auxiliares	Serv. Admin. e Auxiliares - Outros	25% trabalhadores	Teletrabalho / Presencial restrito	Teleatendimento	X	X		
Serviços	103*	Serv. Admin. e Auxiliares	Agência de turismo, passeios e excursões	25% trabalhadores	Teletrabalho / Presencial restrito	Teleatendimento	X	X		Selo Turismo Responsável - Ministério do Turismo
Serviços	80	Vigilância, Segurança e Investigação	Vigilância, Segurança e Investigação	75% trabalhadores	Teletrabalho / Presencial restrito		X			
Serviços	97	Serv. Domésticos	Faxineiros, cozinheiros, motoristas, babás, jardineiros e similares	50% trabalhadores	Presencial restrito / Obrigatório uso correto da máscara por empregado(s) e empregador(es) durante a prestação do serviço, para proteção de ambos / Circulação de ar cruzada (janelas abertas)		X			
Serviços	81	Condomínios prediais, residenciais e comerciais	*Áreas comuns*	50% trabalhadores<br><br>Fechamento de áreas comuns	Teletrabalho / Presencial restrito	Teleatendimento / Presencial restrito / Fechamento de áreas comuns, tais como espreguiçadeiras, brinquedos infantis, piscinas, saunas, quadras, salões de festas, churrasqueiras compartilhadas e demais locais para eventos sociais e de entretenimento / Academias com atendimento individualizado ou coabitante, sob agendamento, com ventilação cruzada e higienização constante, conforme Portaria SES nº 582 e alterações /	X	X		Portaria SES nº 319<br><br>Portaria SES nº 582<br><br>Portaria SES nº 617
Serviços	81	Condomínios prediais, residenciais e comerciais	Serviços de Limpeza e Manutenção de eficícios e condomínios	50% trabalhadores	Teletrabalho / Presencial restrito		X	X		
Serviços	72	Serv. Profissionais, Científicas e Técnicas	Pesquisa científica e laboratórios (pandemia)	100% trabalhadores	Teletrabalho / Presencial restrito		X	X		
Serviços	82	Serv. Admin. e Auxiliares	Call-center	50% trabalhadores	Teletrabalho / Presencial restrito	Teleatendimento	X	X		

Notas:
(*) Representam agregações de atividades 2 dígitos:
101* = 64, 65, 66
102* = 69, 70, 71, 72, 73, 74, 75
103* = 77, 78, 79, 82
104* = 90, 91, 92, 93
105* = 94, 95, 96, 99

BANDEIRA PRETA - Serviços

// Atividade				// Critérios específicos de funcionamento (conforme bandeira)			// Protocolos obrigatório (todas as bandeiras)	// Protocolos variáveis (recomendados)		// Restrições adicionais
Grupo	CNAE (2 díg.)	Tipo	Subtipos	**Teto de Operação**<br>Determina o percentual máximo de trabalhadores/público presentes no mesmo turno, ao mesmo tempo.<br><br>Deve respeitar ao nº máximo de pessoas no espaço físico, considerando o distanciamento interpessoal mínimo obrigatório (teto de ocupação).	**Modo de Operação**<br>Forma de operação da atividade, respeitando ao teto de operação, ao teto de ocupação do espaço físico e aos protocolos obrigatórios (ao lado).<br><br>**Trabalhadores**	**Atendimento**	**Decreto nº 55.2540:**<br>- Máscara / EPIs,<br>- Distanciamento,<br>- Teto de ocupação,<br>- Higienização,<br>- Proteção de grupo de risco,<br>- Afastamento de casos,<br>- Cuidados com o público,<br>- Atendimento do grupos de risco<br>- Informativo visível (operação, ocupação e cuidados)	**Monitora- mento de tempera- tura**	**Testagem dos trabalha- dores**	Conteúdo completo das normas obrigatórias específicas à atividade: coronavirus.rs.gov.br/portarias-da-ses
Serviços	104*	Artes, Cultura, Esportes e Lazer	Parques Temáticos, Parques de Diversão, Parques de Aventura, Parques Aquáticos, Atrativos Turísticos e Similares - fixos ou itinerantes	25% trabalhadores	Teletrabalho /<br>Presencial restrito /<br>Restaurantes, bares, lanchonetes e espaços coletivos de alimentação: conforme protocolo de "Restaurantes" e "Lanchonetes" e Portaria SES nº 319 /	Sem atendimento ao público	X	X		Selo Turismo Responsável - Ministério do Turismo
Serviços	104*	Artes, Cultura, Esportes e Lazer	Parques e reservas naturais, jardins botânicos e zoológicos	50% trabalhadores	Teletrabalho /<br>Presencial restrito /<br>Restaurantes, bares, lanchonetes e espaços coletivos de alimentação: conforme protocolo de "Restaurantes" e "Lanchonetes" e Portaria SES nº 319 /	Sem atendimento ao público	X	X		Selo Turismo Responsável - Ministério do Turismo
Serviços	104*	Artes, Cultura, Esportes e Lazer	Teatros, auditórios, casas de espetáculos, casas de show, circos e similares<br><br>(em ambiente aberto ou fechado, com público exclusivamente <u>sentado</u> e restrito ao período da <u>apresentação</u>)	Fechado						
Serviços	104*	Artes, Cultura, Esportes e Lazer	Espetáculos tipo drive-in (cinema, shows, etc.)	Fechado						
Serviços	104*	Artes, Cultura, Esportes e Lazer	Cinemas	Fechado						
Serviços	104*	Artes, Cultura, Esportes e Lazer	Museus, centros culturais e similares	25% trabalhadores	Teletrabalho /<br>Presencial restrito	Sem atendimento ao público	X	X		Recomendações aos Museus em Tempos de Covid-19, do Instituto Brasileiro de Museus (Ibram)
Serviços	104*	Artes, Cultura, Esportes e Lazer	Bibliotecas, arquivos, acervos e similares	Fechado						
Serviços	104*	Artes, Cultura, Esportes e Lazer	Ateliês (artes plásticas, restauração de obras de arte, escrita, artistas independentes e similares)	Fechado						
Serviços	104*	Artes, Cultura, Esportes e Lazer	Atividades de organizações associativas ligadas à arte e à cultura (MTG e similares)	Fechado						
Serviços	104*	Artes, Cultura, Esportes e Lazer	Convenções partidárias	Fechado						
Serviços	104*	Artes, Cultura, Esportes e Lazer	Feiras e Exposições corporativas e comerciais	Fechado						
Serviços	104*	Artes, Cultura, Esportes e Lazer	Seminários, congressos, convenções, simpósios e similares	Fechado						
Serviços	104*	Artes, Cultura, Esportes e Lazer	Reuniões corporativas, oficinas, treinamentos e cursos corporativos	Fechado						

Notas:
(*) Representam agregações de atividades 2 dígitos:
101* = 64, 65, 66 104* = 90, 91, 92, 93
102* = 69, 70, 71, 72, 73, 74, 75 105* = 94, 95, 96, 99
103* = 77, 78, 79, 82

BANDEIRA PRETA - Serviços

// Atividade				// Critérios específicos de funcionamento (conforme bandeira)			// Protocolos obrigatório (todas as bandeiras)	// Protocolos variáveis (recomendados)		// Restrições adicionais
Grupo	CNAE (2 díg.)	Tipo	Subtipos	**Teto de Operação** Determina o percentual máximo de trabalhadores/público presentes no mesmo turno, ao mesmo tempo. Deve respeitar ao nº máximo de pessoas no espaço físico, considerando o distanciamento interpessoal mínimo obrigatório (teto de ocupação).	**Modo de Operação** Forma de operação da atividade, respeitando ao teto de operação, ao teto de ocupação do espaço físico e aos protocolos obrigatórios (ao lado). **Trabalhadores**	**Atendimento**	**Decreto nº 55.2540:** - Máscara / EPIs, - Distanciamento, - Teto de ocupação, - Higienização, - Proteção de grupo de risco, - Afastamento de casos, - Cuidados com o público, - Atendimento do grupos de risco - Informativo visível (operação, ocupação e cuidados)	**Monitora- mento de tempera- tura**	**Testagem dos trabalha- dores**	Conteúdo completo das normas obrigatórias específicas à atividade: coronavirus.rs.gov.br/portarias-da-ses
Serviços	104*	Artes, Cultura, Esportes e Lazer	Eventos infantis em buffets, casas de festas ou similares (em ambiente aberto ou fechado)	Fechado						
Serviços	104*	Artes, Cultura, Esportes e Lazer	Eventos sociais e de entretenimento em buffets, casas de festas, casas de shows, casas noturnas, bares e pubs ou similares (em ambiente fechado, com público em pé)	Fechado						
Serviços	104*	Artes, Cultura, Esportes e Lazer	Eventos sociais e de entretenimento em ambiente aberto, com público em pé	Fechado						
Serviços	104*	Artes, Cultura, Esportes e Lazer	Demais tipos de eventos, em ambiente fechado ou aberto	Fechado						
Serviços	104*	Artes, Cultura, Esportes e Lazer	Serviços de educação física (academias, centros de treinamento, estúdios e similares)	Fechado						
Serviços	104*	Artes, Cultura, Esportes e Lazer	Serviços de educação física em piscina (aberta ou fechada)	Fechado						
Serviços	104*	Artes, Cultura, Esportes e Lazer	Clubes sociais, esportivos e similares	Fechado						
Serviços	104*	Artes, Cultura, Esportes e Lazer	Clubes de futebol profissional em disputa no Campeonato Gaúcho (Gauchão Ipiranga 2020), no Campeonato Brasileiro 2020 e na Copa Libertadores (Conmebol Libertadores 2020)	Fechado						
Serviços	104*	Artes, Cultura, Esportes e Lazer	Competições esportivas	Fechado						
Serviços	105*	Outros Serviços	Outros Serviços - Outros	Fechado						
Serviços	105*	Outros Serviços	Reparação e manutenção de objetos e equipamentos	Fechado						
Serviços	105*	Outros Serviços	Lavanderias e similares	25% trabalhadores	Teletrabalho / Presencial restrito	(exclusivo) Telentrega / Pegue e Leve / Drive-thru	X	X		
Serviços	105*	Outros Serviços	Serviços de higiene pessoal (cabelereiro e barbeiro)	Fechado						

Notas:
(*) Representam agregações de atividades 2 dígitos:
101* = 64, 65, 66
102* = 69, 70, 71, 72, 73, 74, 75
103* = 77, 78, 79, 82
104* = 90, 91, 92, 93
105* = 94, 95, 96, 99

DISTANCIAMENTO CONTROLADO
.GOV.RS
NOVAS FAÇANHAS

BANDEIRA PRETA - Serviços

// Atividade				// Critérios específicos de funcionamento (conforme bandeira)			// Protocolos obrigatório (todas as bandeiras)	// Protocolos variáveis (recomendados)		// Restrições adicionais
Grupo	CNAE (2 díg.)	Tipo	Subtipos	**Teto de Operação** Determina o percentual máximo de trabalhadores/público presentes no mesmo turno, ao mesmo tempo. Deve respeitar ao nº máximo de pessoas no espaço físico, considerando o distanciamento interpessoal mínimo obrigatório (teto de ocupação).	**Modo de Operação** Forma de operação da atividade, respeitando ao teto de operação, ao teto de ocupação do espaço físico e aos protocolos obrigatórios (ao lado). **Trabalhadores**	**Atendimento**	**Decreto nº 55.2540:** - Máscara / EPIs, - Distanciamento, - Teto de ocupação, - Higienização, - Proteção de grupo de risco, - Afastamento de casos, - Cuidados com o público, - Atendimento do grupos de risco - Informativo visível (operação, ocupação e cuidados)	**Monitora- mento de tempera- tura**	**Testagem dos trabalha- dores**	Conteúdo completo das normas obrigatórias específicas à atividade: coronavirus.rs.gov.br/portarias-da-ses
Serviços	105*	Outros Serviços	Serviços de higiene e alojamento de animais domésticos (*petshop*)	Fechado						
Serviços	105*	Outros Serviços	Missas e serviços religiosos	25% trabalhadores	Teletrabalho / Presencial restrito exclusivo para captação audiovisual	Sem atendimento ao público	X	X		
Serviços	105*	Outros Serviços	Festas, festejos e procissões religiosas ou similares, em ambiente público ou privado, aberto ou fechado	Vedada qualquer atividade que não esteja de acordo com o Modo de Operação deste subtipo.		Permitido apenas manifestações individuais ou em grupos de no máx. 10 pessoas, com uso obrigatório de máscara e distanciamento interpessoal de no mín. 1 metro. Carreatas permitidas, com permanência das pessoas exclusivamente no interior dos veículos. Vedada qualquer aglomeração, sujeita à fiscalização e à dispersão pelas autoridades.	X			
Serviços	105*	Outros Serviços	Funerária	100% trabalhadores	Teletrabalho / Presencial restrito	Teleatendimento / Presencial restrito (máx. 10, se Covid-19)	X			
Serviços	105*	Outros Serviços	Organizações sindicais, patronais, empresariais e profissionais	25% trabalhadores	Teletrabalho	Teleatendimento	X	X		
Serviços	105*	Outros Serviços	Atividades administrativas dos serviços sociais autônomos	25% trabalhadores	Teletrabalho / Presencial restrito	Teleatendimento	X	X		Nas atividades-fim, observar protocolos específicos conforme medidas sanitárias segmentadas neste decreto.
Serviços	101*	Serv. Financeiros	Bancos, lotéricas e similares	50% trabalhadores	Teletrabalho / Presencial restrito	Teleatendimento / Atendimento individual, sob agendamento	X	X		
Serviços	68	Serv. Imobiliário	Imobiliárias e similares	25% trabalhadores	Teletrabalho	Teleatendimento	X	X		
Serviços	102*	Serv. Profissionais, Científicas e Técnicas	Serviços de auditoria, consultoria, engenharia, arquitetura, publicidade e outros	25% trabalhadores	Teletrabalho	Teleatendimento	X	X		
Serviços	102*	Serv. Profissionais, Científicas e Técnicas	Serviços profissionais de advocacia e de contabilidade	25% trabalhadores	Teletrabalho / Presencial restrito	Teleatendimento / Presencial restrito	X	X		
Serviços	103*	Serv. Admin. e Auxiliares	Serv. Admin. e Auxiliares - Outros	25% trabalhadores	Teletrabalho	Teleatendimento	X	X		
Serviços	103*	Serv. Admin. e Auxiliares	Agência de turismo, passeios e excursões	25% trabalhadores	Teletrabalho	Teleatendimento	X	X		Selo Turismo Responsável - Ministério do Turismo
Serviços	80	Vigilância, Segurança e Investigação	Vigilância, Segurança e Investigação	75% trabalhadores	Teletrabalho / Presencial restrito		X			
Serviços	97	Serv. Domésticos	Faxineiros, cozinheiros, motoristas, babás, jardineiros e similares	Fechado						

Notas:
(*) Representam agregações de atividades 2 dígitos:
101* = 64, 65, 66
102* = 69, 70, 71, 72, 73, 74, 75
103* = 77, 78, 79, 82
104* = 90, 91, 92, 93
105* = 94, 95, 96, 99

BANDEIRA PRETA - Serviços

// Atividade				// Critérios específicos de funcionamento (conforme bandeira)			// Protocolos obrigatório (todas as bandeiras)	// Protocolos variáveis (recomendados)		// Restrições adicionais
Grupo	CNAE (2 díg.)	Tipo	Subtipos	**Teto de Operação** Determina o percentual máximo de trabalhadores/público presentes no mesmo turno, ao mesmo tempo. Deve respeitar ao nº máximo de pessoas no espaço físico, considerando o distanciamento interpessoal mínimo obrigatório (teto de ocupação).	**Modo de Operação** Forma de operação da atividade, respeitando ao teto de operação, ao teto de ocupação do espaço físico e aos protocolos obrigatórios (ao lado). **Trabalhadores**	**Atendimento**	**Decreto nº 55.2540:** - Máscara / EPIs, - Distanciamento, - Teto de ocupação, - Higienização, - Proteção de grupo de risco, - Afastamento de casos, - Cuidados com o público, - Atendimento do grupos de risco - Informativo visível (operação, ocupação e cuidados)	**Monitora- mento de tempera- tura**	**Testagem dos trabalha- dores**	Conteúdo completo das normas obrigatórias específicas à atividade: coronavirus.rs.gov.br/portarias-da-ses
Serviços	81	Condomínios prediais, residenciais e comerciais	*Áreas comuns*	50% trabalhadores<br>Fechamento de áreas comuns	Teletrabalho / Presencial restrito	Teleatendimento / Presencial restrito / Fechamento de áreas comuns, tais como espreguiçadeiras, brinquedos infantis, piscinas, saunas, quadras, salões de festas, churrasqueiras compartilhadas e demais locais para eventos sociais e de entretenimento / Academias com atendimento individualizado ou coabitante, sob agendamento, com ventilação cruzada e higienização constante, conforme Portaria SES nº 582 e alterações /	X	X		Portaria SES nº 582
Serviços	81	Condomínios prediais, residenciais e comerciais	Serviços de Limpeza e Manutenção de eficícios e condomínios	50% trabalhadores	Teletrabalho / Presencial restrito		X	X		
Serviços	72	Serv. Profissionais, Científicas e Técnicas	Pesquisa científica e laboratórios (pandemia)	100% trabalhadores	Teletrabalho / Presencial restrito		X	X		
Serviços	82	Serv. Admin. e Auxiliares	Call-center	25% trabalhadores	Teletrabalho / Presencial restrito	Teleatendimento	X	X		

Notas:
(*) Representam agregações de atividades 2 dígitos:
101* = 64, 65, 66
102* = 69, 70, 71, 72, 73, 74, 75
103* = 77, 78, 79, 82
104* = 90, 91, 92, 93
105* = 94, 95, 96, 99

MODELO DE DISTANCIAMENTO
CONTROLADO DO RS

DISTANCIAMENTO
CONTROLADO

Serviços de Informação e Comunicação

Comitê de DADOS
COVID-19 CORONAVÍRUS

GOV RS
NOVAS FAÇANHAS
NO PLANEJAMENTO, GOVERNANÇA E GESTÃO NA SAÚDE

BANDEIRA AMARELA - Serviços de Informação e Comunicação

// Atividade				// Critérios específicos de funcionamento (conforme bandeira)			// Protocolos obrigatório (todas as bandeiras)	// Protocolos variáveis (recomendados)		// Restrições adicionais
Grupo	CNAE (2 díg.)	Tipo	Subtipos	Teto de Operação (percentual máx. de trabalhadores presentes no turno, ao mesmo tempo, respeitando o teto de ocupação do espaço físico - máx. pessoas)	Modo de Operação (forma de operação, respeitando o teto de operação e o teto de ocupação do espaço físico - máx. pessoas) Trabalhadores	Atendimento	Informativo visível (operação e ocupação) Máscara / EPIs, Distanciamento, Teto de ocupação, Higienização, Proteção de grupo de risco, Afastamento de casos, Cuidados no atendimento ao	Monitora- mento de tempera- tura	Testagem dos trabalha- dores	Normas obrigatórias específicas à atividade https://coronavirus.rs.gov.br/portarias-da-ses
Serviços de Informação e Comunicação	58	Edição e Edição Integrada à Impressão		75% trabalhadores	Teletrabalho / Presencial restrito		X			
Serviços de Informação e Comunicação	59	Produção de Vídeos e Programas de Televisão		75% trabalhadores	Teletrabalho / Presencial restrito		X			
Serviços de Informação e Comunicação	60	Atividades de Rádio e de Televisão		75% trabalhadores	Teletrabalho / Presencial restrito		X			
Serviços de Informação e Comunicação	61	Telecomunicaçõe s		100% trabalhadores	Teletrabalho / Presencial restrito	Teleatendimento / Presencial restrito	X			
Serviços de Informação e Comunicação	62	Serviços de TI		100% trabalhadores	Teletrabalho / Presencial restrito	Teleatendimento / Presencial restrito	X			
Serviços de Informação e Comunicação	63	Prestação de Serviços de Informação		100% trabalhadores	Teletrabalho / Presencial restrito	Teleatendimento	X			

DISTANCIAMENTO CONTROLADO
GOV RS
NOVAS FAÇANHAS

BANDEIRA LARANJA - Serviços de Informação e Comunicação

// Atividade				// Critérios específicos de funcionamento (conforme bandeira)			// Protocolos obrigatório (todas as bandeiras)	// Protocolos variáveis (recomendados)		// Restrições adicionais
Grupo	CNAE (2 díg.)	Tipo	Subtipos	Teto de Operação (percentual máx. de trabalhadores presentes no turno, ao mesmo tempo, respeitando o teto de ocupação do espaço físico - máx. pessoas)	Modo de Operação (forma de operação, respeitando o teto de operação e o teto de ocupação do espaço físico - máx. pessoas) Trabalhadores	Atendimento	Informativo visível (operação e ocupação) Máscara / EPIs, Distanciamento, Teto de ocupação, Higienização, Proteção de grupo de risco, Afastamento de casos, Cuidados no atendimento ao	Monitora- mento de tempera- tura	Testagem dos trabalha- dores	Normas obrigatórias específicas à atividade https://coronavirus.rs.gov.br/portarias-da-ses
Serviços de Informação e Comunicação	58	Edição e Edição Integrada à Impressão		75% trabalhadores	Teletrabalho / Presencial restrito		X			
Serviços de Informação e Comunicação	59	Produção de Vídeos e Programas de Televisão		75% trabalhadores	Teletrabalho / Presencial restrito		X			
Serviços de Informação e Comunicação	60	Atividades de Rádio e de Televisão		75% trabalhadores	Teletrabalho / Presencial restrito		X			
Serviços de Informação e Comunicação	61	Telecomunicaçõe s		100% trabalhadores	Teletrabalho / Presencial restrito	Teleatendimento / Presencial restrito	X			
Serviços de Informação e Comunicação	62	Serviços de TI		100% trabalhadores	Teletrabalho / Presencial restrito	Teleatendimento / Presencial restrito	X			
Serviços de Informação e Comunicação	63	Prestação de Serviços de Informação		100% trabalhadores	Teletrabalho / Presencial restrito	Teleatendimento	X			

DISTANCIAMENTO CONTROLADO
GOV RS
NOVAS FAÇANHAS

BANDEIRA VERMELHA - Serviços de Informação e Comunicação

// Atividade				// Critérios específicos de funcionamento (conforme bandeira)			// Protocolos obrigatório (todas as bandeiras)	// Protocolos variáveis (recomendados)		// Restrições adicionais
Grupo	CNAE (2 díg.)	Tipo	Subtipos	Teto de Operação (percentual máx. de trabalhadores presentes no turno, ao mesmo tempo, respeitando o teto de ocupação do espaço físico - máx. pessoas)	Modo de Operação (forma de operação, respeitando o teto de operação e o teto de ocupação do espaço físico - máx. pessoas) Trabalhadores	Atendimento	Informativo visível (operação e ocupação) Máscara / EPIs, Distanciamento, Teto de ocupação, Higienização, Proteção de grupo de risco, Afastamento de casos, Cuidados no atendimento ao público, Atendimento do grupos de risco	Monitora- mento de tempera- tura	Testagem dos trabalha- dores	Normas obrigatórias específicas à atividade https://coronavirus.rs.gov.br/portarias-da-ses
Serviços de Informação e Comunicação	58	Edição e Edição Integrada à Impressão		50% trabalhadores	Teletrabalho / Presencial restrito		X	X		
Serviços de Informação e Comunicação	59	Produção de Vídeos e Programas de Televisão		50% trabalhadores	Teletrabalho / Presencial restrito		X	X		
Serviços de Informação e Comunicação	60	Atividades de Rádio e de Televisão		75% trabalhadores	Teletrabalho / Presencial restrito		X	X		
Serviços de Informação e Comunicação	61	Telecomunicações		100% trabalhadores	Teletrabalho / Presencial restrito	Teleatendimento / Presencial restrito	X	X		
Serviços de Informação e Comunicação	62	Serviços de TI		100% trabalhadores	Teletrabalho / Presencial restrito	Teleatendimento / Presencial restrito	X	X		
Serviços de Informação e Comunicação	63	Prestação de Serviços de Informação		100% trabalhadores	Teletrabalho / Presencial restrito	Teleatendimento	X	X		

DISTANCIAMENTO CONTROLADO
GOV RS
NOVAS FAÇANHAS

BANDEIRA PRETA - Serviços de Informação e Comunicação

// Atividade				// Critérios específicos de funcionamento (conforme bandeira)			// Protocolos obrigatório (todas as bandeiras)	// Protocolos variáveis (recomendados)		// Restrições adicionais
Grupo	CNAE (2 díg.)	Tipo	Subtipos	Teto de Operação (percentual máx. de trabalhadores presentes no turno, ao mesmo tempo, respeitando o teto de ocupação do espaço físico - máx. pessoas)	Modo de Operação (forma de operação, respeitando o teto de operação e o teto de ocupação do espaço físico - máx. pessoas)<br>Trabalhadores	Atendimento	Informativo visível (operação e ocupação)<br>Máscara / EPIs,<br>Distanciamento,<br>Teto de ocupação,<br>Higienização,<br>Proteção de grupo de risco,<br>Afastamento de casos,<br>Cuidados no atendimento ao público,<br>Atendimento do grupos de risco	Monitora- mento de tempera- tura	Testagem dos trabalha- dores	Normas obrigatórias específicas à atividade https://coronavirus.rs.gov.br/portarias-da-ses
Serviços de Informação e Comunicação	58	Edição e Edição Integrada à Impressão		50% trabalhadores	Teletrabalho / Presencial restrito		X	X		
Serviços de Informação e Comunicação	59	Produção de Vídeos e Programas de Televisão		50% trabalhadores	Teletrabalho / Presencial restrito		X	X		
Serviços de Informação e Comunicação	60	Atividades de Rádio e de Televisão		75% trabalhadores	Teletrabalho / Presencial restrito		X	X		
Serviços de Informação e Comunicação	61	Telecomunicações		100% trabalhadores	Teletrabalho / Presencial restrito	Teleatendimento / Presencial restrito	X	X		
Serviços de Informação e Comunicação	62	Serviços de TI		100% trabalhadores	Teletrabalho / Presencial restrito	Teleatendimento / Presencial restrito	X	X		
Serviços de Informação e Comunicação	63	Prestação de Serviços de Informação		100% trabalhadores	Teletrabalho / Presencial restrito	Teleatendimento	X	X		

DISTANCIAMENTO CONTROLADO
RS
NOVAS FAÇANHAS

MODELO DE DISTANCIAMENTO
CONTROLADO DO RS

Serviços de Utilidade Pública

Comitê de DADOS
COVID-19 CORONAVÍRUS

GOV RS
NOVAS FAÇANHAS
NO PLANEJAMENTO, GOVERNANÇA E GESTÃO NA SAÚDE

BANDEIRA AMARELA - Serviços de Utilidade Pública

// Atividade				// Critérios específicos de funcionamento (conforme bandeira)			// Protocolos obrigatório (todas as bandeiras)	// Protocolos variáveis (recomendados)		// Restrições adicionais
Grupo	CNAE (2 díg.)	Tipo	Subtipos	Teto de Operação (percentual máx. de trabalhadores presentes no turno, ao mesmo tempo, respeitando o teto de ocupação do espaço físico - máx. pessoas)	Modo de Operação (forma de operação, respeitando o teto de operação e o teto de ocupação do espaço físico - máx. pessoas) Trabalhadores	Atendimento	Informativo visível (operação e ocupação) Máscara / EPIs, Distanciamento, Teto de ocupação, Higienização, Proteção de grupo de risco, Afastamento de casos, Cuidados no atendimento ao	Monitora- mento de tempera- tura	Testagem dos trabalha- dores	Normas obrigatórias específicas à atividade https://coronavirus.rs.gov.br/portarias-da-ses
Serviços de Utilidade Pública	35	Eletricidade, Gás e Outras Utilidades		100% trabalhadores	Teletrabalho / Presencial restrito	Teleatendimento / Presencial restrito	X			
Serviços de Utilidade Pública	36	Captação, Tratamento e Distribuição De Água		100% trabalhadores	Teletrabalho / Presencial restrito	Teleatendimento / Presencial restrito	X			
Serviços de Utilidade Pública	37	Esgoto e Atividades Relacionadas		100% trabalhadores	Teletrabalho / Presencial restrito	Teleatendimento / Presencial restrito	X			
Serviços de Utilidade Pública	38	Coleta, Tratamento e Disposição de Resíduos		100% trabalhadores	Teletrabalho / Presencial restrito	Teleatendimento / Presencial restrito	X			
Serviços de Utilidade Pública	39	Descontaminação e Gestão De Resíduos		100% trabalhadores	Teletrabalho / Presencial restrito	Teleatendimento / Presencial restrito	X			

DISTANCIAMENTO CONTROLADO
GOV RS NOVAS FAÇANHAS

BANDEIRA LARANJA - Serviços de Utilidade Pública

// Atividade				// Critérios específicos de funcionamento (conforme bandeira)			// Protocolos obrigatório (todas as bandeiras)	// Protocolos variáveis (recomendados)		// Restrições adicionais
Grupo	CNAE (2 díg.)	Tipo	Subtipos	Teto de Operação (percentual máx. de trabalhadores presentes no turno, ao mesmo tempo, respeitando o teto de ocupação do espaço físico - máx. pessoas)	Modo de Operação (forma de operação, respeitando o teto de operação e o teto de ocupação do espaço físico - máx. pessoas) Trabalhadores	Atendimento	Informativo visível (operação e ocupação) Máscara / EPIs, Distanciamento, Teto de ocupação, Higienização, Proteção de grupo de risco, Afastamento de casos, Cuidados no atendimento ao	Monitora- mento de tempera- tura	Testagem dos trabalha- dores	Normas obrigatórias específicas à atividade https://coronavirus.rs.gov.br/portarias-da-ses
Serviços de Utilidade Pública	35	Eletricidade, Gás e Outras Utilidades		100% trabalhadores	Teletrabalho / Presencial restrito	Teleatendimento / Presencial restrito	X			
Serviços de Utilidade Pública	36	Captação, Tratamento e Distribuição De Água		100% trabalhadores	Teletrabalho / Presencial restrito	Teleatendimento / Presencial restrito	X			
Serviços de Utilidade Pública	37	Esgoto e Atividades Relacionadas		100% trabalhadores	Teletrabalho / Presencial restrito	Teleatendimento / Presencial restrito	X			
Serviços de Utilidade Pública	38	Coleta, Tratamento e Disposição de Resíduos		100% trabalhadores	Teletrabalho / Presencial restrito	Teleatendimento / Presencial restrito	X			
Serviços de Utilidade Pública	39	Descontaminação e Gestão De Resíduos		100% trabalhadores	Teletrabalho / Presencial restrito	Teleatendimento / Presencial restrito	X			

DISTANCIAMENTO CONTROLADO
GOV RS
NOVAS FAÇANHAS

BANDEIRA VERMELHA - Serviços de Utilidade Pública

// Atividade				// Critérios específicos de funcionamento (conforme bandeira)			// Protocolos obrigatório (todas as bandeiras)	// Protocolos variáveis (recomendados)		// Restrições adicionais
Grupo	CNAE (2 díg.)	Tipo	Subtipos	Teto de Operação (percentual máx. de trabalhadores presentes no turno, ao mesmo tempo, respeitando o teto de ocupação do espaço físico - máx. pessoas)	Modo de Operação (forma de operação, respeitando o teto de operação e o teto de ocupação do espaço físico - máx. pessoas)<br>Trabalhadores	Atendimento	Informativo visível (operação e ocupação) Máscara / EPIs, Distanciamento, Teto de ocupação, Higienização, Proteção de grupo de risco, Afastamento de casos, Cuidados no atendimento ao público, Atendimento do grupos de risco	Monitora- mento de tempera- tura	Testagem dos trabalha- dores	Normas obrigatórias específicas à atividade https://coronavirus.rs.gov.br/portarias-da-ses
Serviços de Utilidade Pública	35	Eletricidade, Gás e Outras Utilidades		100% trabalhadores	Teletrabalho / Presencial restrito	Teleatendimento / Presencial restrito	X	X		
Serviços de Utilidade Pública	36	Captação, Tratamento e Distribuição De Água		100% trabalhadores	Teletrabalho / Presencial restrito	Teleatendimento / Presencial restrito	X	X		
Serviços de Utilidade Pública	37	Esgoto e Atividades Relacionadas		100% trabalhadores	Teletrabalho / Presencial restrito	Teleatendimento / Presencial restrito	X	X		
Serviços de Utilidade Pública	38	Coleta, Tratamento e Disposição de Resíduos		100% trabalhadores	Teletrabalho / Presencial restrito	Teleatendimento / Presencial restrito	X	X		
Serviços de Utilidade Pública	39	Descontaminação e Gestão De Resíduos		100% trabalhadores	Teletrabalho / Presencial restrito	Teleatendimento / Presencial restrito	X	X		

DISTANCIAMENTO CONTROLADO
GOV RS
NOVAS FAÇANHAS

BANDEIRA PRETA - Serviços de Utilidade Pública

// Atividade				// Critérios específicos de funcionamento (conforme bandeira)			// Protocolos obrigatório (todas as bandeiras)	// Protocolos variáveis (recomendados)		// Restrições adicionais
Grupo	CNAE (2 díg.)	Tipo	Subtipos	Teto de Operação (percentual máx. de trabalhadores presentes no turno, ao mesmo tempo, respeitando o teto de ocupação do espaço físico - máx. pessoas)	Modo de Operação (forma de operação, respeitando o teto de operação e o teto de ocupação do espaço físico - máx. pessoas) Trabalhadores	Atendimento	Informativo visível (operação e ocupação) Máscara / EPIs, Distanciamento, Teto de ocupação, Higienização, Proteção de grupo de risco, Afastamento de casos, Cuidados no atendimento ao público, Atendimento do grupos de risco	Monitora- mento de tempera- tura	Testagem dos trabalha- dores	Normas obrigatórias específicas à atividade https://coronavirus.rs.gov.br/portarias-da-ses
Serviços de Utilidade Pública	35	Eletricidade, Gás e Outras Utilidades		100% trabalhadores	Teletrabalho / Presencial restrito	Teleatendimento / Presencial restrito	X	X		
Serviços de Utilidade Pública	36	Captação, Tratamento e Distribuição De Água		100% trabalhadores	Teletrabalho / Presencial restrito	Teleatendimento / Presencial restrito	X	X		
Serviços de Utilidade Pública	37	Esgoto e Atividades Relacionadas		100% trabalhadores	Teletrabalho / Presencial restrito	Teleatendimento / Presencial restrito	X	X		
Serviços de Utilidade Pública	38	Coleta, Tratamento e Disposição de Resíduos		100% trabalhadores	Teletrabalho / Presencial restrito	Teleatendimento / Presencial restrito	X	X		
Serviços de Utilidade Pública	39	Descontaminação e Gestão De Resíduos		100% trabalhadores	Teletrabalho / Presencial restrito	Teleatendimento / Presencial restrito	X	X		

DISTANCIAMENTO CONTROLADO
.GOV RS
NOVAS FAÇANHAS

MODELO DE DISTANCIAMENTO
CONTROLADO DO RS

DISTANCIAMENTO
CONTROLADO
GOV RS
NOVAS FAÇANHAS

Transporte

BANDEIRA AMARELA - Transportes

// Atividade				// Critérios específicos de funcionamento (conforme bandeira)			// Protocolos obrigatório (todas as bandeiras)	// Protocolos variáveis (recomendados)		// Restrições adicionais
Grupo	CNAE (2 díg.)	Tipo	Subtipos	**Teto de Operação** Determina o percentual máximo de trabalhadores/público externo presentes no mesmo turno, ao mesmo tempo. Deve respeitar ao nº máximo de pessoas no espaço físico, considerando o distanciamento interpessoal mínimo obrigatório (teto de ocupação).	**Modo de Operação** Forma de operação da atividade, respeitando ao teto de operação, ao teto de ocupação do espaço físico e aos protocolos obrigatórios (ao lado). Trabalhadores	Atendimento	**Decreto nº 55.2540:** - Máscara / EPIs, - Distanciamento, - Teto de ocupação, - Higienização, - Proteção de grupo de risco, - Afastamento de casos, - Cuidados com o público, - Atendimento do grupos de risco - Informativo visível (operação, ocupação e cuidados)	**Monitora- mento de tempera- tura**	**Testagem dos trabalha- dores**	Conteúdo completo das normas obrigatórias específicas à atividade: coronavirus.rs.gov.br/portarias-da-ses
Transporte	49	Transporte terrestre	Transporte rodoviário fretado de passageiros	100% assentos	Teletrabalho / Presencial restrito / Ventilação cruzada (janelas abertas) ou sistema de renovação de ar	Teleatendimento / Presencial restrito	X	X		Decreto nº 55.240, Subseção II
Transporte	49	Transporte terrestre	Transporte rodoviário de carga	100% trabalhadores	Teletrabalho / Presencial restrito / Ventilação cruzada (janelas abertas) ou sistema de renovação de ar	Teleatendimento / Presencial restrito	X			
Transporte	49	Transporte terrestre	Transporte coletivo de passageiros (municipal)	60% capacidade total do veículo (ou normativa municipal)	Teletrabalho / Presencial restrito / Ventilação cruzada (janelas e/ou alçapão abertos) ou sistema de renovacão de ar	Teleatendimento / Presencial restrito	X			Decreto nº 55.240, Subseção II
Transporte	49	Transporte terrestre	Transporte coletivo de passageiros (metropolitano tipo Executivo/Seletivo)	100% assentos	Teletrabalho / Presencial restrito / Ventilação cruzada (janelas e/ou alçapão abertos) ou sistema de renovação de ar	Teleatendimento / Presencial restrito	X			Decreto nº 55.240, Subseção II
Transporte	49	Transporte terrestre	Transporte coletivo de passageiros (metropolitano tipo Comum)	70% capacidade total do veículo	Teletrabalho / Presencial restrito / Ventilação cruzada (janelas e/ou alçapão abertos) ou sistema de renovação de ar	Teleatendimento / Presencial restrito	X			Decreto nº 55.240, Subseção II
Transporte	49	Transporte terrestre	Transporte rodoviário de passageiros (intermunicipal, tipo Comum, Semidireto, Direto, Executivo ou Seletivo)	100% assentos	Teletrabalho / Presencial restrito / Ventilação cruzada (janelas e/ou alçapão abertos) ou sistema de renovação de ar (NBR 15570)	Teleatendimento / Presencial restrito	X	X		Decreto nº 55.240, Subseção II<br>NBR 15570<br>Resolução Nº 5.917, de 24 de novembro de 2020, da ANTT
Transporte	49	Transporte terrestre	Transporte rodoviário de passageiros (interestadual)	50% assentos (janela) 25% coabitantes (corredor)	Teletrabalho / Presencial restrito / Ventilação cruzada (janelas e/ou alçapão abertos) ou sistema de renovação de ar (NBR 15570)	Teleatendimento / Presencial restrito	X	X		Decreto nº 55.240, Subseção II<br>NBR 15570<br>Resolução Nº 5.917, de 24 de novembro de 2020, da ANTT
Transporte	49	Transporte terrestre	Transporte ferroviário de passageiros (metropolitano)	50% capacidade total do vagão	Teletrabalho / Presencial restrito / Ventilação cruzada (janelas e/ou alçapão abertos) ou sistema de renovacão de ar	Teleatendimento / Presencial restrito	X	X		Decreto nº 55.240, Subseção II
Transporte	50	Transporte aquaviário	Transporte aquaviário de carga	100% trabalhadores	Teletrabalho / Presencial restrito / Ventilação cruzada (janelas e/ou alçapão abertos) ou sistema de renovacão de ar	Teleatendimento / Presencial restrito	X			
Transporte	50	Transporte aquaviário	Transporte aquaviário de passageiros	100% assentos	Teletrabalho / Presencial restrito / Ventilação cruzada (janelas e/ou alçapão abertos) ou sistema de renovacão de ar	Teleatendimento / Presencial restrito	X			Decreto nº 55.240, Subseção II
Transporte	51	Transporte aéreo	Aeroclubes e aeródromos	50% trabalhadores	Teletrabalho / Presencial restrito (exclusivo para emergência Covid-19)	Teleatendimento / Presencial restrito	X			

BANDEIRA AMARELA - Transportes

// Atividade				// Critérios específicos de funcionamento (conforme bandeira)			// Protocolos obrigatório (todas as bandeiras)	// Protocolos variáveis (recomendados)		// Restrições adicionais
Grupo	CNAE (2 díg.)	Tipo	Subtipos	**Teto de Operação**<br>Determina o percentual máximo de trabalhadores/público externo presentes no mesmo turno, ao mesmo tempo.<br><br>Deve respeitar ao nº máximo de pessoas no espaço físico, considerando o distanciamento interpessoal mínimo obrigatório (teto de ocupação).	**Modo de Operação**<br>Forma de operação da atividade, respeitando ao teto de operação, ao teto de ocupação do espaço físico e aos protocolos obrigatórios (ao lado).<br><br>**Trabalhadores**	**Atendimento**	**Decreto nº 55.2540:**<br>- Máscara / EPIs,<br>- Distanciamento,<br>- Teto de ocupação,<br>- Higienização,<br>- Proteção de grupo de risco,<br>- Afastamento de casos,<br>- Cuidados com o público,<br>- Atendimento do grupos de risco<br>- Informativo visível (operação, ocupação e cuidados)	**Monitora- mento de tempera- tura**	**Testagem dos trabalha- dores**	Conteúdo completo das normas obrigatórias específicas à atividade: coronavirus.rs.gov.br/portarias-da-ses
Transporte	52	Armazenamento de Transporte	Armazenamento, carga e descarga	100% trabalhadores	Teletrabalho / Presencial restrito	Teleatendimento / Presencial restrito	X			
Transporte	52	Armazenamento de Transporte	Estacionamentos	100% trabalhadores	Teletrabalho / Presencial restrito	Teleatendimento / Presencial restrito	X			
Transporte	53	Correios	Atividades de correios, serviços postais e similares	75% trabalhadores	Teletrabalho / Presencial restrito	Teleatendimento / Presencial restrito	X			

DISTANCIAMENTO CONTROLADO

BANDEIRA LARANJA - Transportes

// Atividade				// Critérios específicos de funcionamento (conforme bandeira)			// Protocolos obrigatório (todas as bandeiras)	// Protocolos variáveis (recomendados)		// Restrições adicionais
Grupo	CNAE (2 díg.)	Tipo	Subtipos	**Teto de Operação**<br>Determina o percentual máximo de trabalhadores/público externo presentes no mesmo turno, ao mesmo tempo.<br><br>Deve respeitar ao nº máximo de pessoas no espaço físico, considerando o distanciamento interpessoal mínimo obrigatório (teto de ocupação).	**Modo de Operação**<br>Forma de operação da atividade, respeitando ao teto de operação, ao teto de ocupação do espaço físico e aos protocolos obrigatórios (ao lado).<br><br>**Trabalhadores**	**Atendimento**	**Decreto nº 55.2540:**<br>- Máscara / EPIs,<br>- Distanciamento,<br>- Teto de ocupação,<br>- Higienização,<br>- Proteção de grupo de risco,<br>- Afastamento de casos,<br>- Cuidados com o público,<br>- Atendimento do grupos de risco<br>- Informativo visível (operação, ocupação e cuidados)	**Monitora- mento de tempera- tura**	**Testagem dos trabalha- dores**	Conteúdo completo das normas obrigatórias específicas à atividade: coronavirus.rs.gov.br/portarias-da-ses
Transporte	49	Transporte terrestre	Transporte rodoviário fretado de passageiros	100% assentos	Teletrabalho / Presencial restrito / Ventilação cruzada (janelas abertas) ou sistema de renovação de ar	Teleatendimento / Presencial restrito	X	X		Decreto nº 55.240, Subseção II
Transporte	49	Transporte terrestre	Transporte rodoviário de carga	100% trabalhadores	Teletrabalho / Presencial restrito / Ventilação cruzada (janelas abertas) ou sistema de renovação de ar	Teleatendimento / Presencial restrito	X			
Transporte	49	Transporte terrestre	Transporte coletivo de passageiros (municipal)	60% capacidade total do veículo (ou normativa municipal)	Teletrabalho / Presencial restrito / Ventilação cruzada (janelas e/ou alçapão abertos) ou sistema de renovação de ar	Teleatendimento / Presencial restrito	X			Decreto nº 55.240, Subseção II
Transporte	49	Transporte terrestre	Transporte coletivo de passageiros (metropolitano tipo Executivo/Seletivo)	100% assentos	Teletrabalho / Presencial restrito / Ventilação cruzada (janelas e/ou alçapão abertos) ou sistema de renovação de ar	Teleatendimento / Presencial restrito	X			Decreto nº 55.240, Subseção II
Transporte	49	Transporte terrestre	Transporte coletivo de passageiros (metropolitano tipo Comum)	70% capacidade total do veículo	Teletrabalho / Presencial restrito / Ventilação cruzada (janelas e/ou alçapão abertos) ou sistema de renovação de ar	Teleatendimento / Presencial restrito	X			Decreto nº 55.240, Subseção II
Transporte	49	Transporte terrestre	Transporte rodoviário de passageiros (intermunicipal, tipo Comum, Semidireto, Direto, Executivo ou Seletivo)	100% assentos	Teletrabalho / Presencial restrito / Ventilação cruzada (janelas e/ou alçapão abertos) ou sistema de renovação de ar (NBR 15570)	Teleatendimento / Presencial restrito	X	X		Decreto nº 55.240, Subseção II<br><br>NBR 15570<br><br>Resolução Nº 5.917, de 24 de novembro de 2020, da ANTT
Transporte	49	Transporte terrestre	Transporte rodoviário de passageiros (interestadual)	50% assentos (janela)<br>25% coabitantes (corredor)	Teletrabalho / Presencial restrito / Ventilação cruzada (janelas e/ou alçapão abertos) ou sistema de renovação de ar (NBR 15570)	Teleatendimento / Presencial restrito	X	X		Decreto nº 55.240, Subseção II<br><br>NBR 15570<br><br>Resolução Nº 5.917, de 24 de novembro de 2020, da ANTT
Transporte	49	Transporte terrestre	Transporte ferroviário de passageiros (metropolitano)	50% capacidade total do vagão	Teletrabalho / Presencial restrito / Ventilação cruzada (janelas e/ou alçapão abertos) ou sistema de renovação de ar	Teleatendimento / Presencial restrito	X	X		Decreto nº 55.240, Subseção II
Transporte	50	Transporte aquaviário	Transporte aquaviário de carga	100% trabalhadores	Teletrabalho / Presencial restrito / Ventilação cruzada (janelas e/ou alçapão abertos) ou sistema de renovação de ar	Teleatendimento / Presencial restrito	X			
Transporte	50	Transporte aquaviário	Transporte aquaviário de passageiros	75% assentos	Teletrabalho / Presencial restrito / Ventilação cruzada (janelas e/ou alçapão abertos) ou sistema de renovação de ar	Teleatendimento / Presencial restrito	X			Decreto nº 55.240, Subseção II
Transporte	51	Transporte aéreo	Aeroclubes e aeródromos	50% trabalhadores	Teletrabalho / Presencial restrito (exclusivo para emergência Covid-19)	Teleatendimento / Presencial restrito	X			

BANDEIRA LARANJA - Transportes

// Atividade				// Critérios específicos de funcionamento (conforme bandeira)			// Protocolos obrigatório (todas as bandeiras)	// Protocolos variáveis (recomendados)		// Restrições adicionais
Grupo	CNAE (2 díg.)	Tipo	Subtipos	**Teto de Operação** Determina o percentual máximo de trabalhadores/público externo presentes no mesmo turno, ao mesmo tempo. Deve respeitar ao nº máximo de pessoas no espaço físico, considerando o distanciamento interpessoal mínimo obrigatório (teto de ocupação).	**Modo de Operação** Forma de operação da atividade, respeitando ao teto de operação, ao teto de ocupação do espaço físico e aos protocolos obrigatórios (ao lado). Trabalhadores	Atendimento	**Decreto nº 55.2540:** - Máscara / EPIs, - Distanciamento, - Teto de ocupação, - Higienização, - Proteção de grupo de risco, - Afastamento de casos, - Cuidados com o público, - Atendimento do grupos de risco - Informativo visível (operação, ocupação e cuidados)	**Monitora- mento de tempera- tura**	**Testagem dos trabalha- dores**	Conteúdo completo das normas obrigatórias específicas à atividade: coronavirus.rs.gov.br/portarias-da-ses
Transporte	52	Armazenamento de Transporte	Armazenamento, carga e descarga	100% trabalhadores	Teletrabalho / Presencial restrito	Teleatendimento / Presencial restrito	X			
Transporte	52	Armazenamento de Transporte	Estacionamentos	100% trabalhadores	Teletrabalho / Presencial restrito	Teleatendimento / Presencial restrito	X			
Transporte	53	Correios	Atividades de correios, serviços postais e similares	75% trabalhadores	Teletrabalho / Presencial restrito	Teleatendimento / Presencial restrito	X			

DISTANCIAMENTO CONTROLADO
.GOV RS
NOVAS FAÇANHAS

BANDEIRA VERMELHA - Transportes

// Atividade				// Critérios específicos de funcionamento (conforme bandeira)			// Protocolos obrigatório (todas as bandeiras)	// Protocolos variáveis (recomendados)		// Restrições adicionais
Grupo	CNAE (2 díg.)	Tipo	Subtipos	**Teto de Operação**<br>Determina o percentual máximo de trabalhadores/público presentes no mesmo turno, ao mesmo tempo.<br><br>Deve respeitar ao nº máximo de pessoas no espaço físico, considerando o distanciamento interpessoal mínimo obrigatório (teto de ocupação).	**Modo de Operação**<br>Forma de operação da atividade, respeitando ao teto de operação, ao teto de ocupação do espaço físico e aos protocolos obrigatórios (ao lado).<br><br>**Trabalhadores**	**Atendimento**	**Decreto nº 55.2540:**<br>- Máscara / EPIs,<br>- Distanciamento,<br>- Teto de ocupação,<br>- Higienização,<br>- Proteção de grupo de risco,<br>- Afastamento de casos,<br>- Cuidados com o público,<br>- Atendimento do grupos de risco<br>- Informativo visível (operação, ocupação e cuidados)	**Monitora- mento de tempera- tura**	**Testagem dos trabalha- dores**	Conteúdo completo das normas obrigatórias específicas à atividade: coronavirus.rs.gov.br/portarias-da-ses
Transporte	49	Transporte terrestre	Transporte rodoviário fretado de passageiros	100% assentos	Teletrabalho / Presencial restrito / Ventilação cruzada (janelas abertas) ou sistema de renovação de ar	Teleatendimento / Presencial restrito	X	X		Decreto nº 55.240, Subseção II
Transporte	49	Transporte terrestre	Transporte rodoviário de carga	100% trabalhadores	Teletrabalho / Presencial restrito / Ventilação cruzada (janelas abertas) ou sistema de renovação de ar	Teleatendimento / Presencial restrito	X			
Transporte	49	Transporte terrestre	Transporte coletivo de passageiros (municipal)	50% capacidade total do veículo (ou normativa municipal)	Teletrabalho / Presencial restrito / Ventilação cruzada (janelas e/ou alçapão abertos) ou sistema de renovação de ar	Teleatendimento / Presencial restrito	X			Decreto nº 55.240, Subseção II
Transporte	49	Transporte terrestre	Transporte coletivo de passageiros (metropolitano tipo Executivo/Seletivo)	100% assentos	Teletrabalho / Presencial restrito / Ventilação cruzada (janelas e/ou alçapão abertos) ou sistema de renovação de ar	Teleatendimento / Presencial restrito	X			Decreto nº 55.240, Subseção II
Transporte	49	Transporte terrestre	Transporte coletivo de passageiros (metropolitano tipo Comum)	70% capacidade total do veículo	Teletrabalho / Presencial restrito / Ventilação cruzada (janelas e/ou alçapão abertos) ou sistema de renovação de ar	Teleatendimento / Presencial restrito	X			Decreto nº 55.240, Subseção II
Transporte	49	Transporte terrestre	Transporte rodoviário de passageiros (intermunicipal, tipo Comum, Semidireto, Direto, Executivo ou Seletivo)	100% assentos	Teletrabalho / Presencial restrito / Ventilação cruzada (janelas e/ou alçapão abertos) ou sistema de renovação de ar (NBR 15570)	Teleatendimento / Presencial restrito	X	X		Decreto nº 55.240, Subseção II<br><br>NBR 15570<br><br>Resolução Nº 5.917, de 24 de novembro de 2020, da ANTT
Transporte	49	Transporte terrestre	Transporte rodoviário de passageiros (interestadual)	50% assentos (janela)<br>25% coabitantes (corredor)	Teletrabalho / Presencial restrito / Ventilação cruzada (janelas e/ou alçapão abertos) ou sistema de renovação de ar (NBR 15570)	Teleatendimento / Presencial restrito	X	X		Decreto nº 55.240, Subseção II<br><br>NBR 15570<br><br>Resolução Nº 5.917, de 24 de novembro de 2020, da ANTT
Transporte	49	Transporte terrestre	Transporte ferroviário de passageiros (metropolitano)	50% capacidade total do vagão	Teletrabalho / Presencial restrito / Ventilação cruzada (janelas e/ou alçapão abertos) ou sistema de renovação de ar	Teleatendimento / Presencial restrito	X	X		Decreto nº 55.240, Subseção II
Transporte	50	Transporte aquaviário	Transporte aquaviário de carga	100% trabalhadores	Teletrabalho / Presencial restrito / Ventilação cruzada (janelas e/ou alçapão abertos) ou sistema de renovação de ar	Teleatendimento / Presencial restrito	X			
Transporte	50	Transporte aquaviário	Transporte aquaviário de passageiros	75% assentos	Teletrabalho / Presencial restrito / Ventilação cruzada (janelas e/ou alçapão abertos) ou sistema de renovação de ar	Teleatendimento / Presencial restrito	X			Decreto nº 55.240, Subseção II
Transporte	51	Transporte aéreo	Aeroclubes e aeródromos	25% trabalhadores	Teletrabalho / Presencial restrito (exclusivo para emergência Covid-19)	Sem atendimento ao público	X			
Transporte	52	Armazenamento de Transporte	Armazenamento, carga e descarga	100% trabalhadores	Teletrabalho / Presencial restrito	Teleatendimento / Presencial restrito	X			

BANDEIRA VERMELHA - Transportes

// Atividade				// Critérios específicos de funcionamento (conforme bandeira)			// Protocolos obrigatório (todas as bandeiras)	// Protocolos variáveis (recomendados)		// Restrições adicionais
Grupo	CNAE (2 díg.)	Tipo	Subtipos	**Teto de Operação**<br>Determina o percentual máximo de trabalhadores/público presentes no mesmo turno, ao mesmo tempo.<br><br>Deve respeitar ao nº máximo de pessoas no espaço físico, considerando o distanciamento interpessoal mínimo obrigatório (teto de ocupação).	**Modo de Operação**<br>Forma de operação da atividade, respeitando ao teto de operação, ao teto de ocupação do espaço físico e aos protocolos obrigatórios (ao lado).<br><br>Trabalhadores	Atendimento	**Decreto nº 55.2540:**<br>- Máscara / EPIs,<br>- Distanciamento,<br>- Teto de ocupação,<br>- Higienização,<br>- Proteção de grupo de risco,<br>- Afastamento de casos,<br>- Cuidados com o público,<br>- Atendimento do grupos de risco<br>- Informativo visível (operação, ocupação e cuidados)	**Monitora- mento de tempera- tura**	**Testagem dos trabalha- dores**	Conteúdo completo das normas obrigatórias específicas à atividade: coronavirus.rs.gov.br/portarias-da-ses
Transporte	52	Armazenamento de Transporte	Estacionamentos	100% trabalhadores	Teletrabalho / Presencial restrito	Teleatendimento / Presencial restrito	X			
Transporte	53	Correios	Atividades de correios, serviços postais e similares	50% trabalhadores	Teletrabalho / Presencial restrito	Teleatendimento / Presencial restrito	X	X		

DISTANCIAMENTO CONTROLADO
.GOV RS
NOVAS FAÇANHAS

BANDEIRA PRETA - Transportes

// Atividade				// Critérios específicos de funcionamento (conforme bandeira)			// Protocolos obrigatório (todas as bandeiras)	// Protocolos variáveis (recomendados)		// Restrições adicionais
Grupo	CNAE (2 díg.)	Tipo	Subtipos	**Teto de Operação**<br>Determina o percentual máximo de trabalhadores/público presentes no mesmo turno, ao mesmo tempo.<br><br>Deve respeitar ao nº máximo de pessoas no espaço físico, considerando o distanciamento interpessoal mínimo obrigatório (teto de ocupação).	**Modo de Operação**<br>Forma de operação da atividade, respeitando ao teto de operação, ao teto de ocupação do espaço físico e aos protocolos obrigatórios (ao lado).<br><br>**Trabalhadores**	**Atendimento**	**Decreto nº 55.2540:**<br>- Máscara / EPIs,<br>- Distanciamento,<br>- Teto de ocupação,<br>- Higienização,<br>- Proteção de grupo de risco,<br>- Afastamento de casos,<br>- Cuidados com o público,<br>- Atendimento do grupos de risco<br>- Informativo visível (operação, ocupação e cuidados)	**Monitora- mento de tempera- tura**	**Testagem dos trabalha- dores**	Conteúdo completo das normas obrigatórias específicas à atividade: coronavirus.rs.gov.br/portarias-da-ses
Transporte	49	Transporte terrestre	Transporte rodoviário fretado de passageiros	50% assentos (janela)	Teletrabalho / Presencial restrito / Ventilação cruzada (janelas abertas) ou sistema de renovação de ar	Teleatendimento / Presencial restrito	X	X		Decreto nº 55.240, Subseção II
Transporte	49	Transporte terrestre	Transporte rodoviário de carga	100% trabalhadores	Teletrabalho / Presencial restrito / Ventilação cruzada (janelas abertas) ou sistema de renovação de ar	Teleatendimento / Presencial restrito	X			
Transporte	49	Transporte terrestre	Transporte coletivo de passageiros (municipal)	50% capacidade total do veículo	Teletrabalho / Presencial restrito / Ventilação cruzada (janelas e/ou alçapão abertos) ou sistema de renovação de ar	Teleatendimento / Presencial restrito	X			Decreto nº 55.240, Subseção II
Transporte	49	Transporte terrestre	Transporte coletivo de passageiros (metropolitano tipo Executivo/Seletivo)	50% assentos (janela)	Teletrabalho / Presencial restrito / Ventilação cruzada (janelas e/ou alçapão abertos) ou sistema de renovação de ar	Teleatendimento / Presencial restrito	X			Decreto nº 55.240, Subseção II
Transporte	49	Transporte terrestre	Transporte coletivo de passageiros (metropolitano tipo Comum)	50% capacidade total do veículo	Teletrabalho / Presencial restrito / Ventilação cruzada (janelas e/ou alçapão abertos) ou sistema de renovação de ar	Teleatendimento / Presencial restrito	X			Decreto nº 55.240, Subseção II
Transporte	49	Transporte terrestre	Transporte rodoviário de passageiros (intermunicipal, tipo Comum, Semidireto, Direto, Executivo ou Seletivo)	50% assentos (janela)	Teletrabalho / Presencial restrito / Ventilação cruzada (janelas e/ou alçapão abertos) ou sistema de renovação de ar (NBR 15570)	Teleatendimento / Presencial restrito	X	X		Decreto nº 55.240, Subseção II<br><br>NBR 15570<br><br>Resolução Nº 5.917, de 24 de novembro de 2020, da ANTT
Transporte	49	Transporte terrestre	Transporte rodoviário de passageiros (interestadual)	50% assentos (janela)	Teletrabalho / Presencial restrito / Ventilação cruzada (janelas e/ou alçapão abertos) ou sistema de renovação de ar (NBR 15570)	Teleatendimento / Presencial restrito	X	X		Decreto nº 55.240, Subseção II<br><br>NBR 15570<br><br>Resolução Nº 5.917, de 24 de novembro de 2020, da ANTT

BANDEIRA PRETA - Transportes

// Atividade				// Critérios específicos de funcionamento (conforme bandeira)			// Protocolos obrigatório (todas as bandeiras)	// Protocolos variáveis (recomendados)		// Restrições adicionais
Grupo	CNAE (2 díg.)	Tipo	Subtipos	**Teto de Operação**<br>Determina o percentual máximo de trabalhadores/público presentes no mesmo turno, ao mesmo tempo.<br><br>Deve respeitar ao nº máximo de pessoas no espaço físico, considerando o distanciamento interpessoal mínimo obrigatório (teto de ocupação).	**Modo de Operação**<br>Forma de operação da atividade, respeitando ao teto de operação, ao teto de ocupação do espaço físico e aos protocolos obrigatórios (ao lado).<br><br>**Trabalhadores**	**Atendimento**	**Decreto nº 55.2540:**<br>- Máscara / EPIs,<br>- Distanciamento,<br>- Teto de ocupação,<br>- Higienização,<br>- Proteção de grupo de risco,<br>- Afastamento de casos,<br>- Cuidados com o público,<br>- Atendimento do grupos de risco<br>- Informativo visível (operação, ocupação e cuidados)	**Monitora-mento de tempera- tura**	**Testagem dos trabalha- dores**	Conteúdo completo das normas obrigatórias específicas à atividade: coronavirus.rs.gov.br/portarias-da-ses
Transporte	49	Transporte terrestre	Transporte ferroviário de passageiros (metropolitano)	50% capacidade total do vagão	Teletrabalho / Presencial restrito / Ventilação cruzada (janelas e/ou alçapão abertos) ou sistema de renovação de ar	Teleatendimento / Presencial restrito	X	X		Decreto nº 55.240, Subseção II
Transporte	50	Transporte aquaviário	Transporte aquaviário de carga	100% trabalhadores	Teletrabalho / Presencial restrito / Ventilação cruzada (janelas e/ou alçapão abertos) ou sistema de renovação de ar	Teleatendimento / Presencial restrito	X			
Transporte	50	Transporte aquaviário	Transporte aquaviário de passageiros	75% assentos	Teletrabalho / Presencial restrito / Ventilação cruzada (janelas e/ou alçapão abertos) ou sistema de renovação de ar	Teleatendimento / Presencial restrito	X			Decreto nº 55.240, Subseção II
Transporte	51	Transporte aéreo	Aeroclubes e aeródromos	25% trabalhadores	Teletrabalho / Presencial restrito (exclusivo para emergência Covid-19)	Sem atendimento ao público	X			
Transporte	52	Armazenamento de Transporte	Armazenamento, carga e descarga	100% trabalhadores	Teletrabalho / Presencial restrito	Teleatendimento / Presencial restrito	X			
Transporte	52	Armazenamento de Transporte	Estacionamentos	100% trabalhadores	Teletrabalho / Presencial restrito	Teleatendimento / Presencial restrito	X			
Transporte	53	Correios	Atividades de correios, serviços postais e similares	50% trabalhadores	Teletrabalho / Presencial restrito	Teleatendimento / Presencial restrito	X	X		

DISTANCIAMENTO CONTROLADO
NOVAS FAÇANHAS

FICHA TÉCNICA

GOVERNO DO ESTADO DO RIO GRANDE DO SUL
Governador: Eduardo Leite
Vice-Governador: Ranolfo Vieira Júnior

GABINETE DE CRISE PARA O ENFRENTAMENTO DA EPIDEMIA COVID-19
COMITÊ DE DADOS
Coordenador: Luís Lamb

GABINETE DO GOVERNADOR
ASSESSORIA TÉCNICA

SECRETARIA DE PLANEJAMENTO, GOVERNANÇA E GESTÃO - SPGG
Secretário: Claudio Leite Gastal
Secretária Adjunta: Izabel Matte

DEPARTAMENTO DE ECONOMIA E ESTATÍSTICA
ASSESSORIA DE COMUNICAÇÃO

SECRETARIA DE ESTADO DA SAÚDE - SES
Secretária: Arita Bergmann
Secretária-Adjunta: Aglaé Regina da Silva

DEPARTAMENTO DE ASSISTÊNCIA HOSPITALAR E AMBULATORIAL
DEPARTAMENTO DE AÇÕES EM SAÚDE
DEPARTAMENTO DE AUDITORIA DO SUS
Departamento de Regulação Estadual
Centro Estadual de Vigilância em Saúde

DISTANCIAMENTO CONTROLADO
RS
NOVAS FAÇANHAS

GOV RS

NOVAS FAÇANHAS

NO PLANEJAMENTO,
GOVERNANÇA E GESTÃO
NA SAÚDE

rs.gov.br

The Wayback Machine - https://web.archive.org/web/20200509175326/https://www.gov.sg/arti...

A Singapore Government Agency Website

What do the different DORSCON levels mean

The colours describe the current disease outbreak situation and what needs to be done

3 min read | Published on 06 Feb 2020

When there is an outbreak resulting in the spread of an infectious disease worldwide, Singapore puts in place prevention and response plans.

As part of this plan, the 'Disease Outbreak Response System Condition' (DORSCON) is a colour-coded framework that shows the current disease situation. The framework provides us with general guidelines on what needs to be done to prevent and reduce the impact of infections.

DORSCON takes into account:

- The current disease situation overseas
- How transmissible the disease is
- How likely it is to arrive in Singapore
- What impact it may have on Singapore's community

DORSCON ALERT LEVELS

(Disease Outbreak Response System Condition)

	GREEN	YELLOW	ORANGE	RED
**Nature of Disease**	Disease is mild **OR** Disease is severe but does not spread easily from person to person (e.g. MERS, H7N9)	Disease is severe and spreads easily from person to person but is occurring outside Singapore. **OR** Disease is spreading in Singapore but is (a) Typically mild i.e only slightly more severe than seasonal influenza. Could be severe in vulnerable groups. (e.g. H1N1 pandemic) **OR** (b) being contained	Disease is severe **AND** spreads easily from person to person, but disease has not spread widely in Singapore and is being contained (e.g. SARS experience in Singapore)	Disease is severe **AND** is spreading widely
**Impact on Daily Life**	Minimal disruption e.g. border screening, travel advice	Minimal disruption e.g. additional measures at border and/or healthcare settings expected, higher work and school absenteeism likely	Moderate disruption e.g. quarantine, temperature screening, visitor restrictions at hospitals	Major disruption e.g. school closures, work from home orders, significant number of deaths.
**Advice to Public**	• Be socially responsible: if you are sick, stay at home<br>• Maintain good personal hygiene<br>• Look out for health advisories	• Be socially responsible: if you are sick, stay at home<br>• Maintain good personal hygiene<br>• Look out for health advisories	• Be socially responsible: if you are sick, stay at home<br>• Maintain good personal hygiene<br>• Look out for health advisories<br>• Comply with control measures	• Be socially responsible: if you are sick, stay at home<br>• Maintain good personal hygiene<br>• Look out for health advisories<br>• Comply with control measures<br>• **Practise social distancing: avoid crowded areas**

MINISTRY OF HEALTH SINGAPORE

gov.sg

There are 4 statuses – Green, Yellow, Orange and Red, depending on the severity and spread of the disease. For each status, it details the impact on the community, such as the measures to be taken in daily life (e.g. temperature screening, border measures), and advice to the public (e.g to look out for travel advisories).

During the SARS experience in Singapore, the status was Orange, meaning the disease was severe and spread easily, but still contained.

You can keep updated on the DORSCON status for COVID-19 (coronavirus disease 2019) on the MOH website.

TOPICS

HEALTH

COVID-19

MOH PANDEMIC READINESS AND RESPONSE PLAN FOR INFLUENZA AND OTHER ACUTE RESPIRATORY DISEASES (REVISED APRIL 2014)

SCOPE

1. This document covers the readiness and response plan for novel acute respiratory infections with pandemic potential (e.g. influenza or SARS) and recommends appropriate public health measures and response actions prior to and during a pandemic. This plan can be applied to any acute respiratory pathogen (virus or bacteria) and guidance will be provided by MOH for individual novel pathogens where necessary.

INTRODUCTION

BACKGROUND

2. A pandemic is an epidemic[1] of an infectious disease that has spread through human populations across geographical regions globally. Pandemics occur when the general population has no or little immunity against an emerging or re-emerging pathogen. Over the centuries, infectious diseases with different modes of transmission have resulted in pandemics, including influenza (respiratory spread), cholera (water- and food-borne) and bubonic plague (vector-borne). This document focuses on the national response framework against **acute respiratory infections with pandemic potential**.

3. Influenza has been a major acute respiratory infection of interest as it caused four pandemics since the turn of the 20th century. Previous influenza pandemics have shown no predictable periodicity or pattern and all differed with respect to antigenic subtype, epidemiology and disease severity. They can be more or less severe than preceding seasonal epidemics – while the 1918 H1N1 pandemic was associated with high mortality, the 2009 H1N1 pandemic was relatively mild. Additional details on influenza viruses can be found in Annex A.

4. Other acute respiratory diseases can also have pandemic potential. These include the Severe Acute Respiratory Syndrome-associated Coronavirus (SARS-CoV) and the Middle East Respiratory Syndrome Coronavirus (MERS-CoV), which are of concern due to their clinical severity. Additional details of these coronaviruses can be found in Annex B.

PUBLIC HEALTH THREAT ASSESSMENT

5. Both the ability of respiratory pathogens to spread (**transmissibility**) and the ability to cause serious illness (**virulence**) determine the extent of the outbreak and its resulting public health impact.

6. For example, influenza A viruses undergo reassortment events (i.e. exchange and recombination of genetic material between viruses), generating new strains with unpredictable transmissibility, virulence, and pandemic potential. However, the factors that

---

[1] An **epidemic** occurs when new cases of a certain disease, in a given human population, and during a given period, substantially exceed what is expected based on recent experience.

drive the emergence of pandemic influenza are not well understood. Reassortment events in swine remain a significant source of pandemic potential influenza viruses. Mammalian-adapted virus strains in other animal species, along with avian influenza virus strains that have shown the potential to infect humans, are also a potential threat. The laboratory syntheses of H5N1 influenza strains that can transmit efficiently among humans show their pandemic potential.

7. Similarly, coronaviruses undergo genetic mutations and recombination at a rate similar to that of influenza viruses. Due to the extensive genetic diversity of these viruses, infections that result from coronaviruses can be difficult to predict and manage. These viruses may cause epidemic diseases of pandemic potential.

8. Currently, the primary risk factor for human infection for avian influenza and novel coronaviruses appears to be direct or indirect exposure to contaminated environmental sources, with some human spread among close contacts. For efficient human-to-human transmission to occur, these pathogens must undergo further genetic changes and adaptation.

9. Given the unpredictability of the respiratory pathogens, pandemics will likely occur again. Modeling studies notwithstanding, predictions of how particular disease strains will evolve remain highly speculative. Continued surveillance and monitoring of the global developments in the evolution of acute respiratory infections as well as pandemic preparedness and planning will serve to prepare us against the emergence of the next pandemic.

CLINICAL FEATURES OF ACUTE RESPIRATORY INFECTIONS

SYMPTOMS

10. Symptoms of different acute respiratory infections may vary depending on the pathogen. General symptoms may include constitutional symptoms such as fever, myalgia (muscle pains), headache, malaise (body discomfort), and respiratory symptoms such as cough, sore throat, and rhinitis (nasal inflammation). Certain populations such as the young, and elderly and those with underlying medical conditions may be at risk of developing a more severe illness including pneumonia.

11. For example in influenza cases, uncomplicated illness is characterised by the abrupt onset of symptoms. Among children with influenza illness, otitis media (middle ear infection), nausea, and vomiting are also commonly reported. Influenza illness typically resolves after a few days for most patients, although cough and malaise can persist for more than 2 weeks. Influenza can exacerbate underlying medical conditions (e.g. pulmonary or cardiac disease), lead to primary viral or secondary bacterial pneumonia, or occur as a co-infection with other pathogens.

12. It is often difficult to identify a specific pathogen based on clinical symptoms alone because there is a wide range of pathogens that cause similar symptoms including, but not limited to, *Mycoplasma pneumoniae*, adenovirus, respiratory syncytial virus, rhinovirus, parainfluenza viruses, and *Legionella* spp. Laboratory tests are required to conclusively identify these pathogens, although in a pandemic where the majority of cases are caused by the pandemic pathogen, it is possible to intervene based on clinical symptoms alone.

LABORATORY FEATURES

13. Diagnostic tests available for respiratory infections include cultures (e.g. viral cultures), serology, polymerase chain reaction (PCR), immunofluorescence assays, and rapid tests. The sensitivity and specificity of any test may vary by the laboratory that performs the test, the type of test used, and the type of specimen tested. Among respiratory specimens for viral isolation or rapid detection of influenza, nasopharyngeal specimens are typically more effective than throat swab specimens. For SARS and MERS-CoV, lower respiratory specimens are preferred as these viruses preferentially affect the lower respiratory tract. As with any diagnostic test, results should be evaluated in the context of other clinical information available to health-care providers.

14. Commercial rapid diagnostic tests are also available for some diseases. For example, rapid tests to detect influenza viruses within 30 minutes are widely available. These rapid tests differ in the types of influenza viruses they detect and whether they can distinguish between influenza types. The specificity and, in particular, the sensitivity of rapid tests are lower than culture or PCR, and vary by test.

SEVERE INFECTIONS AND MORTALITY

15. The proportion of severe infections and mortality from respiratory infections varies widely, depending on risk factors such as comorbid conditions, age and immune status. It is therefore important to understand the virulence patterns of the disease in question. MERS-CoV tends to result in severe infections among older individuals and those with comorbid conditions, while SARS resulted in severe infections across a wide age spectrum. Influenza-related hospitalisation and deaths often result from pneumonia as well as from exacerbations of cardiopulmonary conditions and other chronic diseases. However, in an influenza pandemic, the mortality rates among the different population groups could be vastly different from that in seasonal influenza cases.

EPIDEMIOLOGY

16. We assume that the epidemiological features of a future acute respiratory infection pandemic will be consistent with previous known human epidemics and pandemics. However, these features should not be interpreted as being definitive of a novel pandemic pathogen. In the event of a pandemic, active and enhanced surveillance will be required in the early stages of the outbreak to determine the true nature of the pandemic pathogen.

17. **Incubation period.** The incubation period will vary depending on the pathogen, and will have to be determined through surveillance. The incubation period for human influenza is typically 2 days, with a range of 1 - 4 days. However, human cases of avian influenza have incubation periods around 2 - 8 days and as long as 14 - 17 days. The incubation period for SARS was up to 10 days, and MERS-CoV up to 14 days. Depending on the pathogen, the incubation period for field investigations and monitoring of contacts will also vary correspondingly.

18. **Transmission.** There are 3 modes of transmission for respiratory pathogens:

a. Large droplet spread (the main route of transmission for influenza);

b. Transmission through droplet nuclei, i.e. airborne spread (Sneezing, coughing and even talking can produce droplets of wide variety of particle sizes that can facilitate droplet or droplet nuclei infection); and

c. Contact, either direct or indirect, with respiratory secretions.

19. **Infectious period.** The infectious period depends on the pathogen. In the case for influenza, infected persons can be infectious (i.e. spread the disease to others) one day before the onset of symptoms. Conversely, for coronaviruses, infected persons are usually infectious when symptomatic.

20. **Organism survival in different environments.** Survival of respiratory pathogens outside the body varies with multiple factors including temperature and humidity. The few available studies have been on the influenza virus, showing that it generally survives 24-48 hours on hard, non-porous surfaces, 8-12 hours on cloth/paper/tissue, and 5 minutes on hands. Survival is generally enhanced under conditions of low humidity and in the cold.

NATIONAL STRATEGY FOR PANDEMIC RESPONSE

OVERVIEW

21. The national strategy for pandemic response is to establish an effective surveillance system to detect the importation of a novel acute respiratory pathogen with pandemic potential and to mitigate the consequences when the first wave hits. For influenza viruses and other pathogens where vaccines production is possible, vaccination will be provided as soon as a vaccine becomes available, which is likely to be beyond the first wave. To assist planners in preparing for the worst-case scenario, we have provided some planning assumptions for consideration in Annex C. However, the actual epidemic will likely differ and real-time information will guide the response.

22. Our objective is to sustain the nation through the first epidemic wave by minimising mortality and morbidity through the use of measures that are proportional to the assessed public health impact, while ensuring preparedness for vaccination of the entire population when a vaccine becomes available.

23. The recommended national response measures are dependent on the phase of the local epidemic and the assessed public health impact of the epidemic.

a. In the initial **phase when cases are mostly overseas**, efforts will be to detect and **minimise importation** of the disease for as long as possible to allow time to gain a better understanding of the disease and to prevent disease spread. Depending on the extent of transmission and the virulence of the disease, this may necessitate border health control measures such as travel advisories or temperature screening. For diseases with potentially severe public health impact, border health control measures similar to that implemented for SARS will be adopted. Some containment measures may be needed for the individual cases that are imported to Singapore, especially if these cases result in local clusters.

b. **When the disease spreads in the Singapore community**, for a relatively **mild disease** with low to moderate public health impact, efforts to contain the disease may be halted and a move towards **reducing the community impact** of the pandemic may be made

sooner. This will include measures aimed at extending appropriate treatment to community cases and reducing the spread through social distancing.

c. Conversely, a pathogen with greater virulence and causing more **severe public health impact** would necessitate sustained measures to **contain its spread** (e.g. SARS). While we may not be able to fully contain the disease, measures can still be taken to delay or limit spread throughout the community. This will reduce the number of infections at any one point in time and reduce disruption to essential services. When the disease spreads widely across Singapore, **measures to reduce community impact** will set in.

d. A key drive throughout the epidemic will be to **communicate** with and **educate** the public and securing their co-operation with our efforts. The key message to the public will be the importance of each individual's responsibility in preventing disease spread through personal hygiene and being socially responsible in behaviour.

24. In essence, our response aims at achieving the following three broad outcomes:

a. **Reduce morbidity and mortality through providing healthcare and early treatment of infected cases.**
During the local epidemic, outpatient care will be provided by polyclinics and participating primary care clinics[2] including Pandemic Preparedness Clinics (PPCs). Severe cases will be referred to hospitals for further treatment. All healthcare facilities will have to ramp up their surge capacity to cope with the possible increase in cases. For influenza, depending on the epidemic's severity, clinically diagnosed cases may be treated with anti-virals, preferably within 48 hours of the onset of symptoms.

b. **Slow and limit the spread of disease to reduce the surge on the healthcare system.**
During an epidemic, the estimated number of cases requiring medical attention could easily overwhelm our healthcare system. Healthcare workers (HCWs) are vital to combat the disease. They will be protected through infection control measures and personal protection practices. In addition, for severe epidemics of influenza, front-line HCWs working at the restructured hospitals, polyclinics and participating primary care clinics may be given anti-viral prophylaxis to protect them during the peak of the epidemic.

c. **Maintain essential services in Singapore and limit community disruptions.**
It is important to limit the epidemic's impact on essential services. Work units will be able to improve staff availability if there is an established business continuity plan to ensure that proper health control measures for both individuals and communities within the organisation are in place. In severe epidemics, certain segments of essential services will need added protection to maintain operational capacity with undisrupted services. In such situations, these work units may be provided with prophylaxis, if appropriate, e.g. anti-virals for influenza.

NATIONAL COMMAND AND CONTROL STRUCTURE

25. Singapore has in place a crisis management system designed to prepare for and respond to a wide variety of hazards. The Homefront Crisis Management System (HCMS) is the national framework for coordinating whole-of-government planning and response

[2] Primary care clinics broadly encompass community based GP clinics and paediatric clinics (ie non-hospital/medical centre based).

during a homefront crisis that has national significance and impact. The HCMS is led by a **Homefront Crisis Ministerial Commitee (HCMC)** which is chaired by the Minister for Home Affairs to provide strategic and political guidance during a crisis.

26. Supporting the HCMC is the Homefront Crisis Executive Group (HCEG) chaired by the Permanent Secretary for Home Affairs. The role of the HCEG is to ensure that a comprehensive and integrated multi-agency system is in place to anticipate threat and disaster scenarios, and to prepare contingency plans to avoid, pre-empt, prevent or ultimately deal with any peacetime emergency. The HCEG is supported by various Crisis Management Groups (CMG)s that deal with the operational issues under their charge. MOH chairs the Crisis Management Group (CMG) (Health).

27. **CMG (Health)**

a. **Immediate Response**. In response to a possible novel pathogen case or outbreak, medical surveillance will trigger an immediate response workflow. The CMG (Health) will provide timely situational updates to agencies and to coordinate the initial interagency preparation for **DORSCON (Disease Outbreak Response System Condition)** activation (see the following section). An assessment of the threat and impact to public health will be made, which will include a clinical assessment and the extent of disease spread. Medical directives on case management and infection control measures to undertake in healthcare institutions will be issued by MOH, where necessary. HCMC and/or HCEG may also be activated to coordinate the Whole of Government response to the threat.

b. **Daily Management**. Once the DORSCON is elevated to **Yellow** and beyond, CMG (Health) will be set up and a daily management cycle established to deal with the outbreak. This daily cycle will facilitate the management of daily developments and coordinate the medical and operational responses.

A schematic diagram of the Homefront Crisis Management System (HCMS) structure is given below.

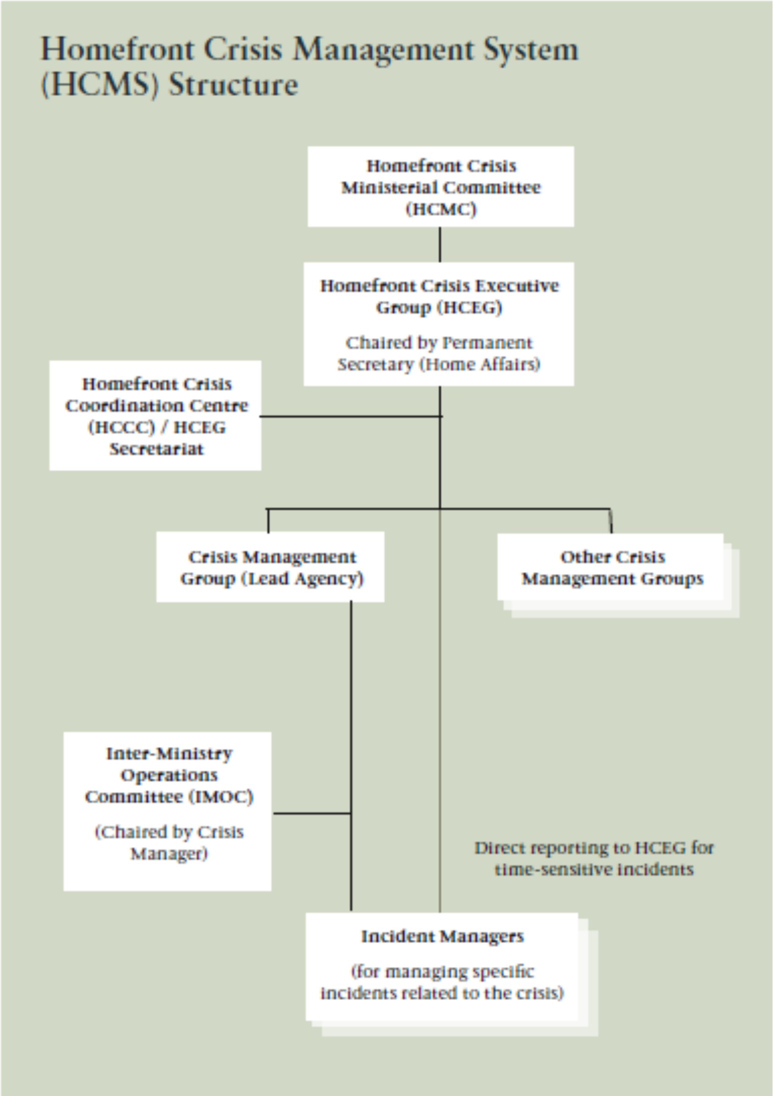

Extracted from National Security Coordination Secretariat website

DISEASE OUTBREAK RESPONSE SYSTEM

OVERVIEW

28. The **DORSCON (Disease Outbreak Response System Condition)** is a generic framework that enables the Whole-Of-Government to respond immediately to any outbreak and serves as the nucleus to ramp up for a higher level of response during a pandemic.

29. The response to any outbreak is determined by the local **disease situation** in Singapore **AND** the **public health impact level**, which is a measure of overall severity based on risk assessment and denoted by four DORSCON levels: Green, Yellow, Orange and Red. This matrix approach provides for the flexibility to adopt responses as building blocks that can be tailored to each unique scenario.

30. There are three **possible disease response phases in Singapore** - Alert, Containment and Mitigation.

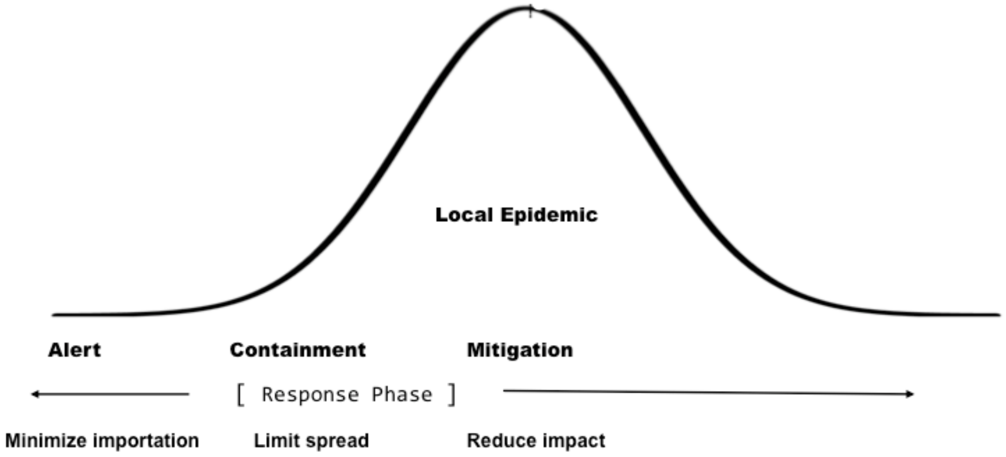

a. **ALERT**: The disease is mainly overseas and the response is to detect and minimise importation of disease. This requires border control measures and may require measures to try to **stop the spread from individual cases or resultant clusters if they are imported** into Singapore.

b. **CONTAINMENT:** The disease has arrived in Singapore and the primary response is to **stop or limit the spread of the disease** as much as possible. This requires extensive contact tracing and quarantine measures.

c. **MITIGATION:** The disease is spreading widely through the community, and measures to try to stop its spread are no longer effective. The response is to **reduce the overall impact** of the disease in the community. This requires an overall activation of business continuity plans, surge capacity for healthcare and essential services, and community-based public health measures.

31. The **scale of the response** for each of the phases is dependent on the overall public health impact of the disease, and represented by different DORSCON levels. The **DORSCON** framework comprises four levels: GREEN, YELLOW, ORANGE and RED, based on the likely public health impact on Singapore. **Assessment of the public health impact** will consider multiple factors such as disease characteristics (ability to cause serious disease (virulence) or spread in humans (transmissibility), availability of preventive interventions, travel connectivity of affected areas to Singapore (i.e. likelihood of importation), geographical spread overseas and in Singapore, and recommendations by international health authorities such as the World Health Organisation (WHO). Activation of the DORSCON levels will be based on MOH's recommendations and endorsed by HCEG and HCMC.

**DORSCON Level**	**Public Health Impact**
**GREEN**	Negligible to low
**YELLOW**	Low to moderate
**ORANGE**	Moderate to high
**RED**	High

32. The DORSCON framework contains a **public communications component** to convey the health impact to the public and to advise them on how to respond. Clear guidance to agencies to maintain operational readiness and undertake preparedness planning is also

provided. In order for the DORSCON framework to be relevant for mild and severe epidemics across the different phases, the definitions of the four DORSCON levels have been crafted to correspond to the public health impact of the disease. We have included scenarios that are applicable at each DORSCON level to highlight the possibilities and the need to allow for flexibility and adaptability in the actual response.

DEFINITION OF THE DORSCON LEVELS

33. **GREEN** - Presence of a disease that is assessed to have **<u>negligible to low</u>** public health impact. Possible scenarios include:

Scenario A: Novel disease **is reported overseas with no or limited person to person transmission.**

- Emphasis on **detecting and minimising importation** (i.e. **ALERT phase**), and if cases are imported, implementing measures to stop further spread (i.e. some CONTAINMENT is needed)**.**
- Examples include Avian influenza (H5N1) and Middle East Respiratory Syndrome (MERS) coronavirus.

Scenario B: Local spread of a novel disease that has **similar or lower virulence and transmissibility as seasonal influenza.**

- Disease is mild and self-limiting, even if it can spread easily from person-to-person.
  Emphasis on **reducing the overall impact on the community** (i.e. **MITIGATION phase**)

34. **YELLOW** - Disease is assessed to have **<u>low to moderate</u>** public health impact. Possible scenarios include:

Scenario A: Disease is largely overseas with high virulence and can spread from person to person. However, the disease situation appears to be controlled overseas.

- There is the risk of importation of the disease to Singapore resulting in local sporadic cases/clusters.
- Emphasis on **detecting and minimising importation (i.e. ALERT phase)**, and if cases are imported, implementing measures to stop further spread (i.e. some CONTAINMENT is needed).
- Examples include community clusters of avian influenza in another country, or imported cases or clusters in Singapore.

Scenario B: Disease spreads easily from person to person and causes generally mild illness, but can cause serious illness in vulnerable groups.

- Disease may be local or overseas but is expected to become widespread in Singapore.
- Emphasis on **reducing the overall impact on the community (i.e. MITIGATION phase)**
- Example includes the H1N1 influenza A (2009) epidemic in Singapore

Scenario C: Vaccine available

- Disease shows high virulence and high transmissibility but a vaccine is available. Cases may occur before vaccination program achieves full coverage or due to individual vaccine failures.

- Emphasis on **reducing overall impact on community through vaccination (i.e. MITIGATION phase).**

35. **ORANGE** – The disease is assessed to have **moderate to high** public health impact. Possible scenarios include:

Scenario A: Disease is of high virulence, can spread from person to person, and is spreading overseas.

- The risk of importation to Singapore is high.
- Emphasis on **detecting and minimising importation** (i.e. **ALERT phase**).
- Examples include a SARS-like virus with spread overseas.

Scenario B: Disease is of high virulence, can spread from person to person, and it is in Singapore but is controlled.

- Cases or clusters occur in Singapore.
- Emphasis on aggressively **trying to stop or limit further spread (i.e. CONTAINMENT phase)**.
- Example includes the Singapore SARS experience in 2003.
- The disease may spread more widely across Singapore, and the DORSCON level may remain in ORANGE or escalate to RED depending on the overall impact assessment. In such cases, selective measures to reduce community impact may be instituted in ORANGE.

36. **RED** – **Mitigation Phase** – The disease is of high virulence, is spreading widely from person to person in Singapore, and the disease is assessed to have **high** public health impact.

- Multiple clusters or widespread community transmission in Singapore
- Emphasis on **reducing the overall impact on the community through social distancing (i.e. MITIGATION phase)**
- Example is if SARS had spread widely in Singapore.

37. Components of pandemic response are outlined in subsequent sections. It should be noted that unless specified, measures for a particular response phase would be applicable to all relevant DORSCON levels. E.g. surveillance recommendations for an Alert Phase would apply regardless of whether we are in DORSCON Green, Yellow or Orange as long as an Alert Phase occurs. A diagram of the DORSCON levels and the phases of the local epidemic are shown below to depict possible scenarios.

	**Response Phases**		
	**Alert**	**Containment**	**Mitigation**
**GREEN**	✓ **Prevent importation**, if disease is mainly overseas (High virulence, no or very limited transmissibility)	✓ **Stop the spread** from imported case(s), if any	✓ **Reduce the impact through patient management** (Similar or lower virulence and transmissibility as seasonal influenza)
**YELLOW**	✓ **Prevent importation**, if disease is mainly overseas (High virulence but low	✓ **Stop the spread** from imported case(s), if any	✓ **Reduce the impact through vaccination or patient management** (Low virulence but high transmissibility)

	transmissibility)		
**ORANGE**<br>High virulence and transmissibility	✓ **Prevent importation**, if disease is mainly overseas	✓ **Stop or limit the spread from** local cases/clusters	✓ **Selective mitigation measures** may be activated as necessary
**RED**<br>High virulence and transmissibility			✓ **Reduce the impact through social distancing** to counter widespread transmission

COMPONENTS OF THE PANDEMIC RESPONSE

SURVEILLANCE

38. Surveillance requires internal and external monitoring for disease pathogens and activity. Early identification of novel pathogens through an integrated surveillance system is essential for pandemic detection and vaccine preparation.

39. An integrated national surveillance system is in place that can detect the first few cases or unusual clusters of disease in animals or humans and the timely identification of a novel pathogen. Comprehensive novel pathogen surveillance comprises the following main components: community surveillance, laboratory surveillance, hospital surveillance, disease notification, veterinary surveillance, and external surveillance.

a. **Community Surveillance.** Community-wide surveillance of acute respiratory infections (ARI) has been well established in Singapore. Weekly reports are compiled from the public-sector hospitals and polyclinics.

b. **Laboratory Surveillance.** Surveillance of influenza viruses and other acute respiratory infections is routinely performed by the National Public Health Laboratory on respiratory samples from hospitals and polyclinics.

c. **Hospital Surveillance.** Hospitals continue to support the post-SARS epidemic surveillance system. Patients fulfilling the surveillance criteria are reported to MOH.

d. **Disease Notification.** For novel agents, gazetting the disease under the Infectious Diseases Act may be necessary to mandate notification by doctors and laboratories.

e. **Veterinary Surveillance.** The Agri-Food and Veterinary Authority (AVA) carries out surveillance on poultry and other animals, based on the assumption that poultry and animal infection and deaths may precede human infection.

f. **External surveillance.** MOH performs continuous monitoring of infectious disease situations in the region and globally, via various sources, to identify external health risks and threats. Where incidents of concern emerge in a given country, clarification is directly sought through international contacts.

40. In the absence of novel diseases, the strategy is to maintain situational awareness through general surveillance and to monitor for new disease threats. Surveillance enables MOH to monitor the local and global disease situation and ensure an integrated surveillance system that is well prepared to detect the first case or unusual clusters in animals or humans. Routine **laboratory and veterinary surveillance** will strengthen information on emerging infections.

ALERT

41. Where a novel disease is present, **internal surveillance** will be enhanced by closely monitoring the local situation, stepping up surveillance of high-risk groups and enhancing

laboratory capabilities. MOH will also liaise with the WHO and affected country(s) for information on the disease and situation for effective **external surveillance**.

42. Depending on the risk assessment, surveillance may be further enhanced to include **hospital surveillance**, whereby healthcare institutions will report atypical pneumonias, review case definitions, report unusual health events, monitor hospital staff illness and report clusters of concern of acute respiratory illness. Hospital surveillance will also be important in scenarios where there is a risk of hospital clusters occurring as a result of inadvertent transmission within hospitals.

Containment and Mitigation

43. **Wider community surveillance:** Besides maintaining internal, external and hospital surveillance described earlier, wider community surveillance will also be necessary when the disease becomes entrenched in Singapore. The aim is to monitor the disease situation at the national level for optimal allocation of resources for case management. Polyclinics will step up reporting for ARI / influenza-like illness (ILI) attendances from weekly to daily. As there is community transmission, acute respiratory illness clusters among healthcare staff and/or patients in healthcare facilities including nursing homes and step-down care facilities will be monitored closely and reported to MOH.

Management of Suspect Cases

Alert and Containment

44. **Referral of cases**: If screening of suspect cases is done centrally, clinics and step down care facilities will refer cases to TTSH Emergency Department for assessment and admission via 995, if necessary. Children under 16 years of age will be sent to KKH for assessment. MOH will inform the medical community if a dedicated ambulance service for transportation of potentially infectious cases to TTSH/ CDC is to be activated. Other hospitals can continue to manage suspect cases that present at their emergency departments without the need to transfer them to TTSH. Transfer to TTSH or another hospital is recommended only if clinically indicated, e.g. patient requires care in ICU isolation that is not available in certain hospitals. Paediatric and Obstetric services will be set up at TTSH/ CDC by KKH when necessary – These services will be activated by MOH.

45. **Triaging**: Hospitals will implement triaging of febrile patients at emergency departments so that suspect cases can be identified early. Such cases will be evaluated in designated rooms.

Mitigation

46. **Outpatient management:** Polyclinics and Pandemic Preparedness Clinics (PPCs) will be activated to provide outpatient management. Influenza cases will be treated with anti-virals where clinically indicated. Vaccination will be offered if and when it becomes available.

47. **Referral:** Severe cases seen in primary care settings will be referred to restructured hospitals (RHs) for treatment. Both RHs and private hospitals will need to manage large numbers of pandemic as well as non-pandemic patients. Hospitals, polyclinics and PPCs will

segregate pandemic and non-pandemic cases to minimise transmission through close contact.

48. **Triaging**: Triage for pandemic patients at polyclinics/ primary care clinics, hospital specialist outpatient clinics, and hospital emergency departments should be implemented.

INFECTION CONTROL IN HEALTHCARE SETTINGS

49. Pandemic disease-infected HCWs, patients and visitors can spread infection within and outside healthcare facilities. Transmission risks are primarily from unprotected exposures to unrecognized cases in inpatient and outpatient settings. It can occur through respiratory droplets and close contact with infected patients, and through exposure during aerosol-generating procedures. The risk depends substantially on the type of activity and type of patient contact.

50. Strict adherence to appropriate infection control practices, including the use of appropriate PPE (personal protection equipment), good hand and respiratory hygiene, and environmental hygiene helps prevent transmission. Infection control requirements will differ according to the risk of infection among various clinical areas, and the epidemic phase. For example, emergency departments that deal with high-risk cases will require heightened infection control measures in contrast to healthcare workers in the primary care setting generally.

ALERT AND CONTAINMENT

51. Appropriate PPE[3] are recommended for HCWs when attending to all suspect and confirmed cases. In all other clinical areas, appropriate PPE is recommended based on the patient contact type, activity / procedure, and disease virulence / transmissibility.

52. The use of N95 masks is considered where there is anticipation of aerosolised generating procedures in the presence of exposure to high-risk pathogens.

MITIGATION

53. In all clinical areas, appropriate PPE is recommended based on the patient contact type, activity / procedure, and disease virulence / transmissibility.

VISITOR CONTROL AND TEMPERATURE SCREENING IN HOSPITALS

54. Visitor registration will be implemented at all hospitals as a best practice during peacetime. Routine temperature screening for hospital visitors is not necessary. Hospitals will periodically review the need to restrict hospital visitors and maintain a log of visitor contact details.

55. During a pandemic, more stringent visitor restriction in affected hospitals (including stopping of all visits) will be considered if necessary. Similarly, temperature screening and screening for acute respiratory symptoms will be carried out for visitors to selected or all clinical areas if necessary. In situations where there is widespread community transmission

[3] PPE may include masks, gloves, gowns, and eye protection.

and social distancing strategies are employed, a "No visitor” rule will be implemented in all hospitals.

ISOLATION AND DISCHARGE CRITERIA OF SUSPECT AND CONFIRMED CASES

56. All suspect and confirmed cases will be isolated, as far as operationally feasible. When the number of cases exceeds isolation capacity, cases will be cohorted.

57. Cases will be discharged when well or if deemed non-infectious. Quarantine orders can be served to discharged patients, if necessary to prevent further spread.

HANDLING OF DECEASED PERSONS

58. Guidelines on the handling of the deceased during pandemics are generally independent of DORSCON levels and are instead based on risk assessment of exposure and its consequences, characteristics of the infective agent, and the availability of appropriately trained personnel and adequately equipped facilities to handle the deceased.

59. There is a possibility of the disease spreading when persons handling the body come into contact with body fluids. Such persons include healthcare professionals, health attendants and porters and staff of funeral parlors. Precautions to be taken when handling all bodies of suspect and confirmed cases include:

- Wearing of gloves;
- Wearing of surgical masks (N95 masks may be recommended for certain infections, e.g. SARS, or in high-risk situations);
- Carrying out proper hand-washing after handling the body;
- Wearing of disposable gowns, if the risk of splashes is present;
- Minimising the number of persons handling the body; and
- Disposing used items and potentially contaminated articles in properly labelled biohazard bags.

MOH may issue additional guidelines for specific infectious diseases (e.g. SARS). For example, double-bagging may be required and there may be restrictions on embalming and holding of wakes.

60. Ritual washing of the deceased may be permitted depending on the nature of the organism (e.g. transmissibility, infectiveness, virulence and susceptibility to decontamination). The risk of exposure during ritual washing will be assessed by MOH and guidance will be provided during an actual pandemic.

61. Similarly, whether embalming will be permitted depends on the risk assessment of exposure to the infective agent during the activity. MOH may also restrict the embalmers who can carry out this task based on embalming facilities having the necessary biosafety safeguards and risk management systems in place. Embalming may be prohibited if the risk and consequences of exposure are severe. MOH will issue additional guidance on embalming during a pandemic.

BORDER CONTROL MEASURES

62. Measures implemented at the border checkpoints are our first line of defence against possible importation of pandemic case(s). To facilitate timely and effective responses for ramping up such defense measures, MOH will coordinate with the concerned key border control agencies (Civil Aviation Authority of Singapore, Immigration and Checkpoint Authority & Maritime Port Authority), following the activation of the Border Health Control Work Group by HCEG. The border control measures include inbound and outbound temperature screening, issue of Health Advisories and Health Advisory Notices (HANs), installing Health Advisory Posters and filling in of Health Declaration Cards (HDCs), and will be implemented based on the epidemic phase and DORSCON level.

63. The control measures will be based on the local epidemic phases but flexible in accordance with the changing circumstances. However, any timely initial assessment of an emerging disease would be based on incomplete and limited information; hence the measures taken would err on the side of caution with more intense efforts until such time when the disease profile becomes clearer.

64. Temperature screening may be activated at selected or all border checkpoints. Thermal scanners will be used to pick up potentially fever cases and persons will be screened by the medical personnel and managed accordingly. Depending on the situation, a dedicated ambulance service will ferry suspect cases to hospital for follow-up.

TEMPERATURE SCREENING IN INSTITUTIONS / BUILDINGS

65. **ALERT**: Temperature screening is not required.

66. **CONTAINMENT and MITIGATION**: Depending on the risks in the respective environments, temperature screening will be recommended to institutions in the community, including schools, government agencies and businesses.

CONTACT TRACING AND QUARANTINE

67. Contact tracing is the process of identification of people who have possibly been infected after exposure to cases with infectious diseases for the purpose of containing the spread of the disease. Following contact tracing, phone surveillance or quarantine may be necessary for persons (contacts) who are well, depending on the risk assessment.

68. Quarantine refers to the segregation of well persons who may have been exposed to an infectious agent, and may be infected but are not yet ill. The Infectious Diseases Act (IDA) gives MOH the authority to issue quarantine orders.

69. **ALERT and CONTAINMENT:** Contact tracing will be carried out to allow for active case detection and monitoring of close contacts, including phone surveillance if necessary.

70. Quarantine of close contacts will be carried out for effective containment of cases to limit the spread to the community. The duration of the quarantine will be determined based on the incubation period of the disease. Quarantine usually occurs in the home. However, if a person under quarantine is unable to be quarantined at his/her home, dedicated quarantine facilities will be made available. Contact tracing and quarantine will be sustained for as long as operationally feasible and if it continues to have an impact on reducing transmission.

71. **MITIGATION:** Contact tracing, phone surveillance and quarantine would be stopped, as such measures would no longer be effective.

Social Distancing

72. Social distancing aims to reduce the number of person-to-person contacts in order to slow the spread of infection. This reduces the surge on the medical care system. Social distancing is thus an important public health measure to reduce transmission until an effective vaccine is available.

73. Social distancing measures, such as cancellation of mass events, can be activated in **DORSCON Red**, to stem ongoing community transmission. Public health messaging will emphasize the need to avoid mass gathering. Business continuity plans should kick in to scale down operations and maintain essential services during such periods. Under certain circumstances, selective social distancing measures may be required in **Containment (DORSCON Orange) or even during the Mitigation phase of a milder pandemic (DORSCON Yellow)**, or to reduce spread in specific contexts.

74. School closure carries a high public signature and should be considered in the following scenarios where there is a need to safeguard wider community health:

- **DORSCON Orange:** Selective school closures may be implemented to break the chain of transmission of cases or clusters that are detected in schools.
- **DORSCON Red:** School closures will be implemented if necessary as part of the wider social distancing strategy to mitigate the outbreak.

Medical Treatment

75. Medicines may be effective in the early treatment of some acute respiratory diseases with pandemic potential. For influenza, anti-virals are effective for both the prevention (prophylaxis) and early treatment if administered within 48 hours following the onset of illness. Their use can reduce the duration of symptoms and the likelihood of complications requiring hospitalisation. For example, the currently stockpiled anti-viral drug to be used for prophylaxis and treatment in the event of an influenza pandemic is either Oseltamivir or Zanamivir.

Pandemic Vaccine

76. In a pandemic, it is very likely that vaccines, if technologically feasible, will only be available after 4-6 months. In the initial stages, these will be in short supply. However, vaccination is the key strategy in response to an influenza pandemic.

77. Initially, when vaccines are in short supply, vaccination will be provided to priority groups, such as those at higher risk of disease-related complications and personnel providing essential services (e.g. healthcare workers). As vaccines become more readily available, vaccination will be expanded to the rest of the population.

Communications

78. Members of the public will be guided by the DORSCON alert levels for the appropriate actions to be taken. As the severity and spread of the disease increase, the

public will be advised to take greater measures to keep themselves safe. The public are advised to refer to the MOH website for the latest situational update and appropriate measures at each DORSCON alert level as follows:

**Colour**	**Green**	**Yellow**	**Orange**	**Red**
**Nature of disease**	Disease is mild<br>**OR**<br>Disease is severe but does not spread easily from person to person<br>(e.g. MERS, H7N9)	Disease is severe and spreads easily from person to person but is occurring outside Singapore.<br>**OR**<br>Disease is spreading in Singapore but is typically mild i.e. only slightly more severe than seasonal influenza. Could be severe in vulnerable groups. (e.g. H1N1 pandemic).	Disease is severe AND spreads easily from person to person, but disease has not spread widely in Singapore and is being contained (e.g. SARS experience in Singapore).	Disease is severe AND is spreading widely.
**Impact on daily life**	No disruption	Minimal disruption e.g. border screening measures, higher work and school absenteeism	Moderate disruption e.g. quarantine, temperature screening, visitor restrictions at hospitals.	Major disruption e.g. school closures, work from home orders, significant number of deaths.
**Advice to public**	Be socially responsible: if you are sick, stay home	Be socially responsible: if you are sick, stay home	Be socially responsible: if you are sick, stay home	Be socially responsible: if you are sick, stay home
	Maintain good personal hygiene	Maintain good personal hygiene	Maintain good personal hygiene	Maintain good personal hygiene
		Look out for health advisories	Look out for health advisories	Look out for health advisories
			Comply with control measures	Comply with control measures
				Practice social distancing: avoid crowded areas

INFECTION CONTROL IN NON-HEALTHCARE SETTINGS

79. *Guidelines for infection Control in Non-healthcare workplaces* can be found in **Annex D.** Where applicable, MOH will also issue supplementary guidance on infection control and PPE usage to non-healthcare workplaces during a pandemic.

CONCLUSION

80. This document outlines the general principles for responding to a pandemic of a novel or re-emerging respiratory disease under the DORSCON framework. While the timing of a pandemic cannot be predicted, this plan provides an overview of the components for response that will be considered. Given the possible variations in severity of a pandemic, the need for flexibility in the response plan to address different scenarios has been emphasized. There is a need to continue engaging and working with the public to raise the level of preparedness at the individual, community and national levels. Through our collective efforts, we will be ready to implement a robust and sustainable national response to a pandemic threat, should the situation calls for it.

LIST OF ADDITIONAL RESOURCES FOR PANDEMIC PLANNING

Local resources

- Ministry of Health (http://www.moh.gov.sg)
- Ministry of Home Affairs (http://www.mha.gov.sg)
- National Security Coordination Secretariat (http://www.nscs.gov.sg)
- SPRING Singapore (http://www.spring.gov.sg/Resources/Documents/Guidebook_Flu_Pandemics_Business_Continuity_Guide_Eng.pdf)

International resources

- World Health Organisation (http://www.who.int/influenza/preparedness/pandemic/)
- US Centers for Disease Control and Prevention (CDC) (http:// www.cdc.gov/flu/pandemic-resources)

ANNEX A

Influenza viruses

The minimum requirement for pandemic influenza has historically been associated with a major change or shift in the viral surface protein genes in influenza A viruses. Previous influenza pandemics are thought to have originated as swine reassortants, in which one or both human-adapted viral surface proteins were replaced by proteins from avian influenza virus strains. Current evidence of direct human infection with wholly avian influenza viruses suggest that reassortant events can take place directly in humans, without swine as intermediaries.

Avian Influenza

All subtypes of influenza viruses circulate in wild birds and occasionally result in zoonotic infection of humans. At the time of writing, known avian influenza viruses have not adapted well to humans, and the risk of sustained human-to-human transmission and community spread remains low. Nevertheless, the virus continues to persist in birds and often poultry and continues to evolve genetically by mutation, resulting in the possibility that the virus might eventually adapt to humans.

Human infection with H5N1 was first documented in a 1997 outbreak in Hong Kong, with 18 human cases including six deaths. Most human H5N1 infections can be linked to contact with infected poultry or association with exposure to a contaminated environment, but isolated instances of inefficient human-to-human transmission may have occurred.

Human infections with subgroups of H7 influenza viruses (H7N2, H7N3, and H7N7) had been reported in the Netherlands, Italy, Canada, United States of America, Mexico and the United Kingdom. Most infections occurred in association with poultry outbreaks and mainly resulted in conjunctivitis and mild upper respiratory symptoms, with the exception of one death in the Netherlands. Human infections with a new avian influenza A (H7N9) virus were reported in early 2013 in China. Most patients had severe respiratory illness and case fatality was more than 20%. Many reported contact with poultry or poultry-related environments.

Other variant influenza strains

In addition to the direct threat of avian influenza, the 2009 H1N1 pandemic highlighted the emergence of novel influenza strains through reassortment in swine. The strain that emerged in the 2009 pandemic was an avian-human-swine triple reassortment influenza A(H1N1) of classic swine lineage.

In 2010, an influenza A(H3N2) swine-origin triple assortment virus containing the matrix (M) gene from the A(H1N1)pdm09 virus was first identified in pigs in the U.S. Cases of human infection were detected in July 2011. The majority of the infections were associated with exposure to pigs at agricultural fairs, although limited human-to-human transmission had occurred. 16% of cases reported indirect contact with pigs suggesting environmental contamination as a transmission source.

Clinical Presentation

Uncomplicated influenza illness is characterized by the abrupt onset of constitutional and respiratory signs and symptoms (e.g. fever, myalgia, headache, malaise, non-productive cough, sore throat, and rhinitis). Among children, otitis media, nausea, and vomiting are also commonly reported with influenza illness. Influenza illness typically resolves after a limited number of days for the majority of persons, although cough and malaise can persist for more than 2 weeks. Influenza can exacerbate underlying medical conditions (e.g. pulmonary or cardiac disease), lead to primary viral or secondary bacterial pneumonia, or occur as a co-infection with other pathogens. Young children with influenza infection can have initial symptoms mimicking bacterial sepsis with high fevers, and some children hospitalized with influenza can have febrile seizures. Influenza infection has also been associated with encephalopathy, transverse myelitis, Reye syndrome, myositis, myocarditis, and pericarditis.

Infected persons with minimal symptoms may still shed the virus and be infectious. Primary infection in young children is usually symptomatic although up to 50% may be asymptomatic. Adults can remain infectious for 3 to 5 days, while young children can be infectious for up to 3 weeks. Individuals who are severely immuno-compromised may continue to spread the infection for more than 3 weeks.

Pre-existing antibodies against related influenza strains may be present if the pandemic strain is related to previous seasonal influenza strains (e.g. during the H1N1pdm2009). Such pre-existing antibodies may be partially protective, i.e. a higher infective inoculum is required with a lower likelihood of clinical illness.

Influenza Severity

In general, adults aged over 65 years, children younger than five years and persons with certain chronic medical conditions are at higher risk of influenza-associated hospitalization. Local data suggest that the estimated annual excess rate of hospitalization in Singapore due to pneumonia and influenza annually was 78.6 per 100,000 population for children aged 6 to 23 months, and 72.4 per 100,000 population for those aged 24 to 59 months[4]. Older adults typically account for $\geq$90% of influenza-associated deaths annually[5]. Estimated influenza-associated excess mortality rate in Singapore per 100,000 persons were 0.8 among persons aged 20-64 years and 46.9 among persons aged 65 years and older.[6]

[4] Unpublished data for 1996 to 2005 from MOH

[5] Thompson WW, Shay DK, Weintraub E, Brammer L, Cox N, Anderson LJ, et al. Mortality associated with influenza and respiratory syncytial virus in the United States. JAMA. 2003;289:179–86.

[6] Chow A, Ma S, Ling AE, Chew SK. Influenza-associated deaths in tropical Singapore. Emerg Infect Dis. 2006 Jan;12(1):114-21.

ANNEX B

Coronaviruses

Coronaviruses are RNA viruses that can cause respiratory tract or enteric infections in a variety of animals, including humans, livestock and pets. Coronaviruses primarily infect the upper respiratory and gastrointestinal tract of mammals and birds, and five different currently known strains infect humans. Most coronavirus infections in humans result in mild, self-limiting illness.

One commonly known human coronavirus, Severe Acute Respiratory Syndrome-associated Coronavirus (SARS-CoV) that causes SARS has a unique pathogenesis because it causes both upper and lower respiratory tract infections and can also cause gastroenteritis. The SARS outbreak in 2003 affected over 8,000 people across three continents with a case fatality ratio of about 10%, indicating the potential of an animal coronavirus to cross species and transmit from person to person causing severe illness.

For most cases, SARS began with a high fever (>38.0). Other symptoms included headache, a general feeling of discomfort and body aches, while some had mild respiratory symptoms e.g. cough. About 10 to 20 percent of patients had diarrhea although most patients developed pneumonia. SARS was fatal in about 10% of cases.

Similarly, the Middle East Respiratory Syndrome Coronavirus (MERS-CoV), which was first reported in Saudi Arabia in 2012, is a viral respiratory illness. Most people who were confirmed with MERS-CoV infection developed severe acute respiratory illness. They had symptoms of fever, cough and shortness of breath.

ANNEX C

Planning Assumptions

The following planning assumptions are provided to guide planners in developing their preparedness and response plans:

a. The first local human case is more likely to be imported from affected countries rather than developing from within Singapore through direct animal to human transmission.

b. The warning period will be relatively short should a novel pathogen emerge that is capable of efficient human transmission.

c. It may take several days to confirm that this is a new pandemic strain.

d. The disease could be present in Singapore through imported human cases within days to weeks after it emerges in another part of the world. It is unlikely that we will be able to prevent import of the disease. We could attempt to contain the spread of the disease in the community. If containment is not possible, the spread can be delayed.

e. The length of each local epidemic is assumed to be six weeks. The pandemic pathogen may continue to circulate at lower levels after the pandemic.

f. A second pandemic wave of another six weeks is possible, and may be more or less severe than the first wave.

g. There will not be any vaccine initially. The development of vaccine will take at least 4 to 6 months. When vaccines are eventually developed, the supply would be limited initially.

h. Pandemic preparedness and response measures will be guided by the severity of the pandemic.

ANNEX D

Guidelines for Infection Control in Non-Healthcare Workplaces (revised April 2014)

1. This document provides guidance on prevention and control practices that can be implemented in the workplace to limit the spread of severe respiratory infections which are transmitted via close contact and large respiratory droplets. These guidelines can be incorporated into the existing business continuity plans of companies for ease of operations in a public health emergency. However, please bear in mind that no two outbreaks are ever alike, and flexibility in response is important. **The Ministry of Health (MOH) will review the measures and update the current guidelines with directives and advisories, where appropriate, based on the specific transmission characteristics of the infectious disease.**

2. It is important for companies to note that the measures outlined here may be activated at any DORSCON alert levels in responding to a pandemic disease threat, depending on the public health impact. Therefore, specific measures should not be hardwired to any particular DORSCON alert levels.

I. BASIC PRECAUTIONS

3. All employees should be encouraged to practice good personal hygiene to minimise potential transmission of respiratory infections like influenza at the workplace at all times. Some of the actions and measures that employees can do include:

- Be aware of the symptoms of the disease and how it is transmitted.
- Maintain good personal hygiene:
    - Do not spit on the floor or ground
    - Wash hands
        - Regularly and thoroughly with soap and water
        - Before and after preparing food
        - After going to the toilet
        - Before and after eating
        - After blowing their nose
        - After coughing and sneezing
        - After removing personal protective equipment (PPE)
    - Sneezing and coughing into tissues which should be then be carefully disposed of.
- Be responsible for cleanliness of own workspace.

4. In addition, the following actions should be highlighted to staff for their compliance during a pandemic.

- Comply with health and travel advisories, company and staff directives issued (e.g. to monitor one's temperature, health)
- Practice social distancing measures at work as recommended by the workplace management. Where recommended, employees should also comply with social distancing measures outside the workplace (e.g. avoid crowded places and large gatherings and curtail social activities such as social visiting).

- Comply with further directions on use of PPE and other hygiene measures to avoid cross contamination, especially if employees are tasked to carry out public health measures, e.g. symptom/temperature screening and contact tracing.

II. ENVIRONMENTAL CLEANLINESS

5. Workplaces should maintain environmental cleanliness at all times to minimise transmission of infectious diseases which can be transmitted via close contact and respiratory droplets and through contaminated environmental surfaces, e.g. influenza. Infectious agents such as influenza viruses may live up to 2 days on contaminated non-porous, hard surfaces (depending on the humidity and temperature).

6. **General cleaning of Work Area**

(a) All office space, common facilities e.g. toilets, conference rooms, multi-purpose halls etc should be cleaned daily.
(b) Clean all surfaces, frequently touched surfaces and floors daily with a disinfectant, e.g. bleach (diluted to 1% concentration or 1000 ppm).
(c) Alcohol (e.g. isopropyl 70%, ethyl alcohol 60%) can be used to wipe down surfaces where use of bleach is not suitable e.g. metal.

During a Pandemic

7. As pandemic diseases have different public health impact and virulence, additional cleaning may be necessary to prevent transmission of **more severe pandemic diseases** (e.g. SARS and human cases of avian influenza) via contaminated environmental surfaces. Additional cleaning measures for certain areas should be carried out where a suspected or confirmed case has been in. For **milder pandemic diseases such as influenza A/H1N1**, general cleaning guidelines in para 6 would suffice for effective environmental cleaning.

8. **Additional cleaning guidelines for areas exposed to a suspected/confirmed case during a severe pandemic (i.e. disease of high virulence and public health impact, for example, SARS-like disease)**

(a) When a suspected case was in the premises, the management should seal (where possible) the areas where the person has been. Open the door and windows to the affected areas (if possible) and leave the areas undisturbed for at least 8 hours. Cleaning and disinfection should be carried out after the area has been aired. There is no need for airing and special cleaning of other areas. Routine cleaning of these other areas can be carried out without additional PPE than what is usually used.

(b) When cleaning areas where a suspected case has been, cleaning crews should:

i) Wear disposable gloves, disposable gowns and an N95 mask. Avoid touching the nose and mouth (goggles may help as it will prevent hands from touching eyes). Gloves should be removed and discarded if they become soiled or damaged and a new pair worn. All other disposable PPE should also be removed and discarded after cleaning activities are completed. Goggles, if used, should be disinfected according to manufacturer's instructions.

ii) Wash their hands with soap and water immediately after the PPE are removed and when cleaning is completed.

iii) Keep cleaning equipment to the minimum.
iv) Open window for ventilation.
v) Mop floor with bleach (1:10 dilution or diluted to 0.5% chlorine concentration or 5000 ppm).
vi) Wipe all frequently touched areas (e.g. doorknobs, armrests, seatbacks, tables, air/light controls, keyboards, switches etc) and lavatory surfaces with chemical disinfectants (use according to manufacturer's instructions) and allowed to air dry. Bleach solution can be used. Alcohol (e.g isopropyl 70% or ethyl alcohol 70%) can be used for surfaces where use of bleach is not suitable.
vii) Wipe down walls up to 3m in height as well as blinds with disinfectant.
viii) Remove curtains for washing.
ix) Disinfect cleaning equipment used in one room before using for other rooms.
x) Disinfect buckets with fresh disinfectant solution or rinse in hot water before filling.
xi) Rinse wiping cloths/mops in disinfectant several times or rinse thoroughly in hot water.
xii) Disinfectants should be applied to surfaces using a damp cloth. They should not be applied to surfaces using a spray pack, as coverage is uncertain and spraying may promote the production of aerosols. The creation of aerosols caused by splashing liquid during cleaning should be avoided. A steady sweeping motion should be used when cleaning either floors or horizontal surfaces to prevent the creation of aerosols or splashing. Cleaning methods that might -aerosolize infectious material, such as the use of compressed air, must not be used.
xiii) Avoid using the room for the following morning or afternoon sessions.

(c) Cleaning crews should be aware of the symptoms and should report to their occupational health service if they develop symptoms.

III. PERSONAL PROTECTIVE EQUIPMENT (PPE)

9. **During a pandemic**, use of **Personal Protective Equipment (PPE)** may be advisable in some situations, e.g. when handling employees who are ill or when carrying out symptom screening or temperature checks for employees or visitors**.** The following guides on the use of PPE (e.g. surgical masks, N95 masks, disposable gloves and gowns) should be observed.

10. When using masks,

(a) Masks are effective if worn according to instructions and properly fitted.

(b) In general, **surgical masks should be worn by employees who are in contact with individuals who are unwell or potentially unwell**. Surgical masks, when worn properly and coupled with other precautionary measures like hand-washing and avoiding close contact, can prevent droplet transmission of influenza and other acute respiratory infections. **Additional guidance on the use of N95 masks will be provided by MOH during a severe pandemic, where necessary in specific situations (e.g. symptom or temperature screening and contact tracing), if the risk of transmission warrants the use of N95 masks to protect individuals.** Users of N95 masks need to undergo a mask fit test (normally carried out by supplier) to ensure proper fit.

(c) Repeated adjusting of mask while wearing can be a cause of infection due to contamination of hands with droplets gathered on the mask.

(d) Mask should be discarded and changed if it becomes physically damaged.

(e) Users should be monitored for dizziness, difficulty in breathing and skin irritation.

(f) The mask should be disposed of together with other biohazard waste

(g) Avoid touching the nose and eyes which can be routes of infection.

- Discard all disposable items in a bag securely sealed and labeled.
- Hands should be washed with soap and water immediately after gloves are removed.

11. **Stockpiling of PPE for Pandemics**

<u>Quantity</u>

(a) **<u>It</u>** is recommended that agencies maintain 3 to 6 month stockpile. However, the actual quantity would depend on agencies' own estimation of the requirements for each staff as this is dependent on their operations. Mask numbers will depend on how people use them – e.g. general rule of thumb in a hospital, once a shift **<u>and</u>** change if soiled.

<u>Type of PPE</u>

(b) In general, most non-healthcare workers will be using surgical masks. This is especially true in pandemics of milder diseases where surgical masks would confer adequate protection.

(c) In specific situations where the pandemic disease is **severe** and/or additional barrier protection is required over a surgical mask (e.g. during SARS-like disease scenarios), MOH may advise the use of N95 masks as an added precaution for staff who may be coming into contact with potentially ill persons (e.g. frontline symptom/temperature screeners and contact tracers). Under these circumstances, the risk of transmission warrants the use of N95 masks for adequate protection of the staff. As N95 masks have to fit-tested, agencies are advised to stockpile a smaller quantity for the relevant staff who may be engaged in these higher-risk activities. Agencies should note that in most other situations, surgical masks (where appropriate) would be sufficient to protect staff within the confines of the agencies.

IV. SYMPTOM AND TEMPERATURE CHECKS FOR EMPLOYEES AND VISITORS DURING A PANDEMIC

12. Businesses may implement regular symptom or temperature checks for employees and visitors and monitor employees for symptoms (in line with advisories from MOH at that point in time). Such measures may be activated when there is a risk of community transmission of the virus to facilitate case detection and reduce the likelihood of disease spread in the workplace. However, such checks are meant to supplement, and not replace, individual monitoring and good personal hygiene.

13. **Key activities related to health monitoring for employees**

- Companies may need to provide employees with thermometers for the individual checks and may include supervisor's verification of the temperature checks if needed.

- Employees who are unwell should be directed to seek medical help promptly. (Please refer to later section on *"Management of an Employee with Symptoms Suggestive of Acute Respiratory Infections"*).

- Employees should also be advised to screen themselves for symptoms before coming to work. Those who are sick should not come to work and comply with staff policy (e.g. mandatory sick leave)

14. **Key activities related to symptom or temperature screening for visitors**

- Symptom or temperature screening for visitors will likely be recommended for pandemic of diseases of high virulence and public health impact. For mild diseases (e.g. influenza A/H1N1), symptom screening may not be necessary for all settings.

- In general, employees carrying out symptom checks (including temperature screening) of visitors should don N95 masks. This should be combined with frequent hand washing, especially after touching bodily secretions and after removing gloves (if worn). Guidance on N95 mask usage will also be provided by MOH during a pandemic, if necessary.

- Record information of all visitors[7], including date and time of visit, name of visitor, IC number, telephone number and the location/meeting room he/she will be going to for contact tracing purpose[8].

- MOH will advise on the symptoms to be looked out for in visitors (e.g. fever, cough or runny nose). Temperatures of visitors should be checked and recorded as per MOH's advisory.

- Anyone with symptoms should not be allowed into the facility. They should also be asked to wear a surgical mask and advised to seek medical assessment/ treatment promptly.

- Disposable ear thermometer covers should be used, if using ear thermometers. Otherwise, it should be disinfected between use (e.g. use of disinfectant wipes). To further minimise body contact, workplaces can use thermal scanners.

V. Monitoring and quarantine of employees with travel history or contact with case during a Pandemic

15. Employees may have had contact with a confirmed case or have travelled to areas reporting cases of novel influenza or respiratory infections. In such instances, the following can be carried out:

- **Self-monitoring of symptoms.** Advise the employee to monitor his/her own health for any flu-like symptoms and to seek medical attention immediately if he/she feels unwell.
- **Voluntary home quarantine.** For a severe pandemic, organizations may elect to advise the employee not to report for work/ go on voluntary home quarantine for 1 incubation period/ 10 days (or period as advised by MOH). This may reduce the spread of disease if the employee subsequently develops symptoms, but has to be

[7] It also applies to contractors, suppliers and others.
[8] Contact tracing will be carried by the authorities as long as operationally feasible.

weighed with the impact on absenteeism caused by voluntary home quarantine as not all exposed individuals become ill. The organisation is to decide on the leave and cover arrangements.

- **In-house phone surveillance.** For a severe pandemic, companies may also check on employee's health status by phone during his/her absence from work. This will facilitate treatment if the employee becomes symptomatic.

16. If required, MOH will contact individuals directly if they have been identified as close contacts of confirmed cases and require **mandatory phone surveillance or quarantine**. Close contacts with unprotected exposure may be given post-exposure prophylaxis as advised by an attending physician. These public health measures are recommended based on MOH's risk assessment. In such instances, no further action will be required on part of the companies and in the workplaces, unless specified by MOH.

17. As the purpose of quarantine is to contain a disease so that transmission is halted, for diseases which are highly transmissible, such as influenza, quarantine may only be effective up to a certain point. MOH will cease home quarantine and phone surveillance measures when it is assessed to be no longer feasible or have an impact on community transmission. Other measures such as social distancing will then be emphasised. Measures such as quarantine and social distancing are also necessary only for pandemic diseases with high virulence and public health impact. As mentioned in para 15, for severe diseases, organisations may wish to continue to implement home quarantine (on a voluntary basis) and in-house phone surveillance to prevent the spread of disease in the workplace.

VI. Management of an Employee with Symptoms Suggestive of Acute Respiratory Infection during a Severe Pandemic (i.e. High Virulence and Public Health Impact)

18. During a severe pandemic, companies and organisations should put in place procedures to manage staff who become ill at work. The procedures should be implemented in line with advisories from MOH. Health advisory will be issued at the point in time on the symptoms of the disease, relevant contact and where applicable, travel history.

19. In the event supervisors or work managers observe or receive a report of an employee who is unwell, the unwell person should be:

- Provided with a surgical mask to wear in order to reduce the amount of droplets coughed into the air. Masks should be changed if they become wet, hard to breathe in, physically damaged or visibly soiled.
- Advised to cover his/her mouth and nose with tissues when coughing or sneezing.
- Isolated and moved to a room or area away from other people.
- Advised to use only the toilet facility designated for him/her (if possible).

20. In addition, the management should keep the number of employees attending to the ill person to a minimum. Staff attending to the ill person should wear, at a minimum, surgical masks or, if MOH has explicitly recommended during a pandemic, N95 masks[9] and disposable gloves.

21. The ill person should seek medical attention promptly. He/She should inform the doctor of relevant contact and/or travel history, if any. If necessary, the GP or polyclinic staff

[9] Please follow manufacturer's instructions on the proper use of N95 masks. N95 masks should be discarded after attending to each person suspected to have pandemic influenza.

will transfer the patient to a designated healthcare facility for further assessment/ admission as appropriate.

22. For emergency situations (e.g. person is unconscious or has difficulty breathing), the 995 emergency ambulance should be activated.

23. To facilitate contact tracing, if necessary, the management may decide to take down the names and contact details (IC number, address, telephone number) of all persons who have come into contact with the employee when he/she was unwell. If the ill employee is confirmed to have the disease, MOH officers will contact the organisation to trace all those who came into contact with him/her. Employees who need to be put on phone surveillance or be quarantined will be contacted and advised accordingly.

24. Cleaning of the areas the suspect case has been to have to be carried out. Please refer to the section on **Environmental Cleaning**.

VII. SOCIAL DISTANCING

25. The spread of respiratory infections is exacerbated by increased social contact, crowded places and large gatherings. To protect employees from being exposed to infectious persons, a strategy called social distancing can be employed to minimise contact with others during a **severe** pandemic. Companies and organisations can implement measures to **increase social distance at work,** should the need arises, in line with MOH's latest advisory. Any measure that achieves this would minimize transmission of the disease.

26. Examples of social distancing measures include:

(a) **Dividing staff into work teams.** Where office workflow permits, organisations can consider dividing their staff into work teams. Each team should, where possible, avoid contact with the other teams.

(b) **Telecommuting.** Similarly, organisations can consider the feasibility of telecommuting and allow their staff to work from home.

(c) **Others.**

- Avoid meeting people face-to-face. Use other means to carry out discussion, business.
- If people have to meet, advise staff to maintain a distance of at least 1 metre (or as advised by MOH) from visitors/ colleagues, if possible. Whenever possible, choose a larger venue or meeting room where is possible to maintain this distance.
- Introduce staggered lunch hour to reduce crowding of staff cafeterias.
- Use of systems where customers/clients can pre-order/ request information via phone, mail/fax and prepare requested items ready for fast pickup or delivery.
- Advise employees to avoid activities even outside the workplace where they may be exposed to infected persons e.g. avoid crowded places and large gatherings, and curtail social activities such as social visiting.

VIII. Annual Influenza Vaccination

27. Companies may wish to encourage their employees to have their annual influenza vaccination. While this does not protect them against pandemic influenza strains, the

seasonal influenza vaccination will still protect employees from circulating viruses and reduce the overall burden of disease.

Summary tables for response measures under the DORSCON matrix

Possible scenarios	Applicable response phases	Border Control	Public health measures						
			**Temperature screening in institutions / buildings**	**Social distancing**	**School closures**	**Contact tracing**	**Phone surveillance or quarantine**	**Antivirals for influenza**	**Vaccination**
**GREEN - Negligible to low public health impact**									
• **High virulence**<br>• **No or limited H-H transmission**<br>• **Disease mainly overseas**	Alert (with containment of imported cases)	Health Advisory Notices (HANs) (Posters, Cards)	No	No	No	Yes, if cases are imported	Consider to implement depending on risk	Treatment of case where necessary	No
• **Similar or lower virulence and transmissibility as seasonal flu**	Mitigation	No	Consider to implement if necessary			No	No		Vaccination for high risk groups, if available
**YELLOW**									
• **High virulence but low transmissibility**<br>• **Disease mainly overseas**	Alert (with containment of imported cases)	HANs<br>Health Declaration Cards (HDCs) and temperature screening of inbound passengers, if necessary	No	No	No	Yes, if cases are imported	Consider to implement depending on risk	Treatment of cases where necessary	Procure and offer vaccine when available
• **Local epidemic with low virulence but high transmissibility**	Mitigation	HANs	Consider to implement if necessary			No	No		
• **High virulence and transmissibility, but vaccine available**	Mitigation		No						Distribute vaccine to mitigate impact
**ORANGE**									
• **High virulence and transmissibility**<br>• **Disease mainly overseas**	Alert	HANs<br>HDCs and temperature screening of inbound passengers, if necessary	No	No	No	Yes, if cases are imported	Quarantine	Treatment of cases, consider limited prophylaxis of personnel providing essential services	Procure and offer vaccine when available
• **High virulence and transmissibility**<br>• **Disease in Singapore**	Containment		Yes, depending on risk	Yes, depending on risk	Yes, selective closures if cases or clusters are detected in schools	Yes, as far as operationally feasible	Quarantine, as far as operationally feasible		
• **High virulence and transmissibility**<br>• **More cases in Singapore**	Limited mitigation	HANs<br>Temperature screening of all passengers. if necessary				No	No		
**RED**									
• **High virulence and transmissibility**<br>• **Widespread transmission**	Mitigation	Temperature screening of all passengers, if necessary	Yes	Yes	Yes	No	No	Treatment of cases and prophylaxis of personnel providing essential services	Procure and offer vaccine when available

@PresidencyZA | www.stateofthenation.gov.za

STAY HOME
SAVE SOUTH AFRICA

Summary of alert levels

ALERT LEVEL 5	ALERT LEVEL 4	ALERT LEVEL 3	ALERT LEVEL 2	ALERT LEVEL 1
**OBJECTIVE**				
Drastic measures to contain the spread of the virus and save lives.	Extreme precautions to limit community transmission and outbreaks, while allowing some activity to resume.	Restrictions on many activities, including at workplaces and socially, to address a high risk of transmission.	Physical distancing and restrictions on leisure and social activities to prevent a resurgence of the virus.	Most normal activity can resume, with precautions and health guidelines followed at all times. Population prepared for an increase in alert levels if necessary.

WHATSAPP SUPPORT
0600 123 456
EMERGENCY NUMBER
0800 029 999
sacoronavirus.co.za

REPUBLIC OF SOUTH AFRICA

2030 NDP

@PresidencyZA | www.stateofthenation.gov.za

STAY HOME

SAVE SOUTH AFRICA

How the alert system works

The **level of alert** at any given time will be decided based on the **rate of transmission,** as well as the capacity of the health system to provide care to those infected.

Different **parts of the country** may be at **different levels** simultaneously.

It is possible to **move up and down levels,** as well as to skip levels if necessary.

Essential goods and services will continue to be available at all levels.

Some precautions will **remain in place at all levels,** including **social distancing** guidelines and **safe hygiene** practices.

WHATSAPP SUPPORT
0600 123 456
EMERGENCY NUMBER
0800 029 999
sacoronavirus.co.za

REPUBLIC OF SOUTH AFRICA

2030 NDP

@PresidencyZA | www.stateofthenation.gov.za

STAY HOME
SAVE SOUTH AFRICA

A phased easing of the lockdown

As the full national lockdown ends, South Africa will **shift to a system of alert levels** at the provincial and, in some cases, the district level.

Clear criteria based on the rate of infection as well as **health system capacity will be used to determine the alert level** in each area. Where the alert level is lower, economic activity can resume more quickly.

The National Command Council will **review the alert level at each meeting,** and will impose a lower or higher level as necessary.

Many **restrictions on public life and gatherings,** as well as higher-risk activities, **will remain** regardless of the alert level.

WHATSAPP SUPPORT
0600 123 456
EMERGENCY NUMBER
0800 029 999
sacoronavirus.co.za

REPUBLIC OF SOUTH AFRICA

2030 NDP

STAY HOME
SAVE SOUTH AFRICA

A phased easing of the lockdown

As the full national lockdown ends, South Africa will **shift to a system of alert levels** at the provincial and, in some cases, the district level.

Clear criteria based on the rate of infection as well as **health system capacity will be used to determine the alert level** in each area. Where the alert level is lower, economic activity can resume more quickly.

The National Command Council will **review the alert level at each meeting,** and will impose a lower or higher level as necessary.

Many **restrictions on public life and gatherings,** as well as higher-risk activities, **will remain** regardless of the alert level.

@PresidencyZA | www.stateofthenation.gov.za

STAY HOME

SAVE SOUTH AFRICA

Alert levels will be determined for each province and district

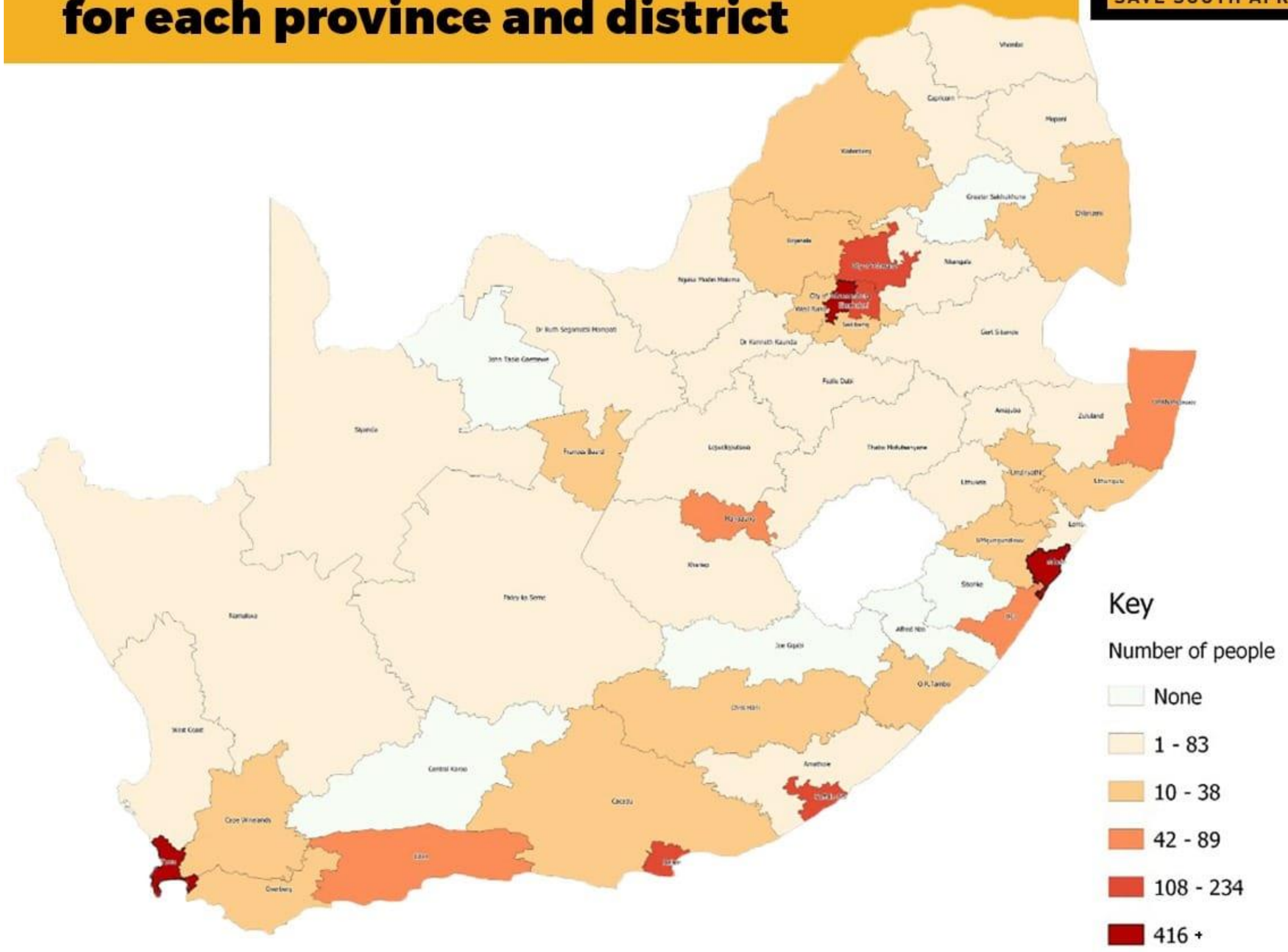

WHATSAPP SUPPORT
0600 123 456
EMERGENCY NUMBER
0800 029 999
sacoronavirus.co.za

REPUBLIC OF SOUTH AFRICA

2030 NDP

STAY HOME
SAVE SOUTH AFRICA

The following restrictions will remain in place

PROHIBITED
Bars and shebeens

PROHIBITED
Conference and convention centres

PROHIBITED
Entertainment venues, including cinemas, theatres, and concerts

PROHIBITED
Sporting events

PROHIBITED
Religious, cultural and social gatherings

The Wayback Machine - https://web.archive.org/web/20210323091135/https://www.gov.za/covid-19/ab...

About alert system

The five-level COVID-19 alert system has been introduced to manage the gradual easing of the lockdown.

This risk-adjusted approach is guided by several criteria, including the level of infections and rate of transmission, the capacity of health facilities, the extent of the implementation of public health interventions and the economic and social impact of continued restrictions.

The country is on adjusted alert level 1 from 1 March 2021.

The country was on adjusted alert level 3 from 29 December 2020 to 28 February 2021.

The country was on alert level 1 from 21 September to 28 December 2020.

The country was on alert level 2 from 00h01 on 18 August 2020.

The country was on alert level 3 from 1 June to 17 August 2020.

The country was on alert level 4 from 1 to 31 May 2020.

The country went into lockdown from midnight 26 March to 30 April 2020. (Alert level 5)

Criteria for determination of alert levels

Alert levels determine the level of restrictions to be applied during the national state of disaster.
(a) 'Alert Level 1' indicates a low Covid-19 spread with a high health system readiness;
(b) 'Alert Level 2' indicates a moderate Covid-19 spread with a high health system readiness;
(c) 'Alert Level 3' indicates a moderate Covid-19 spread with a moderate health system readiness;
(d) 'Alert Level 4' indicates a moderate to a high Covid-19 spread with a low to moderate health system readiness;
(e) 'Alert Level 5' indicates a high Covid-19 spread with a low health system readiness.

The Ministerial Advisory Committee must advise the Minister of Health regarding which Alert Level should be declared nationally, provincially, in a metropolitan area, or a district, when taking into account
(a) the epidemiological trends of Covid-19 infections;
(b) the health system capacity in a specified area to respond to the disease burden; and
(c) any other factor that would influence the level of infection, hospitalisation and mortality.

Epidemiological trends includes a consideration of the trends in the number of tests done, number of persons screened, number of positive cases, number of recoveries and the demographic profile of the positive cases.

Health system capacity includes a consideration of the number of facilities available to support Covid-19, bed-occupancy levels for the various levels of care, human resource capacity, equipment and related resources.

(Gazette 43599, 7 August 2020)

Alert levels summary

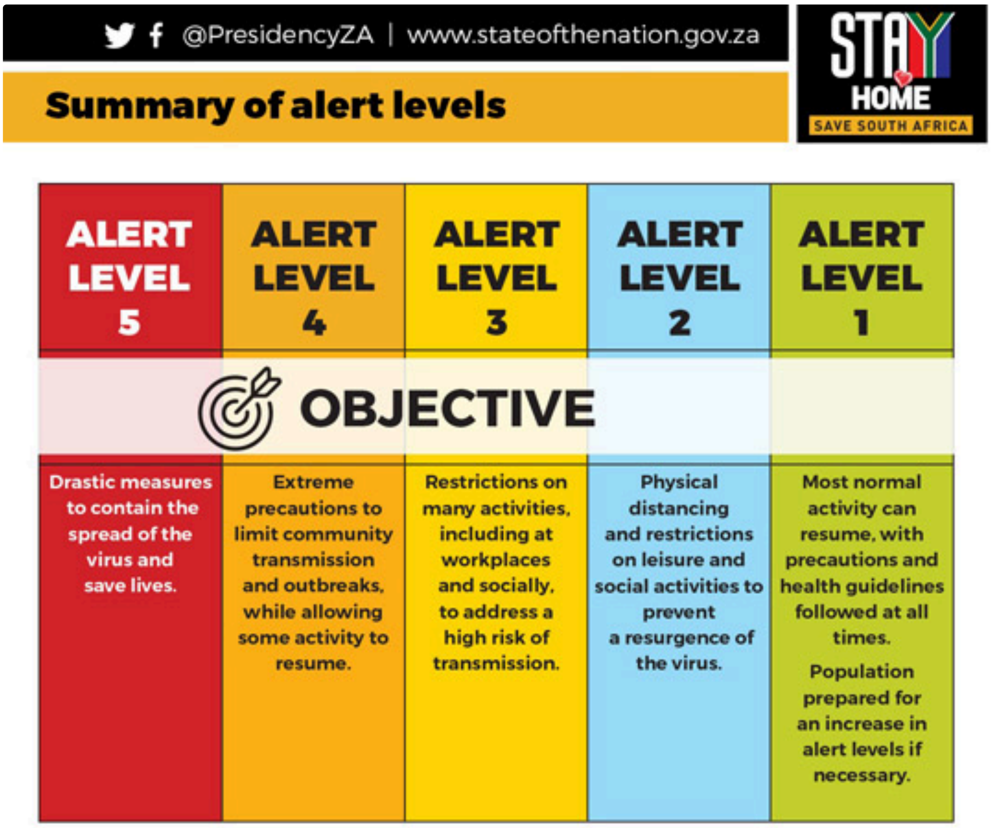

WHATSAPP SUPPORT 0600 123 456
EMERGENCY NUMBER 0800 029 999
sacoronavirus.co.za

REPUBLIC OF SOUTH AFRICA

2030 NDP

Related information:

Coronavirus / COVID-19

EXPLORE GOV.ZA

- Statements
- Documents
- Events
- Key issues
- Government opinion pieces
- About government
- About South Africa
- Contact your government
- Feedback
- Sitemap
- Get e-mail updates
- Services for residents
- Services for organisations
- Services for foreign nationals
- FAQs
- Terms and conditions
- RSS feeds

Copyrig
2021 G
of Sout
All Righ
Reserve

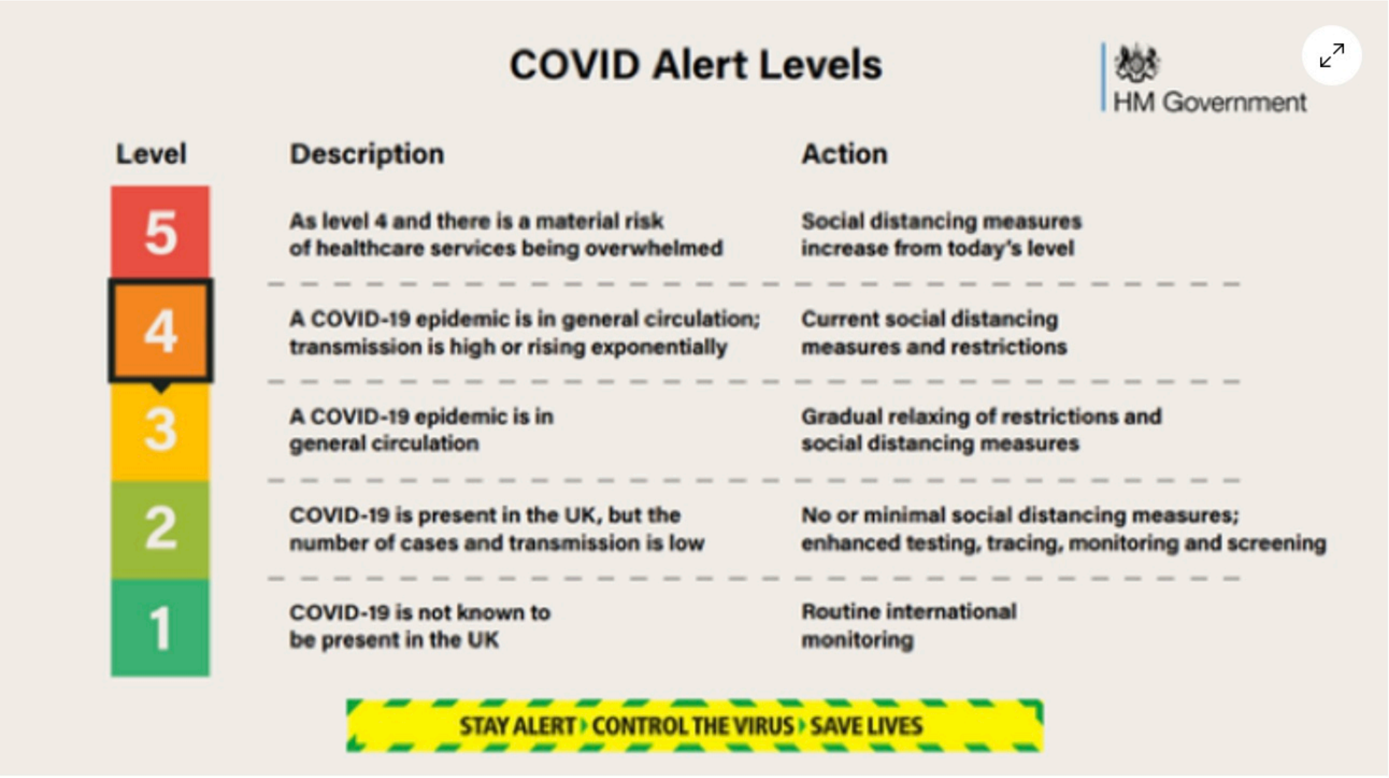

Fig. 1. New UK COVID Alert Levels (UK Government) as of 10th May 2020.

GOV.UK

UK Health
Security
Agency

Guidance

UK COVID-19 alert level methodology: an overview

Updated 29 March 2023

Contents

Introduction

Indicators for escalation and de-escalation of UK COVID-19 alert levels

© Crown copyright 2023

This publication is licensed under the terms of the Open Government Licence v3.0 except where otherwise stated. To view this licence, visit nationalarchives.gov.uk/doc/open-government-licence/version/3 or write to the Information Policy Team, The National Archives, Kew, London TW9 4DU, or email: psi@nationalarchives.gov.uk.

Where we have identified any third party copyright information you will need to obtain permission from the copyright holders concerned.

This publication is available at https://www.gov.uk/government/publications/uk-covid-19-alert-level-methodology-an-overview/uk-covid-19-alert-level-methodology-an-overview

> As of 29 March 2023, the UK COVID-19 alert level system has been suspended.
>
> The suspension of the system reflects the transition to Living with COVID-19 (https://www.gov.uk/government/publications/covid-19-response-living-with-covid-19/covid-19-response-living-with-covid-19), which is thanks to the ongoing success of the vaccination programme and the availability of treatments for those who need them.
>
> The UK Health Security Agency (UKHSA) continues to track the latest COVID-19 epidemiology through numerous surveillance systems, including the weekly National flu and COVID-19 surveillance reports (https://www.gov.uk/government/statistics/national-flu-and-covid-19-surveillance-reports-2022-to-2023-season).

Introduction

One of the key objectives of the UK Health Security Agency (UKHSA) is to provide advice to the UK chief medical officers (CMOs) who in turn advise ministers on the UK coronavirus (COVID-19) alert level. The alert levels were originally defined in the UK government's COVID-19 recovery strategy 'Our Plan to Rebuild' (https://www.gov.uk/government/publications/our-plan-to-rebuild-the-uk-governments-covid-19-recovery-strategy) in May 2020 (most recently revised in August 2022) and communicate the current risk at a UK-wide level.

The alert levels are:

- level 1: COVID-19 is present in the UK, but the number of cases and transmission is low
- level 2: COVID-19 is in general circulation in the UK, but direct COVID-19 healthcare pressures are low and transmission is declining or stable
- level 3: COVID-19 is in general circulation in the UK
- level 4: COVID-19 is in general circulation in the UK; transmission is high and direct COVID-19 pressure on healthcare services is widespread and substantial or rising
- level 5: as level 4 and there is a material risk of healthcare services being directly overwhelmed by COVID-19

This document outlines the criteria used by UKHSA as it considers its recommendation for the CMOs. The initial methodology was developed following consultation with national public health experts, reviewed and

informed by the Scientific Advisory Group for Emergencies (SAGE) and agreed by the UK's CMOs.

The methodology will evolve as UKHSA learns from current operations and as information streams develop. It is therefore subject to future review by the UKHSA Technical Board, chaired on a rotating basis by the 4 UK CMOs.

Approach

UKHSA's approach is focused on the criteria to move between levels, rather than criteria that define an individual level. The indicators presented below are considered in the context of a range of measures. Determining the alert level is not an automated or purely statistical process. The approach aims to blend expert judgement and risk assessment with the more quantifiable indicators and thresholds outlined below. This informs an overall assessment of the situation and an alert level recommendation to CMOs.

The UK COVID-19 alert level is focused on data that directly relates to COVID-19 impacts. The one exception is the move between level 4 and level 5, where the UKHSA and CMOs consider all source pressures as this informs the probability of healthcare services being overwhelmed.

Our operational aim is to avoid unnecessary, unpredictable or frequent changes to the alert level, account for the nationwide complexity of the epidemic and reflect our evolving understanding of COVID-19 and its transmission.

Timing

It will sometimes be necessary to escalate the alert level as rapidly as possible, to signal an urgent, escalating national public health crisis. Conversely, as the risk posed by COVID-19 drops, it will be important to ensure changes to the alert level are undertaken in a stable fashion and that a long-term downward trend in new infections has been established. As a guiding principle, UKHSA will ensure that most, if not all, indicators have been met when recommending to CMOs a reduction in the alert level.

Following any recommendation to de-escalate, a minimum of 4 weeks is allowed before any subsequent recommendation to de-escalate further. During the 4 weeks immediately following a de-escalation, epidemic trajectory will continue to be monitored and a recommendation to escalate the alert level may still be made during this time if required.

Indicators for escalation and de-escalation of UK COVID-19 alert levels

A range of indicators and thresholds are used to support the underpinning analysis for alert level recommendations. A recommendation to raise the alert level is most likely to be made based on a combination of the indicators described for each threshold. The main exception to this is the risk posed by the emergence or spread of a new variant. Should a new variant be more transmissible or have vaccine escape characteristics it could be highly likely to lead to changes in the national epidemiology in the short-term. As a result, in exceptional circumstances a change to the alert level could be recommended based on the risk posed by the emergence of a new variant alone.

To avoid unnecessary, unpredictable or frequent changes to the alert level, at least 4 weeks of consecutive decline in important metrics would be required before considering de-escalation between levels.

Escalation and de-escalation indicators and thresholds for each alert level are listed below.

Alert level 1

Indicator

There is a single holding indicator, with recommendation for level 1 only possible if that indicator is met:

- Are current COVID-19 cases and transmission sufficiently low to support being at level 1?

Rationale

A recommendation to de-escalate to alert level 1 would require consensus between UK CMOs and UKHSA that case numbers and UK transmission of COVID-19 are sufficiently low to pose minimal direct acute threat to the UK population. Evidence will be considered using information provided by health protection and central surveillance teams in each UK nation and other sources as appropriate. Should the above indicator not be met, the alert level recommendation would be escalate to, or remain at, level 2.

Escalating from level 2 to level 3

Indicators

- Is the national R reliably estimated to be ≥1?
- Are case numbers and transmission persistently rising?
- Are direct COVID-19 healthcare pressures rising?
- Is current direct COVID-19 absolute healthcare pressure sufficiently high to support escalation to level 3?

Rationale

At this level many statistical or mathematical measures, such as R, are unlikely to be reliable. Weekly confirmed case rates and other markers of infection burden dependent on testing will be considered alongside representative surveillance measures such as the ONS community infection survey positivity estimates. At all levels of the alert system, the absolute level of direct healthcare pressures will be compared with previous phases of the pandemic and considered alongside contextual factors like disease severity, health inequalities, population immunity (including vaccination uptake) and likely upcoming behavioural changes.

Sources include laboratory test results, public health surveillance systems and modelling from SPI-M/SAGE.

Escalating from level 3 to level 4

Indicators

- Is the national R reliably estimated to be R>1?
- Is the doubling time of confirmed new infections less than 7 days?
- Are there more than 30,000 estimated new infections in the UK per day?
- Are COVID-19 related hospital admissions increasing at ≥25% over the same 7-day period?
- Is COVID-19 related hospital occupancy increasing at ≥25% over the same 7-day period?
- Are COVID-19 related high dependency units (HDU) or intensive care units (ICU) admissions and/or occupancy increasing?
- Are new daily COVID-19 related deaths increasing?
- Is current direct COVID-19 absolute healthcare pressure sufficiently high to support escalation to level 4?

Rationale

Hospital activity and severe health outcomes are the key indicators representing healthcare pressures, but these will be subject to a lag from the point of infection. Hence the inclusion of transmission dynamics, doubling time and estimated incidence.

Sources include laboratory test results, hospital admissions and death data (available on the GOV.UK dashboard (https://coronavirus.data.gov.uk/)). Estimated new infections will be informed by a range of sources, including survey data (for example Office for National Statistics (ONS) and ZOE/KCL) and mathematical modelling provided by SPI-M/SAGE.

Alert level 5

Indicator

There is a single holding indicator, with level 5 only recommended if that indicator is met:

- Has UKHSA, in consultation with NHS senior leadership and CMOs, estimated that forecasted healthcare demand will outmatch forecasted capacity across the UK, regions or devolved administrations within the next 21 days?

Should the above indicator not be met, de-escalation to, or remaining at, level 4 can be considered. This ensures that the focus remains on NHS capacity and operational pressures when COVID-19 infections are at high levels.

Rationale

A recommendation to escalate to COVID-19 alert level 5 should be made in consultation with health service directors and contingency planners, and should be based around their predicted capacity, which includes surge capacity and mutual aid. In principle, escalation to level 5 should allow sufficient time for the implementation of urgent national measures to protect healthcare services from being overwhelmed.

De-escalating from level 4 to level 3

Indicators

- Is the national R reliably estimated to be <1?

- Are there estimated to be less than 30,000 new infections per day?
- Have new daily COVID-19 confirmed infections been on a downward trend, or stable at a low level, for at least 4 weeks?
- Have COVID-19 related hospital admissions been on a downward trend, or stable at a low level, for at least 4 weeks?
- Has COVID-19 related hospital occupancy been on a downward trend, or stable at a low level, for at least 4 weeks?
- Have COVID-19 related HDU or ICU admissions and/or occupancy been on a downward trend, or stable at a low level, for at least 4 weeks?
- Have new daily COVID-19 related deaths been on a downward trend, or stable at a low level, for at least 4 weeks?
- Is current direct COVID-19 absolute healthcare pressure sufficiently low to support de-escalation to level 3?

Rationale

Estimated transmission dynamics of R<1 for a sustained period, combined with a demonstrable reduction in the number of people becoming severely unwell and/or dying, would give confidence that this relatively high degree of risk is receding.

Sources include laboratory test results, hospital admissions and death data (available on the GOV.UK dashboard (https://coronavirus.data.gov.uk/)), survey data (for example ONS and ZOE/KCL), and mathematical modelling provided by SPI-M/SAGE.

De-escalating from level 3 to 2

Indicators

- Is the national R reliably estimated to be <1?
- Are cases and transmission falling?
- Are direct COVID-19 healthcare pressures falling?
- Is current direct COVID-19 absolute healthcare pressure sufficiently low to support de-escalation to level 2?

Rationale

Sources include laboratory test results, hospital admissions and death data (available on the GOV.UK dashboard (https://coronavirus.data.gov.uk/)), mathematical modelling provided by SPI-M/SAGE, and public health surveillance systems.

OGL

All content is available under the Open Government Licence v3.0, except where otherwise stated © Crown copyright

View this email in web browser

EPIC exchange

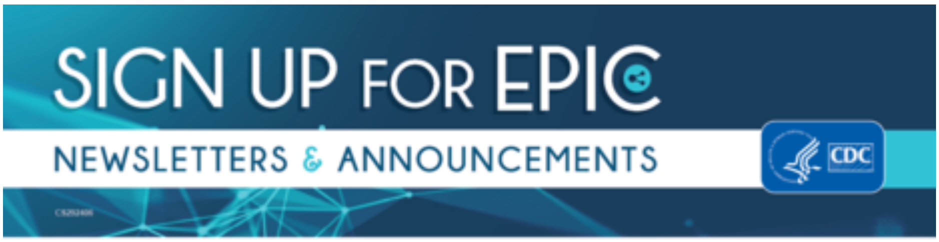

Please share this resource with your colleagues and networks. Visit CDC Emergency Preparedness & Response for more information.

CDC recently launched COVID-19 Community Levels, a new tool for helping people and communities decide on prevention steps based on the latest data.

Some people, such as our oldest citizens, people with weakened immune systems, and people with disabilities, are more likely to get very sick from COVID-19 and may need to take extra steps to protect themselves. Knowing COVID-19 Community Levels can help everyone make choices to protect themselves and others.

COVID-19 Community Levels Tool

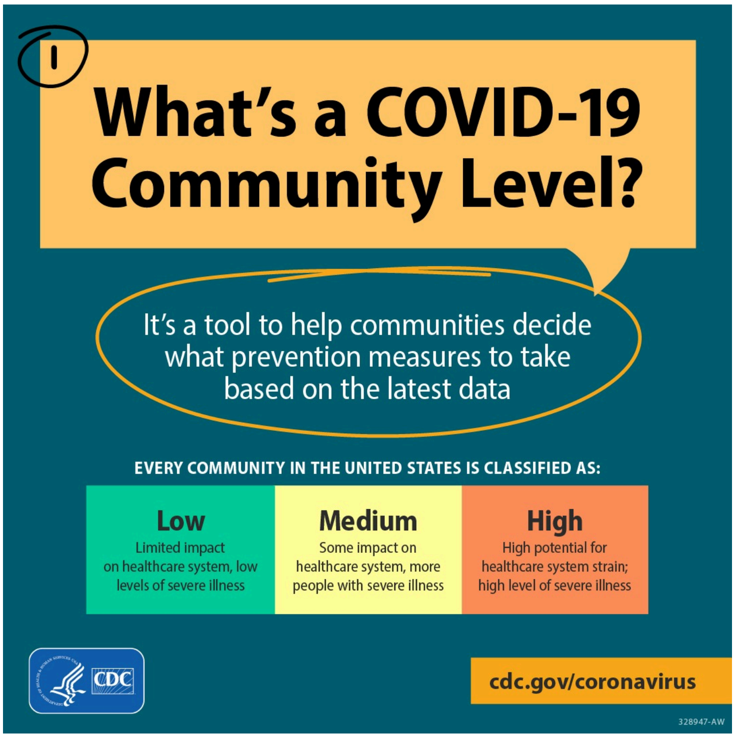

It's easy to check your county's COVID-19 level on CDC's website and find out which COVID-19 prevention measures to use for individuals and communities.

There are three levels (Low, Medium, High), which are determined by looking at new hospital admissions for people with COVID-19; hospital beds being used by patients with COVID-19; and the total number of new COVID-19 cases in your community.

No matter what your COVID-19 Community Level, layered prevention strategies can help protect you and those around you, including someone at increased risk for severe COVID-19.

Recommendations based on COVID-19 Community Levels may not apply to healthcare settings such as hospitals or long-term care facilities.

Learn more about COVID-19 Community Levels.

Prevention Strategies for Those at Increased Risk for Severe COVID-19

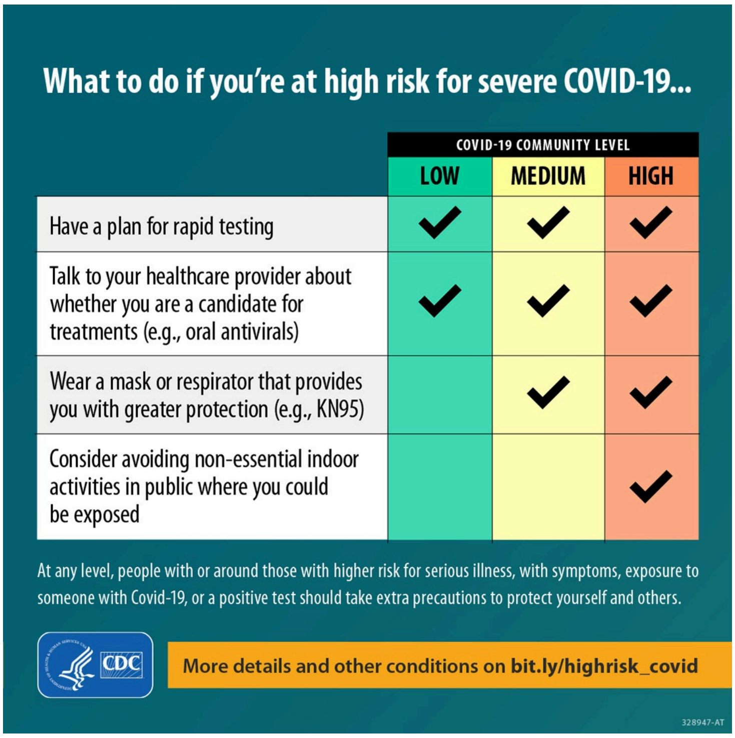
What to do if you're at high risk for severe COVID-19...

	COVID-19 COMMUNITY LEVEL		
	LOW	MEDIUM	HIGH
Have a plan for rapid testing	✓	✓	✓
Talk to your healthcare provider about whether you are a candidate for treatments (e.g., oral antivirals)	✓	✓	✓
Wear a mask or respirator that provides you with greater protection (e.g., KN95)		✓	✓
Consider avoiding non-essential indoor activities in public where you could be exposed			✓

At any level, people with or around those with higher risk for serious illness, with symptoms, exposure to someone with Covid-19, or a positive test should take extra precautions to protect yourself and others.

CDC More details and other conditions on bit.ly/highrisk_covid

328947-AT

Checking COVID-19 Community Levels can help you decide what steps to take to protect yourself and others. If you are at increased risk for severe illness from COVID-19, make sure you're up to date on your COVID-19 vaccines. In areas with medium or high COVID-19 Community Levels, wear a well-fitting mask indoors. If someone you visit with is at increased risk, take a rapid test right before you go. If the visit is indoors, make sure ventilation is good.

If you are at increased risk of getting very sick from COVID-19, treatments may be available. Get tested as soon as possible after symptoms start. Contact your healthcare provider right away if your result is positive. Treatment must be started early to work.

If your area is identified as a Low, Medium, or High COVID-19 Community Level, CDC's recommendations can help you decide what prevention strategies you can use, based on the level in your area and your own risk of severe illness.

Recommendations for people in Low Community Level:

- Stay up to date with COVID-19 vaccines.
- Get tested if you have symptoms.
- Follow CDC recommendations for isolation and quarantine.
- If you have a weakened immune system or high risk for severe illness, have a plan for testing if you need it, and talk to your healthcare provider about treatment options that are right for you.

Recommendations for people in Medium Community Level:
Follow recommendations for people in low levels, plus:

- If you are at high risk for severe illness, talk to your healthcare provider about whether you need to wear a mask and take other precautions (such as testing).
- If you live with someone at high risk or if you will visit someone at high risk, consider self-testing before contact, and consider wearing a mask when indoors with them.

Recommendations for people in High Community Level:

Follow recommendations for people in low and medium levels, plus:

- Wear a well-fitting mask indoors in public.
- If you have a weakened immune system or are at high risk for severe illness, wear a mask or respirator that provides more protection, and consider avoiding non-essential indoor activities in public.

See the full list of recommendations by COVID-19 Community Level.

At all levels, people can wear a mask based on personal preference, informed by their personal level of risk. People with symptoms, a positive test, or exposure to someone with COVID-19 should wear a mask.

Indicators for Monitoring COVID-19 Community Levels and COVID-19 and Implementing COVID-19 Prevention Strategies

Accessible Version: https://www.cdc.gov/coronavirus/2019-ncov/science/community-levels.html

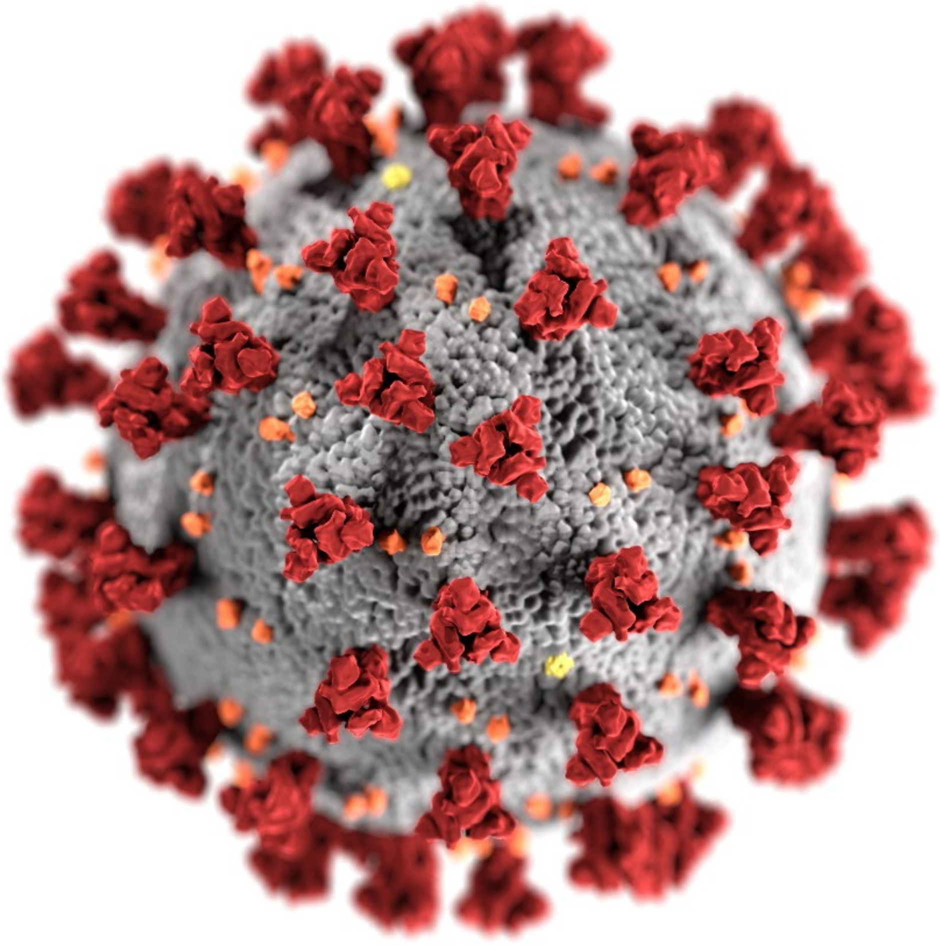

Overview and Scientific Rationale

February 25, 2022

cdc.gov/coronavirus

Why refocus efforts for monitoring COVID-19 in communities?

- **Shift from eliminating SARS-CoV-2 transmission towards more relevant metrics given current levels of population immunity and tools available**
- **Current high levels of population immunity reduce risk of severe outcomes**
  - High rates of vaccination in population as a whole
  - Availability of boosters, and booster coverage among populations at high risk
  - In unvaccinated, high rates of infection-induced protection
- **Breadth of tools available for public health and clinical care**
  - Broad access to vaccines, therapeutics, testing

- **Community measures should focus on minimizing the impact of severe COVID-19 illness on health and society**
  - Preventing medically significant illness
  - Minimizing burden on the healthcare system
  - Protecting the most vulnerable through vaccines, therapeutics, and COVID-19 prevention

CDC's Indicators of Community Transmission

Indicator	Low Transmission	Moderate Transmission	Substantial Transmission	High Transmission
Total new cases per 100,000 persons in the past 7 days	0-9	10-49	50-99	≥100
Percentage of Nucleic Acid Amplification Test results that are positive during the past 7 days	<5.0%	5.0%-7.9%	8.0%-9.9%	≥10.0%

- First released in September 2020
- Relied on two metrics to define community transmission: Total new cases per 100,000 persons in the past 7 days, and percentage of Nucleic Acid Amplification Test results that are positive during the past 7 days
- Used by CDC to inform setting-specific guidance and layered prevention strategies (e.g., screening testing in schools, masking, etc.)
- Public health practitioners, schools, businesses, and community organizations also rely on these metrics to inform decisions about prevention measures

The current state of the pandemic requires a refined approach to monitoring COVID-19

- Community transmission indicators were developed in fall 2020 (prior to availability of vaccines) and reflect goal of limiting transmission in anticipation of vaccines being available

- **Neither of the community transmission indicators reflects medically significant disease or healthcare strain**

- Community transmission levels are largely driven by case incidence, which does not differentiate mild and severe disease

Criteria for Selecting Community Indicators

- Indicators had to meet several criteria:
  1. Data available at the county level or allocated to county level from health service areas
  2. Data source provides nation-wide coverage
  3. Data reflect intended goals of emphasizing medically significant disease and healthcare strain
  4. Data reported at least weekly (or more often) with sufficient timeliness to allow data to inform decisions about prevention measures

Selecting COVID-19 Community Indicators

- Criteria were established to assess potential candidate indicators
- Review of historical data from 18 months of the pandemic
  - Compiled available indicators across data systems
  - Assessed trends in increases and declines in cases, hospital capacity, other indicators
  - Reviewed historical data and thresholds used in COVID-19 Community Profile Report | HealthData.gov and State Profile Report
- Assessed candidate indicators against criteria and eliminated those that did not fully meet established criteria
  - Deaths, while an important metric, are a lagging indicator and have low numbers which result in unstable estimates at local levels
  - Emergency Department visits from the National Syndromic Surveillance Program are a promising indicator, but include 71% of emergency departments, so do not have national coverage

Final Selection of COVID-19 Community Indicators

- Narrowed the list of candidate indicators based on criteria:
  - *New hospital admissions with confirmed COVID-19/100,000 people* and *percent of inpatient beds occupied with COVID-19 patients* selected as best candidates
  - ICU beds occupied, new hospital admissions/100 beds, test positivity, and metrics reflecting percent change (e.g., in new admissions, new cases) eliminated
  - New cases retained as a potential candidate to assess performance as leading indicator

Establishing Thresholds for COVID-19 Community Levels

- Used correlation analyses and thresholds from Community Profile Reports and State Profile Reports to assess potential thresholds
- Correlations indicate:
  - 100 cases/100,000 population per week corresponds to about 3-4% of COVID-19 inpatient bed utilization, 6-10 new admissions/100,000 population
  - Inpatient bed occupancy is about half that of ICU occupancy
  - Fewer new admissions, fewer admissions per case, and lower inpatient bed utilization in areas with higher vaccination coverage
- Established candidate thresholds, then tested to calibrate levels

Indicator Performance Analysis Results

Question	Answer
What is the appropriate outcome variable?	Deaths, with ICU bed utilization as a secondary indicator. Both are correlated with transmission levels and COVID-19 community levels.
What is the optimal lag between the community level/transmission level and the outcome?	Correlation with death rates for new cases, hospital admissions and bed utilization peaks when the lag is set at 3 weeks.
How do individual indicators such as admissions, inpatient bed utilization predict outcomes?	Individual indicators have moderate correlation with deaths/100k three weeks later (~0.3) at the county level and high correlation (~0.8) at the state level. COVID-19 community levels (county: 0.3, state: 0.7) have **higher** correlations with death rates than transmission levels (county: 0.2, state: 0.5)
Which scheme (transmission levels or COVID-19 community levels) is more useful for identifying regions that will experience severe outcomes?	COVID-19 community levels are a more effective categorization scheme for identifying regions that will experience high death rates 3 weeks later according to multiple metrics (correlation, AUROC).
Should the thresholds be adjusted in response to this analysis?	Adjusting thresholds shifts the balance between levels and more balanced categorizations are more informative. COVID-19 community levels result in more balanced categories/levels.

Do community transmission levels or COVID-19 community levels better predict deaths and ICU utilization in counties?

- Do higher transmission levels and higher COVID-19 community levels correspond to more severe outcomes 3 weeks later?
  - Multiple analyses using different indicator thresholds were conducted to optimize the levels. COVID-19 community levels provided consistently better prediction compared with community transmission.
  - Analyses used AUROC (area under receiver operating characteristic). This can be interpreted as the probability that given two randomly selected observations from different levels, the one with the more severe outcome comes from a higher transmission/COVID-19 community level. Data analyzed included historical data from March 2021-January 2022
  - A score of 0.5 would correspond to random guessing and a score of 1 would indicate that worse outcomes always correspond to higher COVID-19 community levels/transmission levels.
- COVID-19 community levels are better predictors of deaths and ICU utilization 3 weeks later than community transmission levels at the county level.
  - Analyses using AUROC, Spearman's correlation, and Pearson's correlation coefficient provide consistent results.
  - Analyses used 4-level schemes for COVID-19 community levels and then were pared down to 3 levels based on end-user feedback.

Indicator Thresholds were Further Refined

- Compared different combinations of thresholds
  - With/without case threshold, and with different case thresholds (100, 200, 500, 1000 cases/100,000/week)
  - Different levels of *new COVID-19 hospital admissions* and *inpatient beds occupied by COVID-19 patients*
- Optimized levels based on thresholds with consistently higher performance at predicting ICU bed utilization, deaths, new admissions, and inpatient bed use 3 weeks later

CDC's COVID-19 Community Levels and Indicators

**New Cases** (per 100,000 population in the last 7 days)	**Indicators**	**Low**	**Medium**	**High**
**Fewer than 200**	New COVID-19 admissions per 100,000 population (7-day total)	<10.0	10.0-19.9	≥20.0
	Percent of staffed inpatient beds occupied by COVID-19 patients (7-day average)	<10.0%	10.0-14.9%	≥15.0%
**200 or more**	New COVID-19 admissions per 100,000 population (7-day total)	NA	<10.0	≥10.0
	Percent of staffed inpatient beds occupied by COVID-19 patients (7-day average)	NA	<10.0%	≥10.0%

The COVID-19 community level is determined by the higher of the inpatient beds and new admissions indicators, based on the current level of new cases per 100,000 population in the past 7 days

COVID-19 community levels are better predictors of deaths and ICU utilization in communities

- The proposed COVID-19 community levels provide a **sizeable improvement** over the community transmission levels in identifying regions that will experience severe outcomes 3 weeks later
  - To prevent deaths and ICU bed use, COVID-19 community levels using new indicator metrics provide more robust measures
  - COVID-19 community levels result in more meaningful differences between categories

COVID-19 community levels on March 30, 2021 (post Alpha)

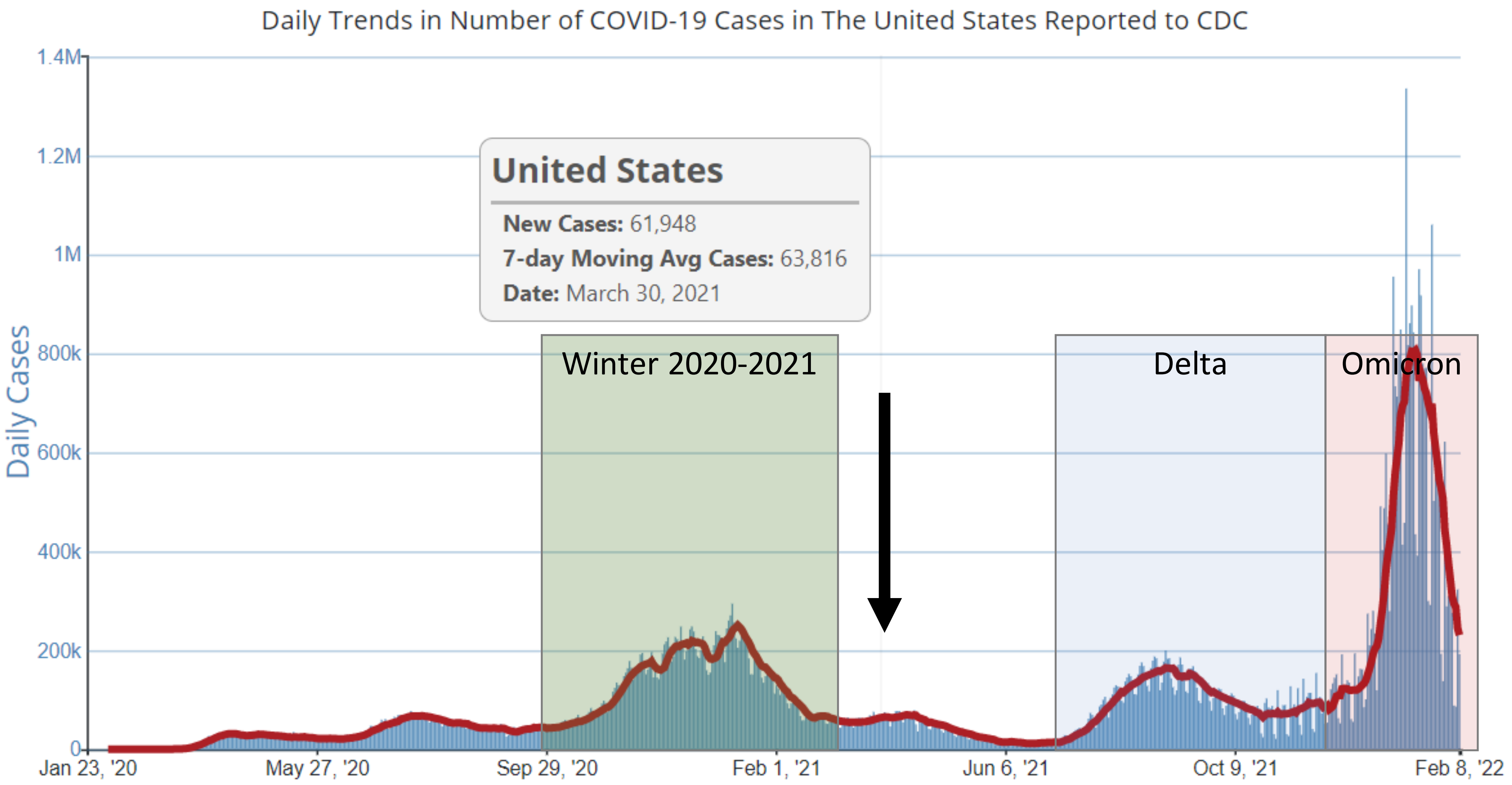

COVID-19 Community Levels on March 30, 2021

COVID-19 Community Level

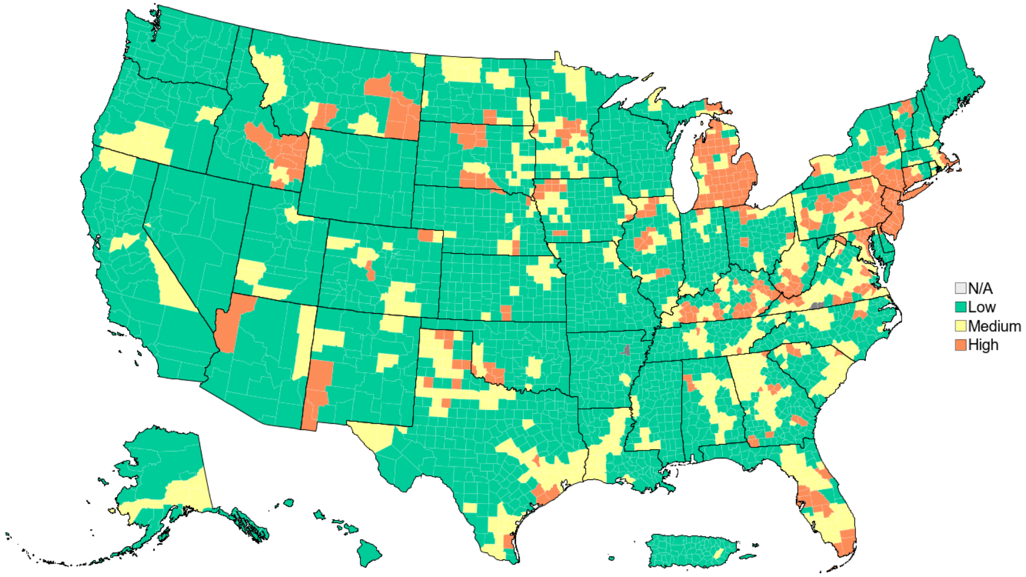

	% of Counties	% of Pop.
**Low**	**67.3%**	**56.9%**
**Medium**	**22.0%**	**23.4%**
**High**	**10.6%**	**19.7%**

Community Transmission

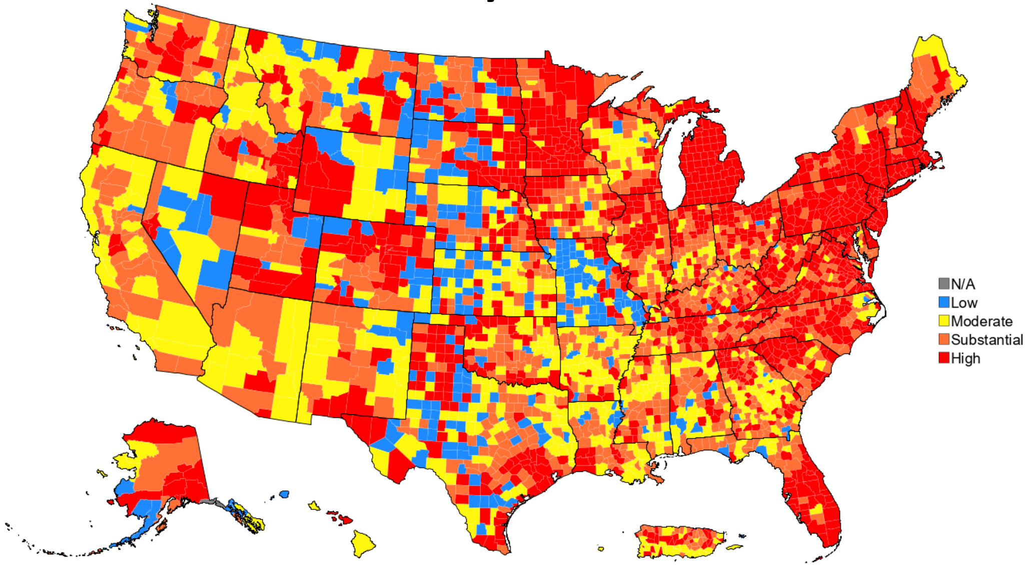

	% of Counties	% of Pop.
**Low**	**9.3%**	**1.4%**
**Moderate**	**22.0%**	**17.3%**
**Subst.**	**28.3%**	**26.4%**
**High**	**40.5%**	**54.9%**

COVID-19 community levels on July 30, 2021 (rise of Delta)

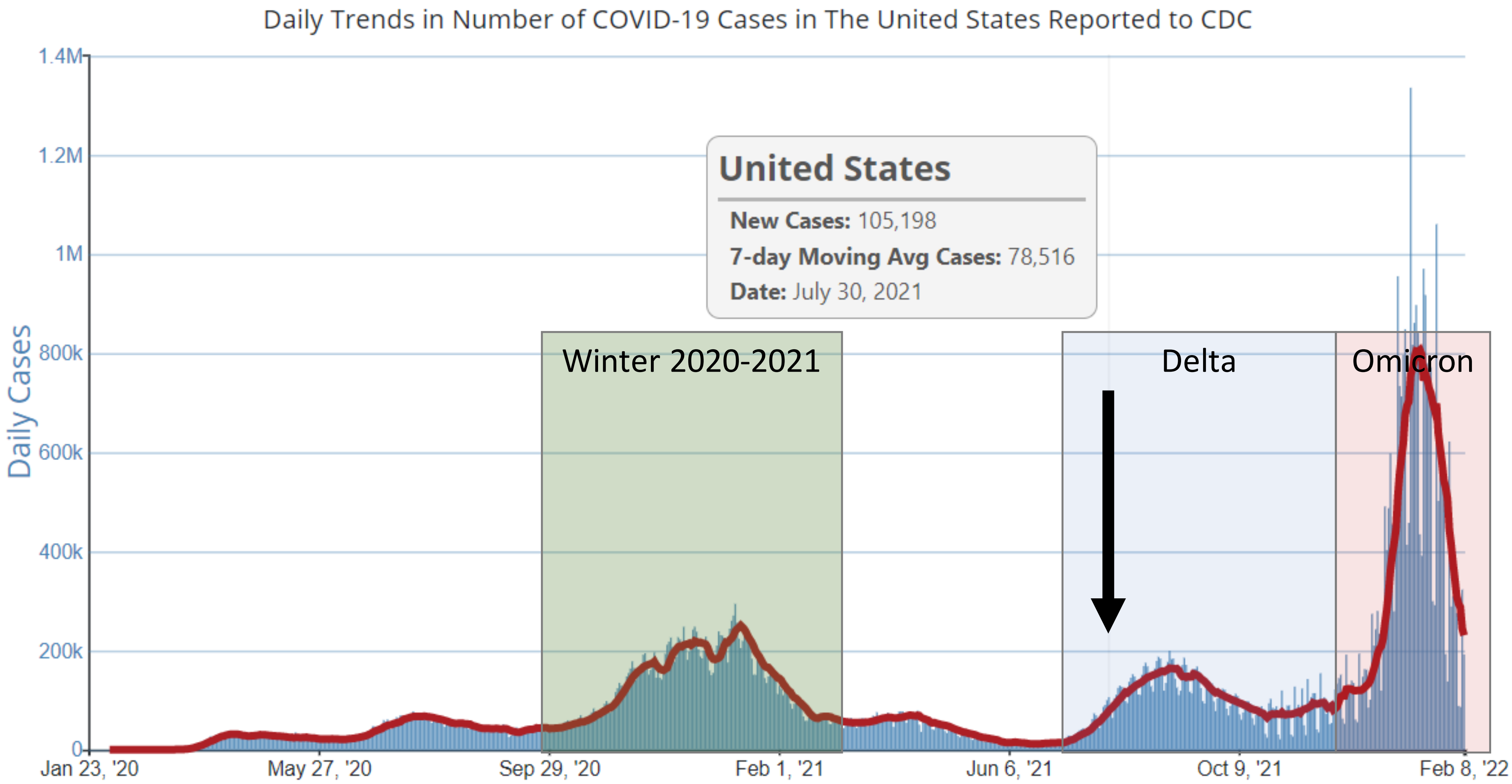

COVID-19 Community Levels on July 30, 2021

COVID-19 Community Level

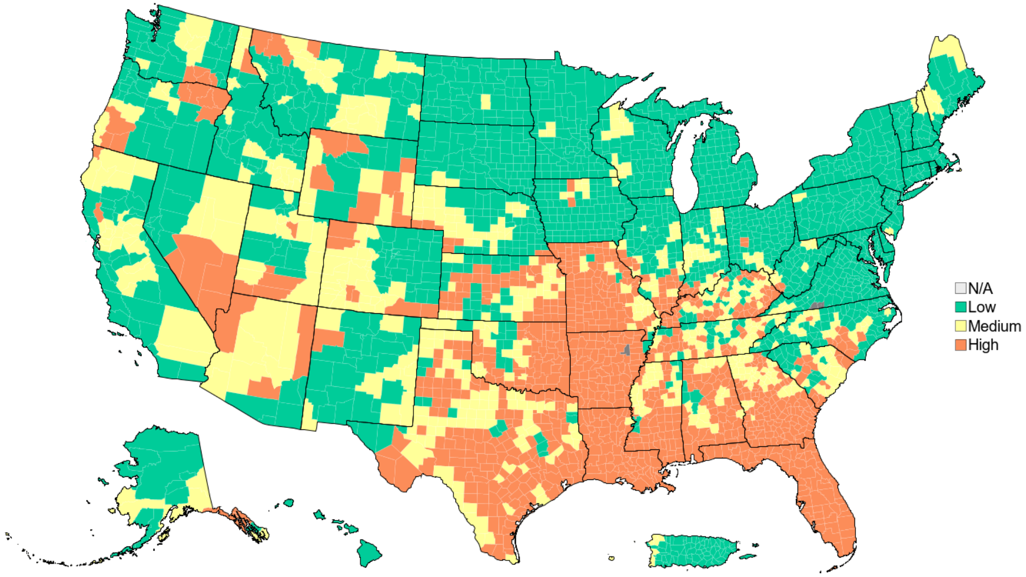

	% of Counties	% of Pop.
Low	49.6%	57.7%
Medium	20.2%	18.3%
High	30.1%	23.9%

Community Transmission

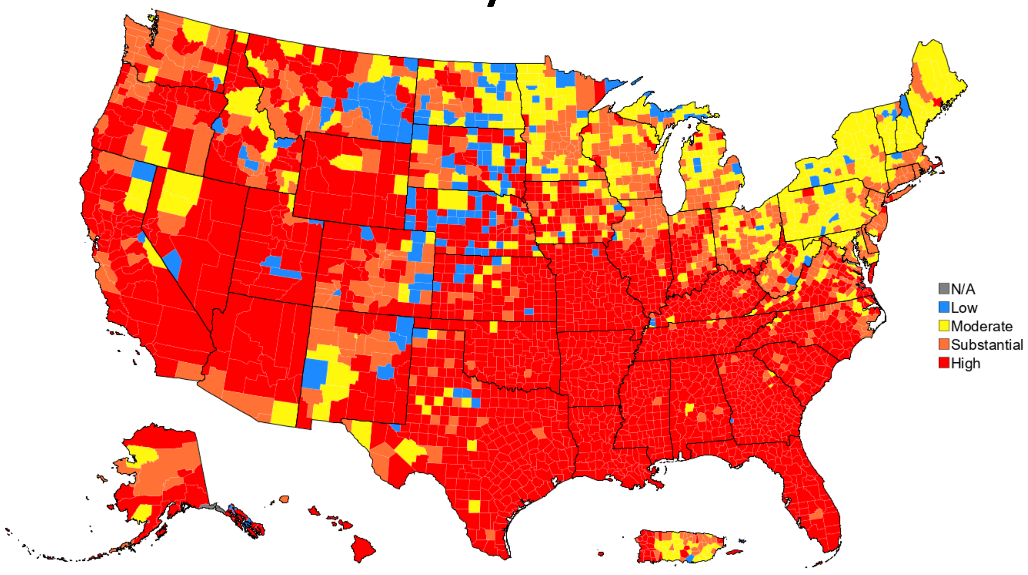

	% of Counties	% of Pop.
Low	4.8%	0.4%
Moderate	15.7%	12.1%
Subst.	18.2%	28.0%
High	61.3%	59.4%

COVID-19 community levels on September 3, 2021 (peak of Delta)

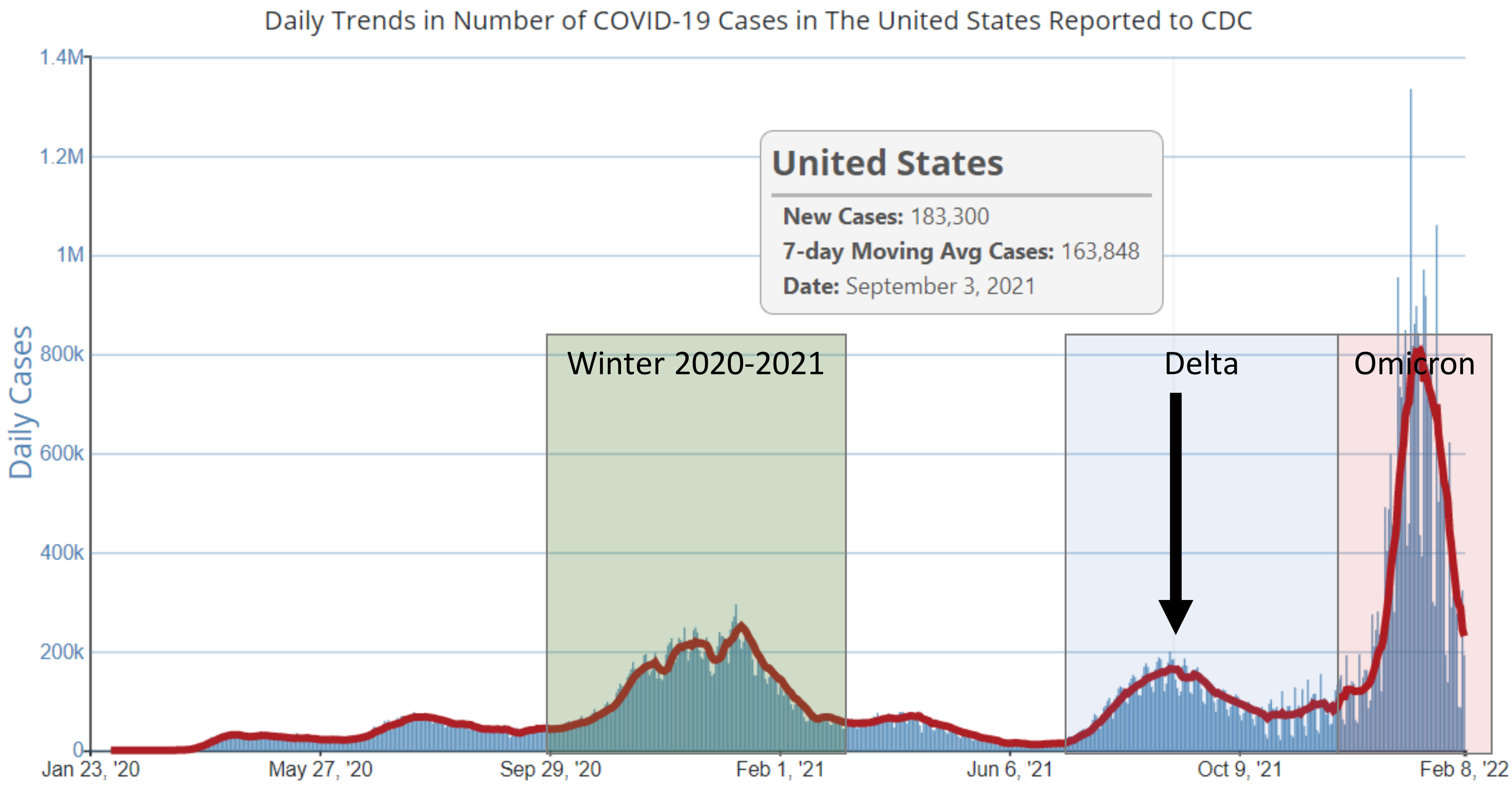

COVID-19 Community Levels on September 3, 2021

COVID-19 Community Level

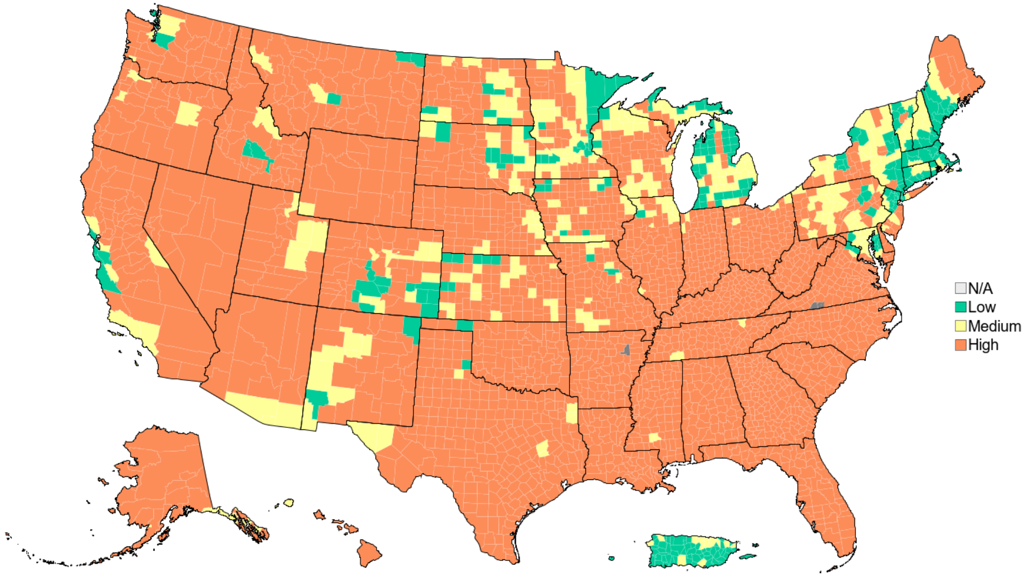

	% of Counties	% of Pop.
Low	8.1%	14.9%
Medium	12.2%	20.5%
High	79.6%	64.7%

Community Transmission

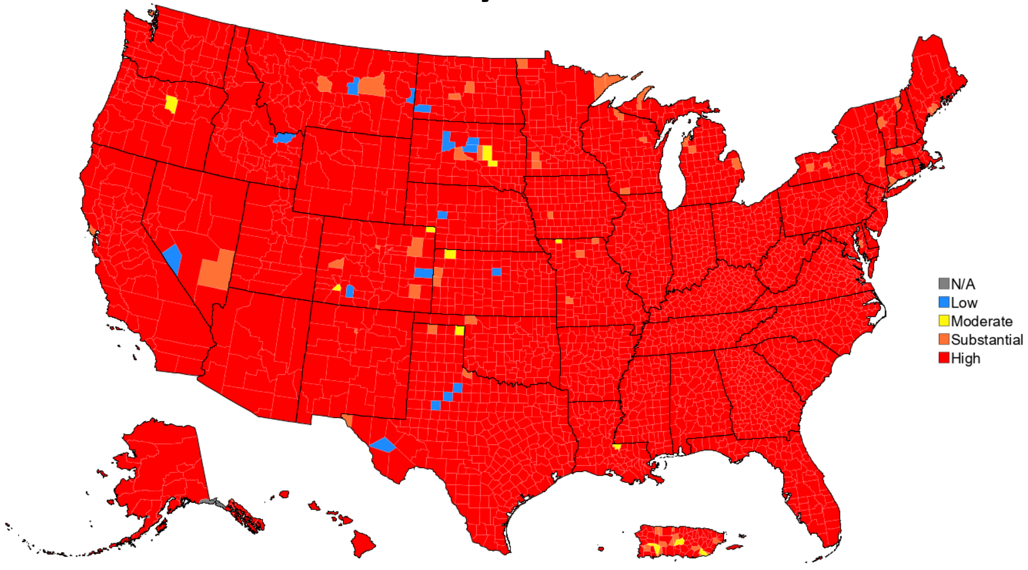

	% of Counties	% of Pop.
Low	0.5%	0.0%
Moderate	0.4%	0.0%
Subst.	2.0%	1.2%
High	97.0%	98.8%

COVID-19 community levels on November 5, 2021 (between Delta and Omicron)

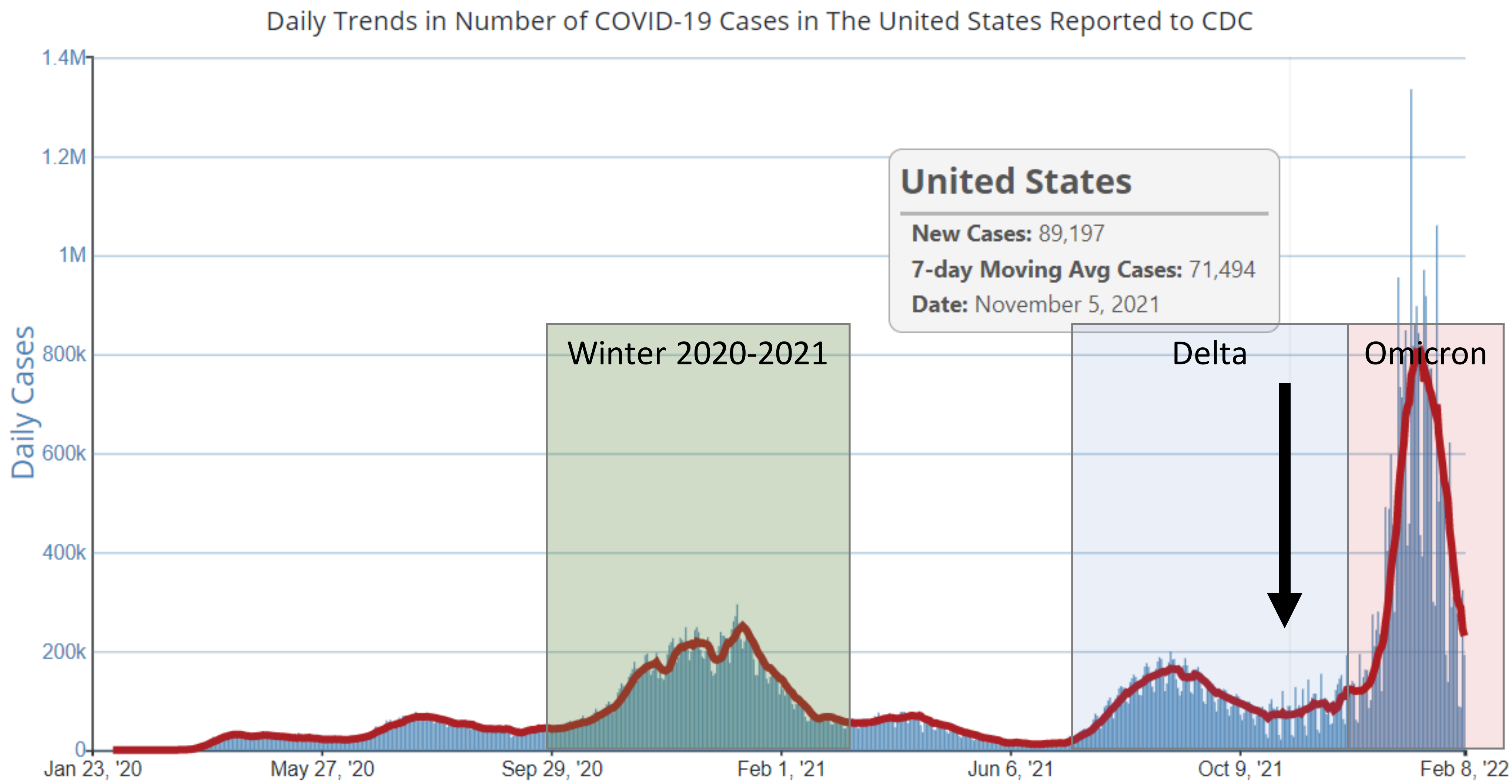

COVID-19 Community Levels on November 5, 2021

COVID-19 Community Level

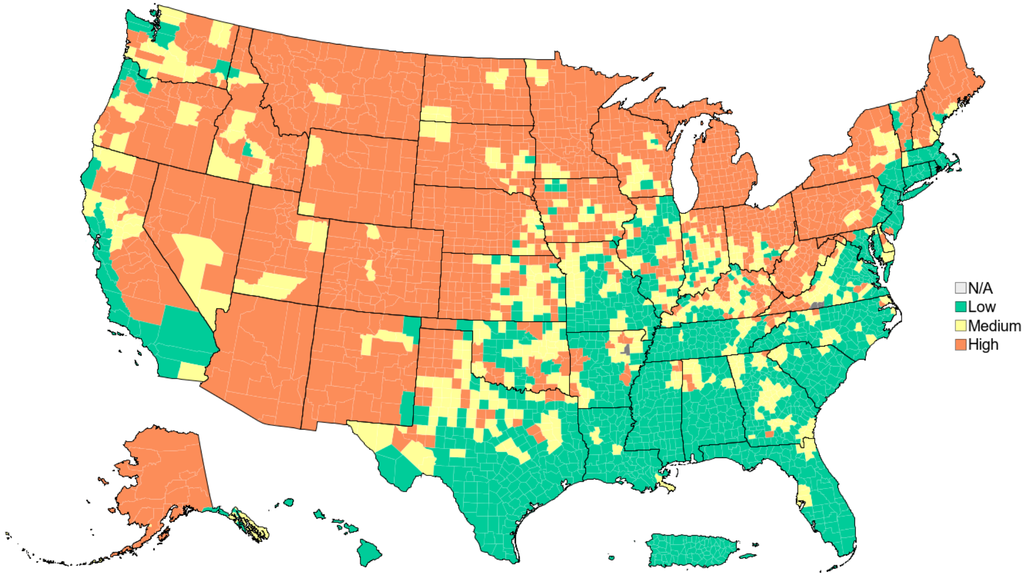

	% of Counties	% of Pop.
Low	38.3%	58.5%
Medium	21.5%	16.5%
High	40.1%	25.0%

Community Transmission

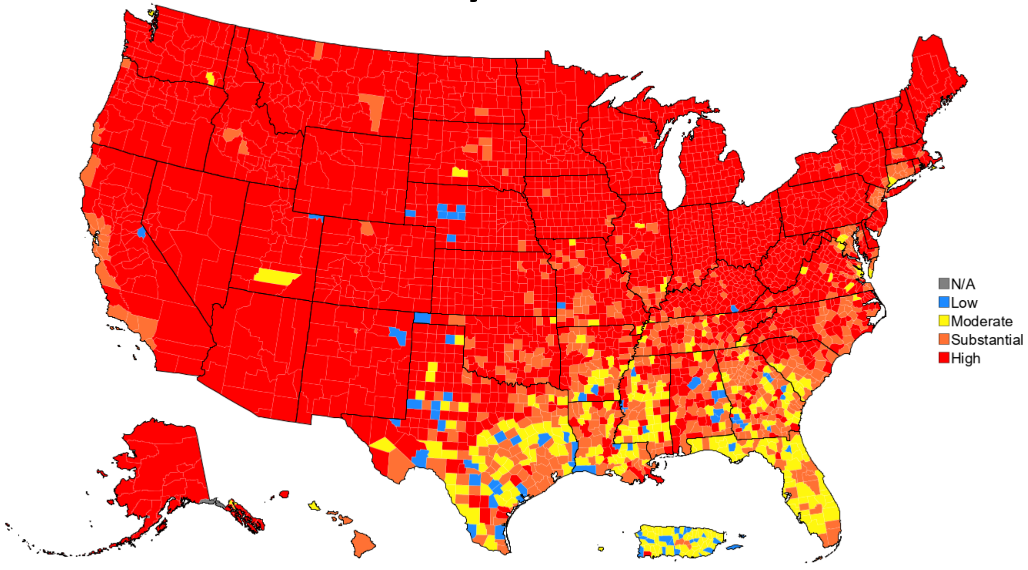

	% of Counties	% of Pop.
Low	2.6%	0.6%
Moderate	9.4%	8.6%
Subst.	16.8%	32.5%
High	71.2%	58.2%

COVID-19 community levels on January 15, 2022 (peak of Omicron)

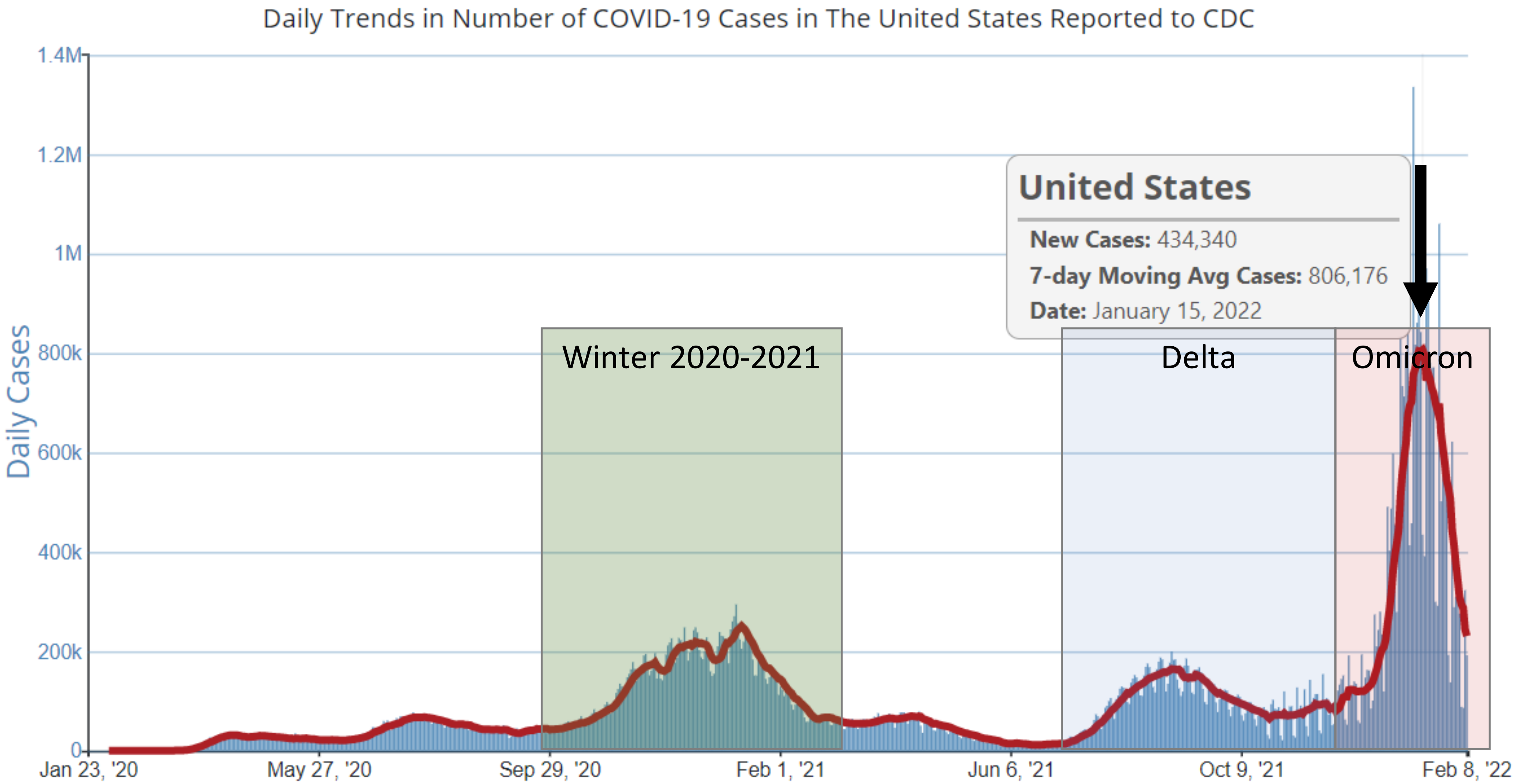

COVID-19 Community Levels on January 15, 2022

COVID-19 Community Level

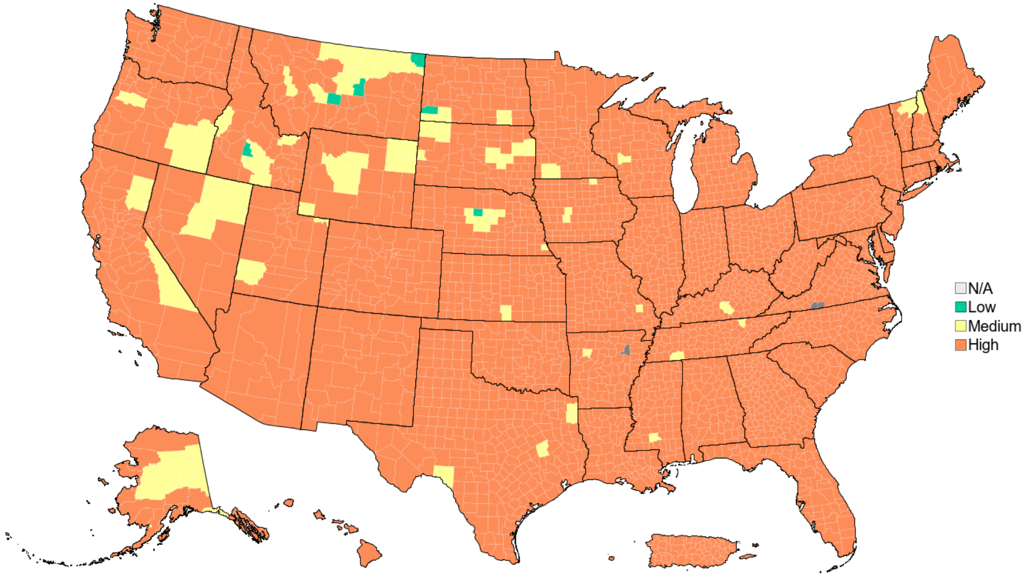

	% of Counties	% of Pop.
Low	0.2%	0.0%
Medium	3.2%	0.5%
High	96.5%	99.5%

Community Transmission

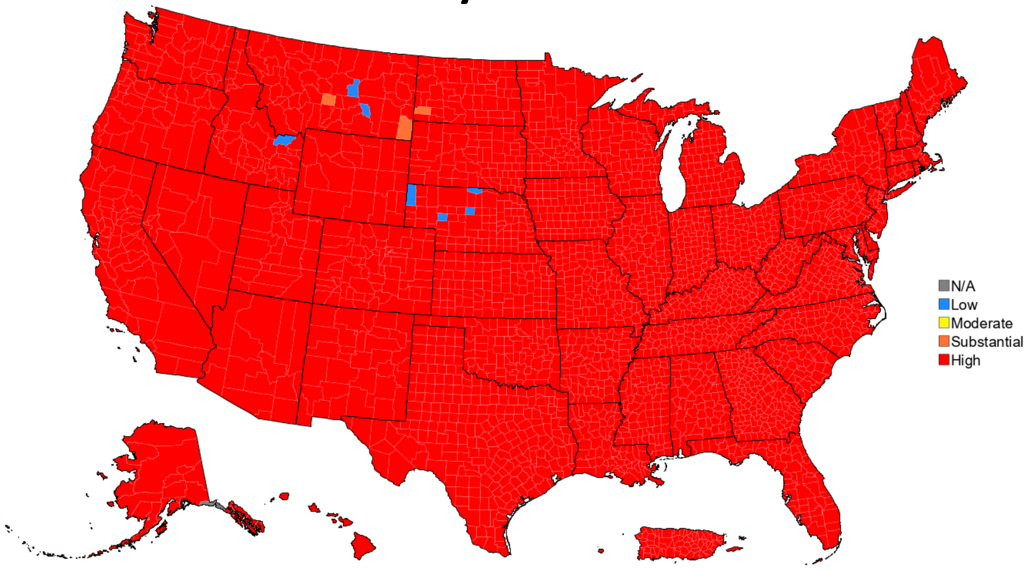

	% of Counties	% of Pop.
Low	0.3%	0.0%
Moderate	0.0%	0.0%
Subst.	0.1%	0.0%
High	99.6%	100.0%

Proposed Framework for Monitoring and Prevention

Community Metrics

COVID-19 Vaccination Coverage

- Overall coverage of people up to date
- Coverage among people at increased risk of severe illness and health equity

Inform

COVID-19 Community Indicators

- Healthcare strain
- Hospital admissions of severely ill patients
- New cases (leading indicator)

Inform

Local Metrics and Information

- Wastewater surveillance
- Circulating novel variants of concern
- Local high-risk congregate settings
- Upcoming large events
- Health equity

Inform

Higher vaccination coverage likely to result in lower community levels

Local metrics and information provide context to interpret community level

Community Actions

Vaccine Activities

- Outreach
- Campaigns
- Distribution
- Equity

Prevention Measures

- Masking
- Testing
- Other individual prevention behaviors
- Other community-level prevention strategies

Local Decisions

Local vaccine activities and recommended prevention measures for different community levels inform local decisions

Implications for Using COVID-19 Community Levels to Inform Public Health Recommendations

- COVID-19 community levels can inform recommendations for **community-level preventive strategies** and **individual preventive behaviors**
- At higher COVID-19 community levels recommendation would include:
  - Masking
  - Testing Strategies (e.g., screening testing)
  - High-risk individuals and their household or social contacts (e.g., masking, testing, and access to treatments)
  - Setting-specific recommendations (e.g., K-12 schools, healthcare)
  - High-risk congregate settings (e.g., masking and screening testing)

Key Considerations

- Vaccination is the leading public health prevention strategy to prevent severe disease and deaths from COVID-19.
- People who are up to date on vaccines have much lower risk of severe illness and death from COVID-19 compared with unvaccinated people.
- When making decisions about individual preventive behaviors and community prevention strategies in addition to vaccination, people and health officials should consider the COVID-19 community level.
- Health departments should consider health equity, and make use of other surveillance information (wastewater, ED surveillance, etc.), if available, to inform local decisions.
- Layered prevention strategies — like staying up to date on vaccines and wearing masks — can help prevent severe disease and reduce strain on the healthcare system.

COVID-19 community levels on February 24, 2022

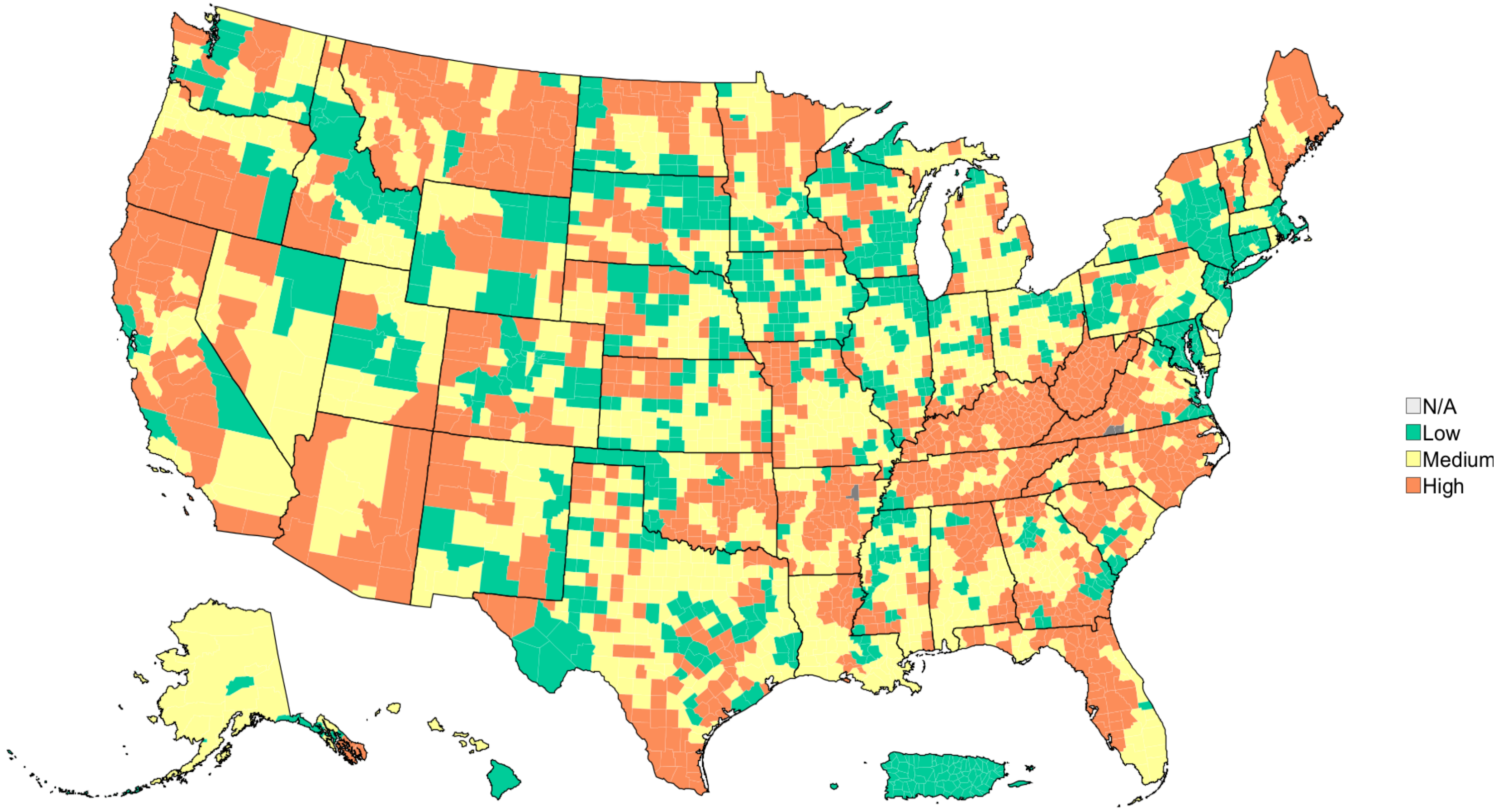

	% of Counties	% of Pop.
**Low**	**23.0%**	**29.5%**
**Medium**	**39.6%**	**42.2%**
**High**	**37.3%**	**28.2%**

Data sources and acknowledgments

- **Data sources**
  - Unified Hospital Data Surveillance System (UHDSS)
  - Aggregate Case and Death Counts (ACDC)
- **Acknowledgments**
  - Johns Hopkins University's Applied Physics Laboratory
  - CDC COVID-19 Response
